# In-silico detection of aneuploidy and chromosomal deletions in wheat using genotyping-by-sequencing

**DOI:** 10.1186/s13007-020-00588-3

**Published:** 2020-04-06

**Authors:** Narinder Singh, John Raupp, Dal-Hoe Koo, Bernd Friebe, Bikram Gill, Jesse Poland

**Affiliations:** 1 Wheat Genetics Resource Center, Department of Plant Pathology, Kansas State University, Manhattan, KS 66506 USA; 2 Present Address: Bayer U.S.–Crop Science, 700 Chesterfield Pkwy W, Chesterfield, MO 63017 USA

**Keywords:** Computational, Chinese Spring, Aneuploidy, Monosomic, Trisomic, Nullisomic, Chromosome deletion

## Abstract

**Background:**

Short read sequencing technologies, such as genotyping-by-sequencing (GBS), have been utilized in genetic mapping, marker development, and population genomic studies. High-throughput and multiplexing capability coupled with low cost make GBS an appropriate tool for molecular research. Here, we present the application of GBS to characterize wheat aneuploid stocks and detect chromosomal aberrations including aneuploidy and chromosomal deletions. These aneuploids are an important resource that have been used in wheat genetics and genomics studies to localize genes, determine physical positions, and develop chromosome bin maps.

**Results:**

Using GBS, we mapped sequence reads and quantified read coverage across chromosome bins. Using this approach, we confirmed known deletions and aneuploid stocks. In addition, we were also able to fully characterize these stocks and to identify several novel deletions and aneuploids. With this knowledge and a quick detection tool at our disposal, we can easily isolate these deletions and aneuploids into distinct lines.

**Conclusion:**

We envision this tool to replace the intensive cytogenetics techniques, such as C-banding, and fluorescent- and genomic-in situ hybridization to accurately detect chromosome dosage and segmental deletions in wheat genetic stocks as well as other crop species.

## Background

Wheat is a polyploid species that inherited its three different genomes from three distinct diploid species [1–4]. Unlike many species, wheat can tolerate chromosomal deletions due to its buffered polyploid nature. This has enabled the development of complete sets of chromosome aneuploid stocks and many chromosome deletion stocks [5–7]. These genetic stocks are an important resource for wheat genetics, which have been used extensively in genetic mapping, the development of genomic resources, and genome mapping [6, 8–11].

The maintenance of these genetic stocks requires extensive cytology to identify and confirm the chromosome deletion/aneuploidy. Therefore, the accurate characterization of the presence and dosage of chromosome deletions and aneuploidy in a high-throughput manner is an important objective for more efficient curation and utilization of these stocks. Subsets of these deletion and aneuploid stocks have been characterized before using C-banding, fluorescent- and genomic- in situ hybridization, and expressed sequenced tags (ESTs) [12–14]. However, the low-throughput, time intensiveness and limited resolution of these methods limit their application for large scale characterization of these stocks. We therefore approached the question if genotyping-by-sequencing approaches could be utilized for characterizing chromosome deletions and chromosome dosage in a high-throughput and low-cost manner.

Short-read sequencing technologies nowadays have become a mainstay for genomic studies in crop species due to their reducing cost and high-throughput. One such technology is genotyping-by-sequencing (GBS) that uses restriction enzymes to capture the reduced portion of genome for sequencing [15, 16]. GBS has been used for genome-wide single nucleotide polymorphisms (SNPs) discovery, genetic mapping, marker assisted selection (MAS), curating genebanks, population genomics, and genome-wide association studies (GWAS) [16–21]. Ability to multiplex samples combined with the high-throughput and low cost of GBS make it a robust tool for all these targets. We here present the application of low coverage GBS to detect terminal chromosomal deletions and check chromosome dosage of individual chromosomes and chromosome segments based on the read counts of GBS.

## Results and discussion

### Distribution of tag counts

**Fig. 1:**
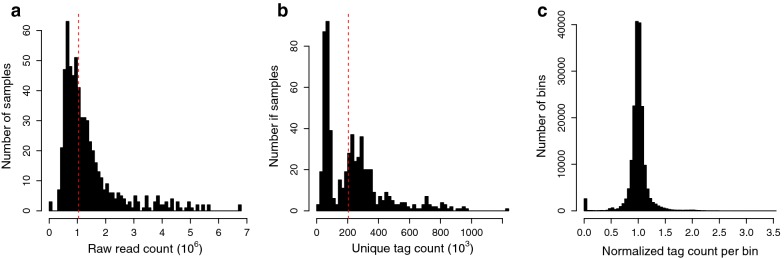
Histograms showing the distribution of **a** raw read count per sample, **b** unique tag count per sample, and **c** normalized tag count per bin relative to median. Vertical red dotted lines represent the median values

Genotyping-by-sequencing (GBS) resulted in a total of ~ 834 million 100 bp raw reads from 606 samples. Tassel5 GBSv2 pipeline was used to retrieve valid reads with unique barcode followed by an enzyme cut site and trim them to 64 bp in length (hereafter called tags). A total of 1,049,622 such unique tags were identified from 606 samples. Because of their short length, these tags tend to map at multiple positions. Therefore, to reduce the error due to multiple mapping, tags were filtered to retain only uniquely mapped tags, which resulted in a total of 480,204 unique tags. Further filtering was performed to remove those tags that mapped to unanchored scaffolds or had ambiguous mapping positions. This filtering resulted in a final count of 452,123 unique tags that were used for further analysis. The distribution of raw read count and unique tag count per sample is shown in Fig. 1a, b. Both raw read and tag counts per sample showed heavily skewed distributions with a median of ~ 1.37 million raw reads and 204,628 tags per sample, respectively.

**Fig. 2:**
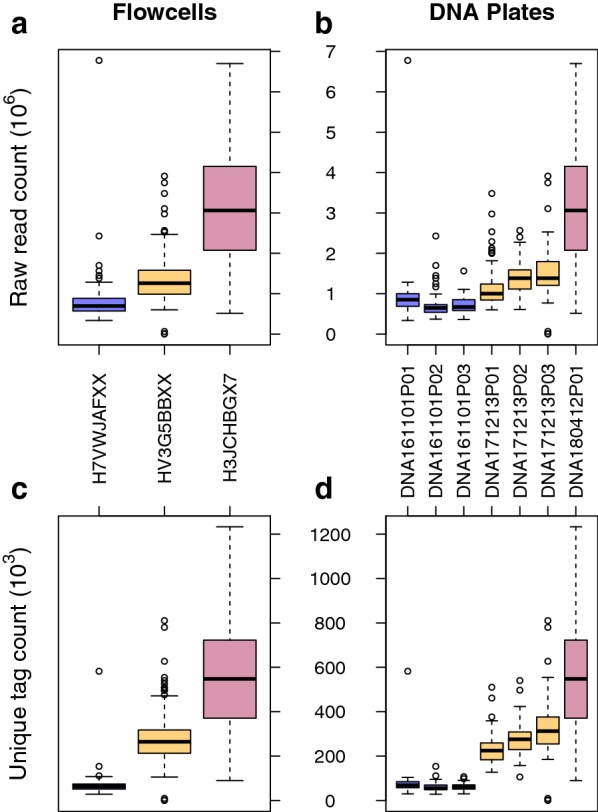
Boxplots showing distribution of **a** raw read count per sample across three flowcells, **b** raw read count per sample across seven DNA plates, **c** tag counts per sample across three flowcells, and **d** tag counts per sample across seven DNA plates. Same colors across flowcell and DNA plates means that the plates were sequenced on the corresponding flowcell

Tag count distribution showed a bimodal distribution, and we tested if this could be attributed to separate GBS runs. Samples in this study were collected in seven DNA plates at different times and sequenced on three different Illumina flowcells. Distribution of the counts within and across flowcells and DNA plates revealed that the different GBS runs contributed to the variability and skewness of the counts (Fig. 2). Latest GBS run (H3JCHBGX7) contributed the most reads and tags per sample, but had wider distribution, whereas other two previous runs had lower median counts but narrow distribution.

### Normalization of tag counts

Due to reduced representation and random sampling of genomic regions in the GBS, not all samples or all genomic regions within samples are sequenced at the same depth. Due to this differential in the read and tag counts, normalization across the genome was performed to allow comparison within and across samples. Firstly, total tag counts were normalized across samples such that all samples had the same number of tags. This normalization further allows the comparison of tag counts within each bin across samples (see “Methods” section). Then tag counts were normalized to median count for each bin across samples. Normalized tag counts per bin had a normal distribution (Fig. 1c). We observed a minor peak in the count of bins with zero tags per bin representing the deletion bins. Most of the bins had the normalized tag counts close to the median value of one, representing normal two copies of the respective chromosome or chromosome segment (dosage). Normalized tag counts around 0.5 represent 1x dosage, at 1.5 represent 3x dosage, and 2.0 is 4x dosage. On a whole chromosome level the dosage of 1x, 2x, 3x and 4x, would represent monosomic, normal disomic, trisomic and tetrasomic condition, respectively.

### Detection of chromosomal deletions and aneuploidy

**Fig. 3:**
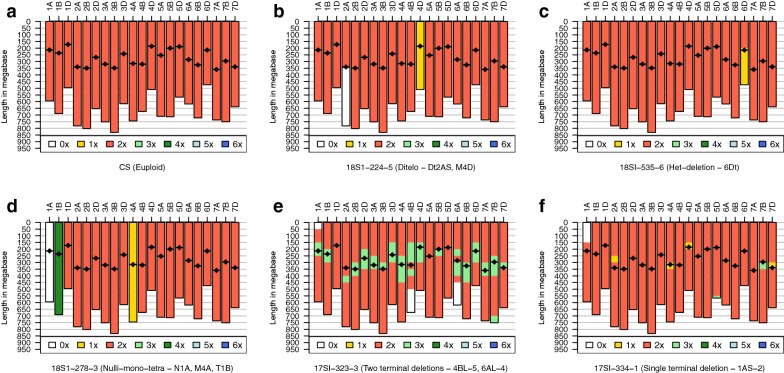
Karyotypes of reference Chinese Spring (CS) and other deletion and aneuploid stocks for comparison. X-axis on the top represent chromosome identifiers, y-axis is the length along the chromosome in megabase. Black lines with diamond represent centromeres. 0x, 1x, 2x, 3x, 4x, 5x, and 6x represents the dosage of a specific chromosome or chromosome segment, where 0x = no copy, 1x = single copy, 2x = 2 copies and so on

Sample karyotypes using normalized tag counts were plotted with R-programming language and analyzed visually for chromosome segment deletion or aneuploidy. Most of the samples (86%) had been characterized previously using traditional cytogenetic techniques, such as C-banding, and fluorescent- and genomic- in situ hybridization (FISH and GISH) [13, 22]. For almost all the previously characterized samples, we were able to confirm the known deletions. In addition, we also found several new deletions and previously undetected aneuploidy events. To show the range of karyotypes as a reference point, six distinct karyotypes, including the euploid Chinese Spring (CS) are shown in Fig. 3. As expected, CS did not show any deletion or aberration, and had two copies for all chromosomes (2x). Other examples include 18S1-224-5, which is double-ditelo for chromosome 2AS (0x), and monosomic for chromosome 4D (1x). Sample 18-SI-535-6 has a heterozygous deletion for the short arm of chromosome 6D around the centromere but has only one copy for the long arm of 6D (1x). Sample 18S1-278-3 is a complex line with three different chromosomal aberrations. This sample is lacking both copies of chromosome 1A (N1A; 0x) but has four copies of chromosome 1B (T1B; 4x). Furthermore, this sample is also monosomic for chromosome 4A (M4A; 1x).

Although we were able to identify majority of deletions and aberrations without any ambiguity, we did observe uncertainty in few samples, especially around the centromere (Fig. 3, sample 17SI-323-3). This is possibly due to the biased amplification of some sequences during polymerase chain reaction (PCR) as well as the methylation sensitive enzymes used for GBS, which have very few sites in the centromeric regions and would have much less sampling. Variation around the centromere should not be trusted at this time unless continually associated with a terminal aberration. However, it did not affect the detection of terminal deletions in the chromosomes as evident from the samples 17SI-323-3 and 17SI-334-1 in Fig. 3. Complete set of karyotypes for all other lines are presented in Additional file 1: Fig S1.

Additionally, there were several samples that produced unexpected karyotypes that were hard to interpret (Additional file 1: Fig S1). One such group of samples include 17SI-357-4 to 17SI-359-3 and 18S1-216-1 to 18S1-220-3. These karyotypes could be interpreted to have a single copy (hemizygous; 1x) deletions at almost all chromosomes, which does not seem plausible. All these samples had overlapping terminal deletions at chromosome 2BL (2BL-1 and 2BL-3). However, it is hard to say without further investigation if these terminal deletions cause these karyotypes because other samples with a different 2BL terminal deletions produced interpretable karyotypes, such as 17SI-362-2 (2BL-7). This could also possibly be attributed to sequencing and PCR bias as these samples had relatively lower but comparable number of tags to other representative samples. However, the total number of tags is just one measure of good sequencing and does not guarantee uniform coverage across genome. Other example includes 17SI-372-1 that had a terminal deletion on chromosome 3DS. Even with these anomalous karyotypes, using this method we were able to detect the known deletion(s) in these samples. Other anomalous samples include 18S1-284-4 and 18S1-289-5, however, these results could be confidently attributed to the low number of tags in these two samples, 1868 tags and 14 tags, respectively.

### Future refinement of the pipeline

This newly proposed pipeline provides an evidence that it can be applied to complement and/or replace current cytological methods to rapidly characterize, screen, and better understand the chromosomal aberrations in the genetic stocks. We envision that with the reducing cost of DNA sequencing and whole genome sequencing becoming the mainstay for genomic studies, implementation of low-coverage whole-genome re-sequencing will be the next improvement in the pipeline giving higher resolution and reducing the noise due to PCR bias and provide better estimates for the deletion sizes.

## Conclusions

We developed a high-throughput computational method to detect terminal chromosomal deletions and chromosomal aneuploidy in wheat genetic stocks using low cost genotyping-by-sequencing. This methodology has the potential to replace cytological techniques for high-throughput, rapid and efficient screening and characterization of genetic stocks. Implementing this method on a subset of genetic stock, we not only identified known deletions, but also found several new aberrations. These genetic stocks have helped geneticists map desired genes and develop reference genomes [8, 9], therefore their accurate characterization will facilitate the wheat improvement and pave the way towards greater food security.

## Materials and methods

### Genetic stock and tissue collection

Plant genetic stocks analyzed in this study include euploid Chinese Spring, 145 deletion lines, and six aneuploid lines of common wheat, which have been previously described [6, 7, 23] (Additional file 2: Table S1). Multiple plants for each line were planted in the greenhouse in 2 by 2 inches small pots and the tissue was collected from about 2 weeks old seedlings of individual plants. Due to variable number of planted seeds for each line, we had a total of 606 samples. About 5 cm of young leaf tissue was sampled from each plant and collected in 96-well tissue collection box. The tissue was stored at − 80 °C until DNA extraction.

### DNA extraction and genotyping

Tissue was lyophilized for 24–36 h and genomic DNA was extracted using Qiagen BioSprint 96 DNA Plant Kit (QIAGEN, Hilden, Germany), and quantified using Quant-iT™ PicoGreen^®^ dsDNA Assay Kit (ThermoFisher Scientific, Waltham, MA, USA). Genotyping was performed using genotyping-by-sequencing (GBS) following two enzyme technique [16]. GBS libraries were prepared in 384-plexing using two restriction enzymes, a rare cutter PstI and a frequent cutter MspI, with a common reverse adapter ligated, and sequenced on Illumina platform at McGill Univesity-Génome Quebec Innovation Centre (Montreal, Canada) facility.

### Sequence alignment

Using Tassel5 GBSv2 and ‘bowtie2’ pipeline, short reads from GBS FASTQ files were aligned against International Wheat Genome Sequencing Consortium’s RefSeq v1.0 assemble to find physical locations of tags (valid reads with unique barcode and enzyme cut site) [24–26]. The pipeline was run with default parameters and the following changes. Briefly, ‘bowtie2’ was run in multithreaded environment with ‘-end-to-end-D 20-R 3-N 0-L 10-i S,1,0.25′ parameters. The resulting sequence alignment map (SAM) file was filtered using unix ‘grep’ function and ‘XS:i’ flag to retain only uniquely mapped reads. Filtered SAM file was used in the further steps of the pipeline to get tags by taxa distribution across samples. The full shell script used for alignment is available at GitHub link below.

### Tag counts distribution and normalization

Each chromosome was divided into 100 Mb bins and total number of tags were counted within 100 Mb bin with a sliding step size of 50 Mb. To remove the bias due to differential sequencing depth and to compare tag counts within and across samples, normalization was performed across genome for each bin for all samples. Tag counting and normalization was performed in R-programming language using base functions, and packages data.table and dplyr [27].

### Karyotype visualization

Sample karyotypes were plotted in R-programming language using the base ‘barplot’ function and ggplot2 package [27]. 50 Mb bins were categorized into separate dosage groups based on their normalized values. Normalized values were assigned to the respective copy numbers as follows: 0–0.25 as null, 0.25–0.75 to 1, 0.75–1.25 to 2, 1.25–1.75 to 3, 1.75–2.25 to 4, 2.25–2.75 to 5, and 2.75 or greater to 6 + copies. Distinct colors were assigned to each bin for dosage ranging from null (deletion; 0x) to 6x. Individual karyotypes were analyzed visually to detect chromosomal deletions and aneuploidy.

## Supplementary information

**Additional file 1: Figure S1.** Karyotype visualizations for all samples included in this study.

**Additional file 2: Table S1.** List of accessions (samples) analyzed in this study.

## Data Availability

Sequence files are available at NCBI SRA accession number PRJNA609117. The analysis and shell scripts with other required files are available at https://github.com/cropgen/Code_Aneuploidy_Deletions.
