# Supplementary material for: In-silico detection of aneuploidy and chromosomal deletions in wheat using genotyping-by-sequencing
Source: Plant Methods. 2020 Apr 6;16:45. doi: 10.1186/s13007-020-00588-3 (PMC7137276; doi:10.1186/s13007-020-00588-3)
Supplement: Supplementary file 1 — Additional file 1: Figure S1. Karyotype visualizations for all samples included in this study. [file 13007_2020_588_MOESM1_ESM.pdf]

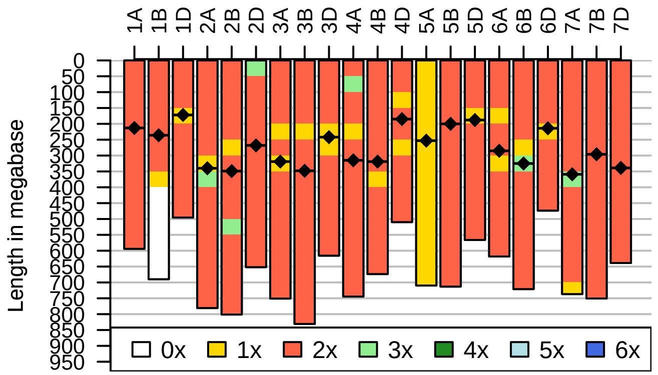

17SI−310−1 (1BL−6)

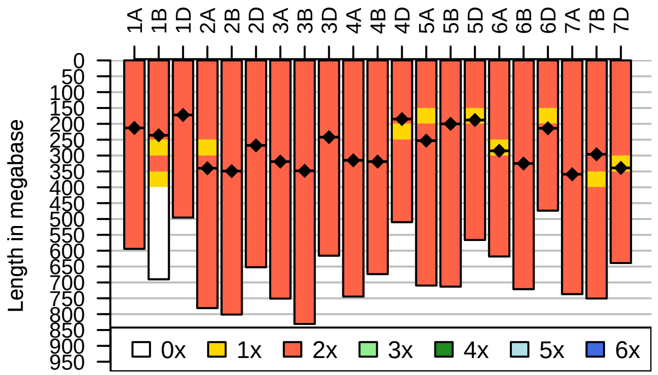

17SI−310−2 (1BL−6)

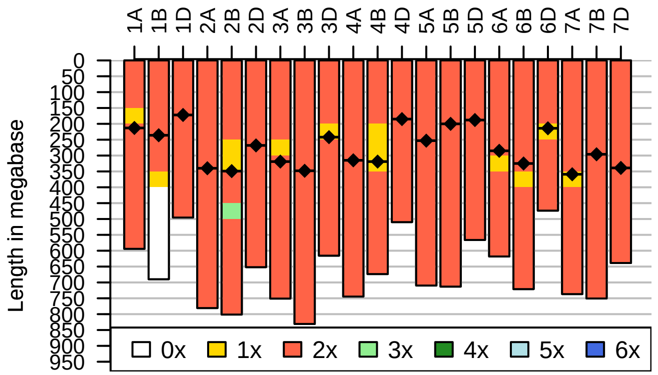

17SI−310−3 (1BL−6)

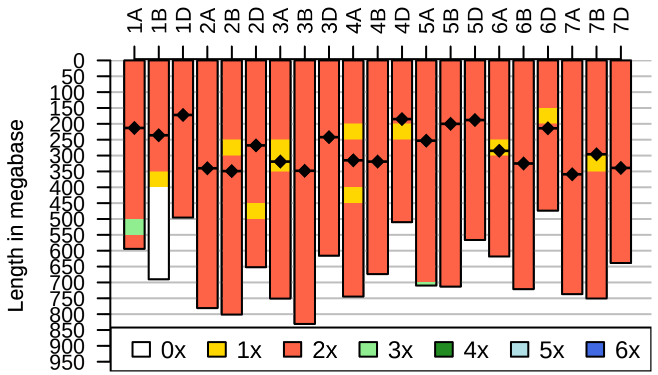

17SI−311−1 (1BL−6)

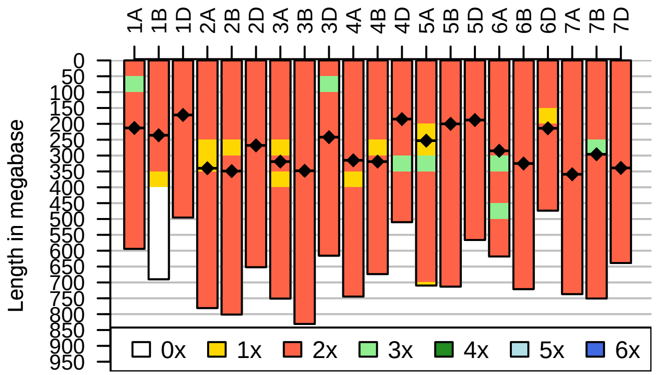

17SI−311−2 (1BL−6)

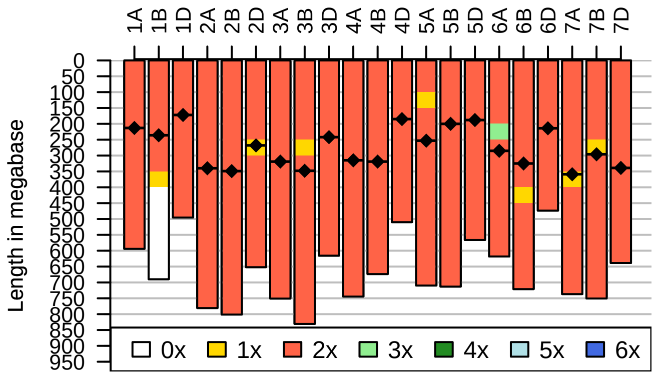

17SI−311−3 (1BL−6)

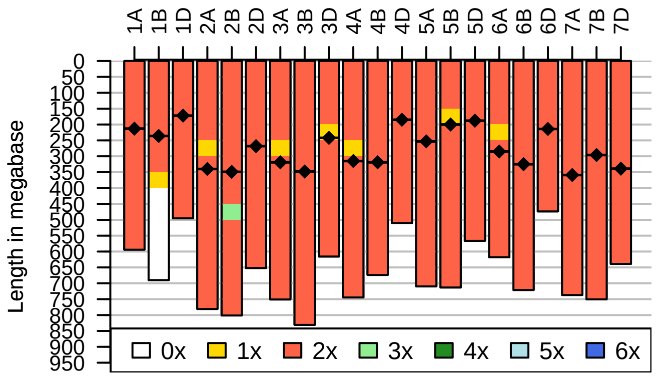

17SI−311−4 (1BL−6)

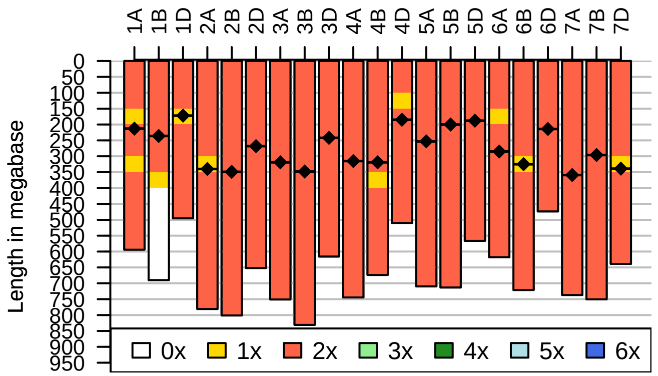

17SI−312−1 (1BL−6)

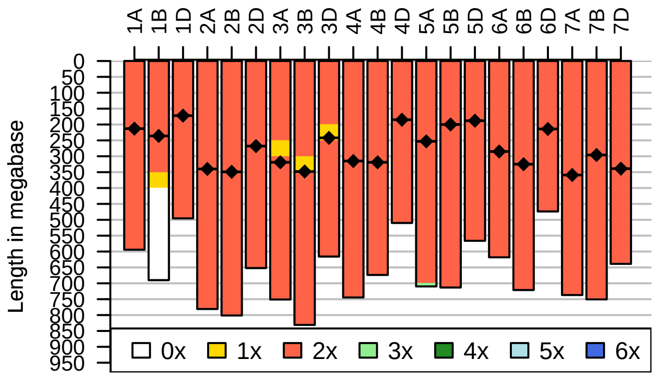

17SI−312−2 (1BL−6)

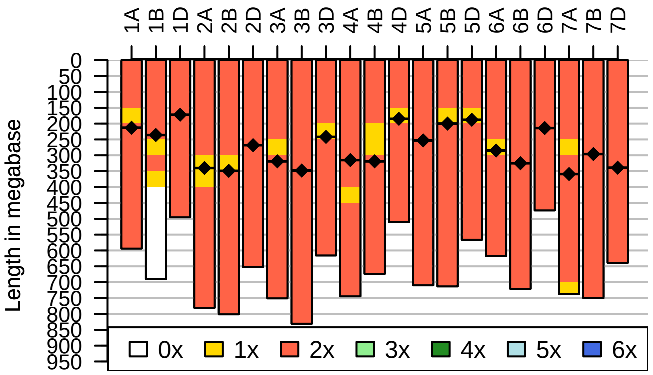

17SI−312−3 (1BL−6)

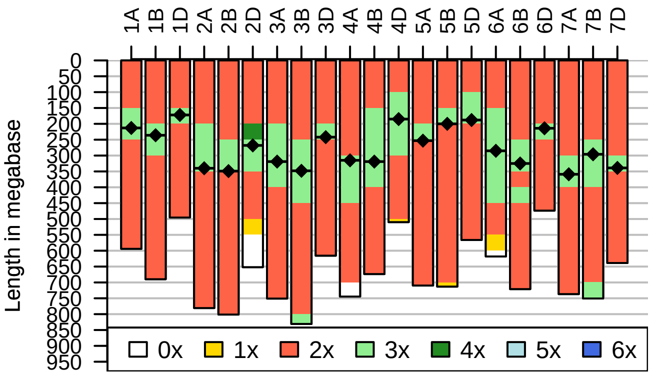

17SI−313−1 (2DL−9)

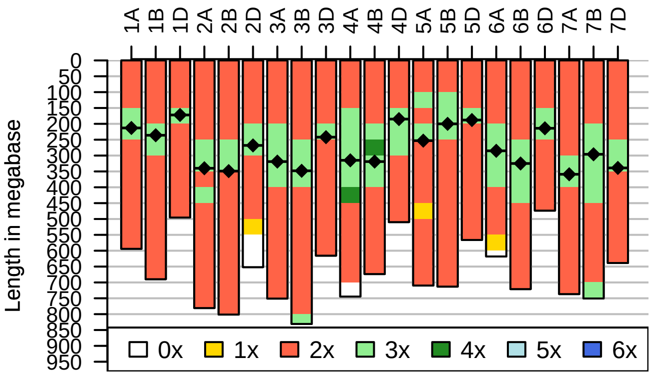

17SI−313−2 (2DL−9)

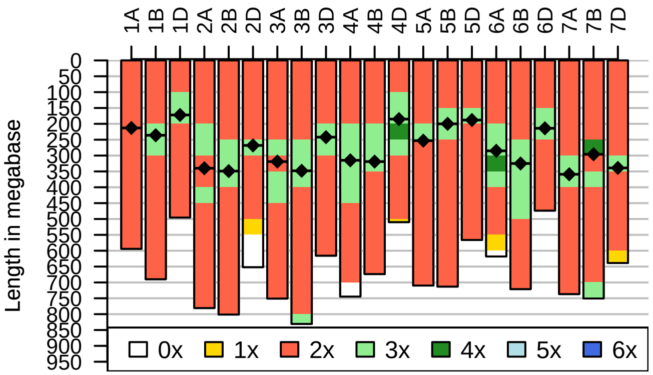

17SI−313−3 (2DL−9)

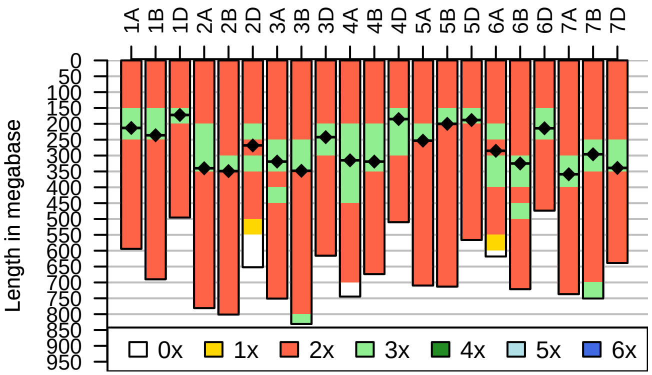

17SI−314−1 (2DL−9)

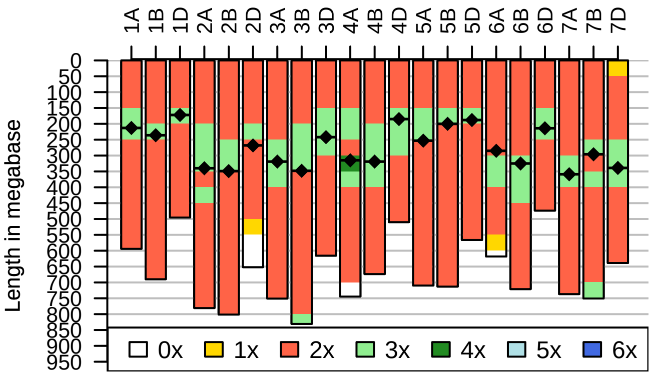

17SI−314−2 (2DL−9)

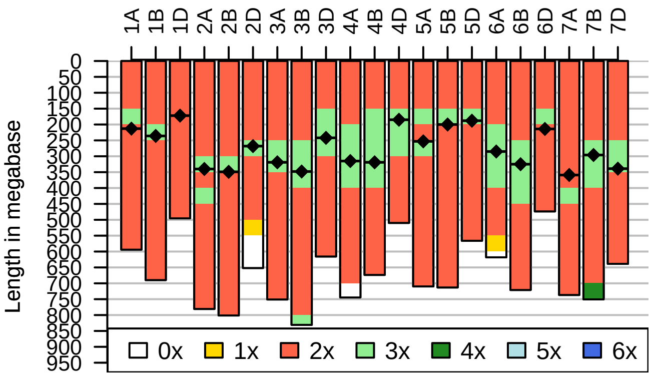

17SI−314−3 (2DL−9)

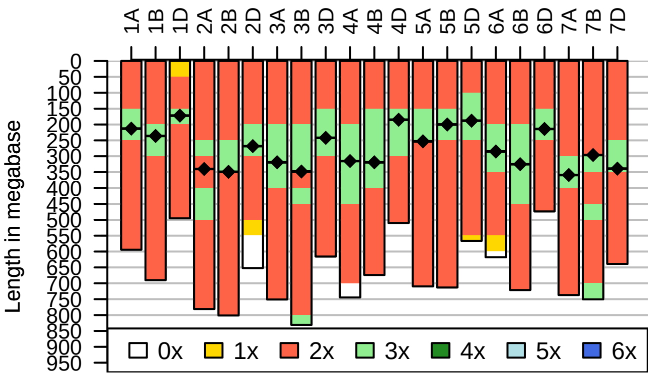

17SI−314−4 (2DL−9)

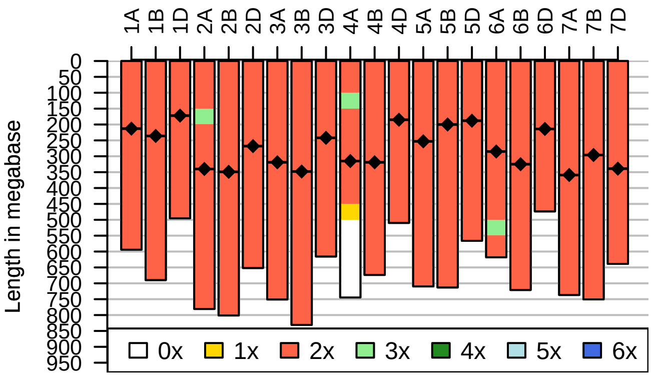

17SI−315−1 (4AL−12)

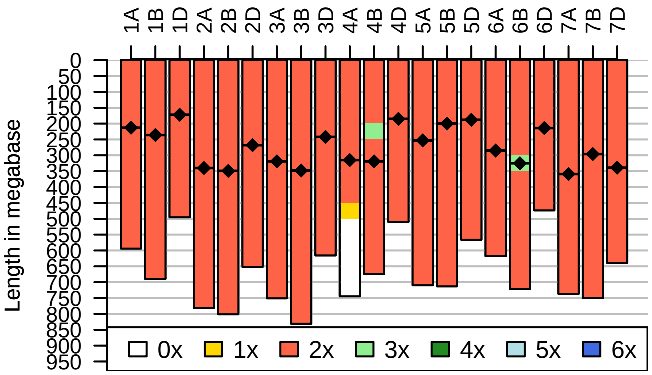

17SI−315−2 (4AL−12)

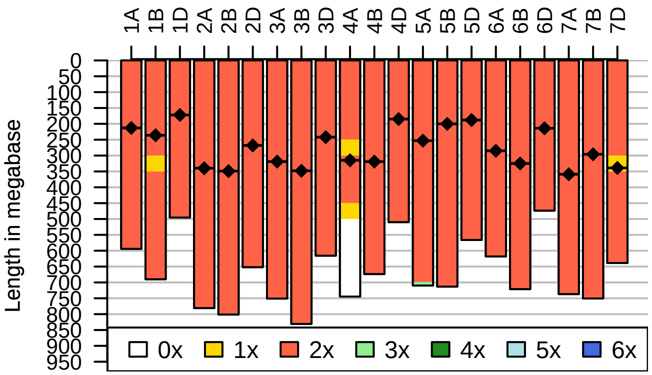

17SI−315−3 (4AL−12)

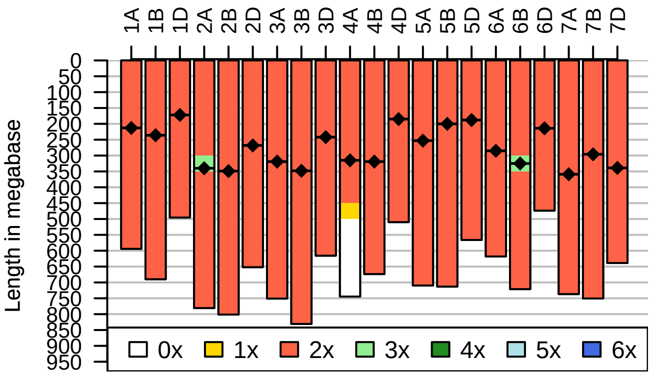

17SI−315−4 (4AL−12)

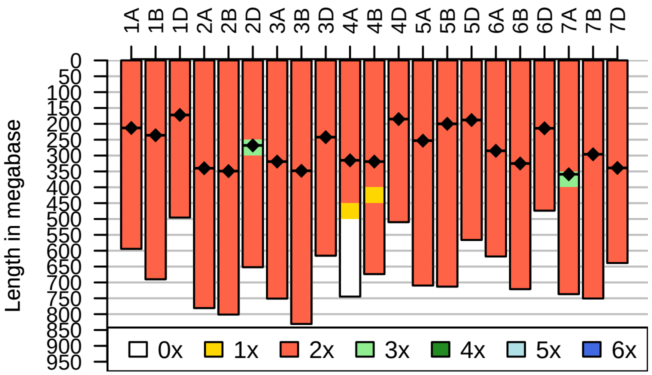

17SI−317 (4AL−12)

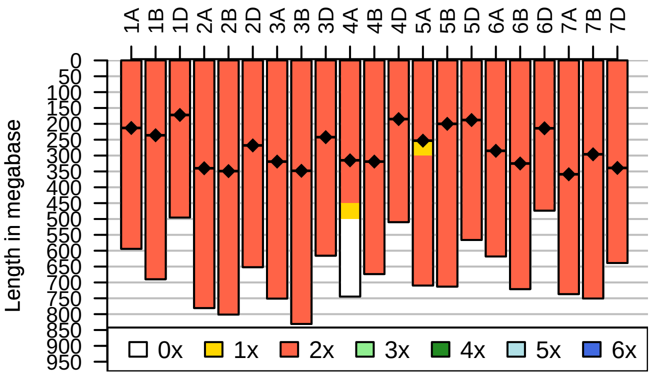

17SI−318−1 (4AL−12)

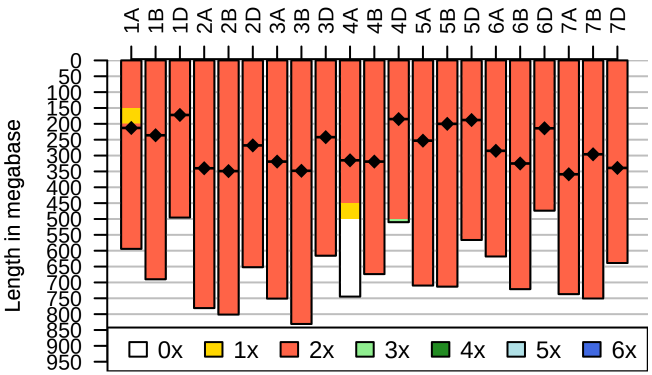

17SI−318−2 (4AL−12)

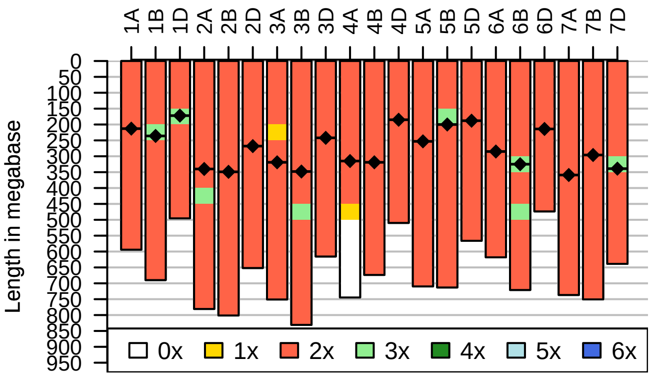

17SI−318−3 (4AL−12)

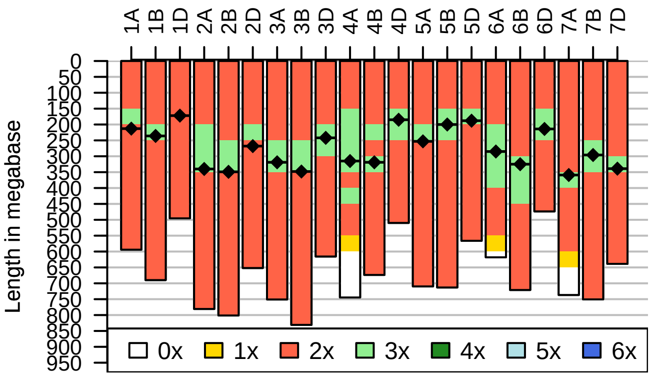

17SI−319−1 (4AL−13)

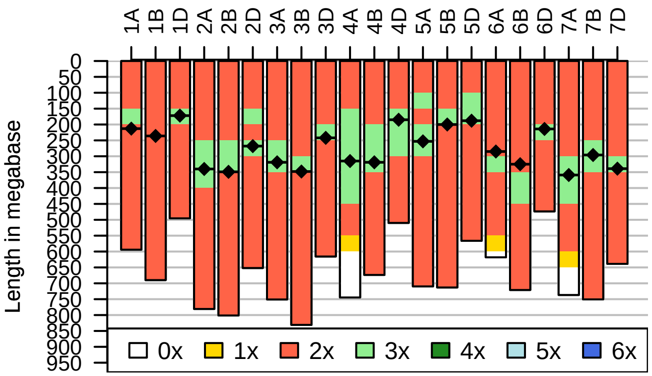

17SI−319−2 (4AL−13)

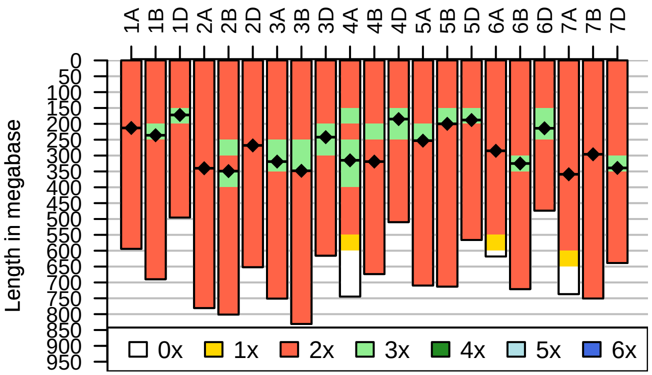

17SI−319−3 (4AL−13)

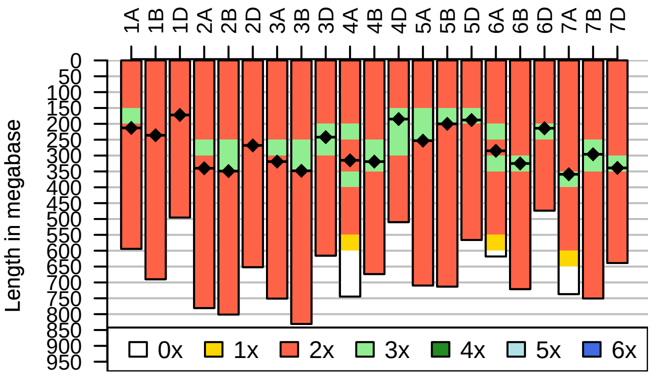

17SI−320−1 (4AL−13)

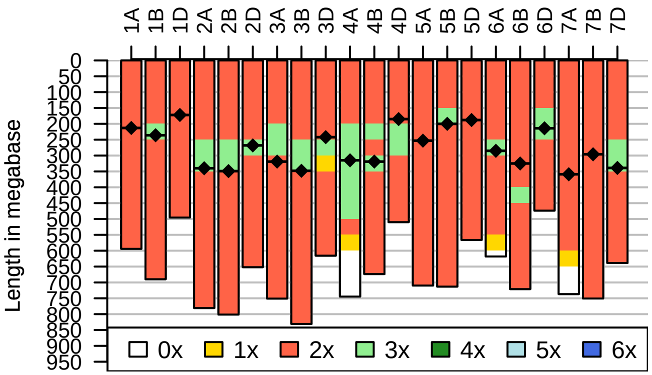

17SI−320−2 (4AL−13)

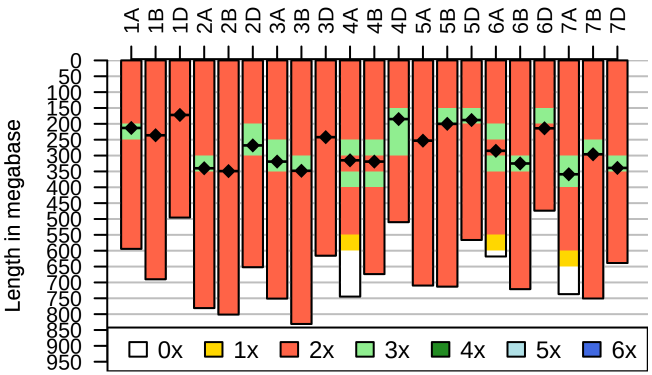

17SI−320−3 (4AL−13)

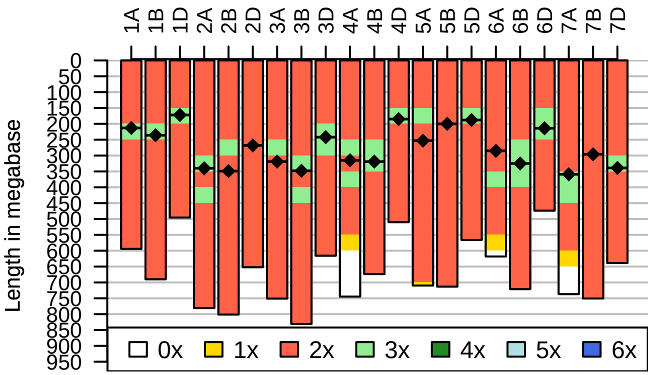

17SI−320−4 (4AL−13)

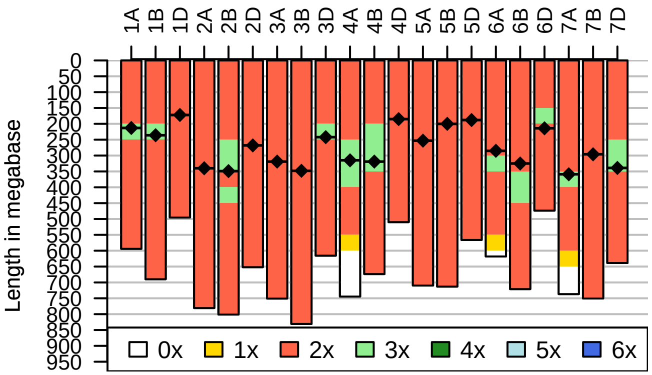

17SI−320−5 (4AL−13)

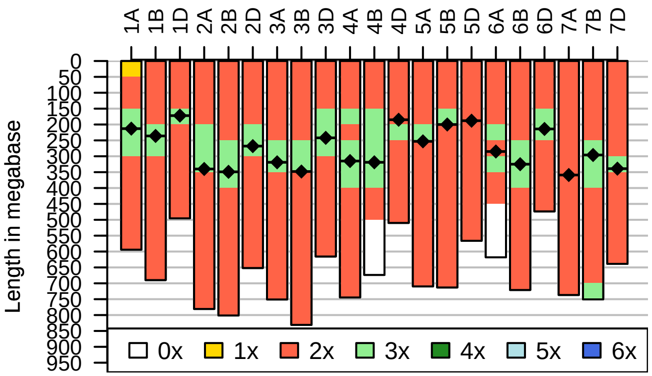

17SI−321−1 (4BL−5, 6AL−4)

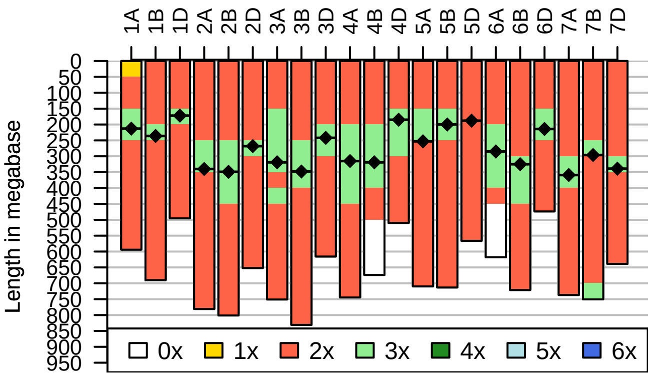

17SI−321−2 (4BL−5, 6AL−4)

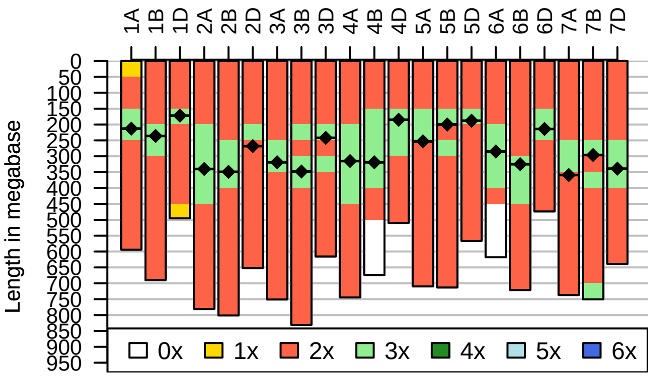

17SI−321−3 (4BL−5, 6AL−4)

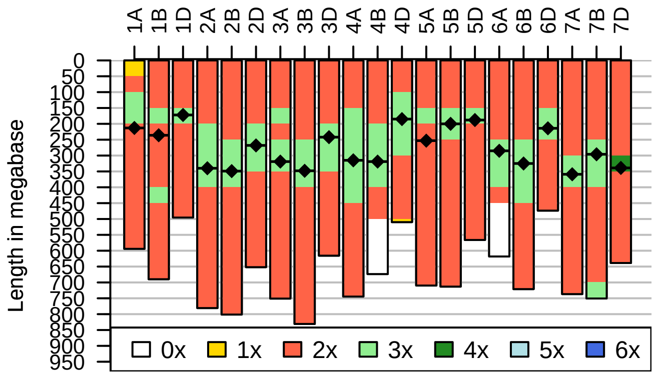

17SI−321−4 (4BL−5, 6AL−4)

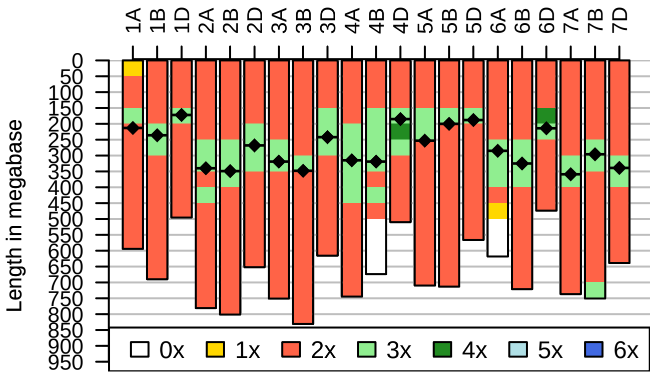

17SI−321−5 (4BL−5, 6AL−4)

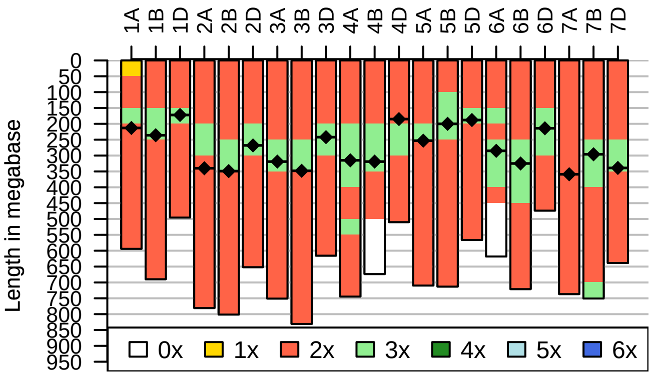

17SI−321−6 (4BL−5, 6AL−4)

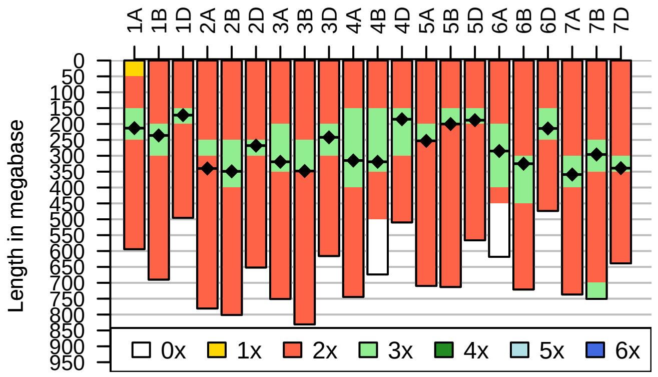

17SI−322−1 (4BL−5, 6AL−4)

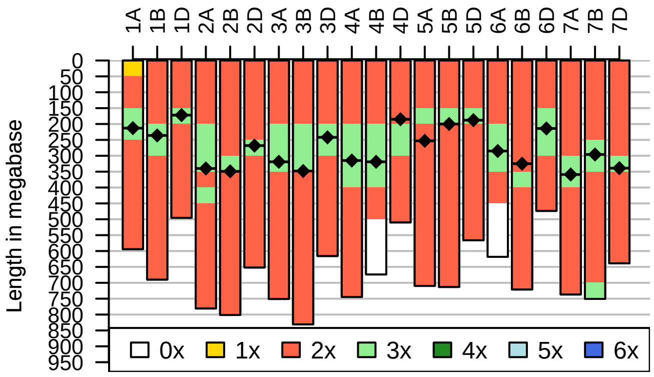

17SI−322−2 (4BL−5, 6AL−4)

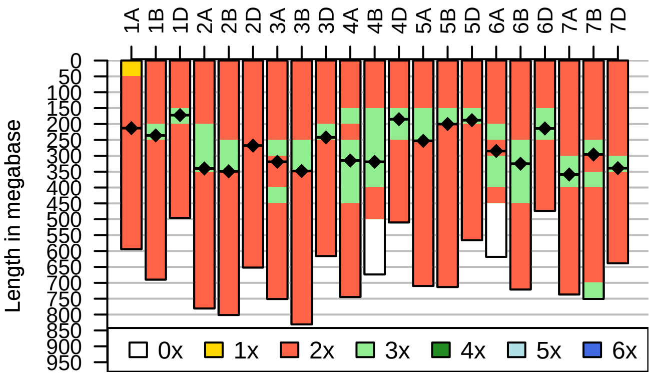

17SI−323−1 (4BL−5, 6AL−4)

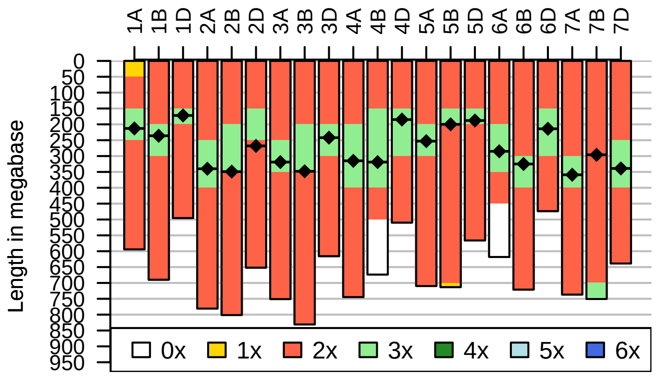

17SI−323−2 (4BL−5, 6AL−4)

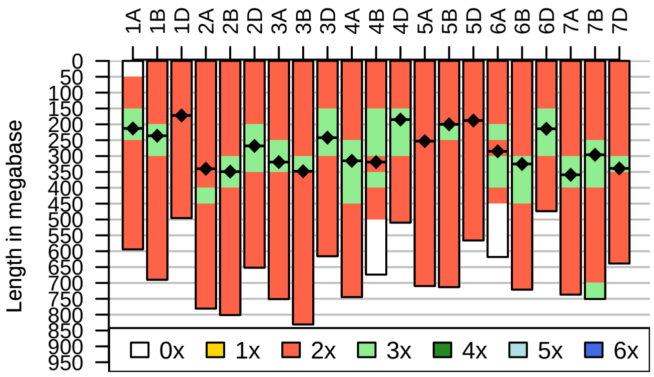

17SI−323−3 (4BL−5, 6AL−4)

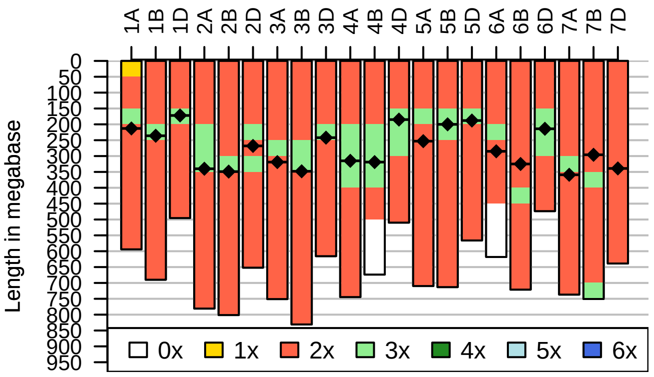

17SI−323−4 (4BL−5, 6AL−4)

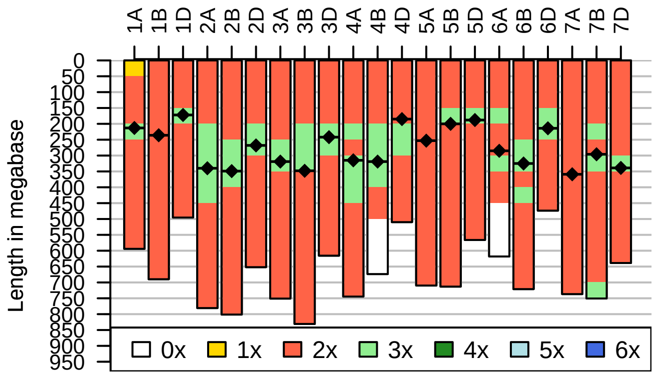

17SI−323−5 (4BL−5, 6AL−4)

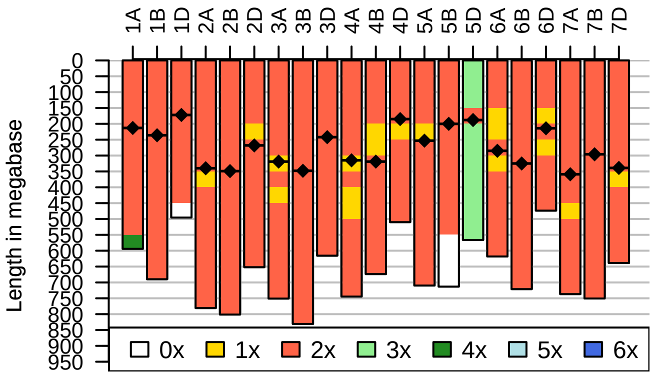

17SI−324−1 (5BL−9)

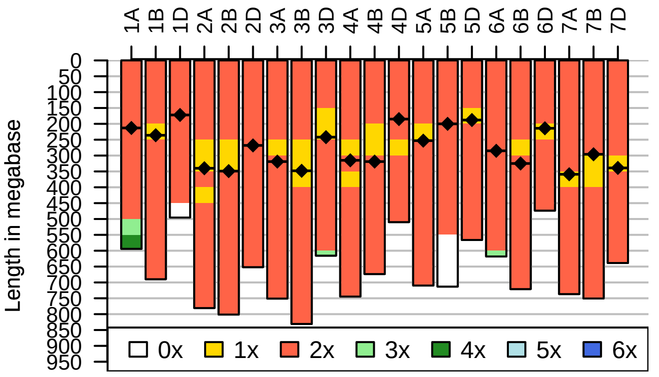

17SI−324−2 (5BL−9)

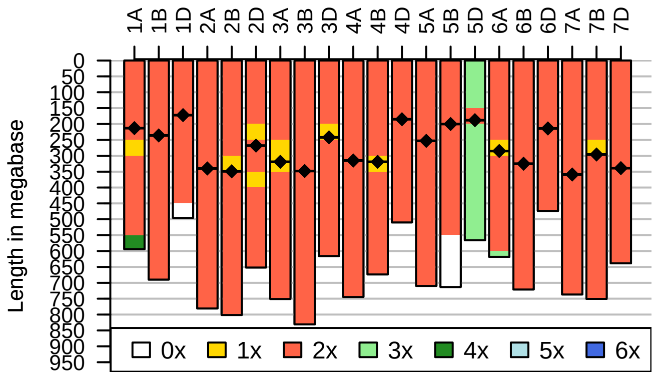

17SI−324−3 (5BL−9)

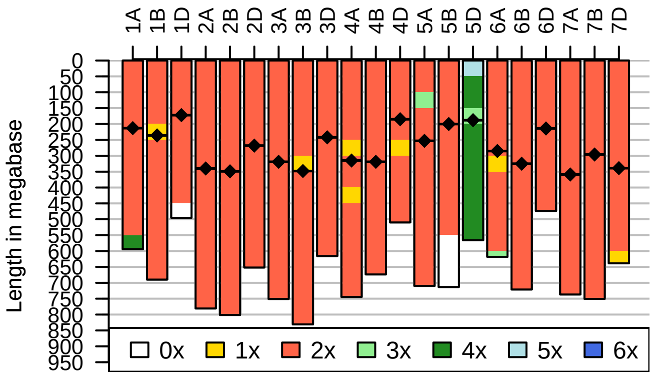

17SI−325−1 (5BL−9)

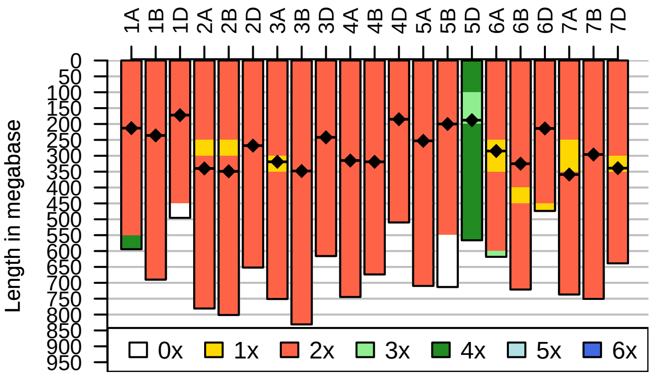

17SI−325−2 (5BL−9)

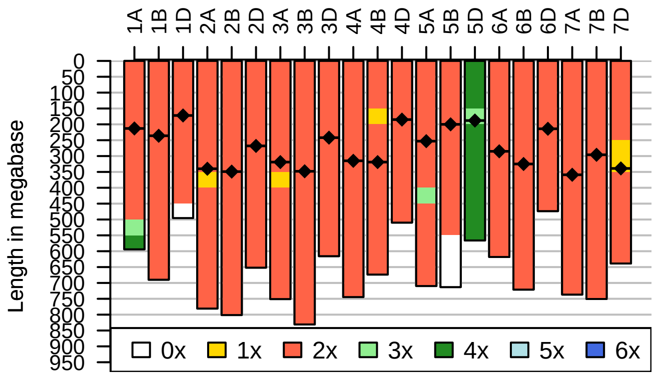

17SI−325−3 (5BL−9)

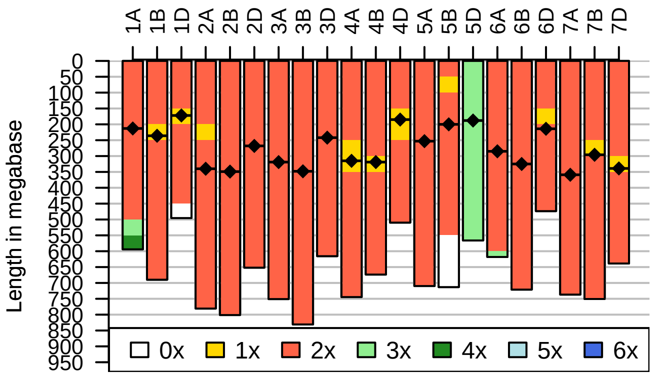

17SI−325−4 (5BL−9)

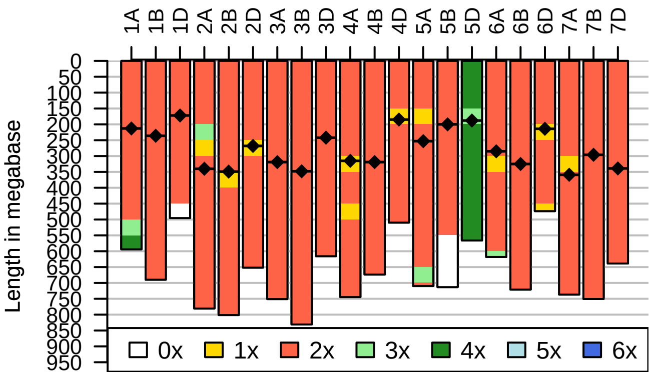

17SI−325−5 (5BL−9)

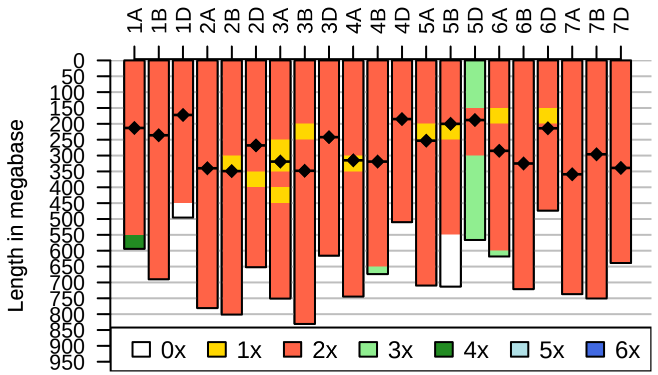

17SI−325−6 (5BL−9)

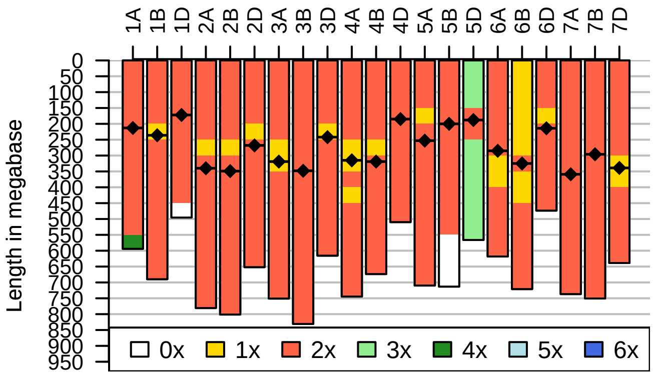

17SI−325−7 (5BL−9)

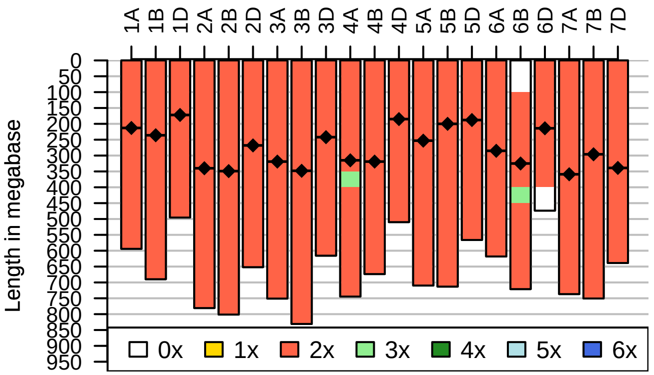

17SI−326 (6BS−3, 6DL−12)

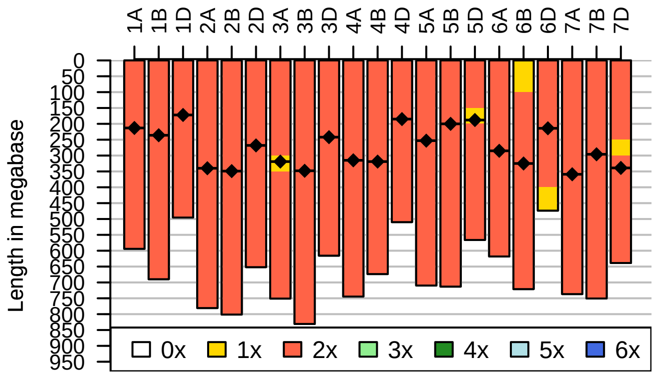

17SI−327 (6BS−3, 6DL−12)

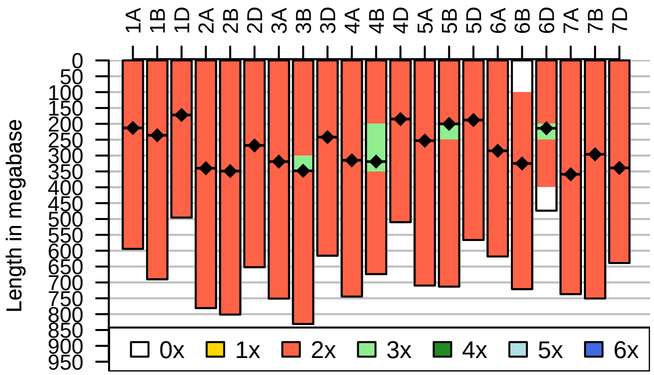

17SI−328 (6BS−3, 6DL−12)

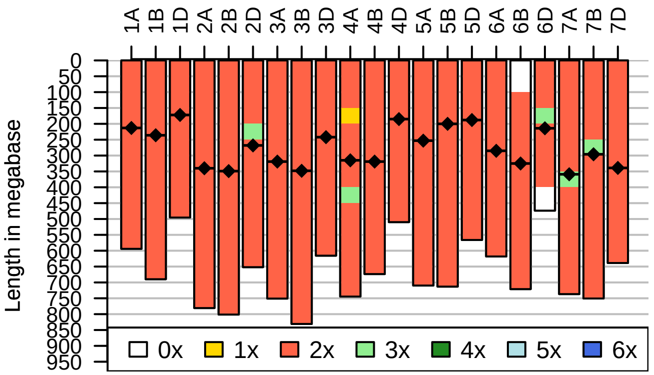

17SI−329−1 (6BS−3, 6DL−12)

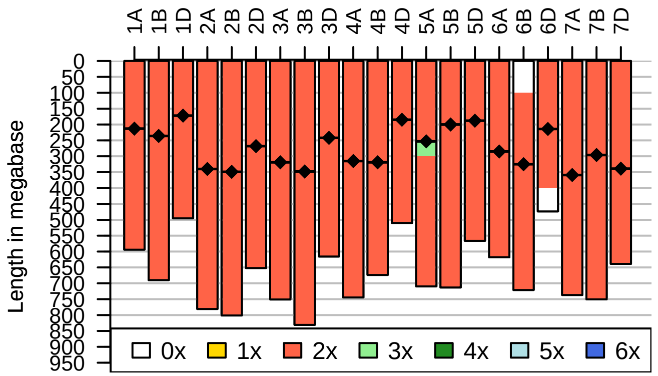

17SI−329−2 (6BS−3, 6DL−12)

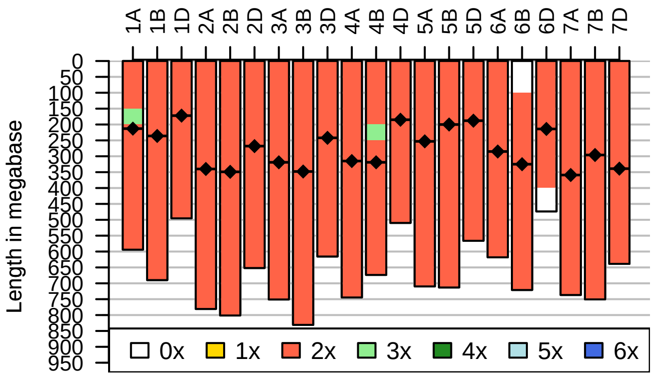

17SI−329−3 (6BS−3, 6DL−12)

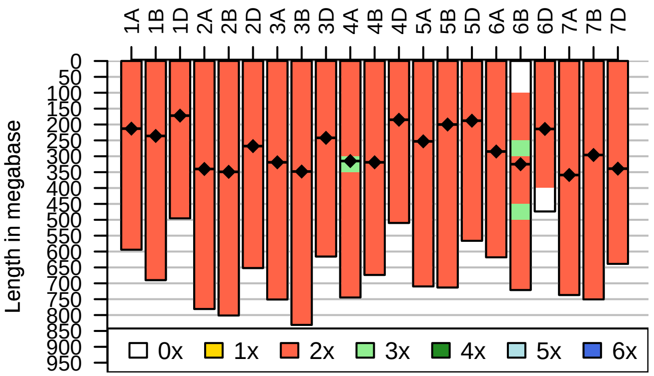

17SI−329−4 (6BS−3, 6DL−12)

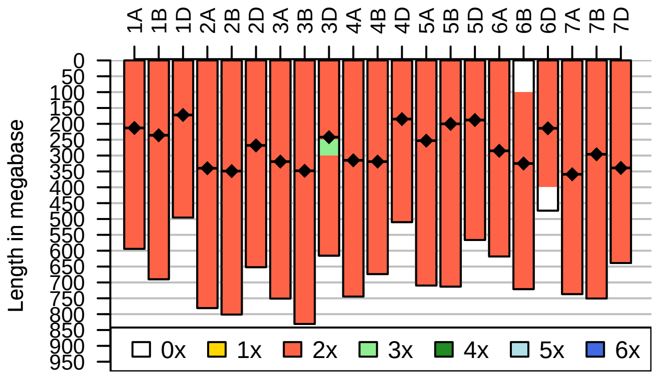

17SI−330 (6BS−3, 6DL−12)

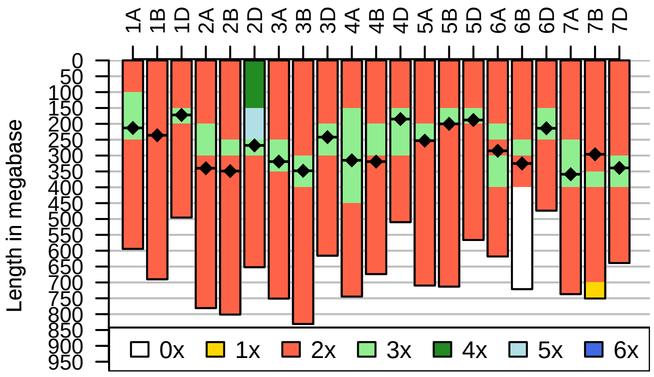

17SI−331−1 (6BL−6)

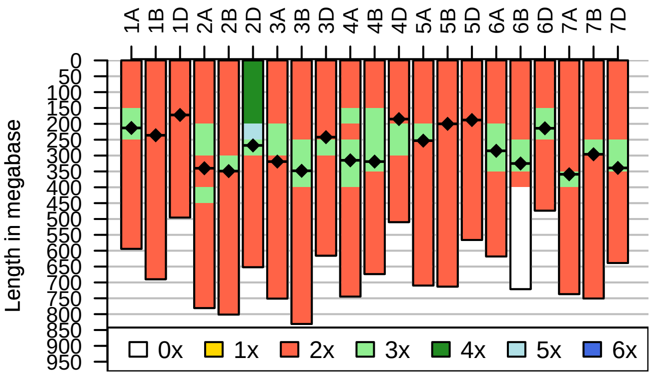

17SI−331−2 (6BL−6)

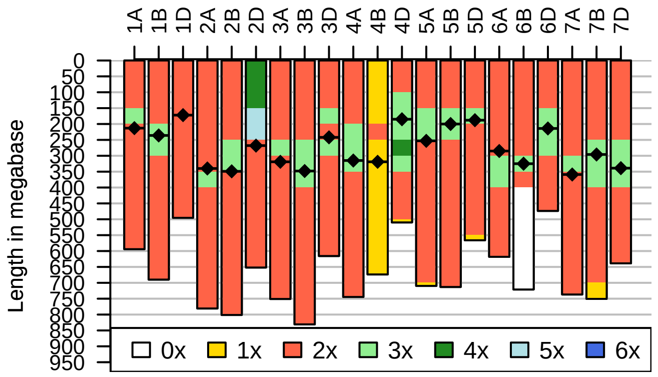

17SI−332−1 (6BL−6)

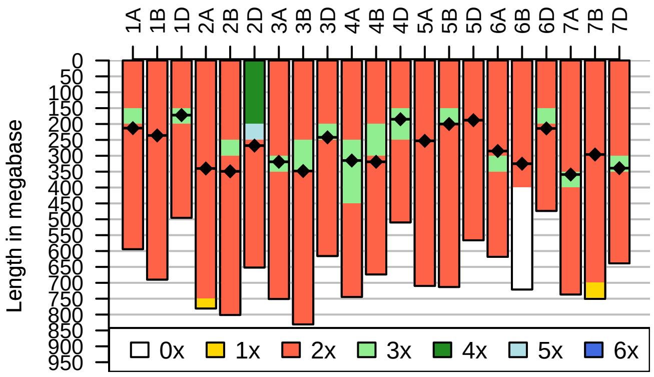

17SI−332−2 (6BL−6)

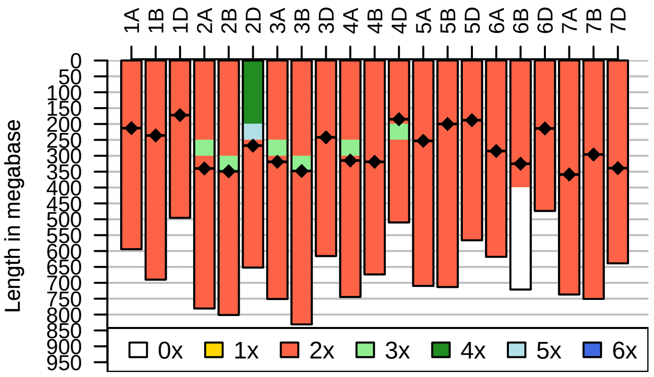

17SI−333−1 (6BL−6)

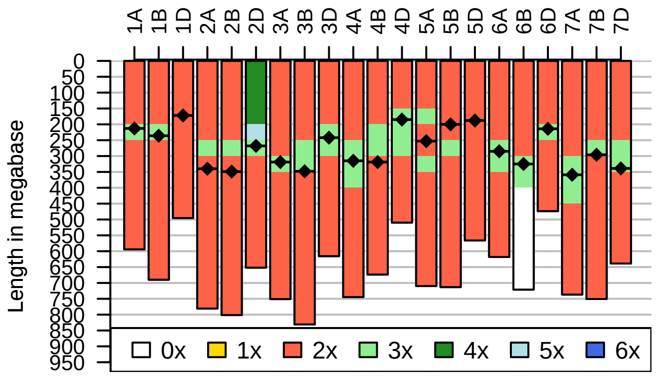

17SI−333−2 (6BL−6)

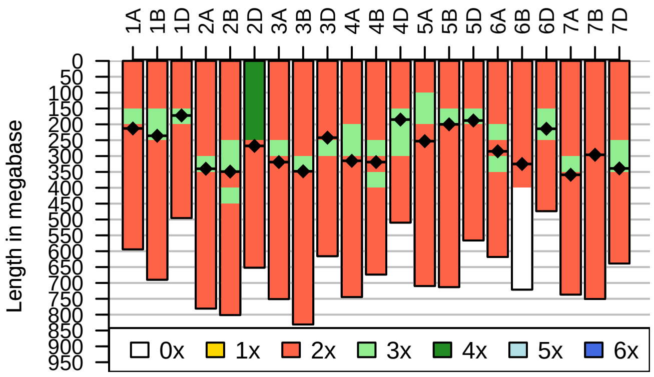

17SI−333−3 (6BL−6)

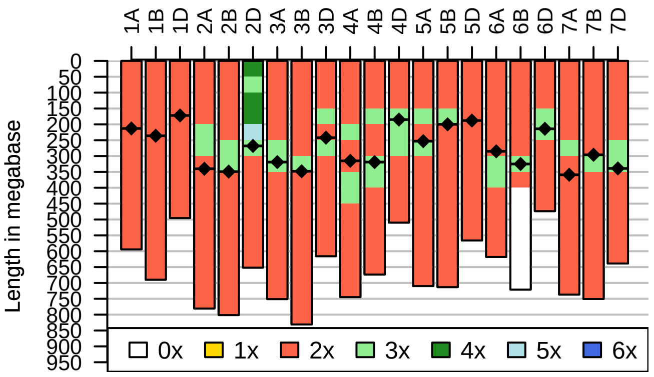

17SI−333−4 (6BL−6)

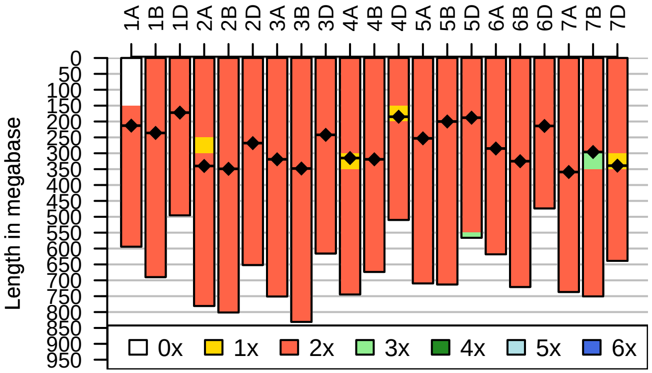

17SI−334−1 (1AS−2)

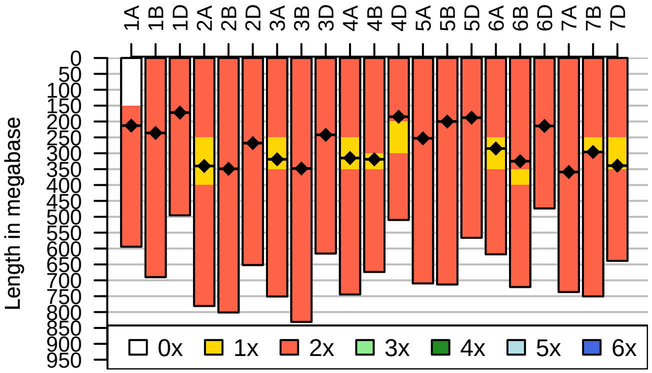

17SI−334−2 (1AS−2)

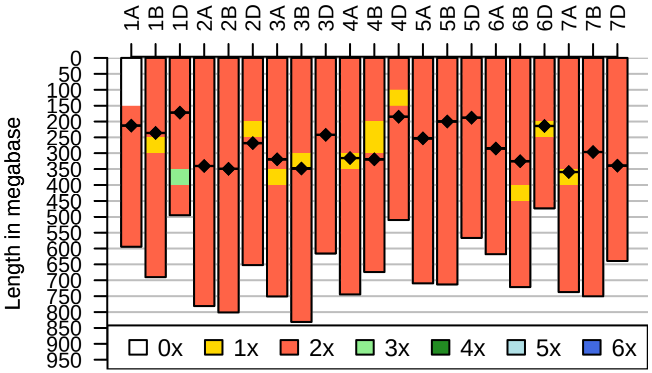

17SI−334−3 (1AS−2)

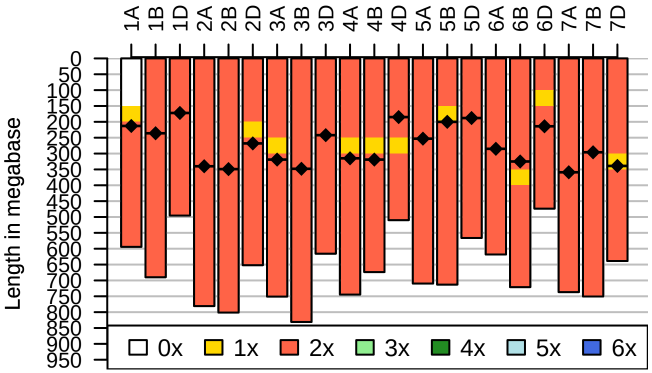

17SI−334−4 (1AS−2)

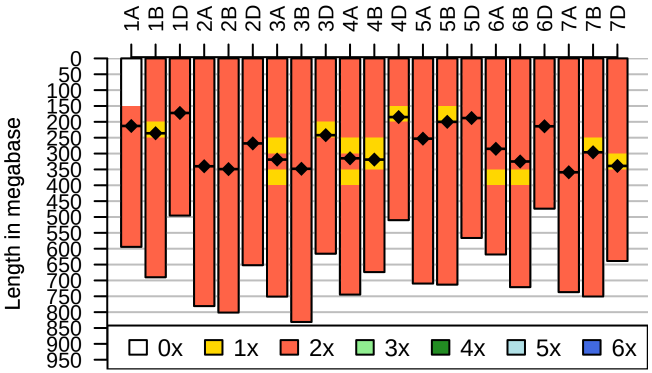

17SI−334−5 (1AS−2)

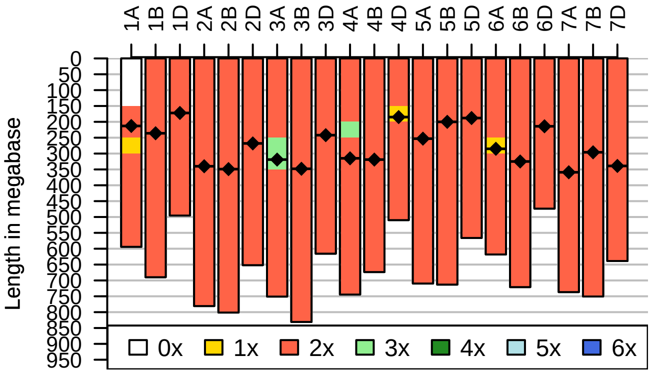

17SI−335−1 (1AS−2)

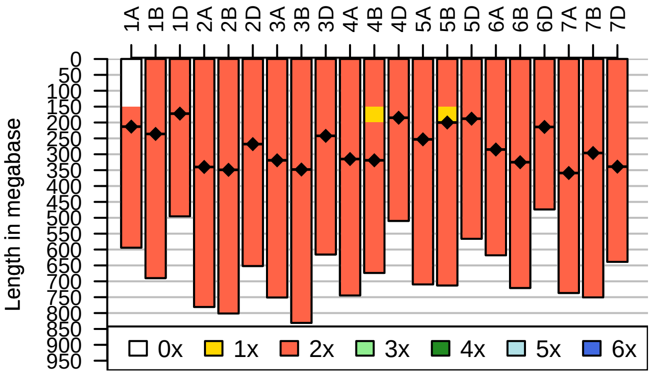

17SI−335−2 (1AS−2)

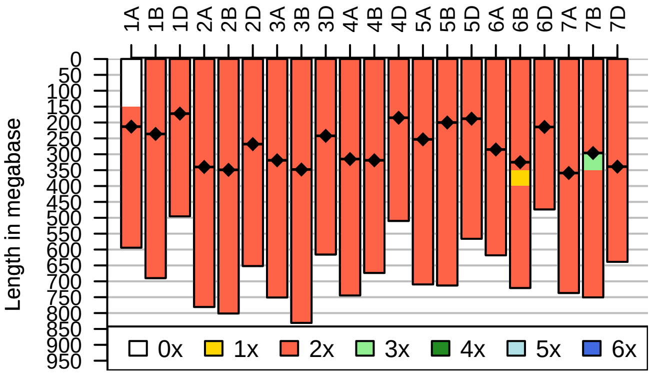

17SI−335−3 (1AS−2)

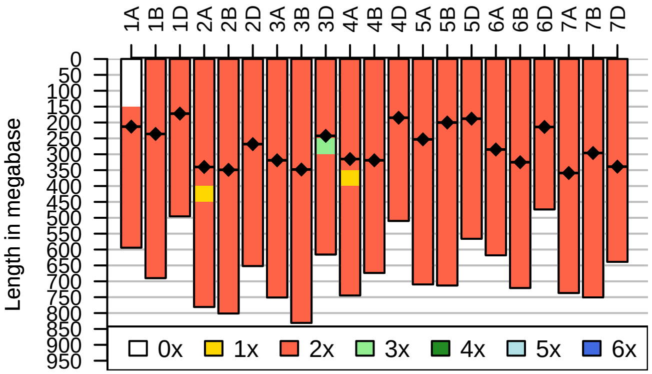

17SI−335−4 (1AS−2)

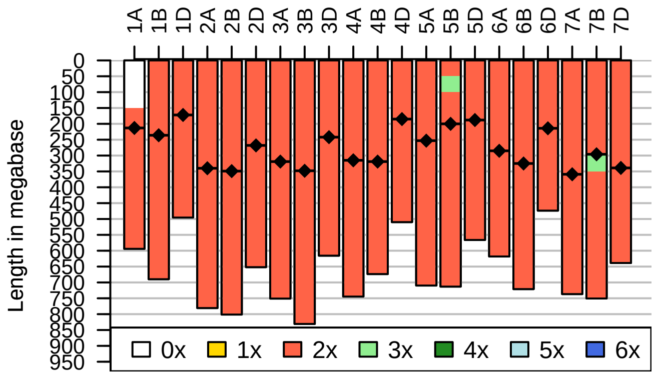

17SI−335−5 (1AS−2)

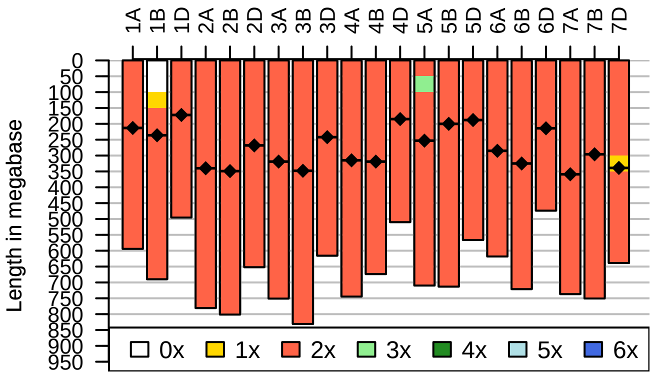

17SI−336−1 (1BS−7)

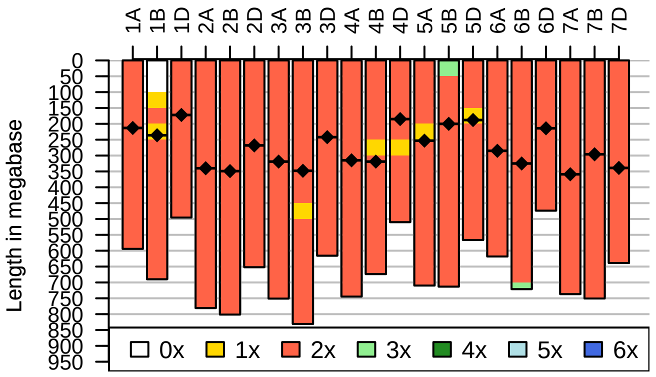

17SI−336−3 (1BS−7)

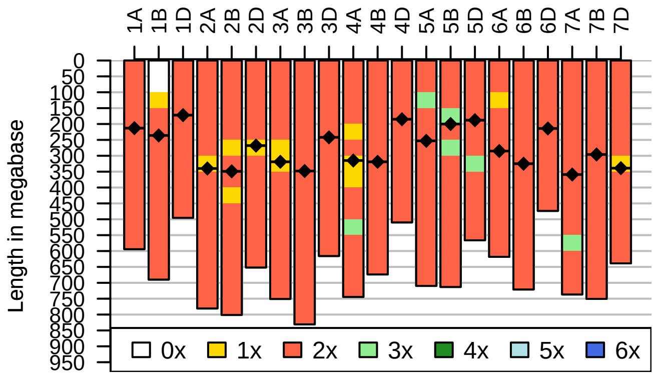

17SI−336−4 (1BS−7)

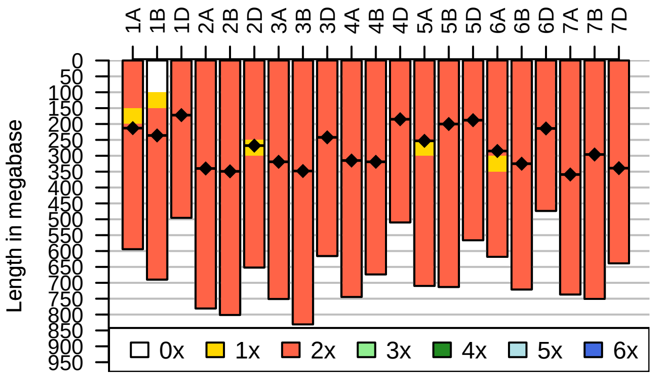

17SI−336−5 (1BS−7)

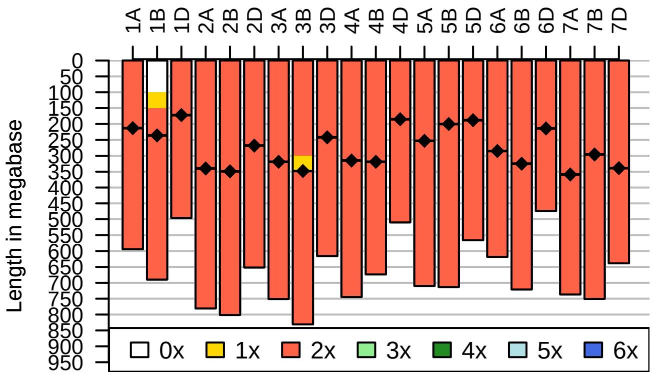

17SI−337−1 (1BS−7)

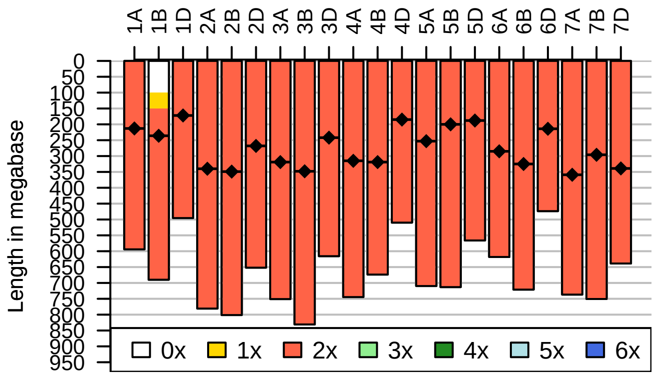

17SI−337−2 (1BS−7)

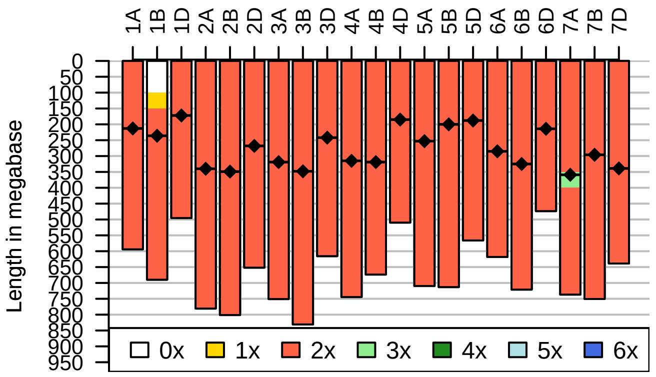

17SI−337−3 (1BS−7)

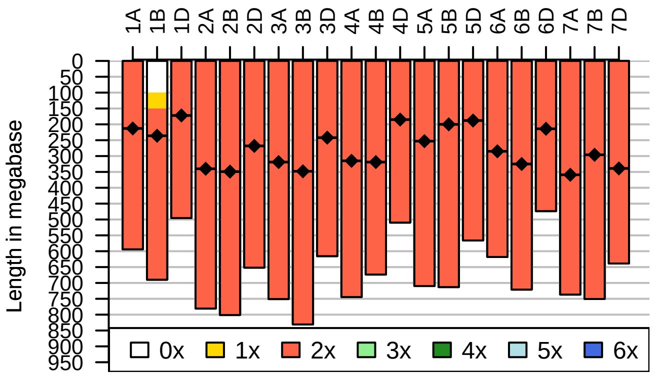

17SI−337−4 (1BS−7)

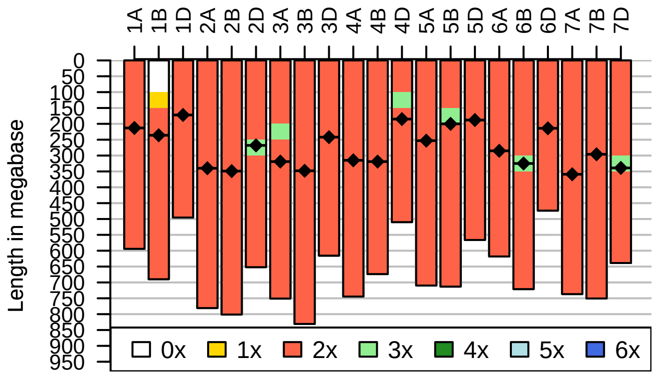

17SI−337−5 (1BS−7)

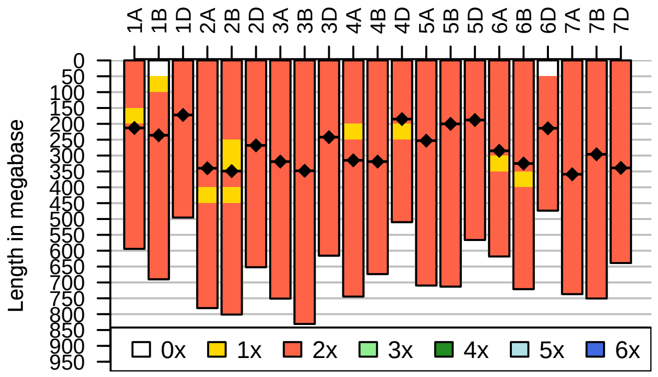

17SI−338−1 (1BS−19)

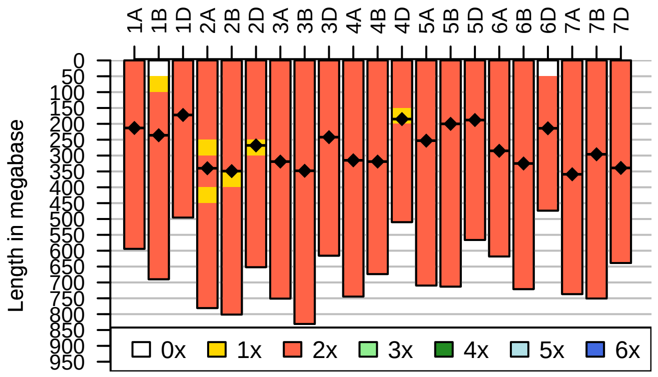

17SI−338−2 (1BS−19)

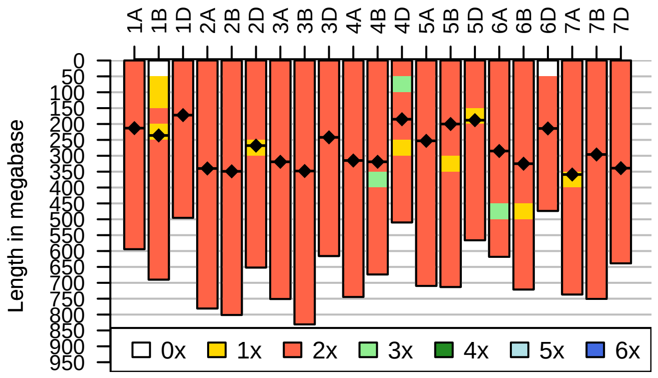

17SI−338−3 (1BS−19)

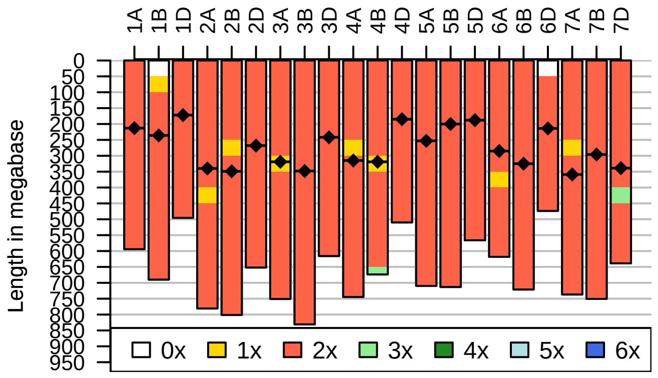

17SI−339−1 (1BS−19)

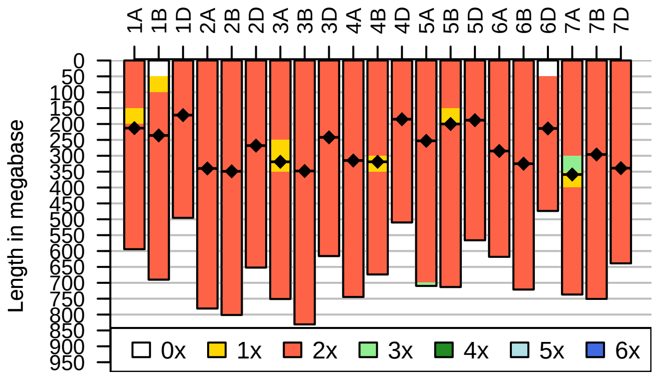

17SI−339−2 (1BS−19)

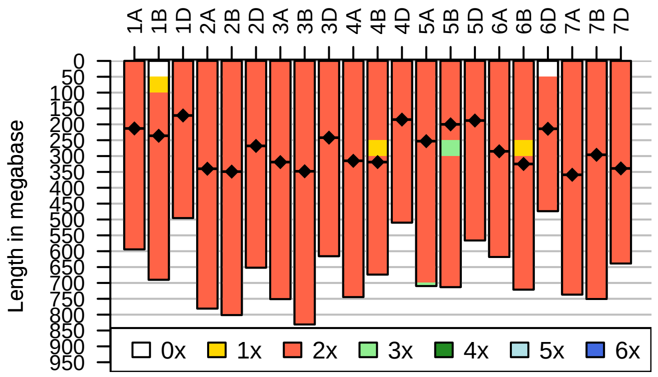

17SI−339−3 (1BS−19)

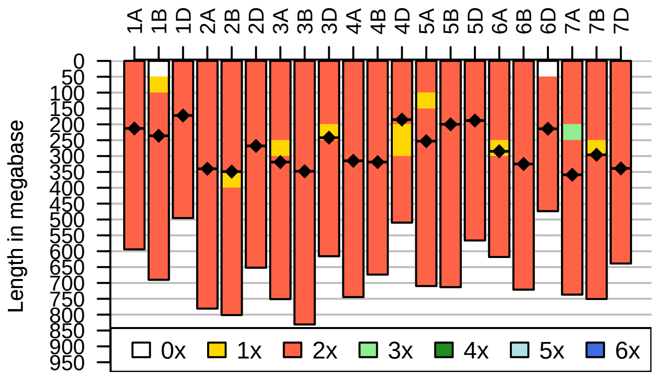

17SI−339−4 (1BS−19)

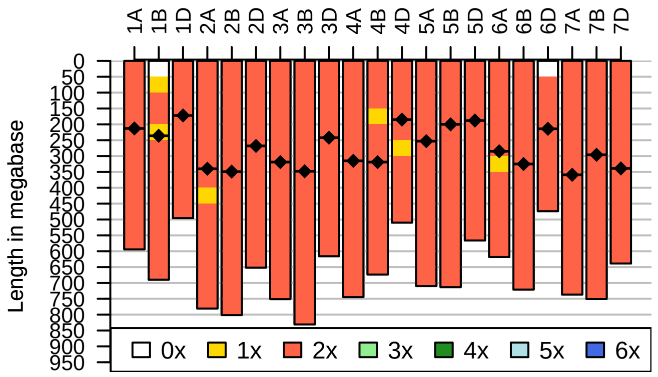

17SI−339−5 (1BS−19)

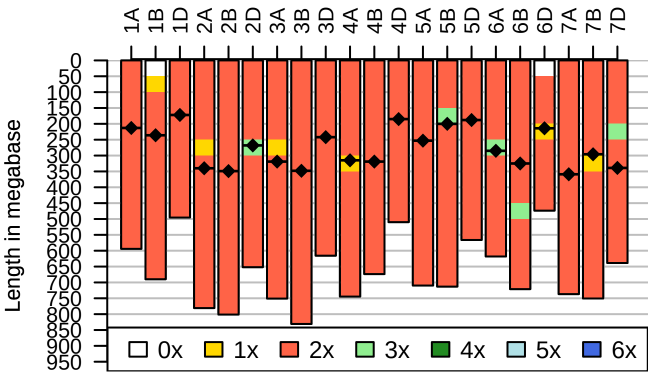

17SI−339−6 (1BS−19)

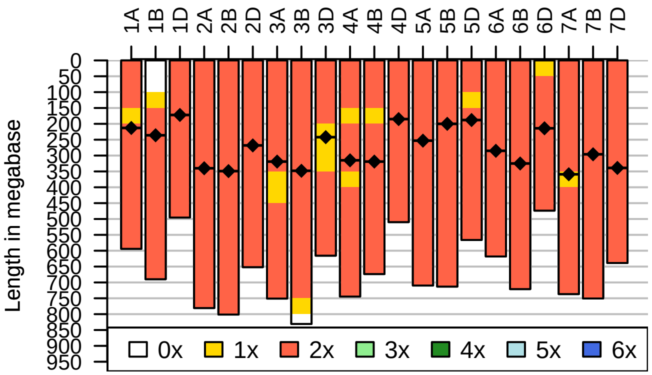

17SI−340−1 (1BS−20)

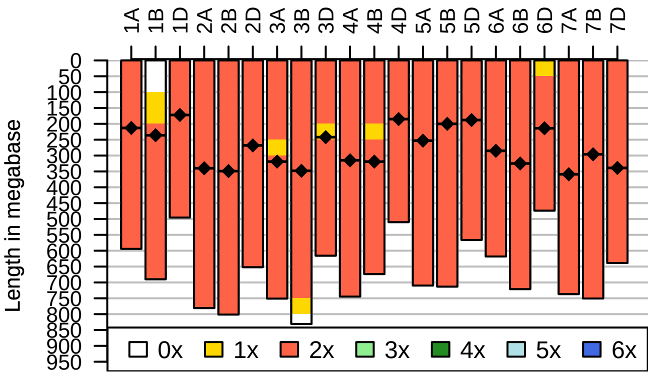

17SI−340−2 (1BS−20)

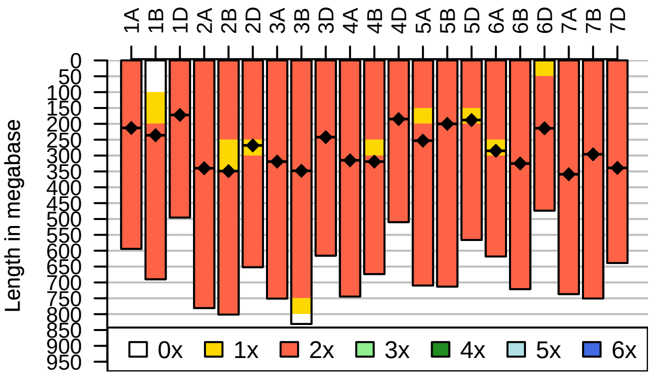

17SI−340−3 (1BS−20)

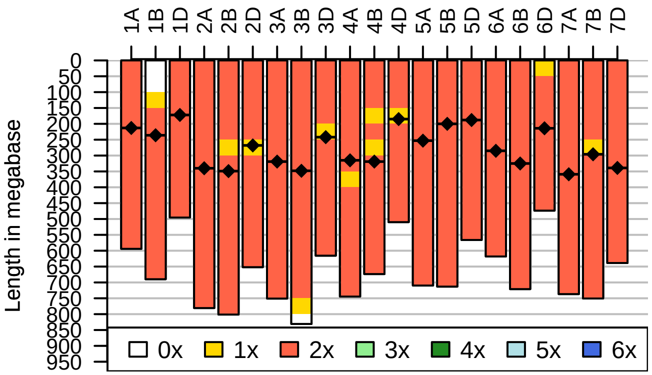

17SI−340−4 (1BS−20)

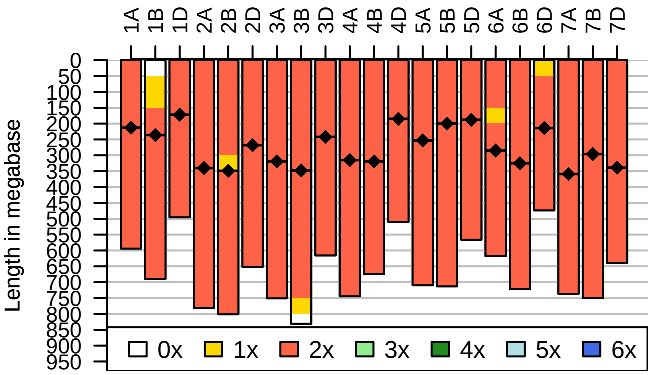

17SI−340−5 (1BS−20)

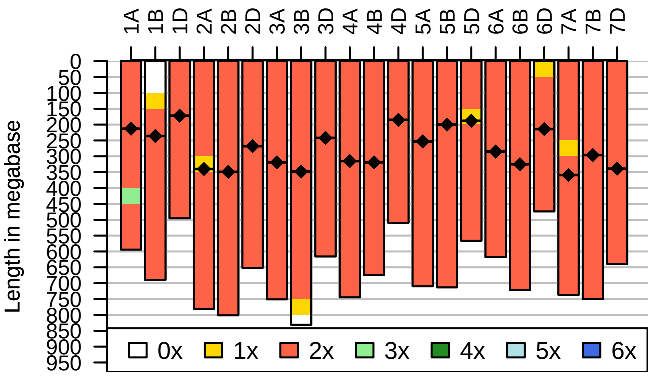

17SI−340−6 (1BS−20)

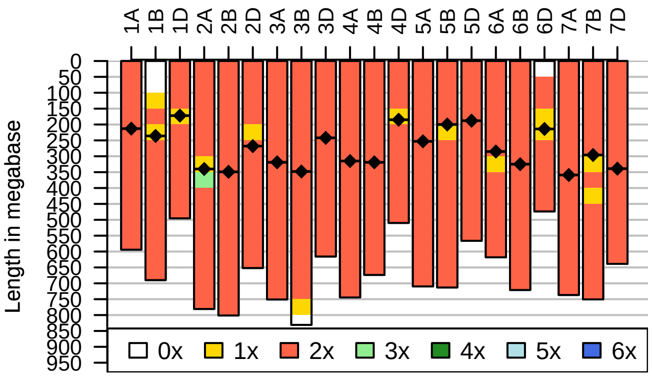

17SI−341−1 (1BS−20)

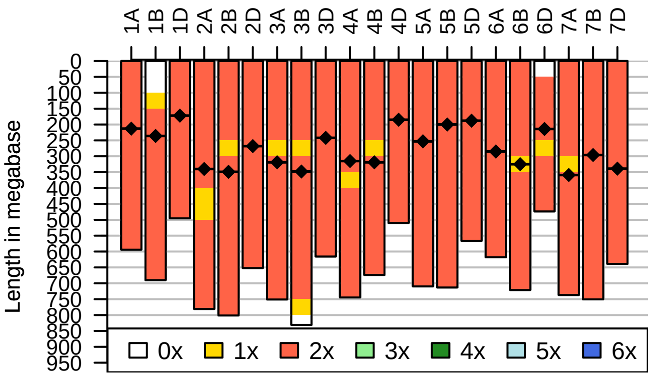

17SI−341−2 (1BS−20)

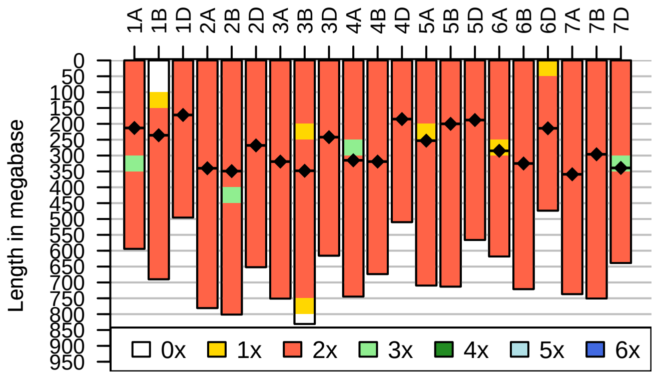

17SI−341−4 (1BS−20)

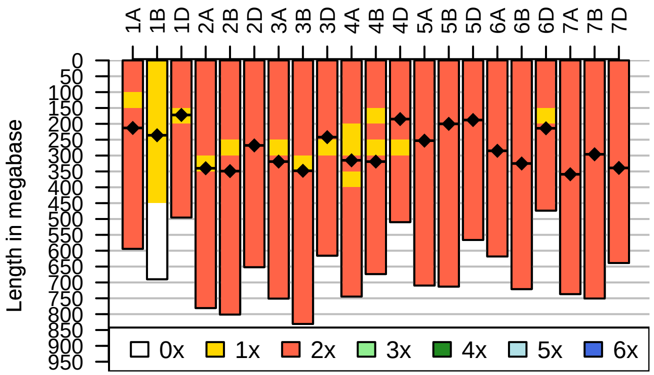

17SI−342−1 (1BL−7)

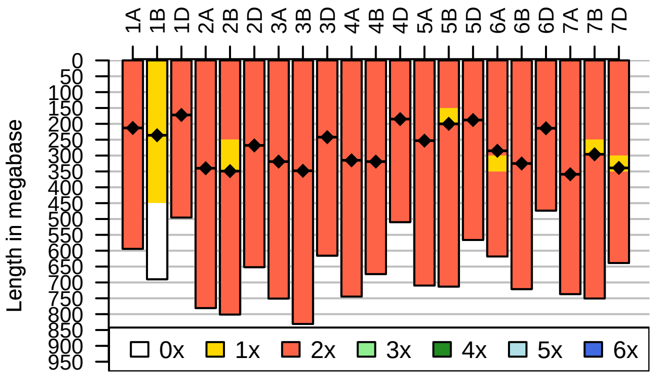

17SI−342−2 (1BL−7)

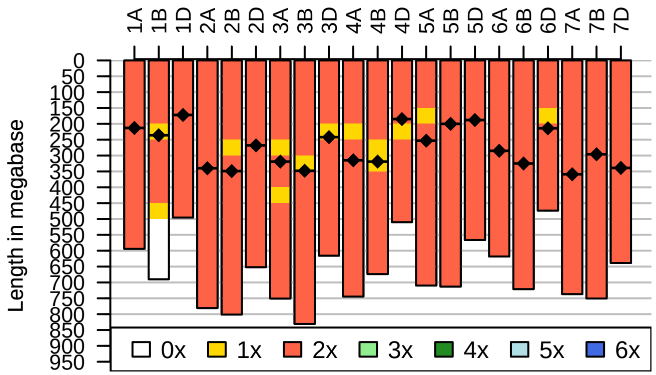

17SI−342−3 (1BL−7)

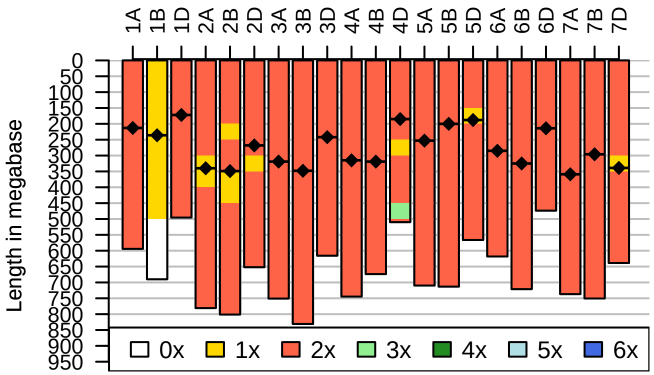

17SI−342−4 (1BL−7)

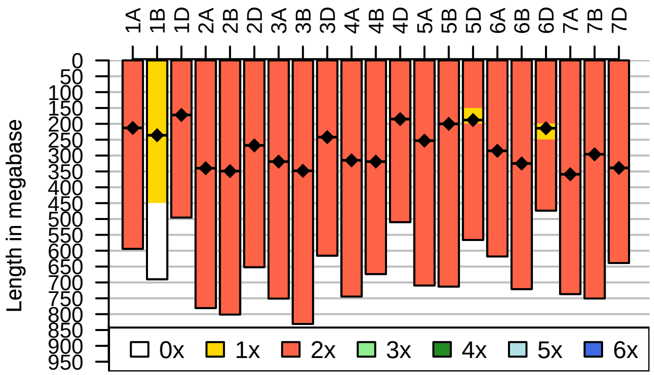

17SI−342−5 (1BL−7)

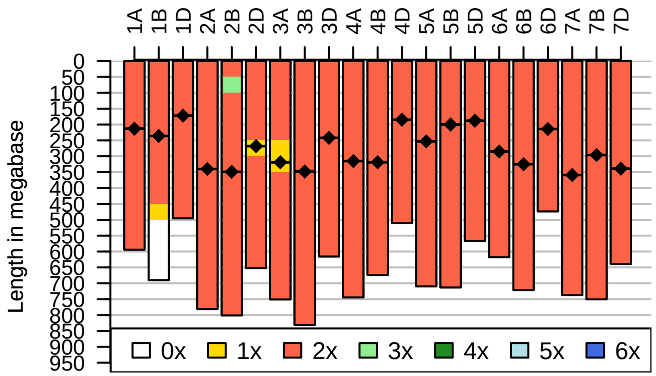

17SI−343−1 (1BL−7)

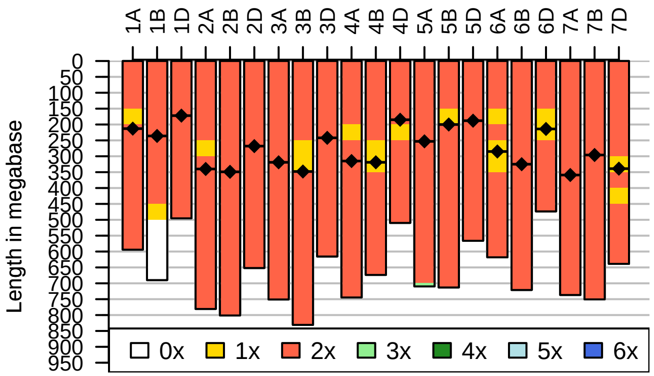

17SI−343−2 (1BL−7)

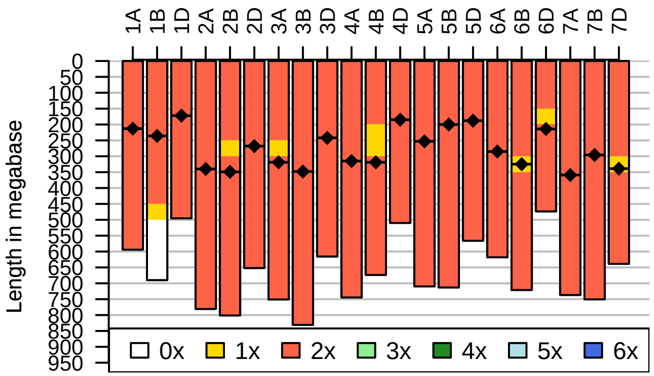

17SI−343−3 (1BL−7)

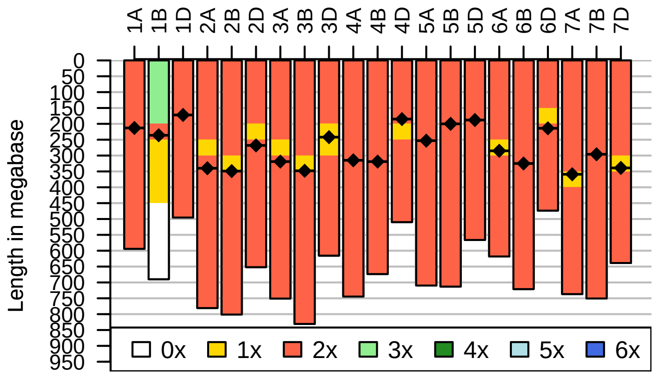

17SI−343−4 (1BL−7)

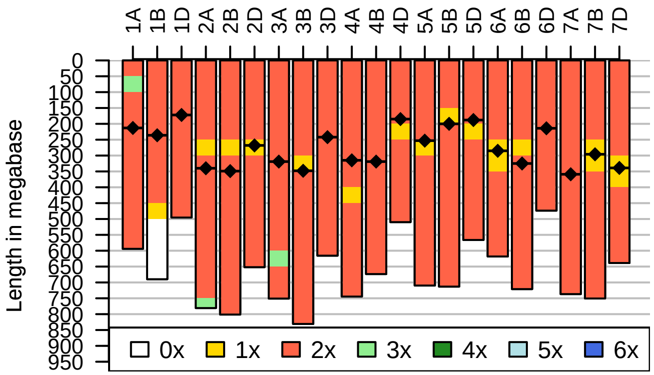

17SI−343−5 (1BL−7)

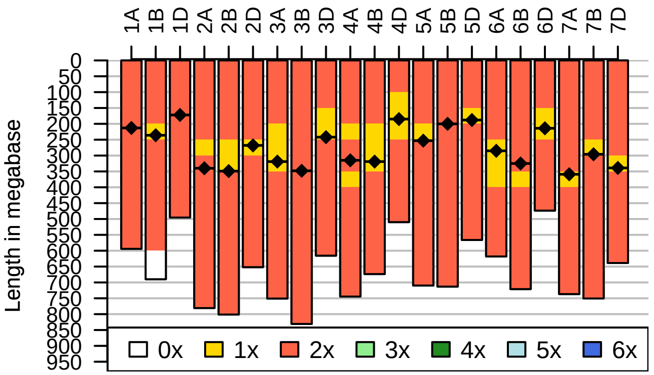

17SI−344−1 (1BL−8)

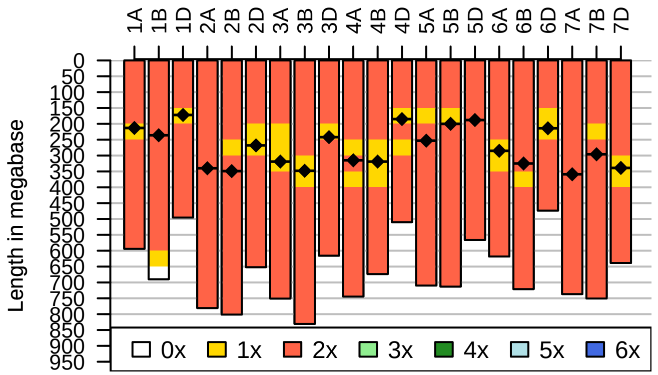

17SI−344−2 (1BL−8)

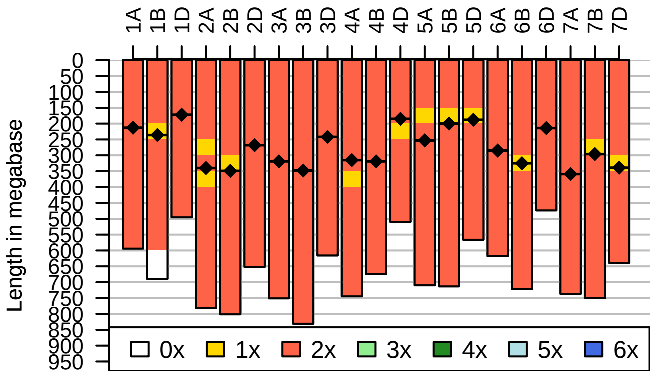

17SI−345−1 (1BL−8)

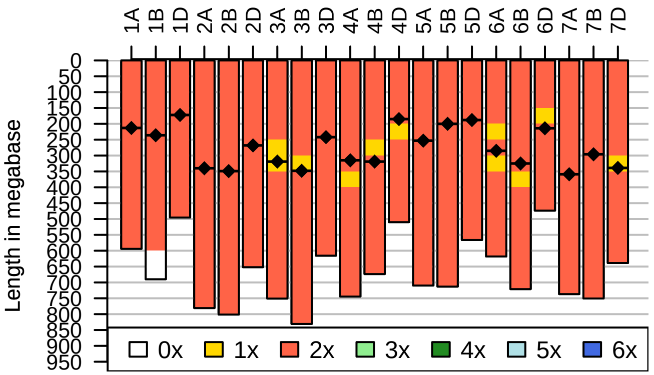

17SI−345−2 (1BL−8)

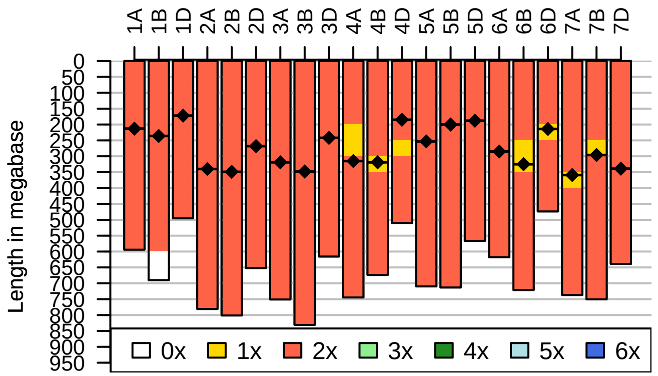

17SI−345−3 (1BL−8)

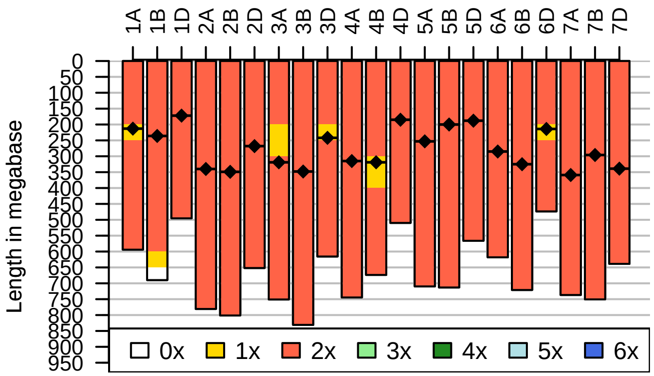

17SI−345−4 (1BL−8)

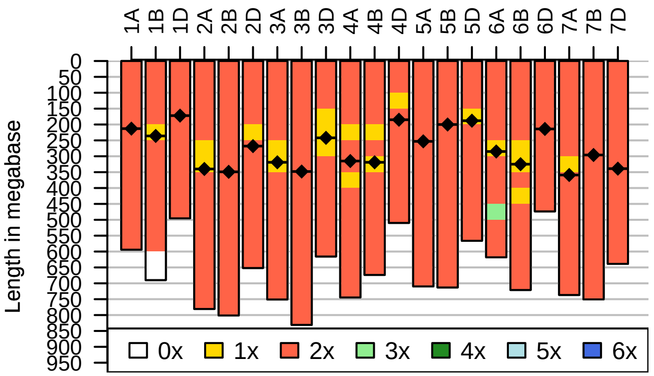

17SI−345−5 (1BL−8)

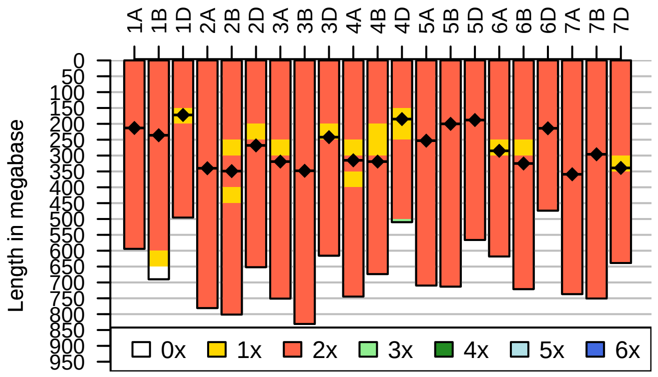

17SI−346−1 (1BL−8)

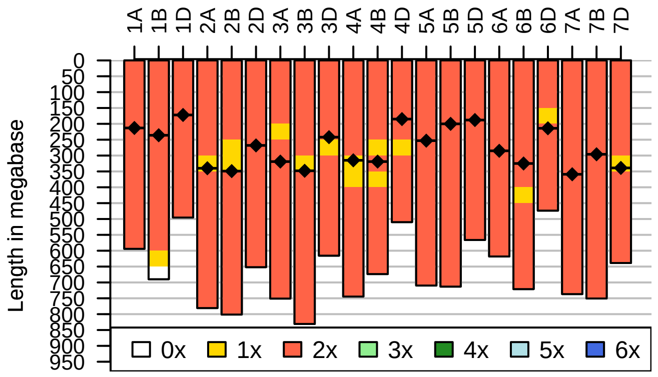

17SI−346−2 (1BL−8)

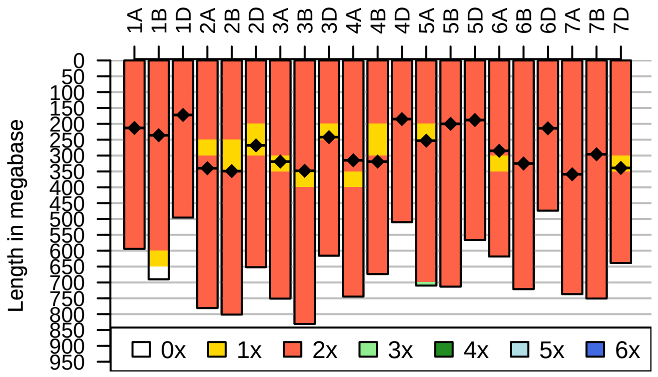

17SI−346−3 (1BL−8)

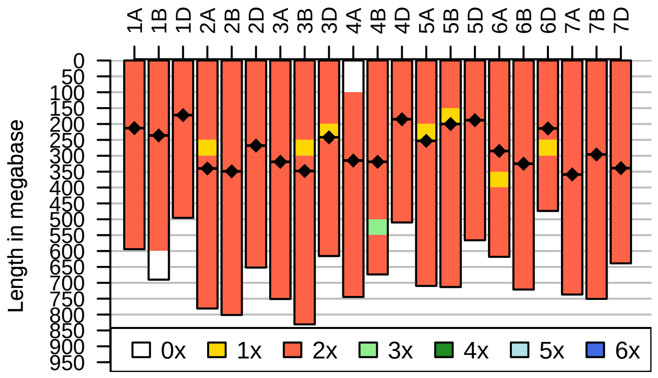

17SI−347−1 (1BL−9)

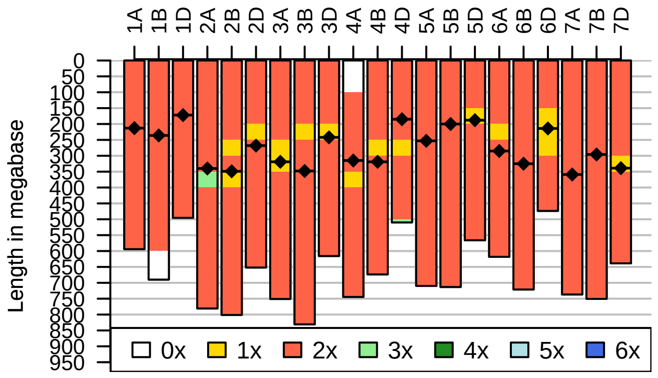

17SI−347−2 (1BL−9)

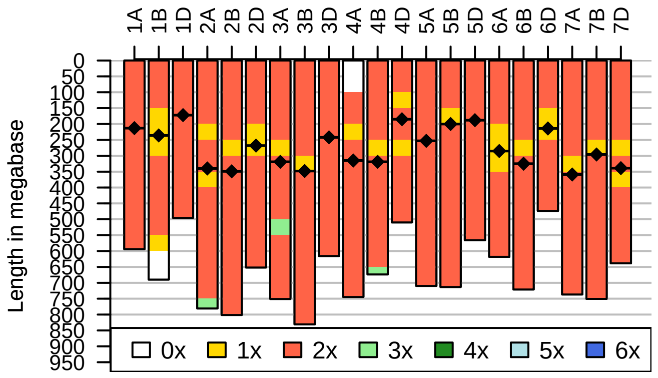

17SI−347−3 (1BL−9)

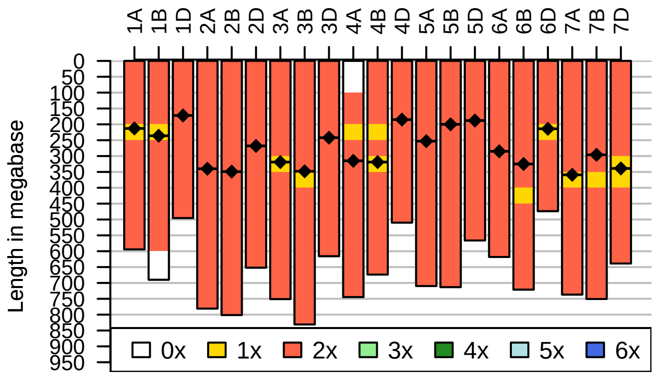

17SI−347−4 (1BL−9)

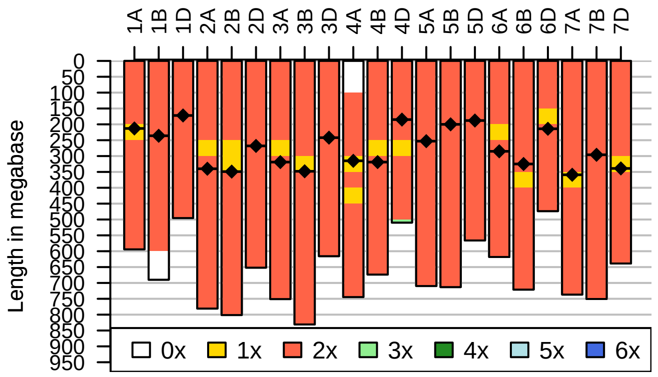

17SI−347−5 (1BL−9)

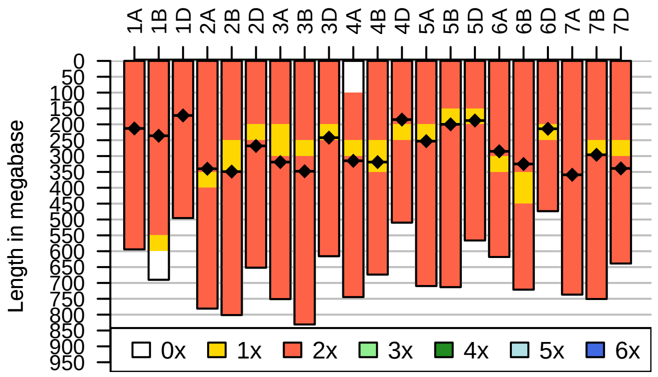

17SI−347−6 (1BL−9)

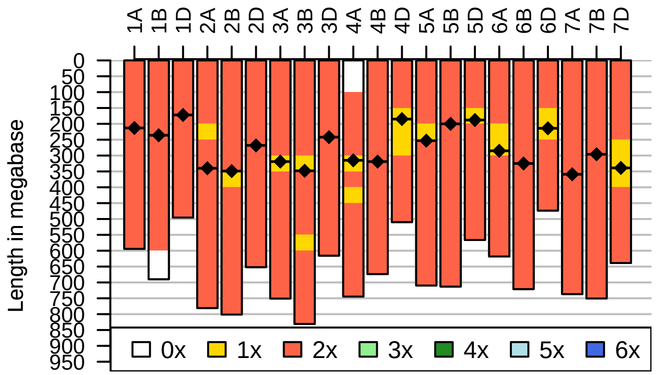

17SI−348−1 (1BL−9)

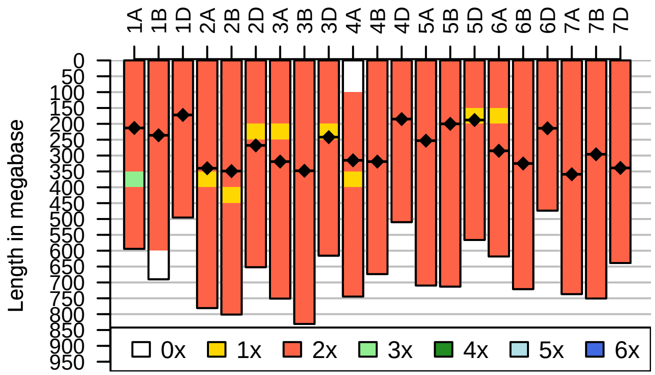

17SI−348−2 (1BL−9)

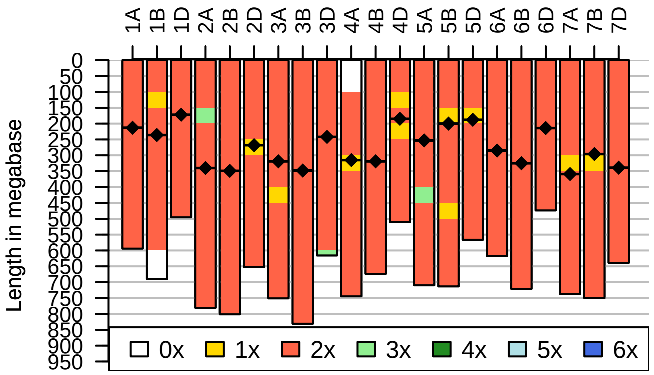

17SI−348−3 (1BL−9)

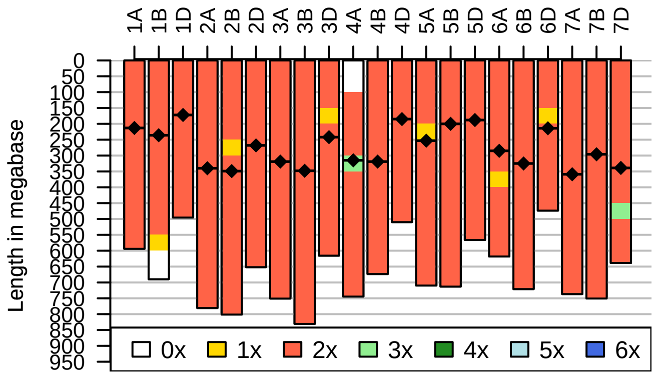

17SI−348−4 (1BL−9)

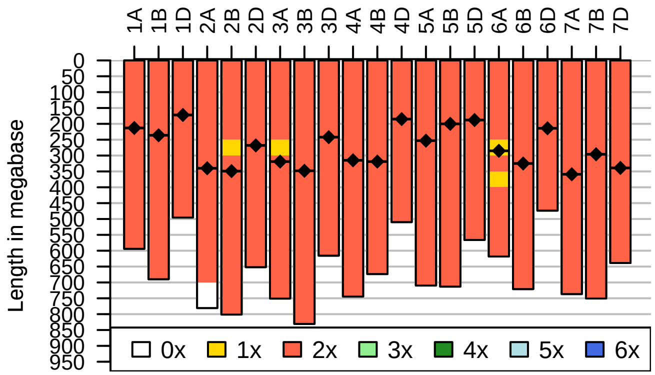

17SI−349−1 (2AL−3)

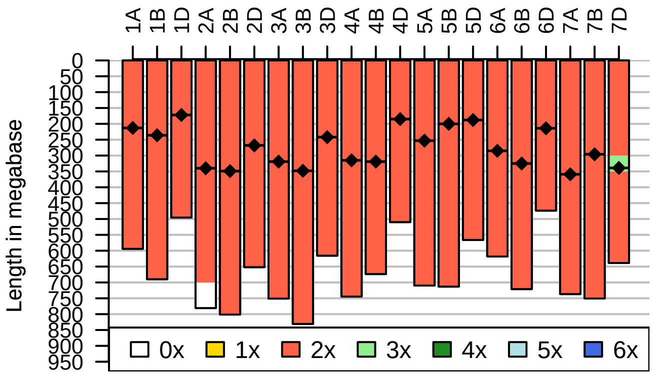

17SI−349−2 (2AL−3)

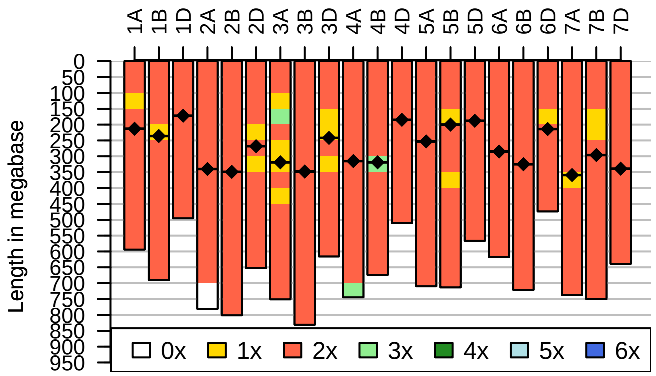

17SI−349−3 (2AL−3)

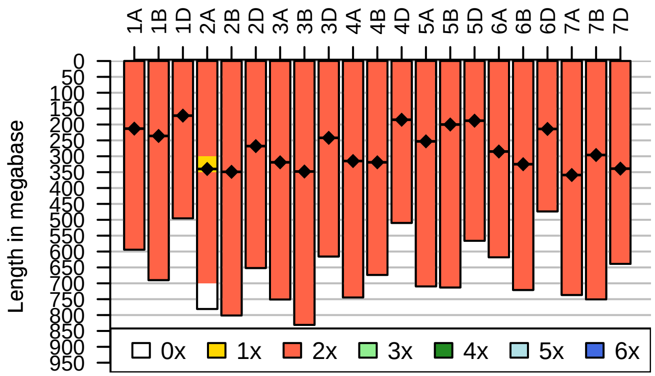

17SI−349−4 (2AL−3)

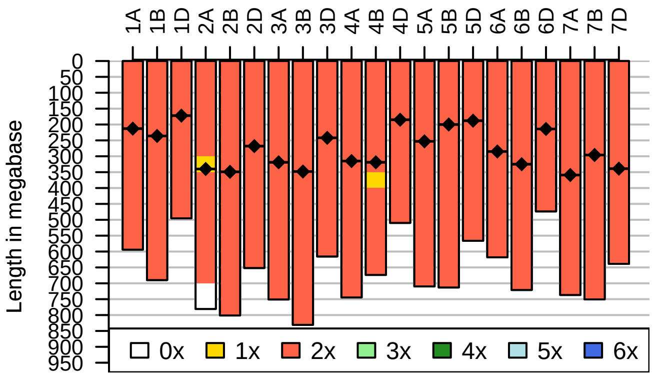

17SI−350−1 (2AL−3)

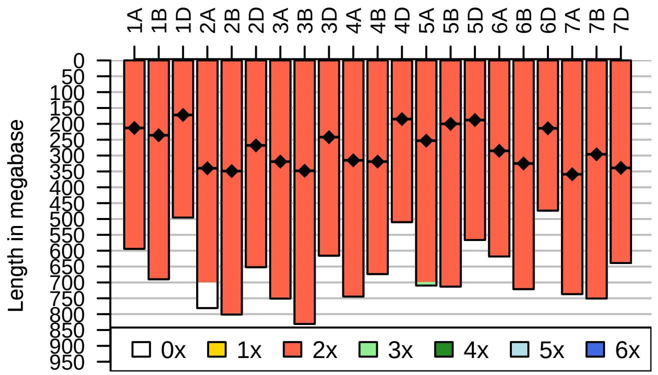

17SI−350−2 (2AL−3)

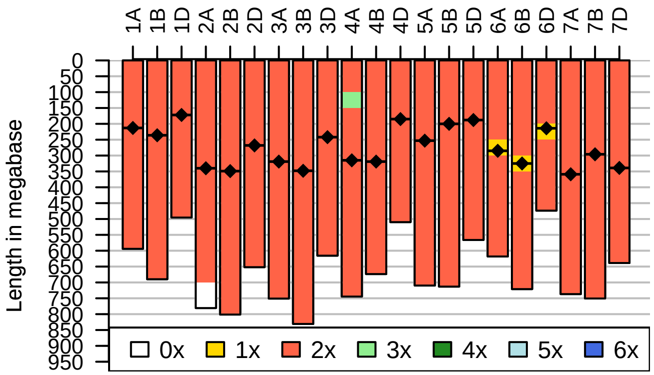

17SI−350−3 (2AL−3)

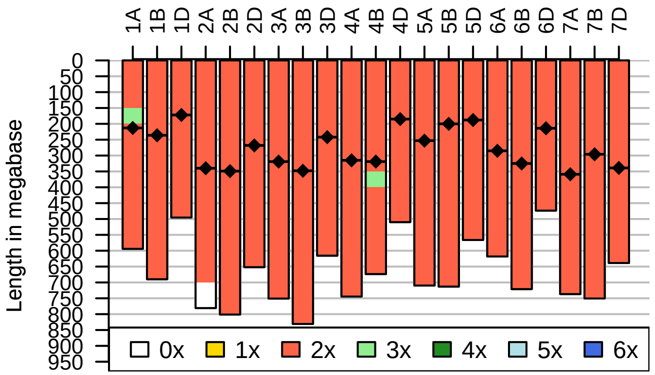

17SI−350−4 (2AL−3)

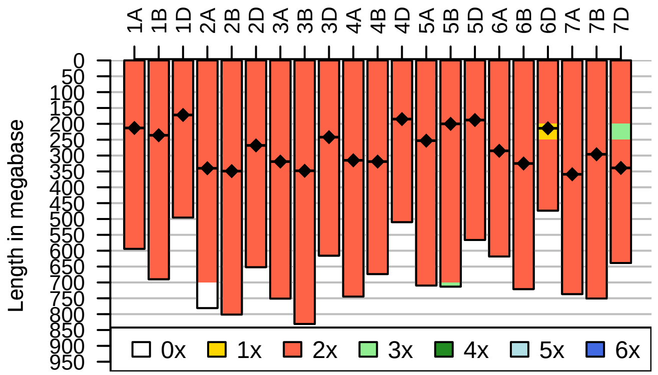

17SI−350−5 (2AL−3)

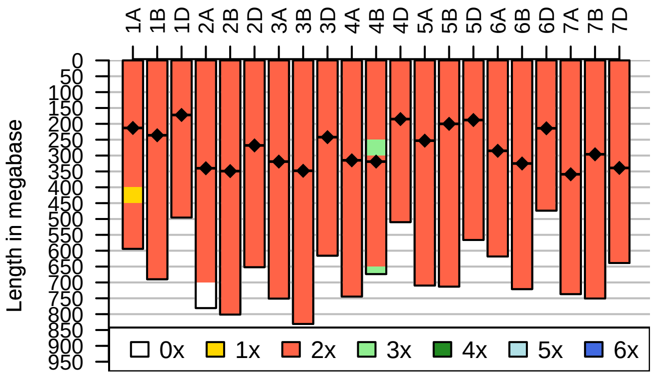

17SI−350−6 (2AL−3)

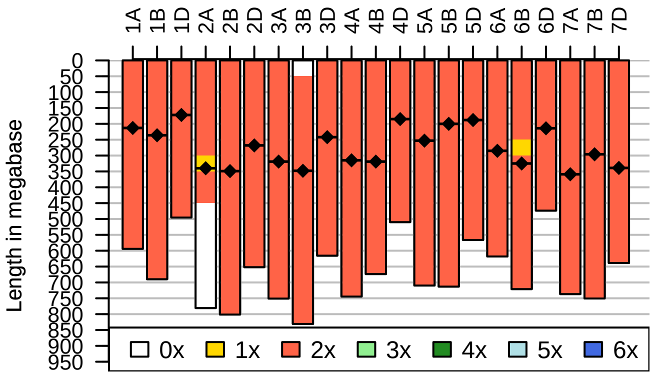

17SI−351−1 (2AL−4)

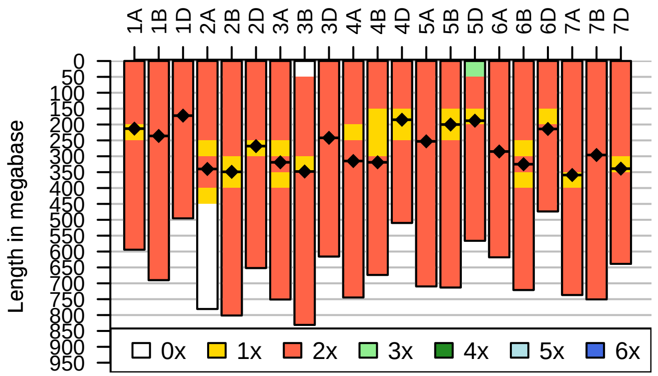

17SI−351−2 (2AL−4)

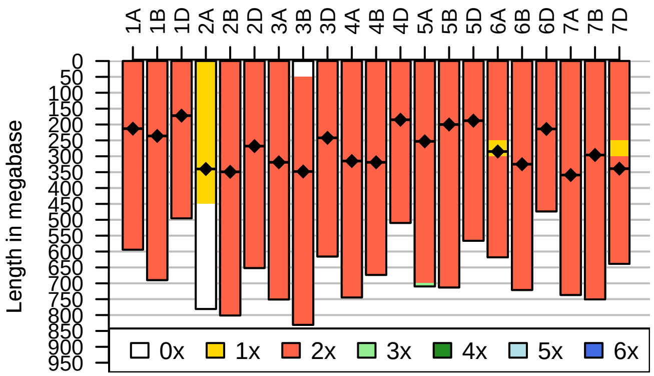

17SI−351−3 (2AL−4)

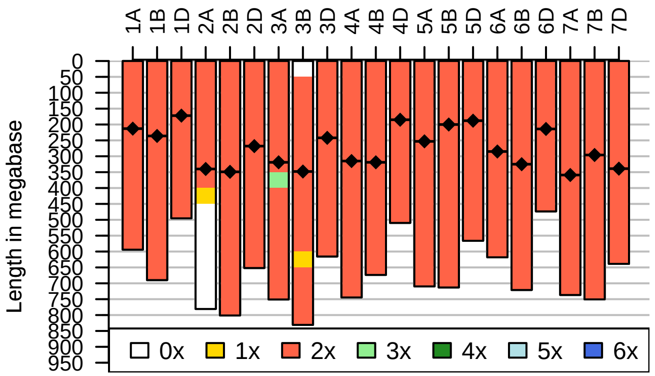

17SI−351−4 (2AL−4)

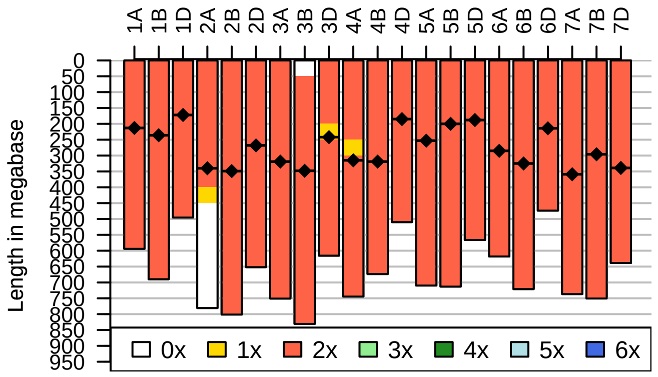

17SI−351−5 (2AL−4)

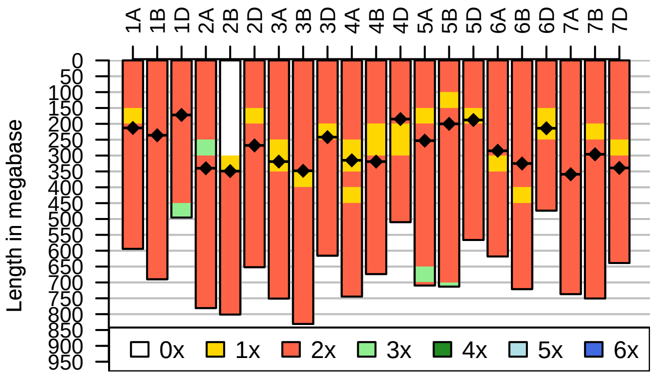

17SI−352−1 (2BS−2)

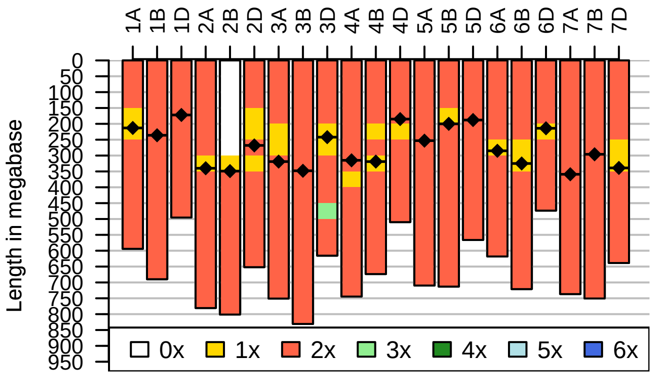

17SI−352−2 (2BS−2)

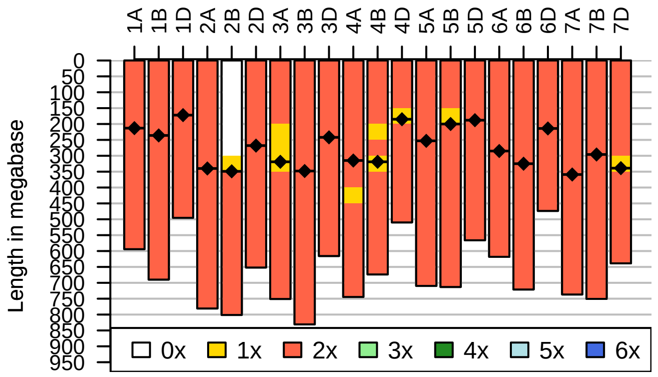

17SI−352−3 (2BS−2)

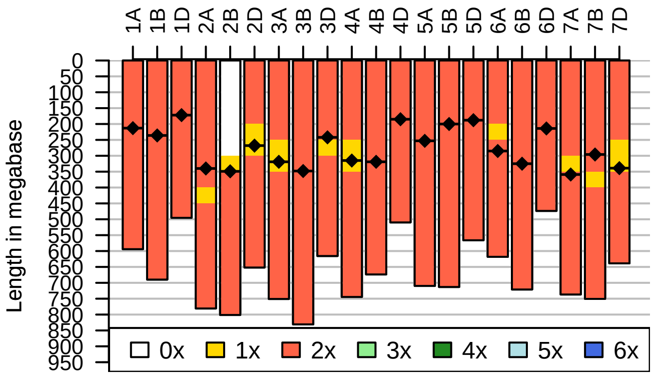

17SI−352−4 (2BS−2)

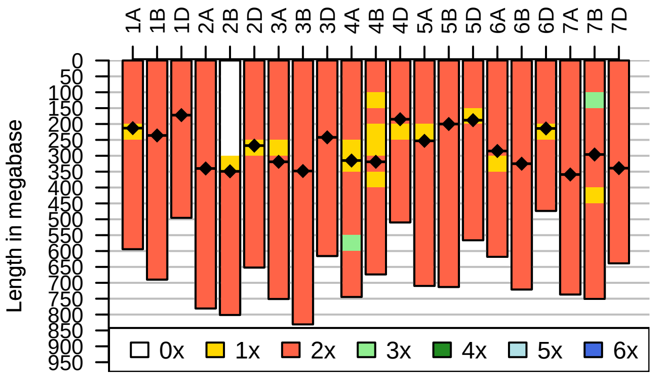

17SI−352−5 (2BS−2)

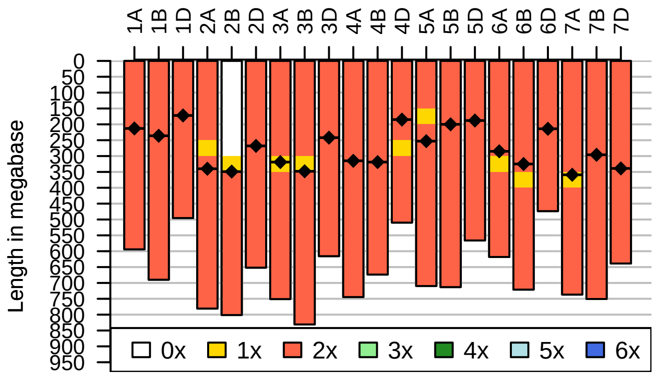

17SI−352−6 (2BS−2)

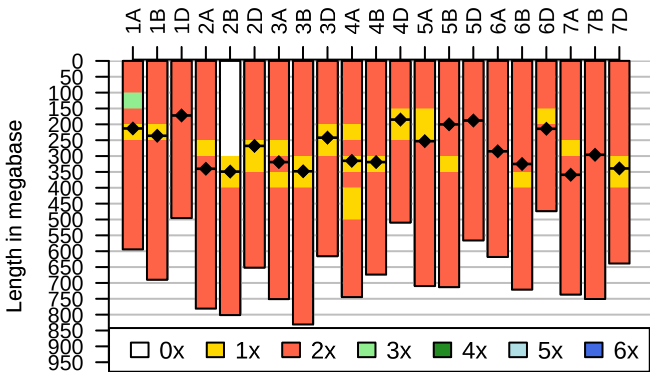

17SI−353−1 (2BS−2)

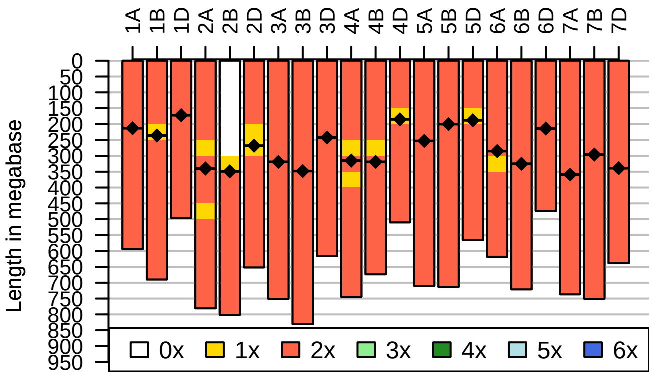

17SI−353−2 (2BS−2)

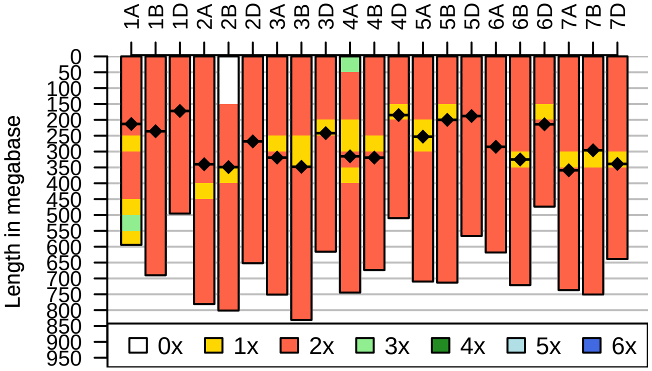

17SI−354−1 (2BS−14, 1AL−6)

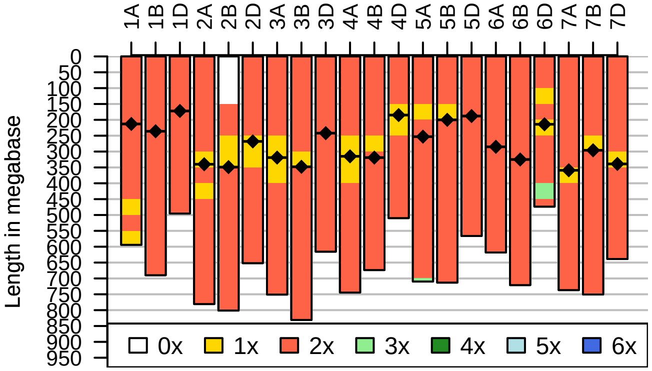

17SI−354−2 (2BS−14, 1AL−6)

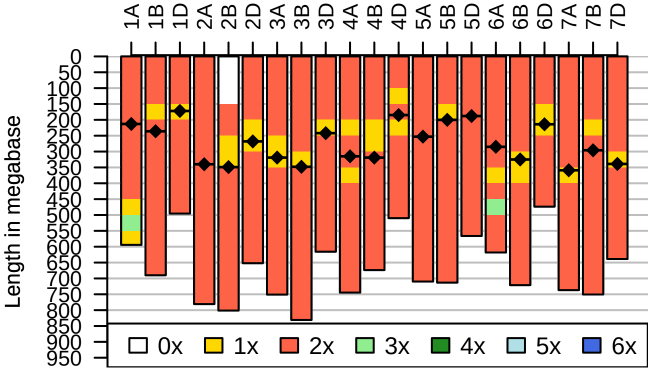

17SI−354−3 (2BS−14, 1AL−6)

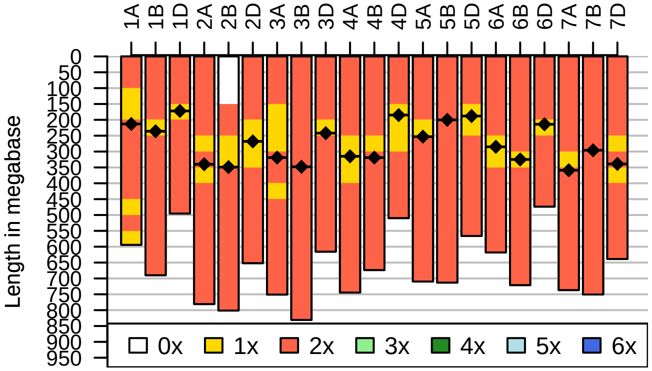

17SI−354−4 (2BS−14, 1AL−6)

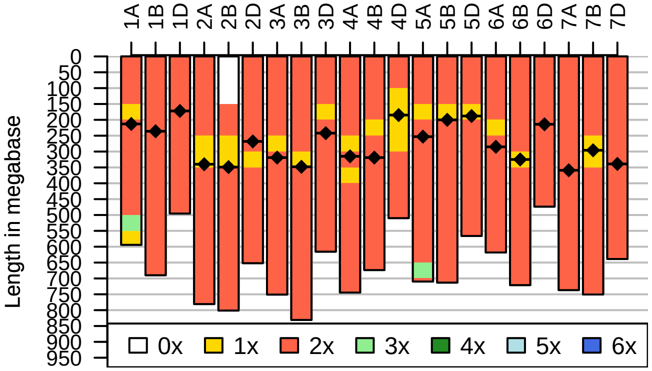

17SI−354−5 (2BS−14, 1AL−6)

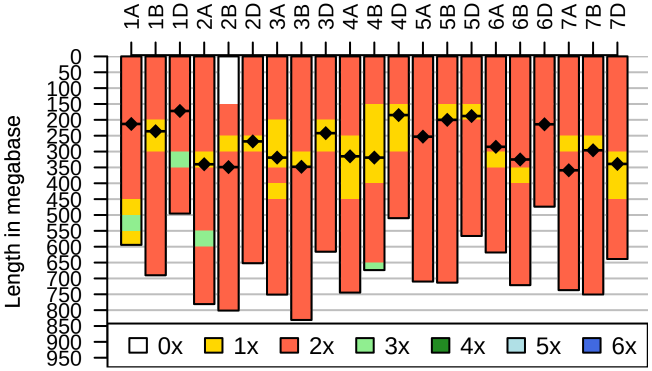

17SI−355−1 (2BS−14, 1AL−6)

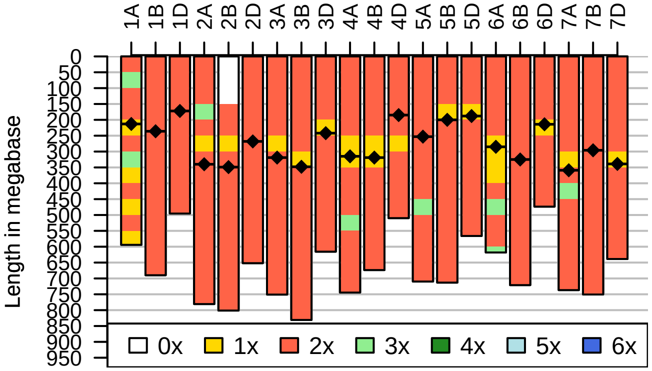

17SI−355−2 (2BS−14, 1AL−6)

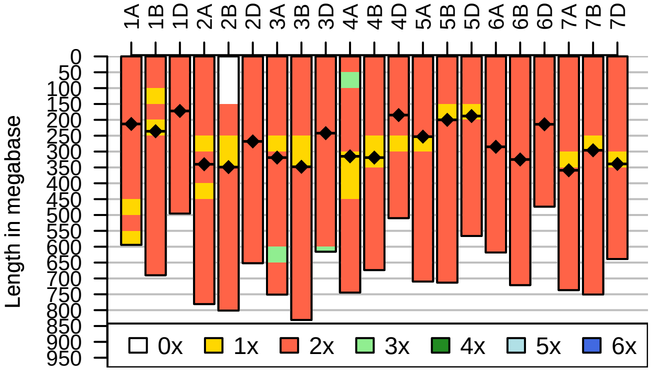

17SI−355−3 (2BS−14, 1AL−6)

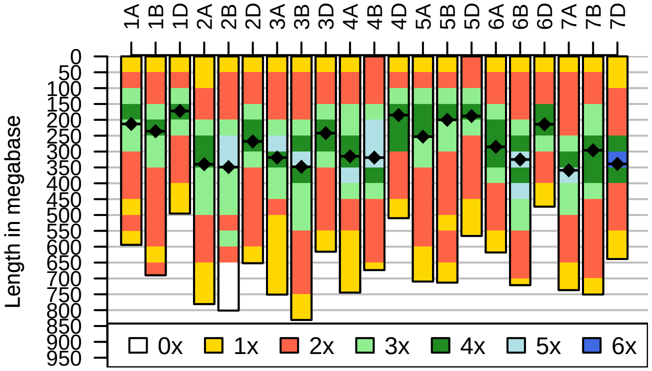

17SI−357−4 (2BL−1)

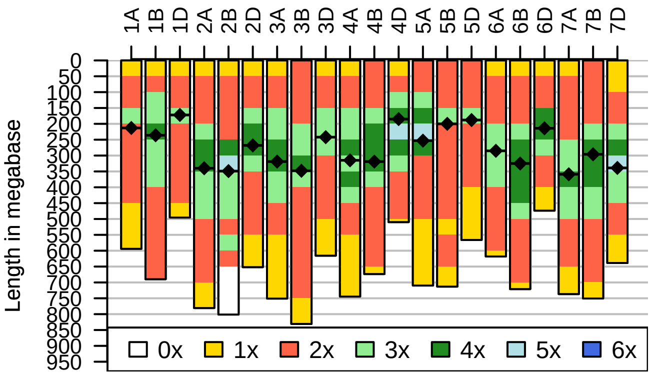

17SI−358−1 (2BL−1)

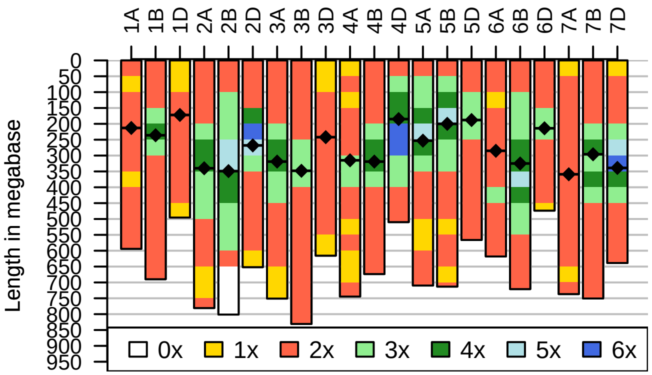

17SI−358−2 (2BL−1)

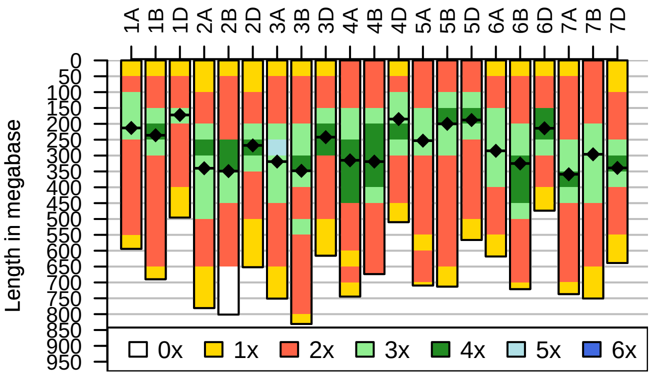

17SI−358−3 (2BL−1)

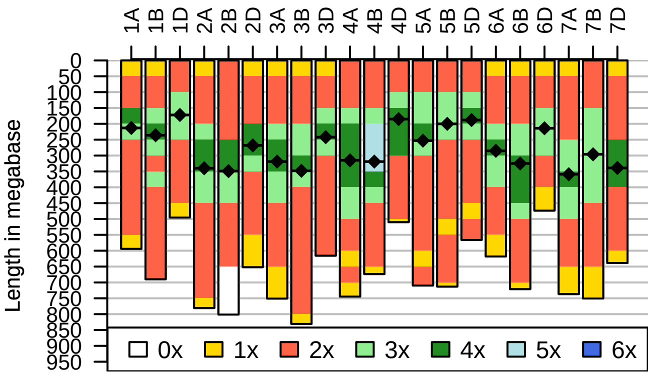

17SI−358−4 (2BL−1)

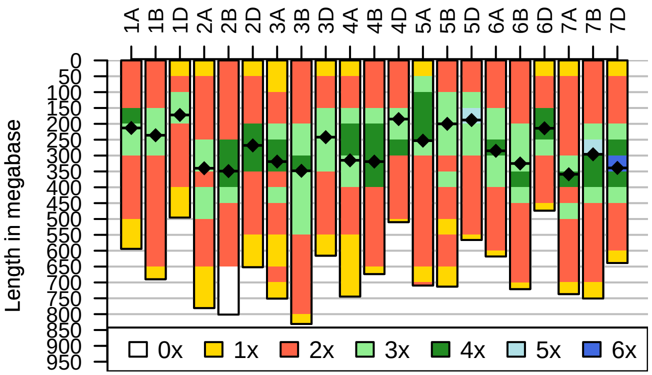

17SI−358−5 (2BL−1)

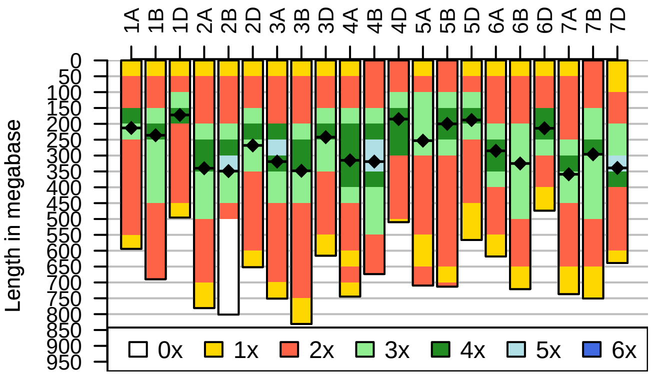

17SI−359−2 (2BL−3)

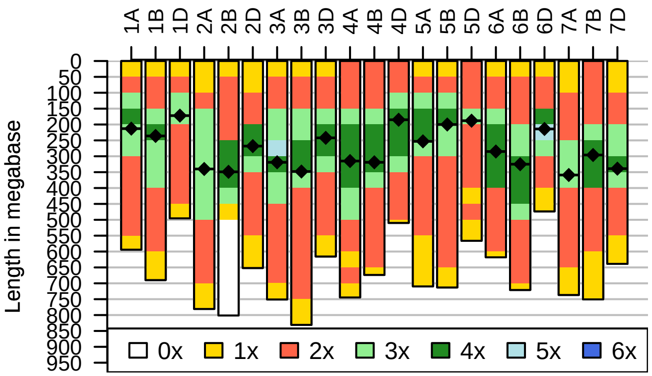

17SI−359−3 (2BL−3)

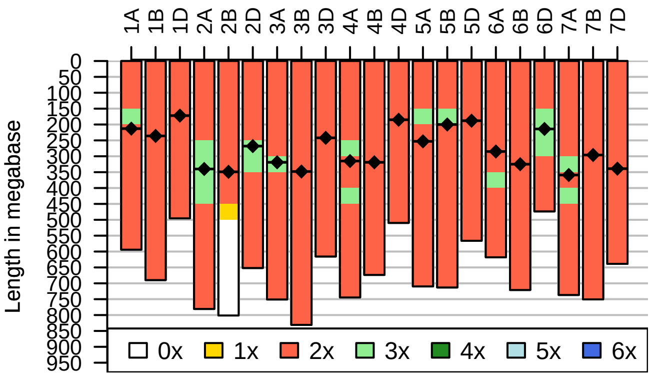

17SI−360−1 (2BL−3)

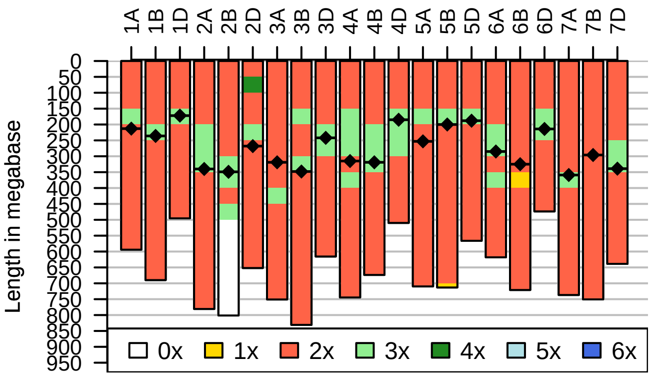

17SI−362−1 (2BL−7)

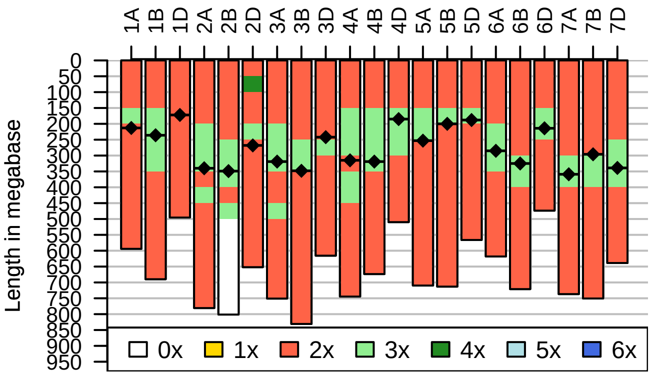

17SI−362−2 (2BL−7)

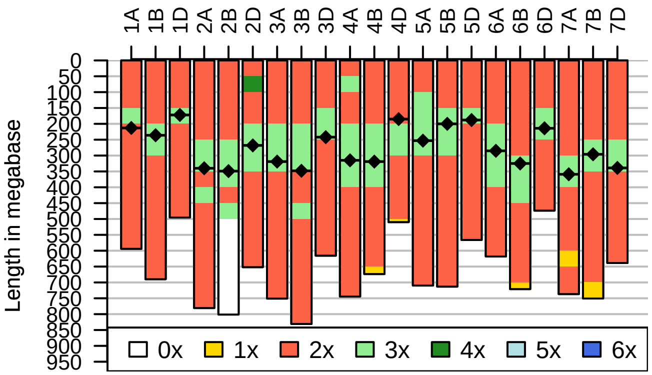

17SI−362−3 (2BL−7)

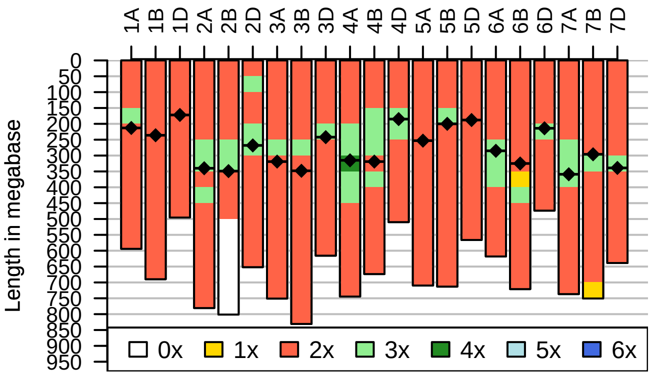

17SI−362−4 (2BL−7)

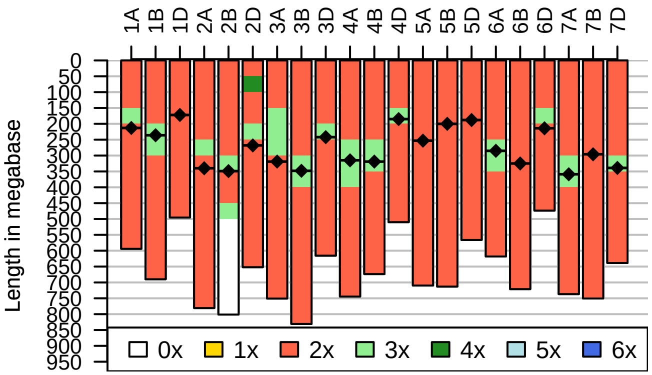

17SI−362−5 (2BL−7)

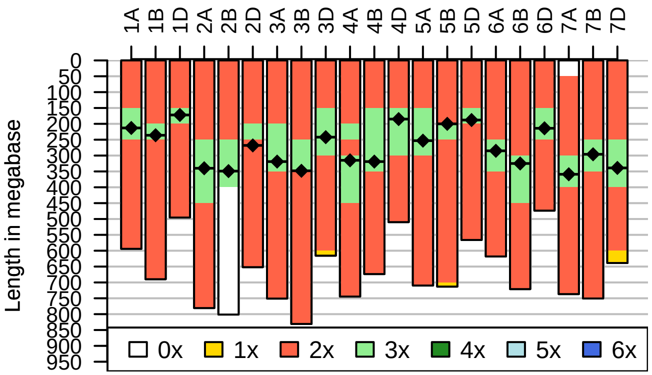

17SI−363−1 (2BL−11, 7AS−1)

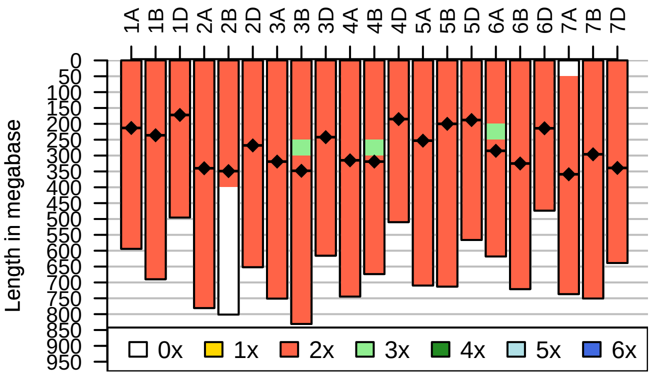

17SI−363−2 (2BL−11, 7AS−1)

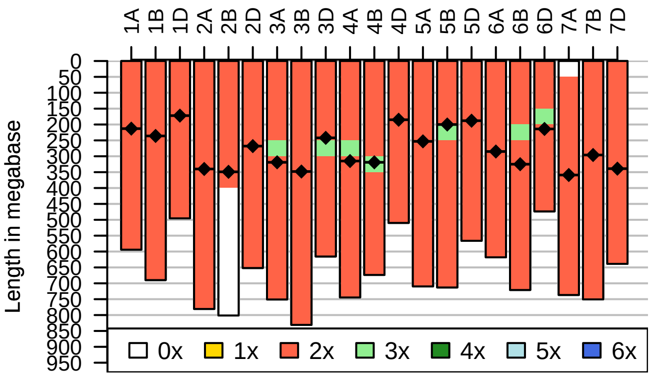

17SI−363−3 (2BL−11, 7AS−1)

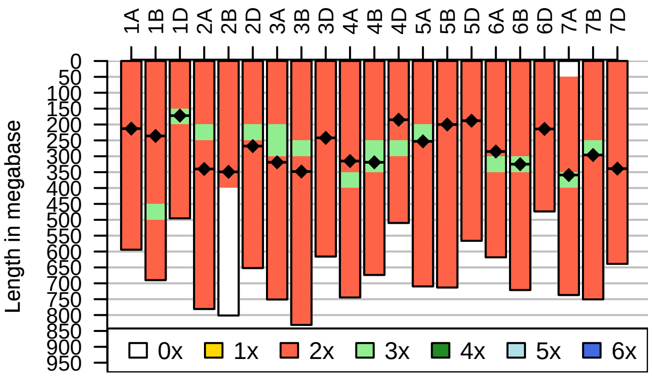

17SI−363−4 (2BL−11, 7AS−1)

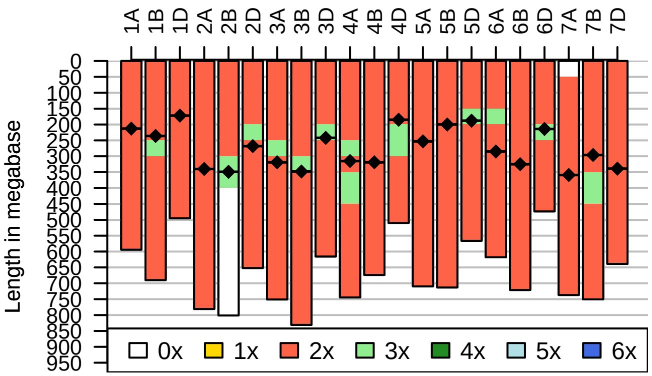

17SI−364−1 (2BL−11, 7AS−1)

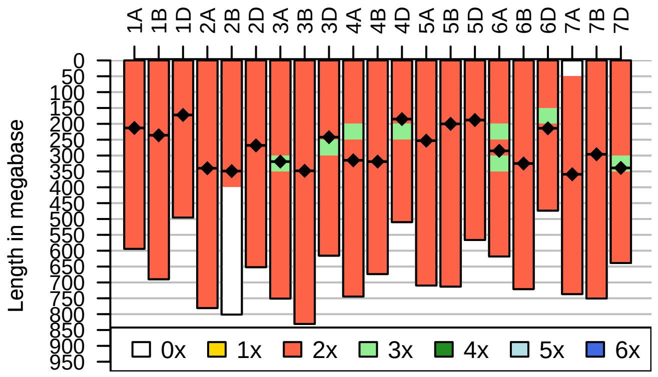

17SI−364−2 (2BL−11, 7AS−1)

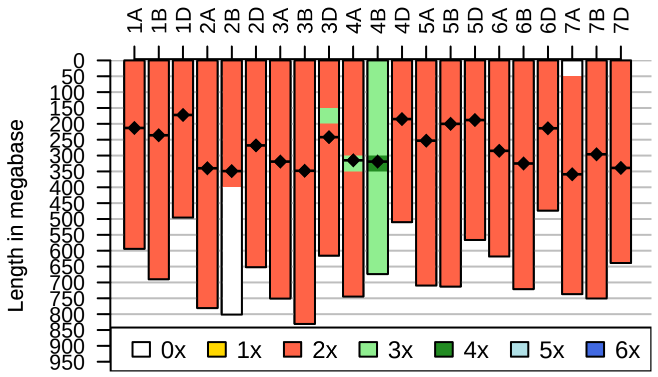

17SI−364−3 (2BL−11, 7AS−1)

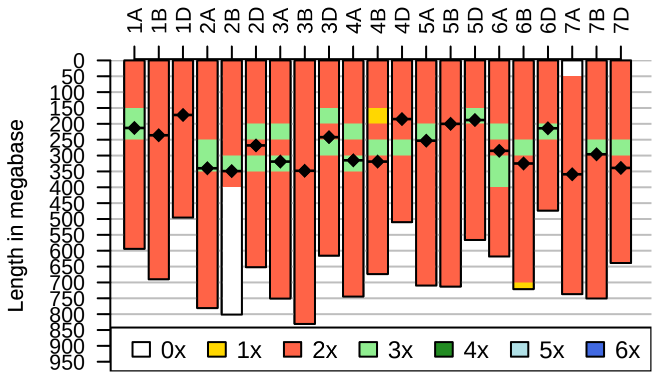

17SI−364−4 (2BL−11, 7AS−1)

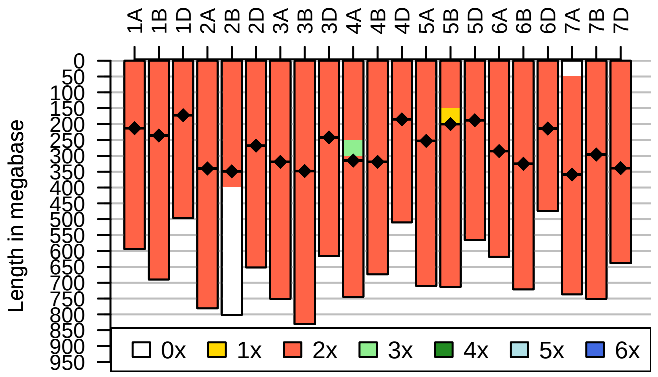

17SI−364−5 (2BL−11, 7AS−1)

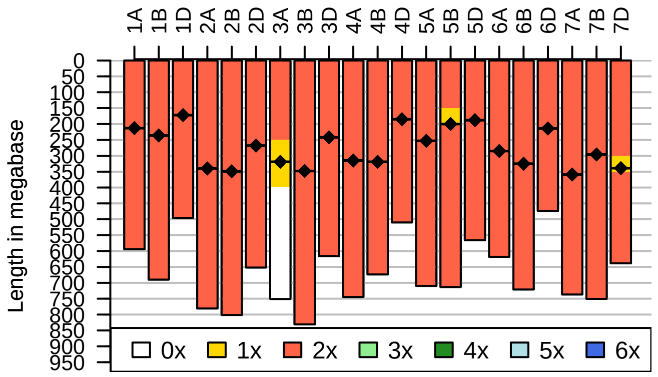

17SI−365−1 (3AL−2)

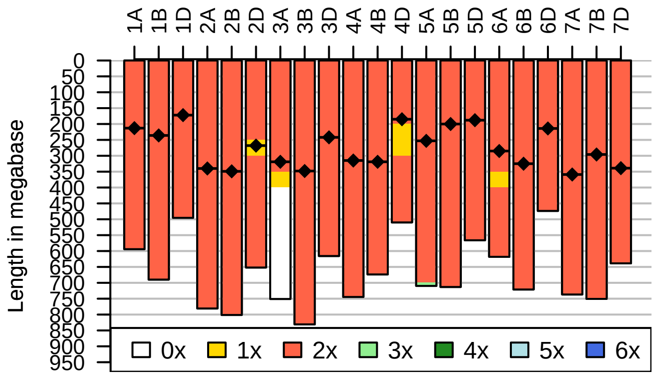

17SI−365−2 (3AL−2)

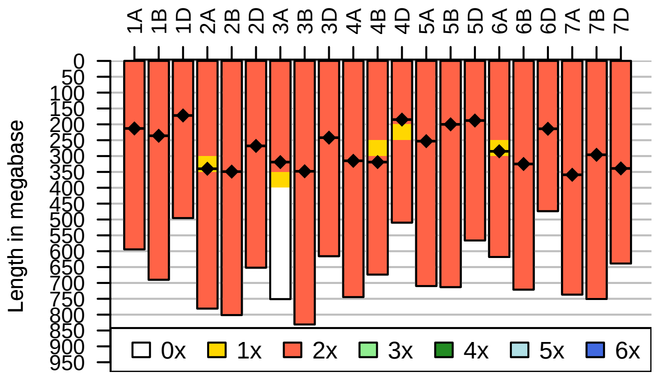

17SI−365−3 (3AL−2)

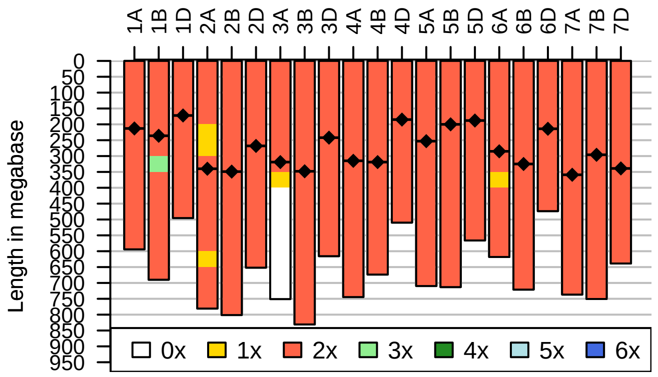

17SI−365−4 (3AL−2)

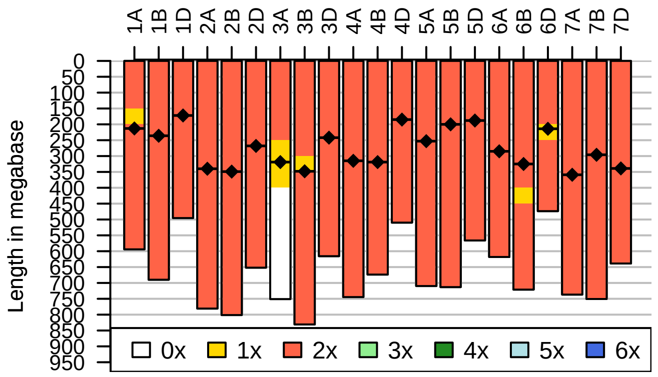

17SI−365−5 (3AL−2)

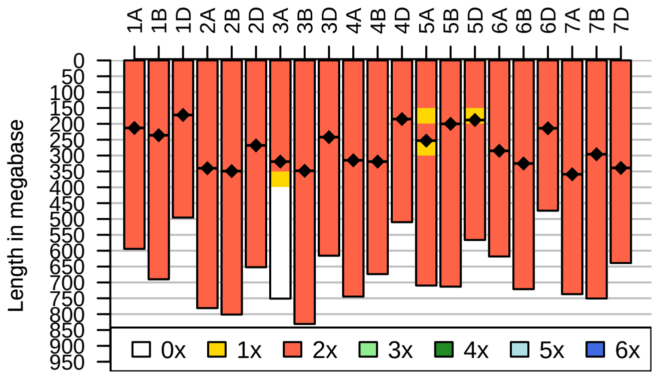

17SI−366 (3AL−2)

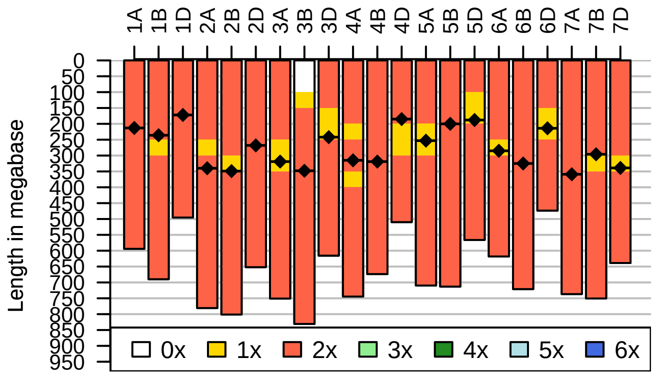

17SI−367−1 (3BS−2)

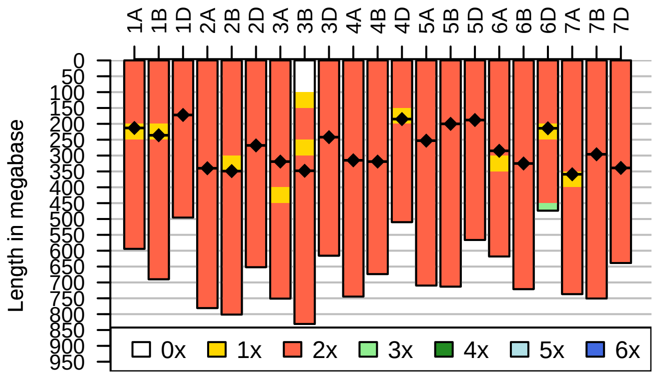

17SI−367−2 (3BS−2)

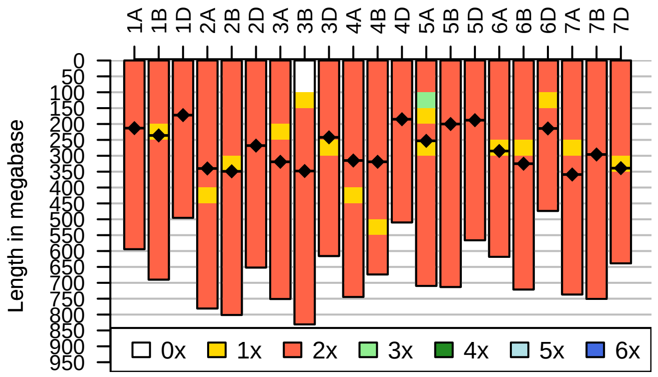

17SI−367−3 (3BS−2)

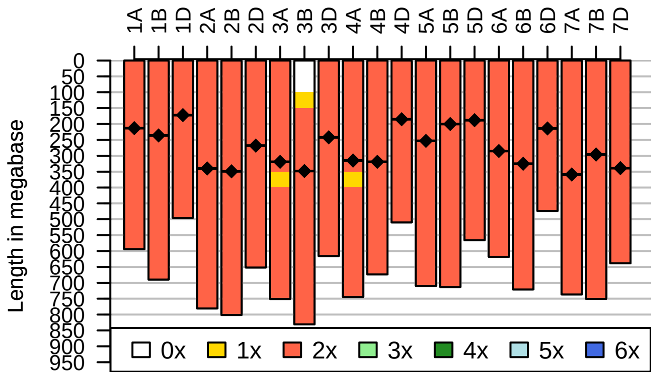

17SI−367−4 (3BS−2)

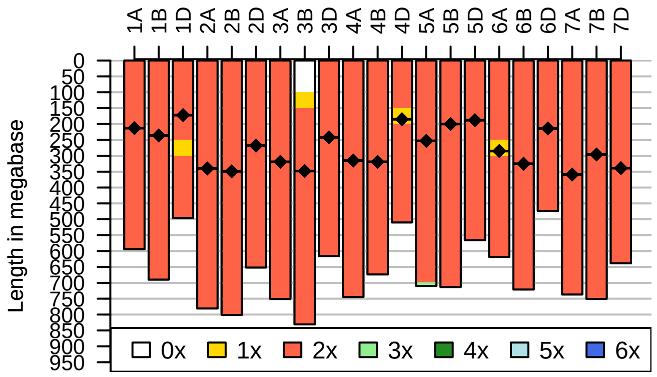

17SI−367−5 (3BS−2)

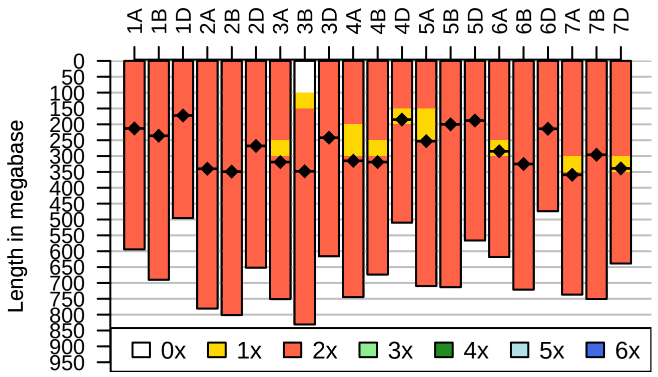

17SI−368−1 (3BS−2)

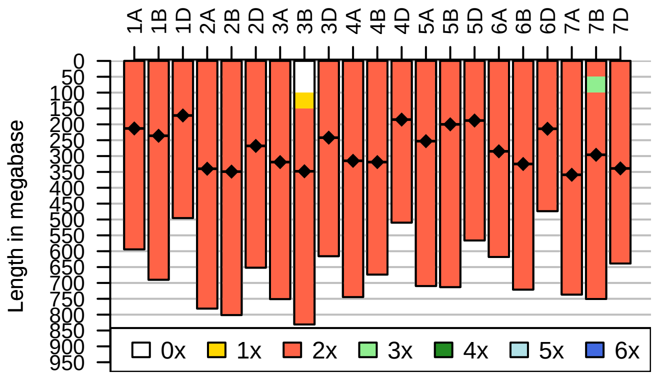

17SI−368−2 (3BS−2)

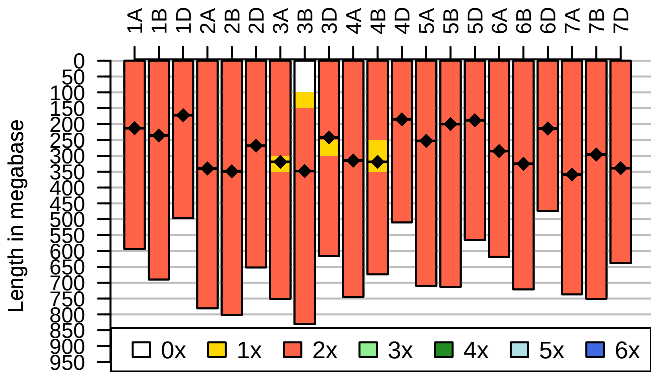

17SI−368−3 (3BS−2)

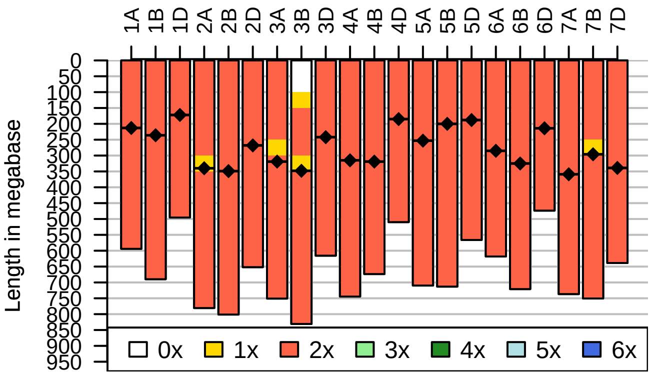

17SI−368−4 (3BS−2)

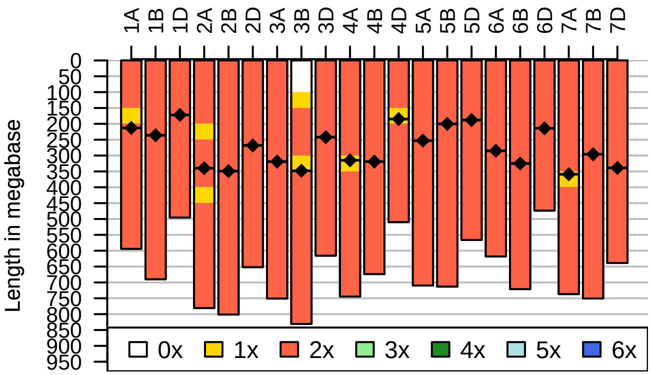

17SI−368−5 (3BS−2)

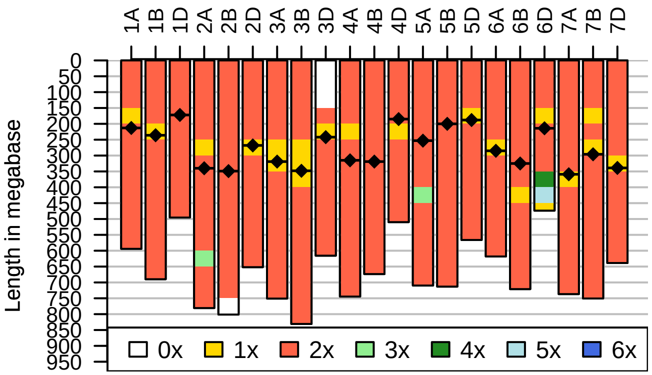

17SI−369 (3DS−1)

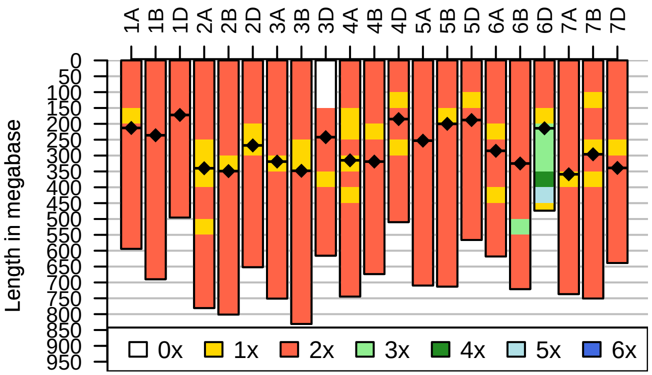

17SI−370−1 (3DS−1)

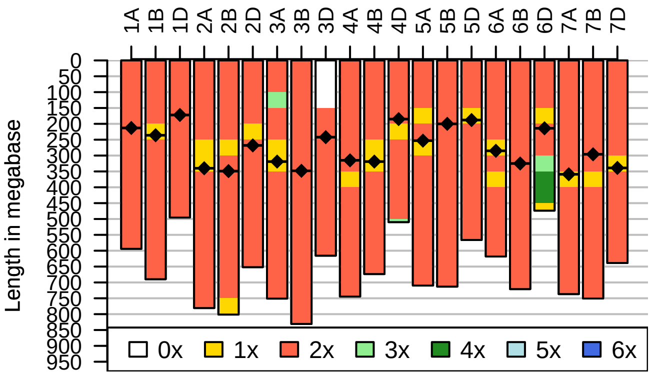

17SI−370−2 (3DS−1)

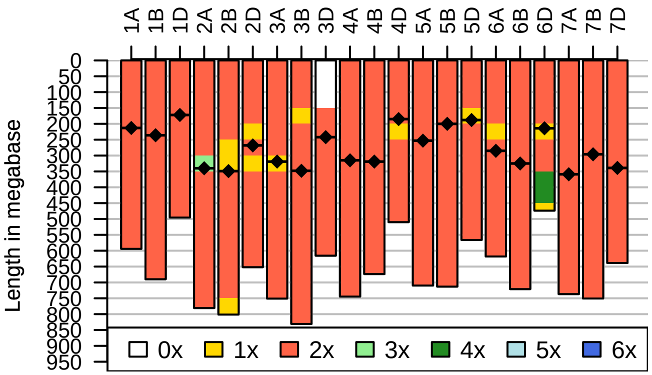

17SI−370−3 (3DS−2)

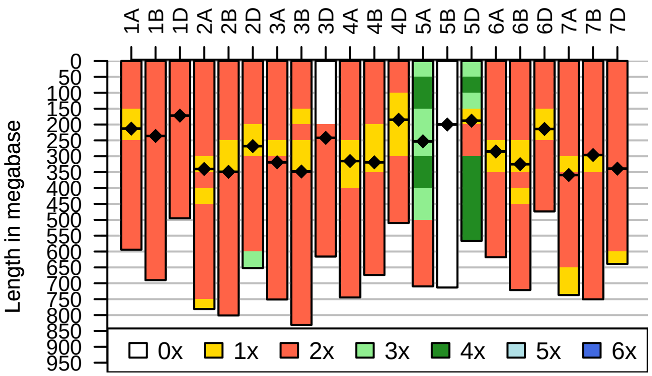

17SI−371−1 (3DS−2)

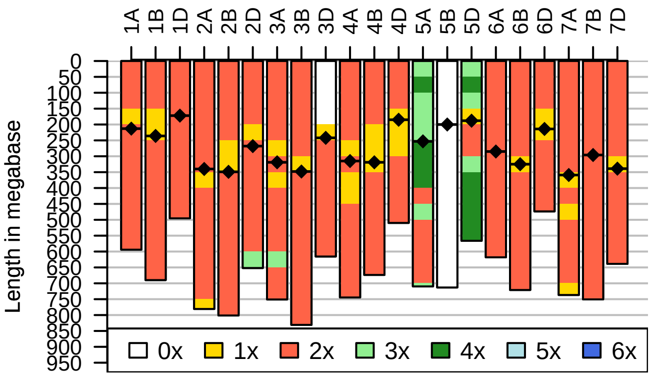

17SI−371−2 (3DS−2)

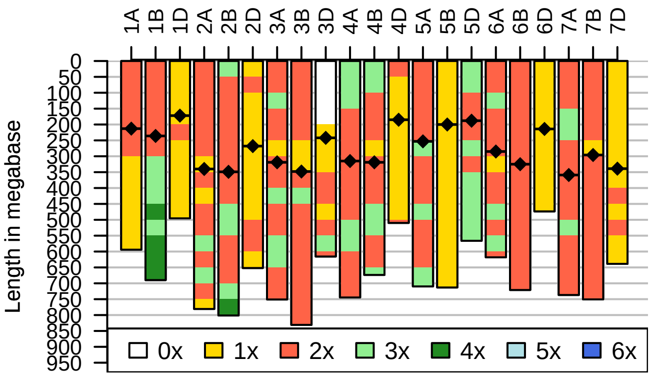

17SI−372−1 (3DS−2)

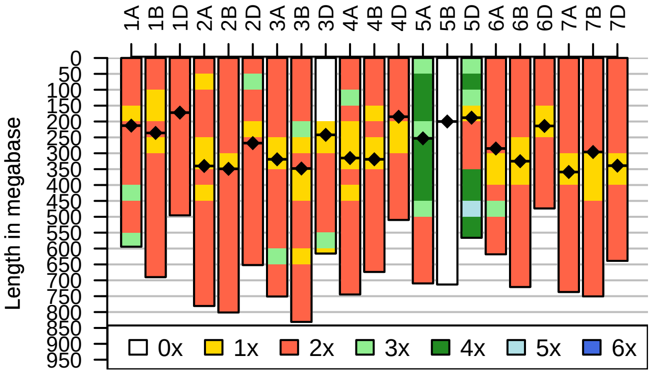

17SI−372−2 (3DS−2)

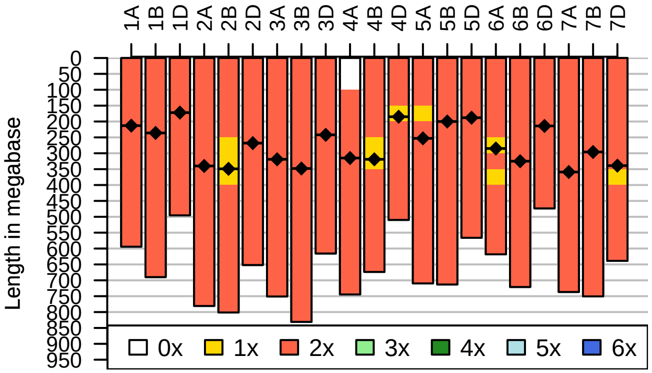

17SI−373−1 (4AS−2)

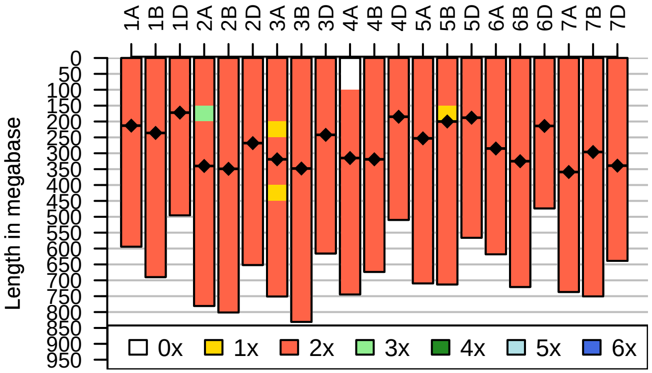

17SI−373−2 (4AS−2)

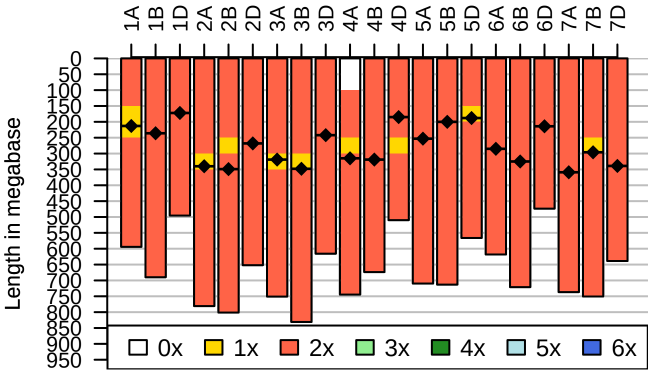

17SI−373−3 (4AS−2)

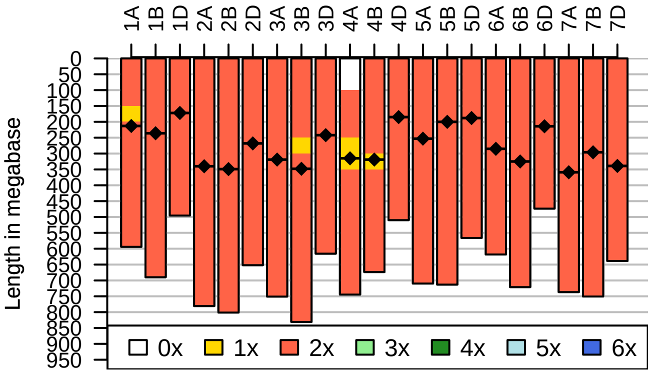

17SI−373−4 (4AS−2)

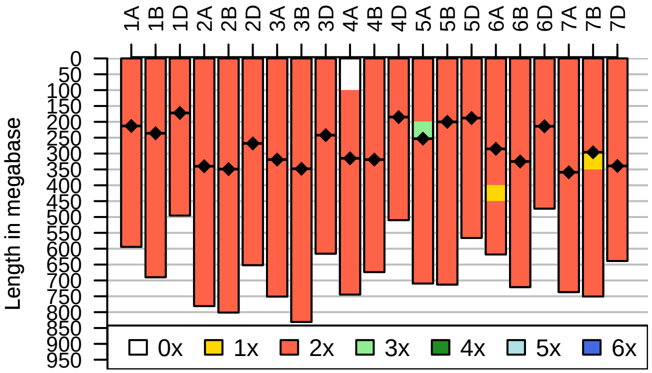

17SI−374−1 (4AS−2)

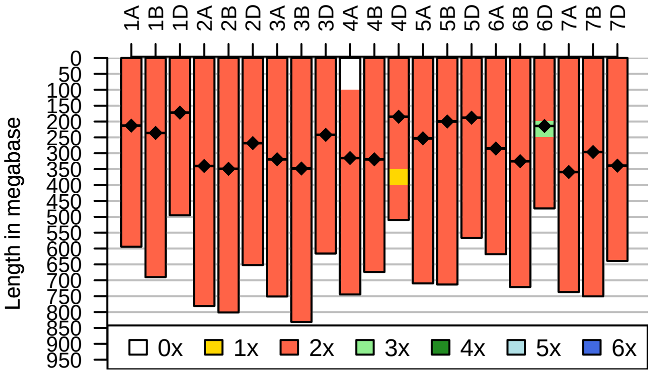

17SI−374−2 (4AS−2)

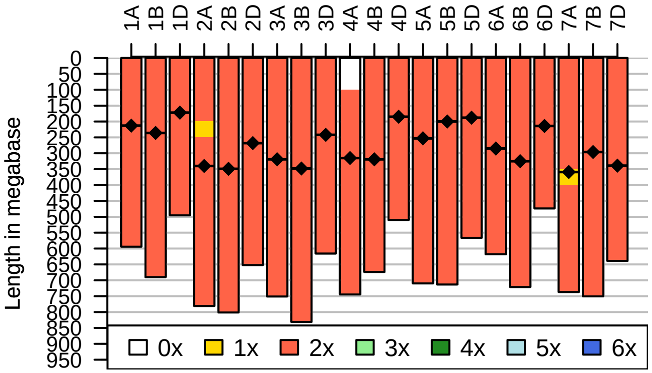

17SI−374−3 (4AS2)

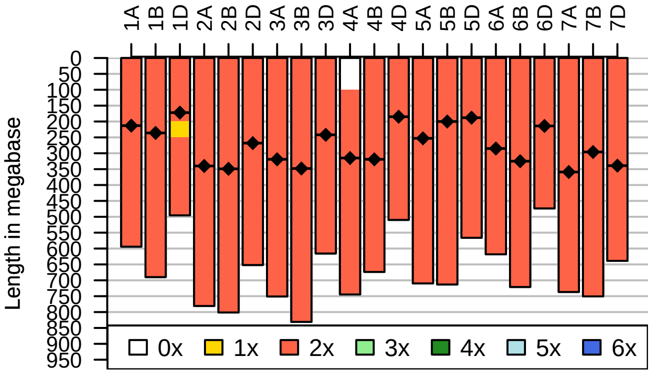

17SI−374−4 (4AS−2)

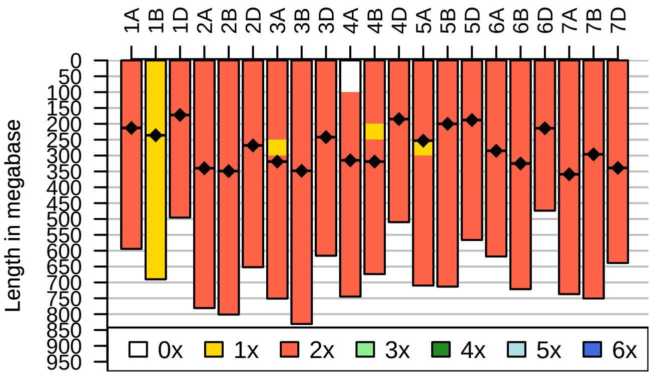

17SI−374−5 (4AS−2)

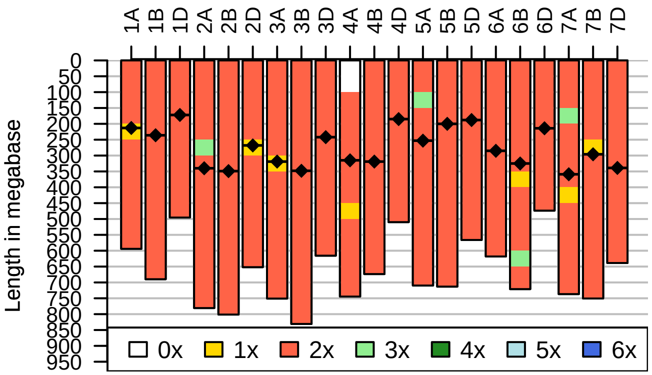

17SI−374−6 (4AS−2)

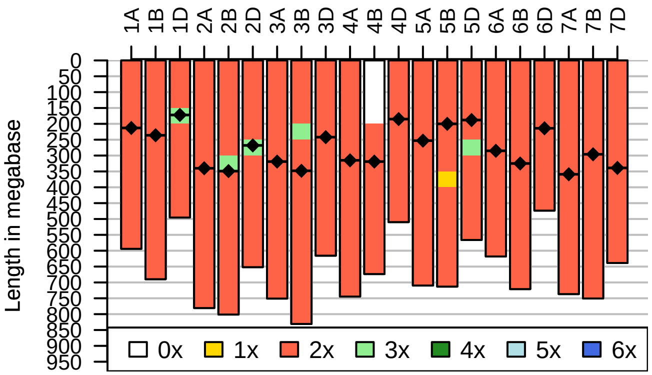

17SI−375−1 (4BS−5, 4BL−11)

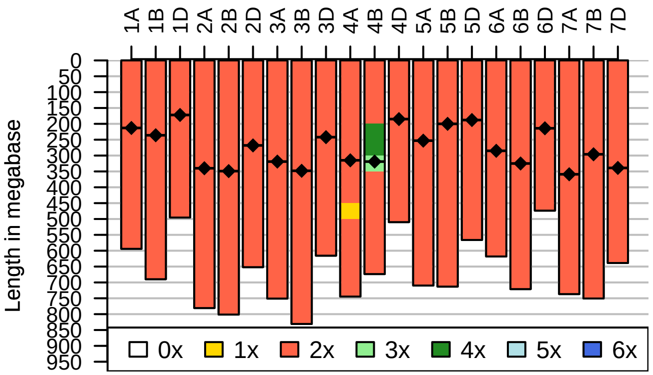

17SI−375−2 (4BS−5, 4BL−11)

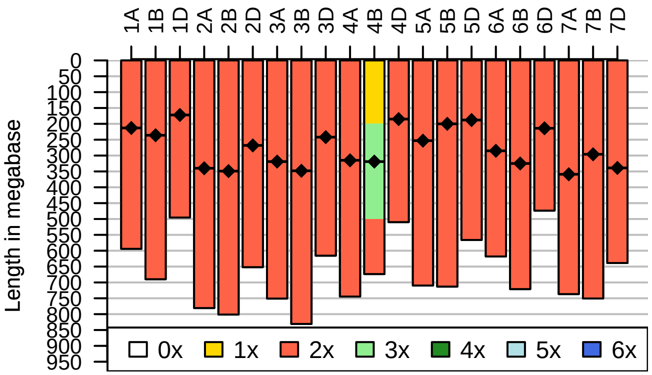

17SI−376−1 (4BS−5, 4BL−11)

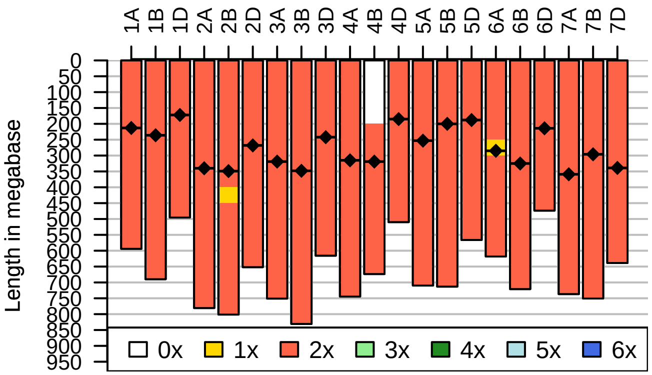

17SI−376−2 (4BS−5, 4BL−11)

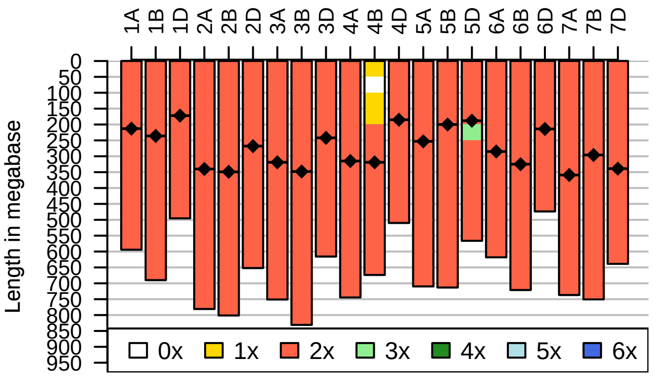

17SI−377−1 (4BS−5, 4BL−11)

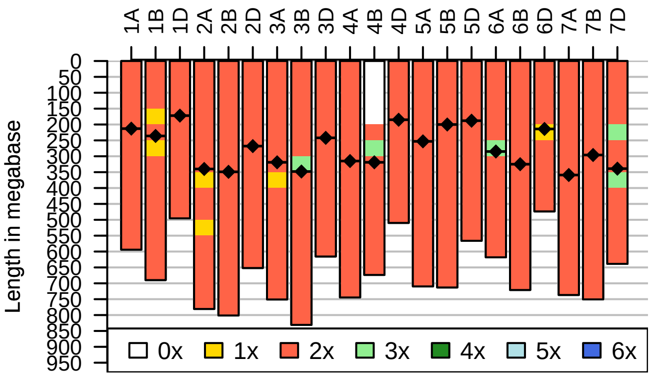

17SI−377−2 (4BS−5, 4BL−11)

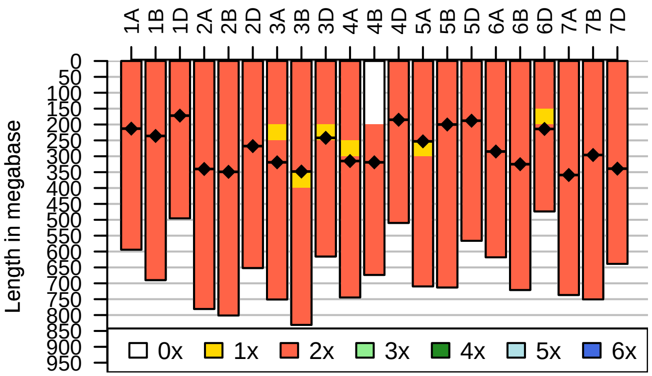

17SI−377−3 (4BS−5, 4BL−11)

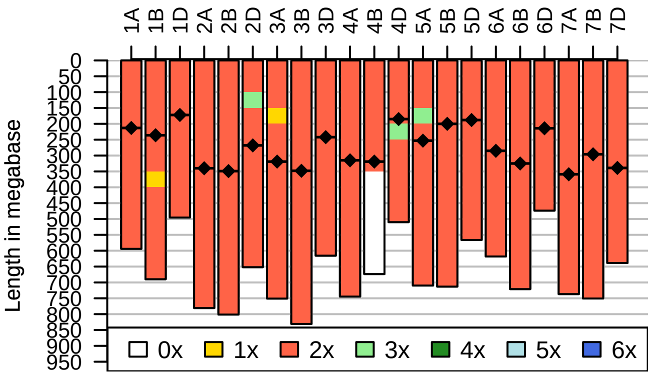

17SI−378−1 (4BL−6)

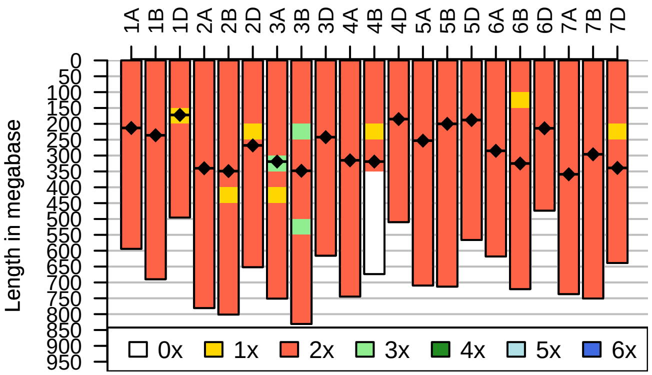

17SI−378−2 (4BL−6)

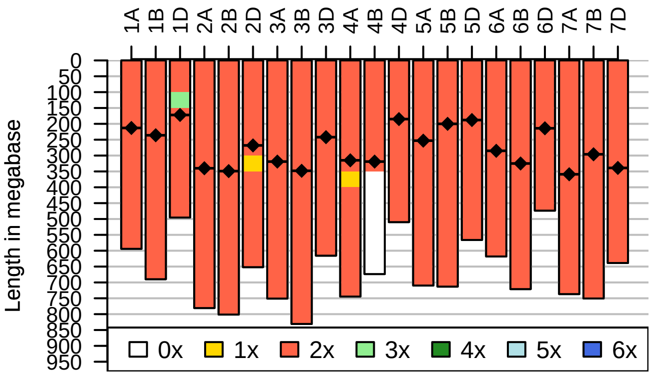

17SI−378−3 (4BL−6)

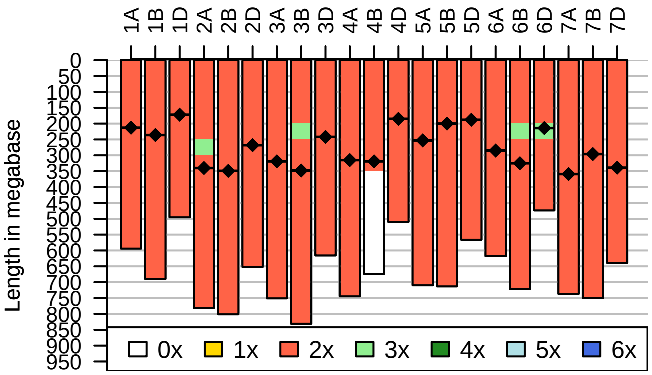

17SI−378−4 (4BL−6)

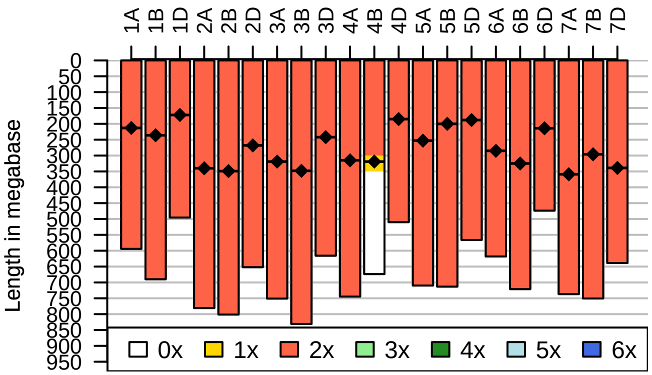

17SI−378−5 (4BL−6)

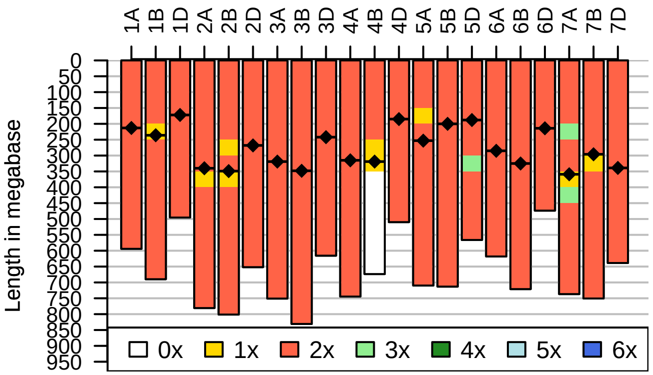

17SI−379−1 (4BL−6)

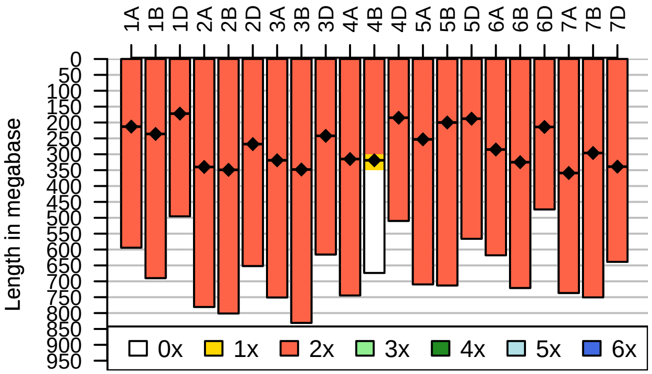

17SI−379−2 (4BL−6)

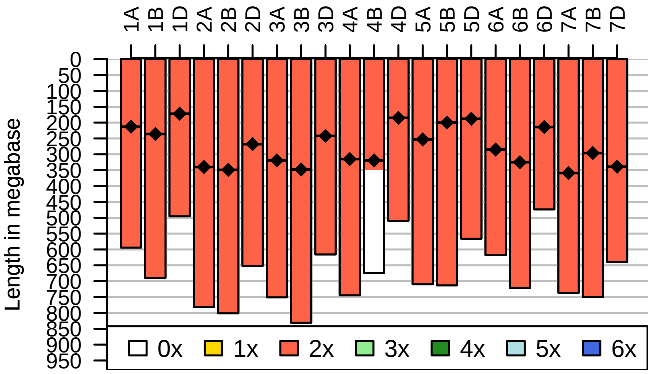

18S1−209−1 (Dt4BS)

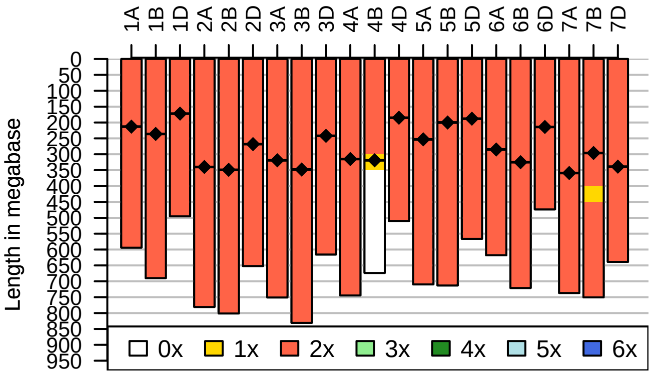

18S1−209−17 (Dt4BS)

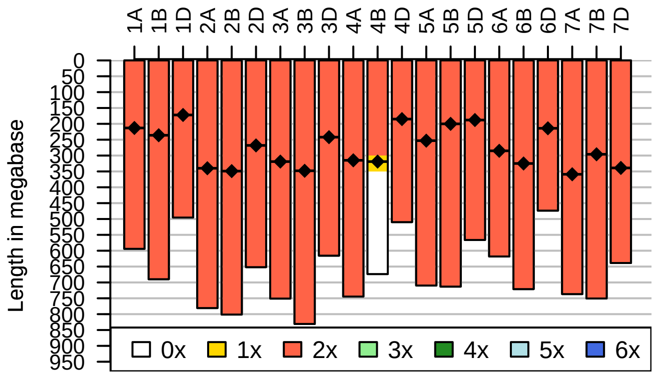

18S1−209−5 (Dt4BS)

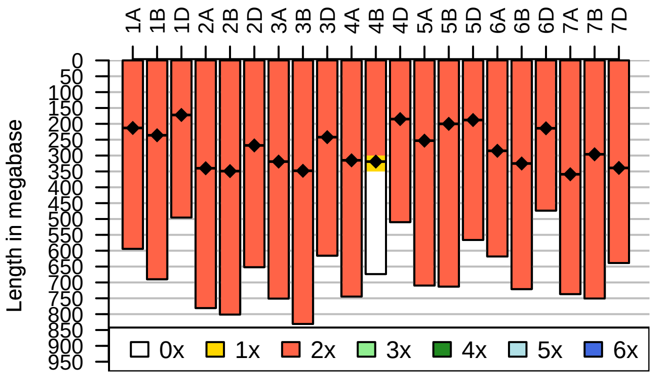

18S1−209−7 (Dt4BS)

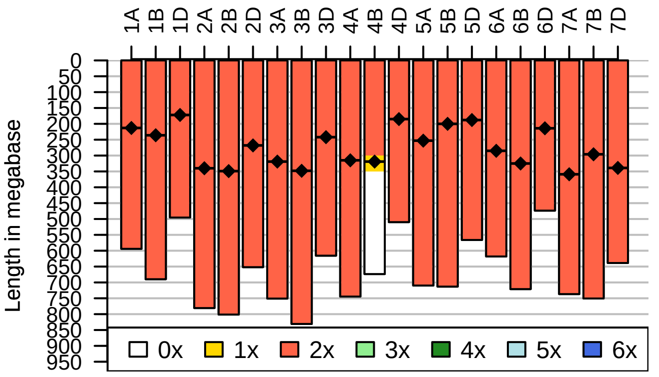

18S1−209−9 (Dt4BS)

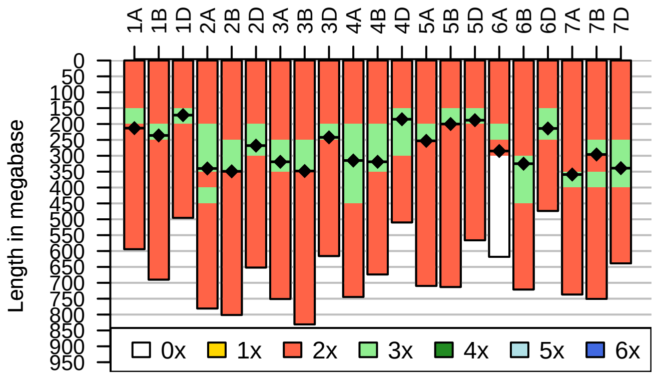

18S1−210−1 (Dt6AS)

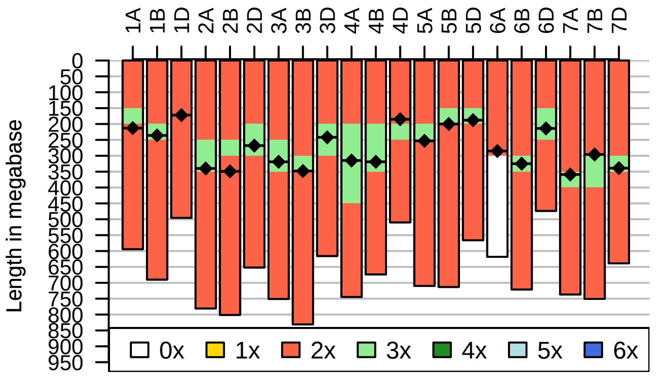

18S1−210−11 (Dt6AS)

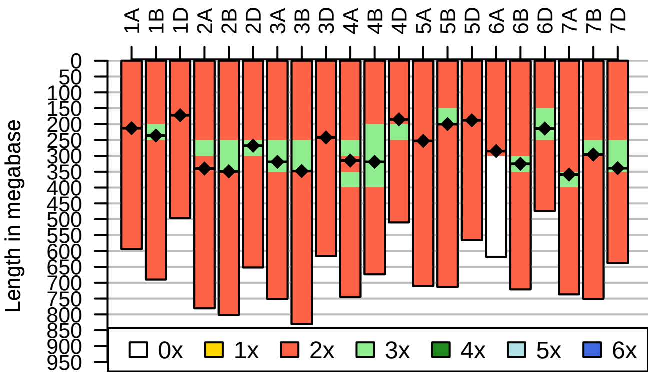

18S1−210−14 (Dt6AS)

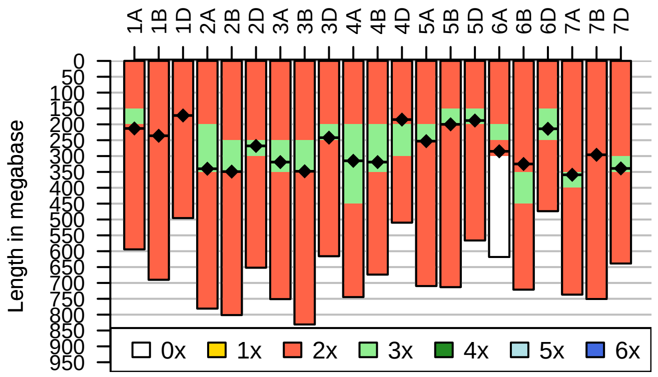

18S1−210−18 (Dt6AS)

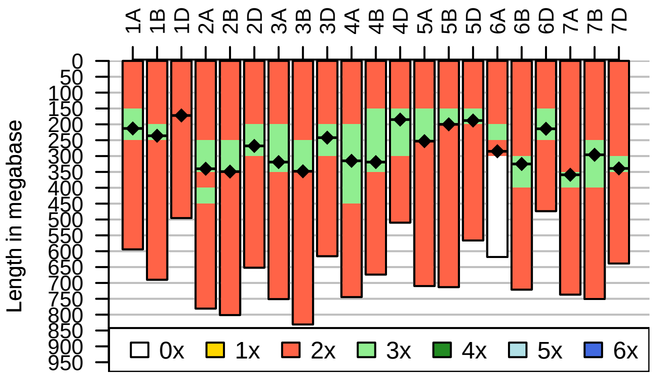

18S1−210−6 (Dt6AS)

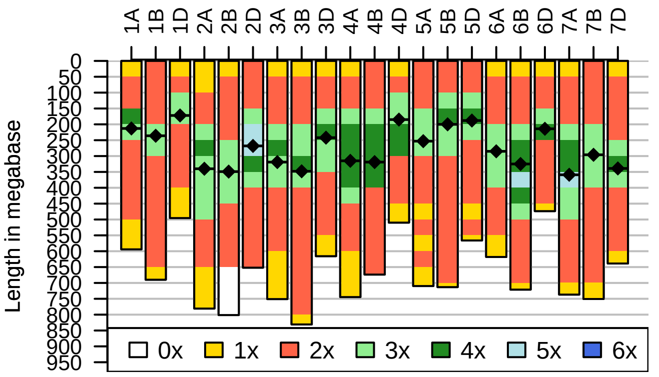

18S1−216−1 (2BL−1)

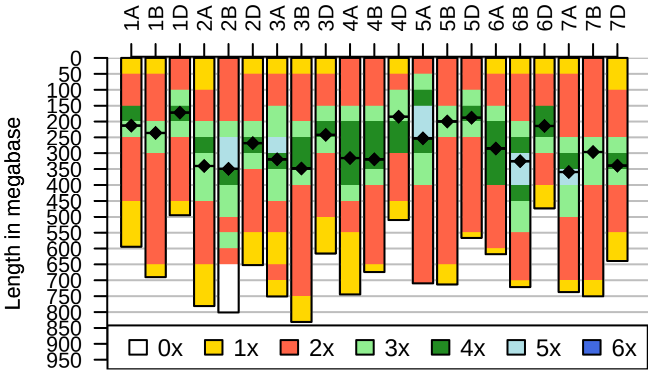

18S1−216−2 (2BL−1)

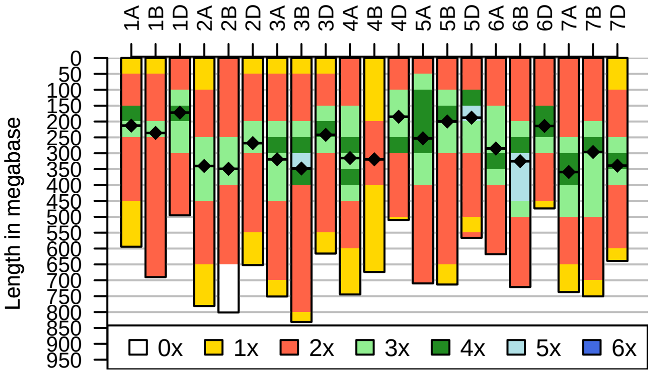

18S1−216−3 (2BL−1)

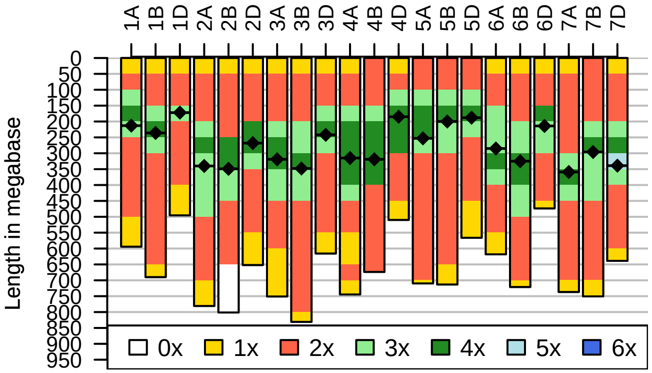

18S1−217−1 (2BL−1)

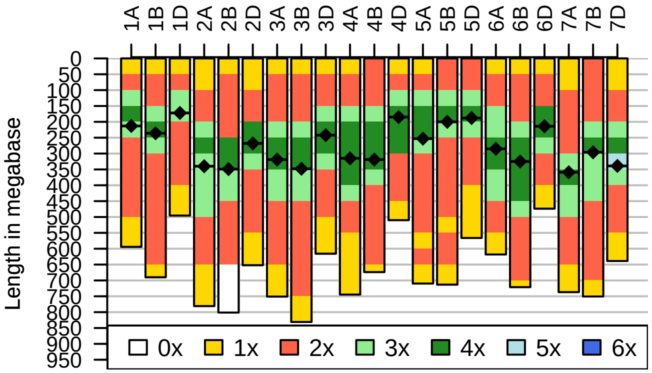

18S1−217−2 (2BL−1)

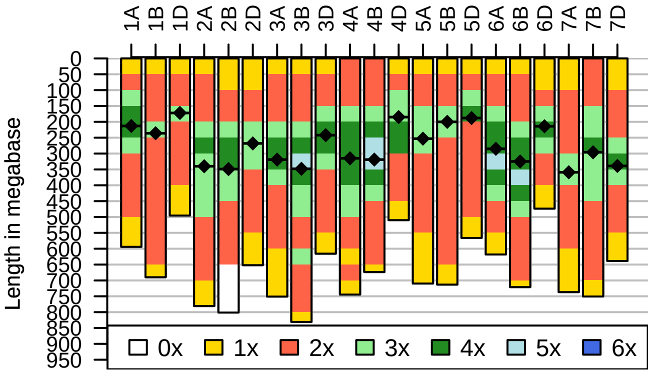

18S1−217−3 (2BL−1)

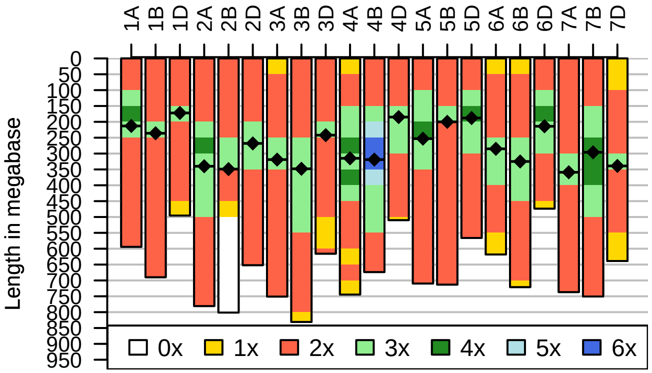

18S1−218−1 (2BL−3)

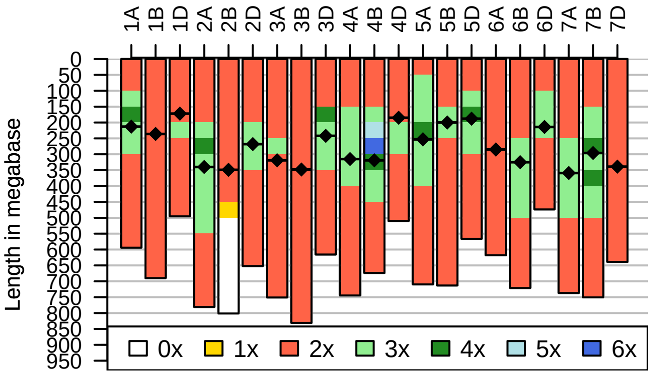

18S1−218−2 (2BL−3)

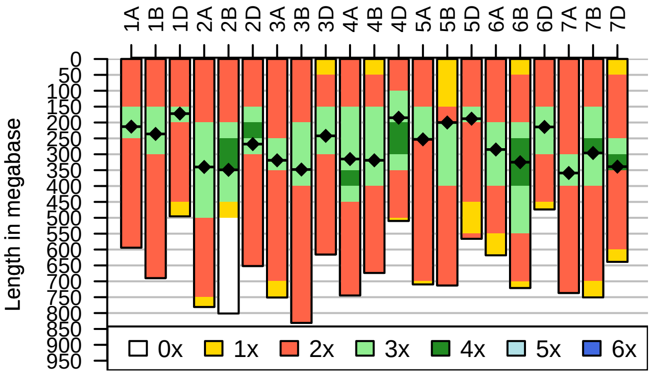

18S1−218−3 (2BL−3)

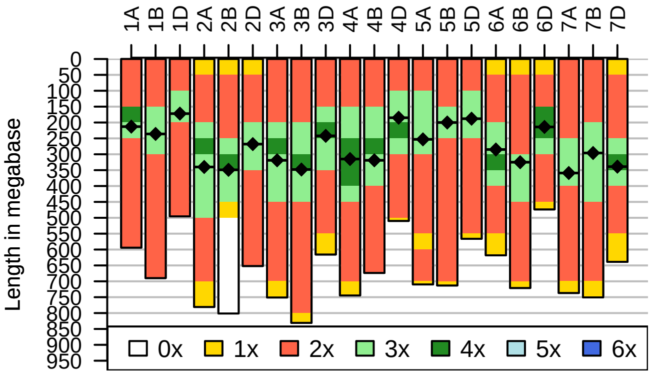

18S1−219−1 (2BL−3)

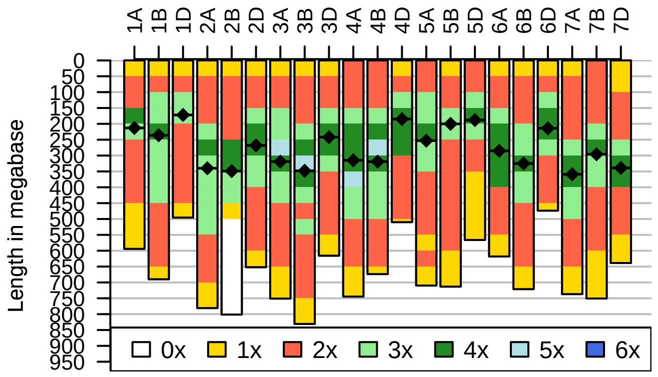

18S1−219−2 (2BL−3)

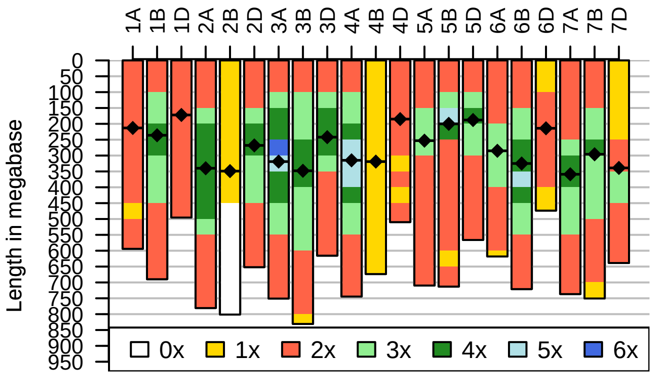

18S1−219−3 (2BL−3)

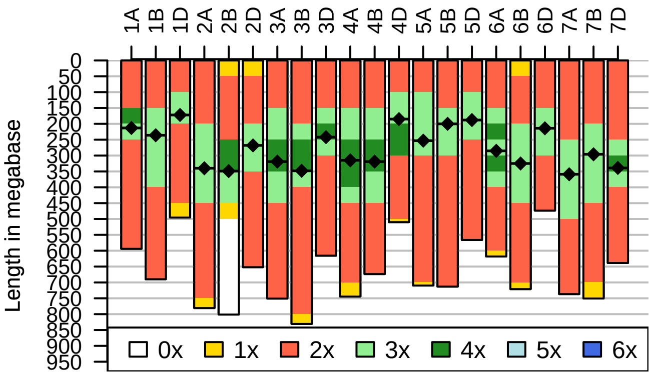

18S1−220−1 (2BL−3)

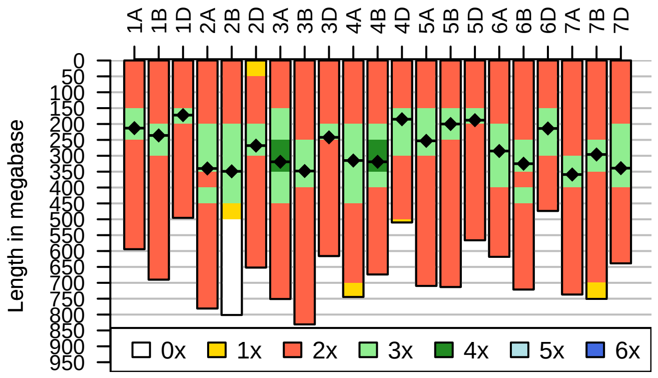

18S1−220−2 (2BL−3)

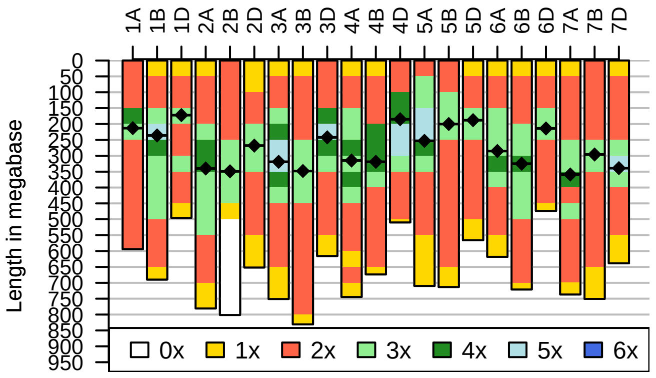

18S1−220−3 (2BL−3)

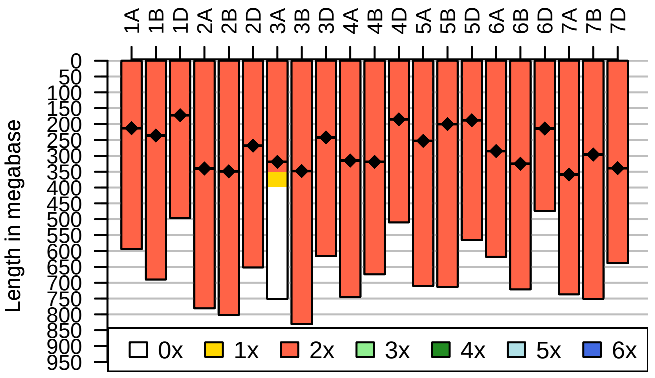

18S1−221−1 (3AL−2)

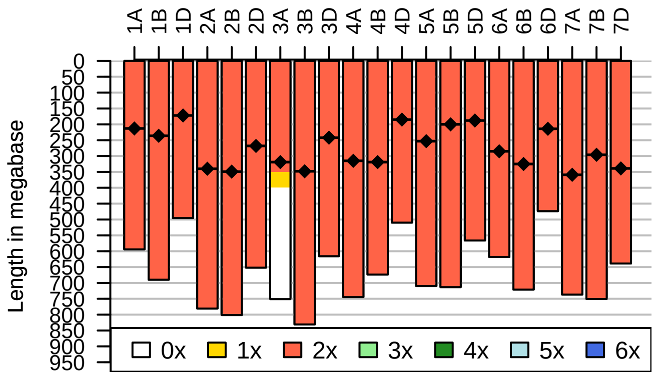

18S1−221−2 (3AL−2)

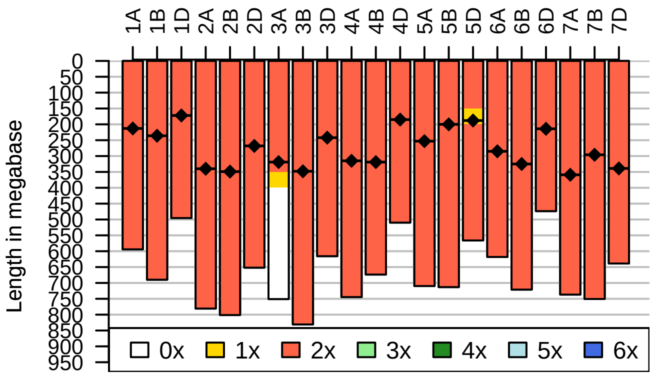

18S1−221−3 (3AL−2)

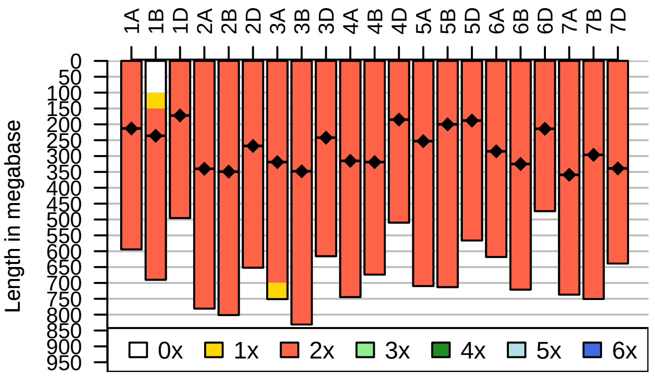

18S1−222−1 (3AL−2)

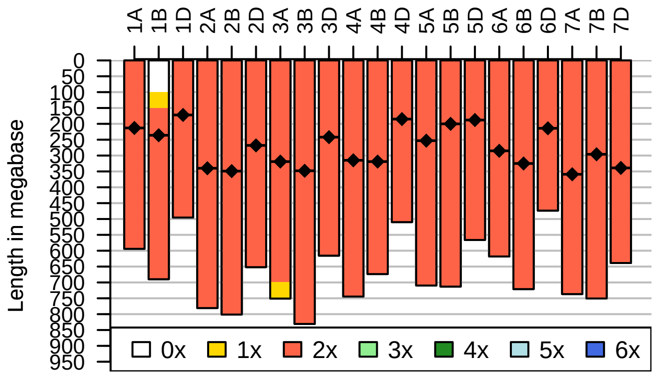

18S1−222−2 (3AL−2)

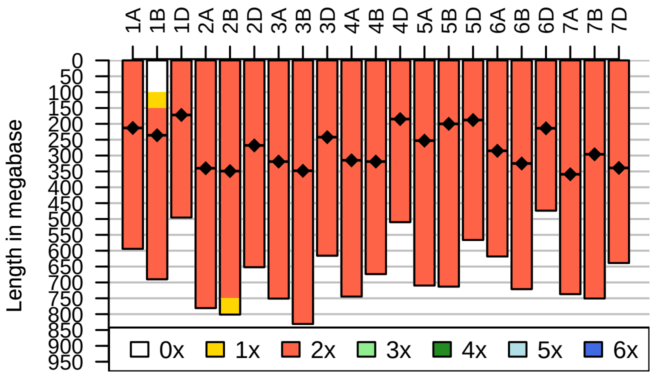

18S1−223−1 (3AL−2)

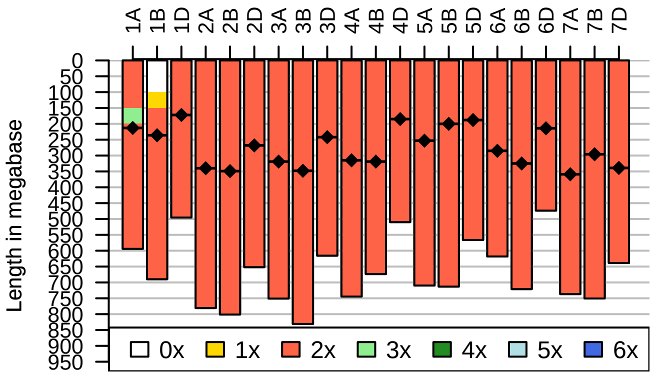

18S1−223−2 (3AL−2)

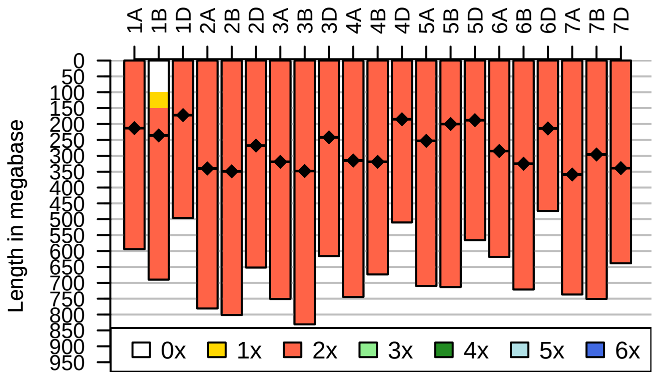

18S1−223−3 (3AL−2)

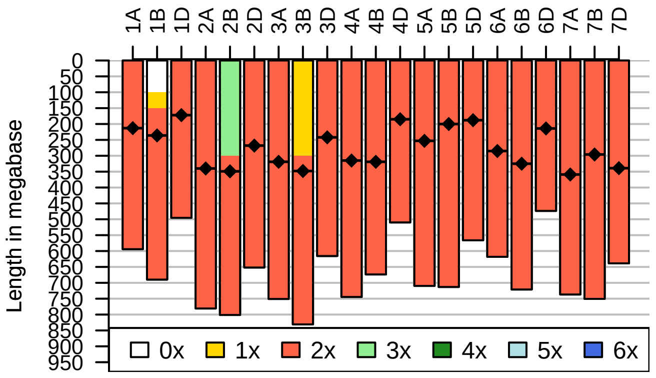

18S1−223−4 (3AL−2)

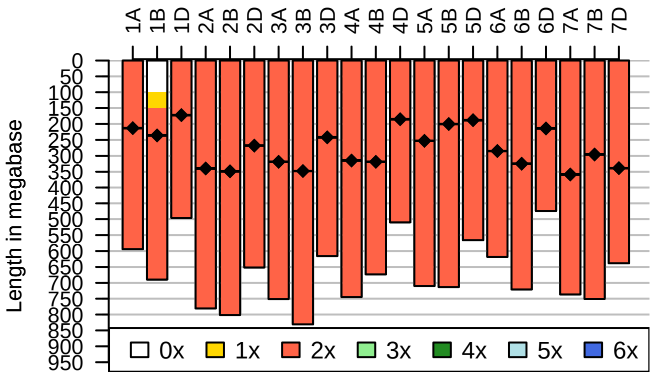

18S1−223−5 (3AL−2)

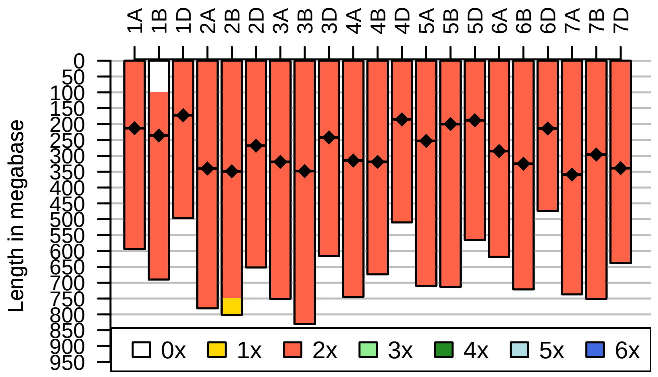

18S1−223−6 (3AL−2)

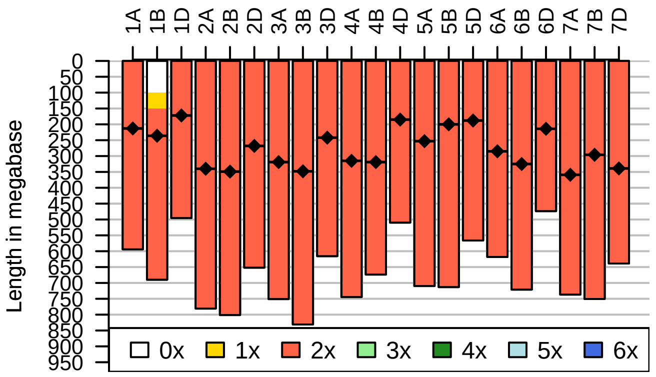

18S1−223−7 (3AL−2)

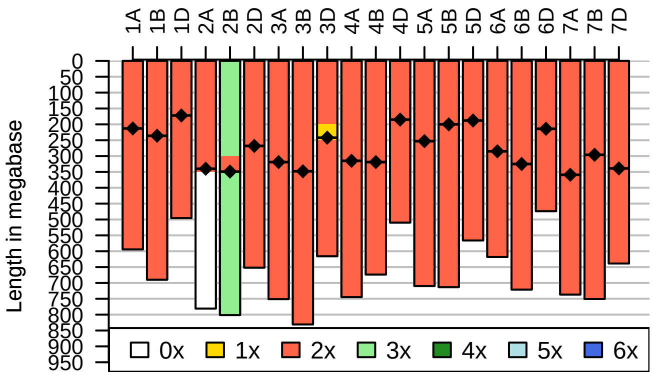

18S1−224−1 (2AL−2=Dt2AS)

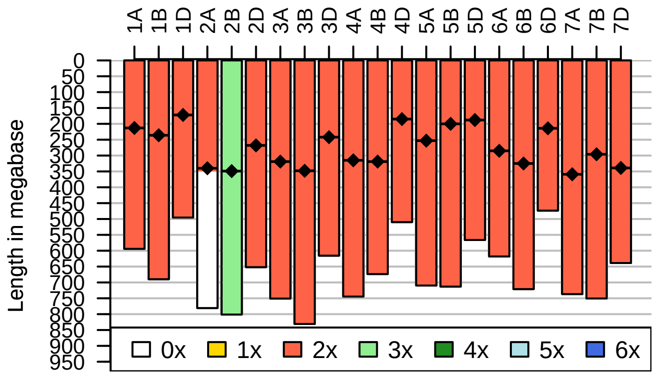

18S1−224−2 (2AL−2=Dt2AS)

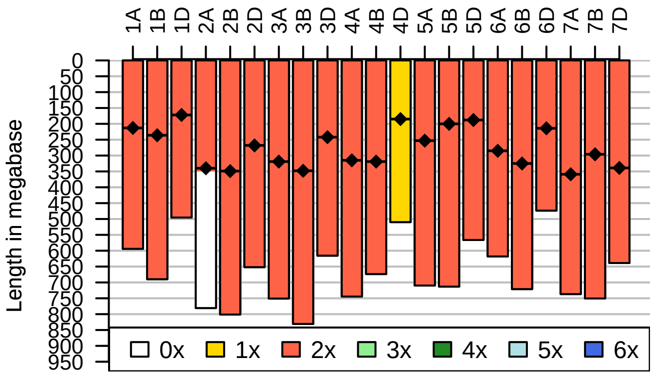

18S1−224−3 (2AL−2=Dt2AS)

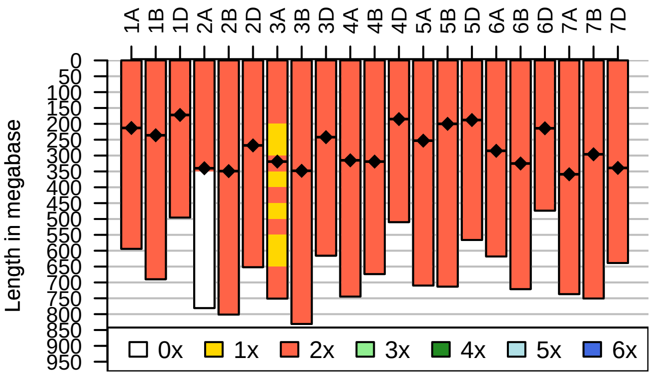

18S1−224−4 (2AL−2=Dt2AS)

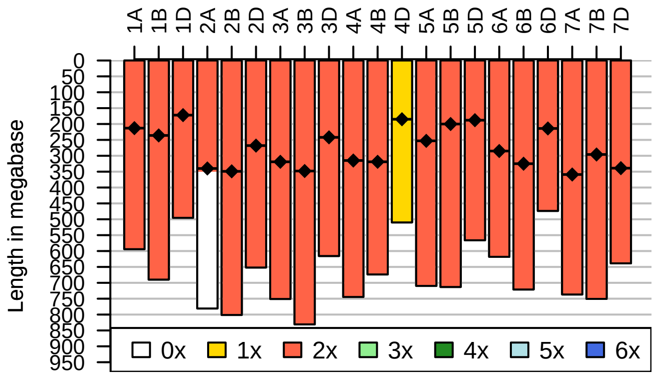

18S1−224−5 (2AL−2=Dt2AS)

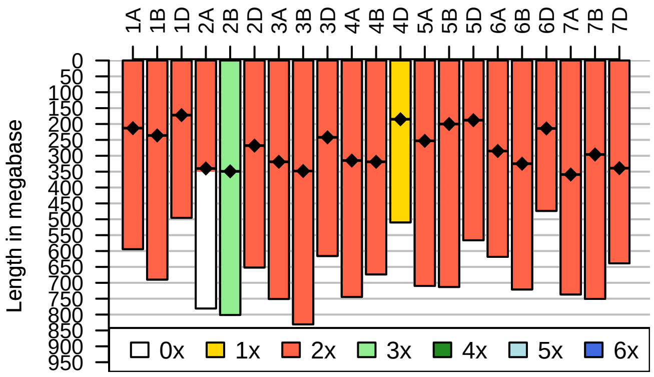

18S1−224−6 (2AL−2=Dt2AS)

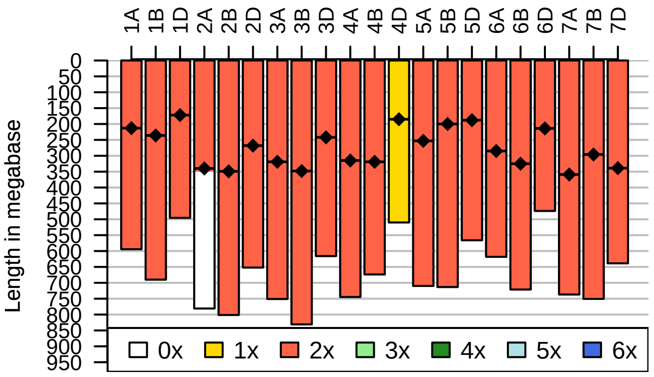

18S1−224−7 (2AL−2=Dt2AS)

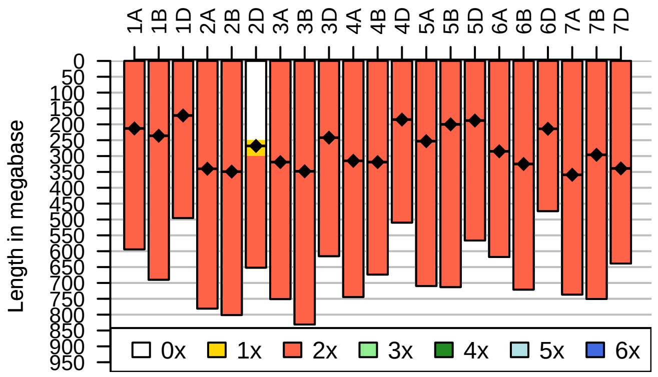

18S1−225−1 (2DS=Dt2DL)

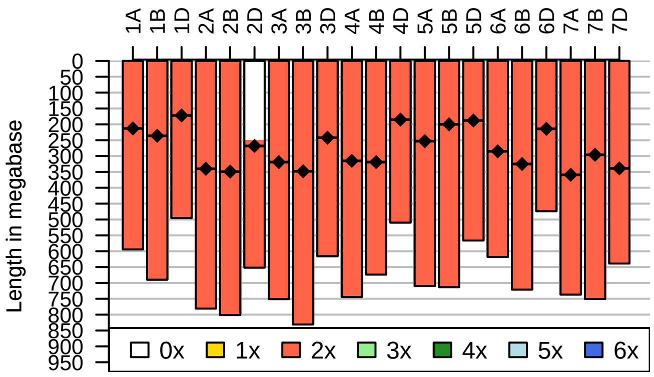

18S1−225−2 (2DS=Dt2DL)

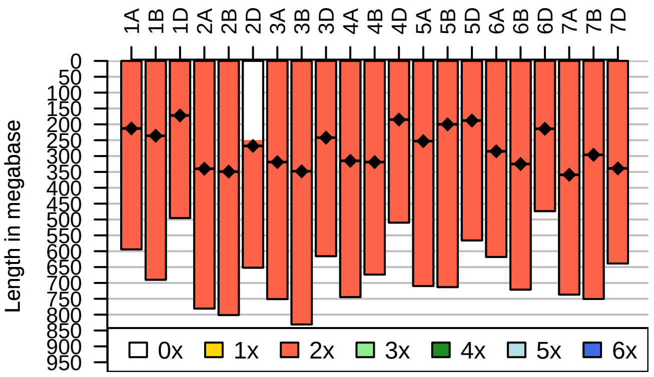

18S1−225−3 (2DS=Dt2DL)

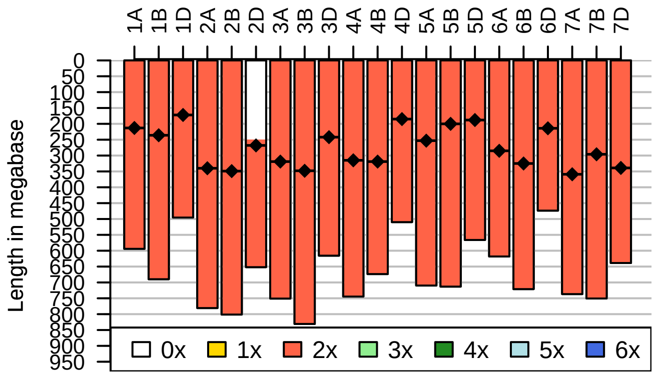

18S1−225−4 (2DS=Dt2DL)

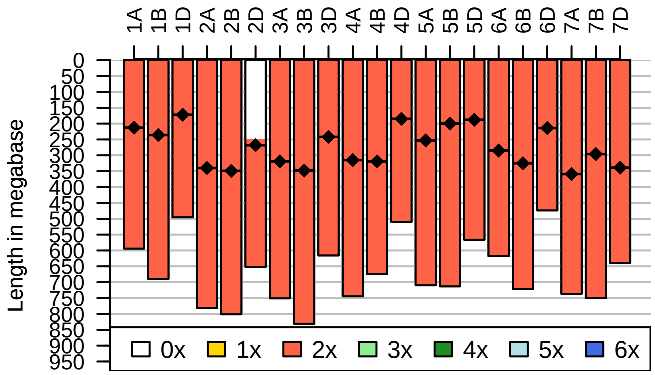

18S1−225−5 (2DS=Dt2DL)

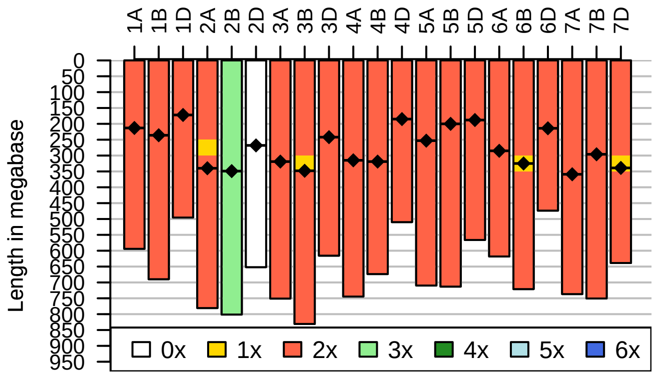

18S1−231 (2DL−1)

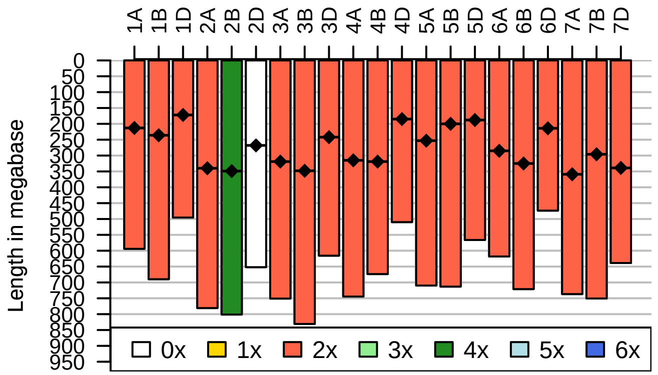

18S1−232−1 (2DL−2)

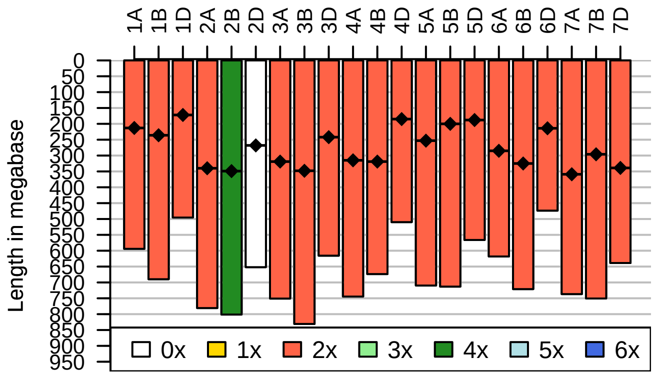

18S1−232−2 (2DL−2)

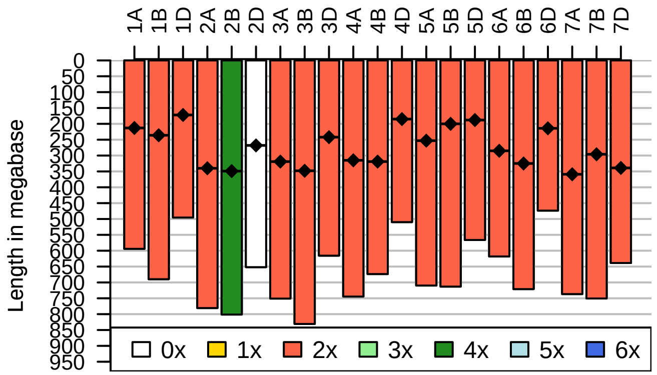

18S1−232−3 (2DL−2)

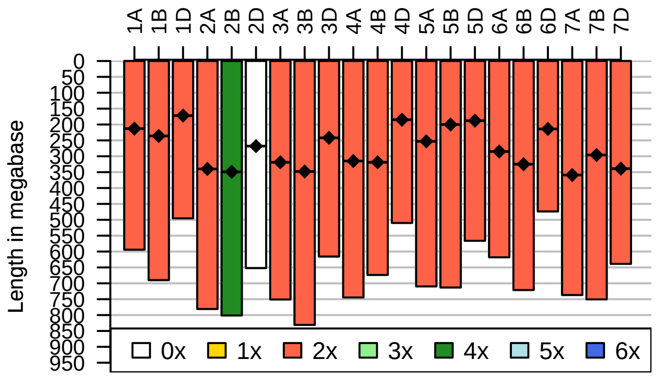

18S1−232−4 (2DL−2)

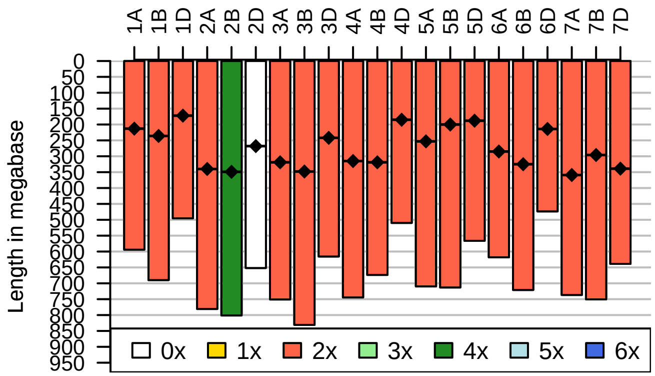

18S1−232−5 (2DL−2)

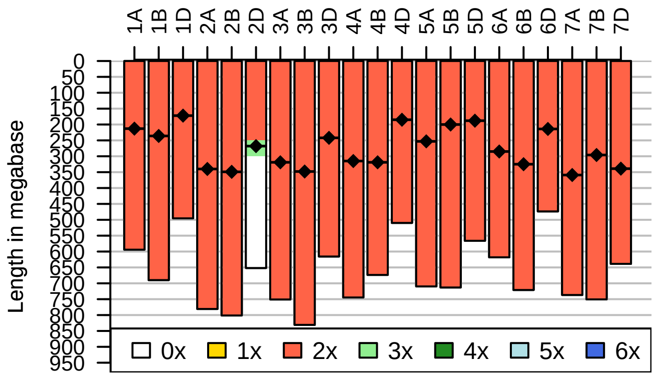

18S1−233 (2DL−7)

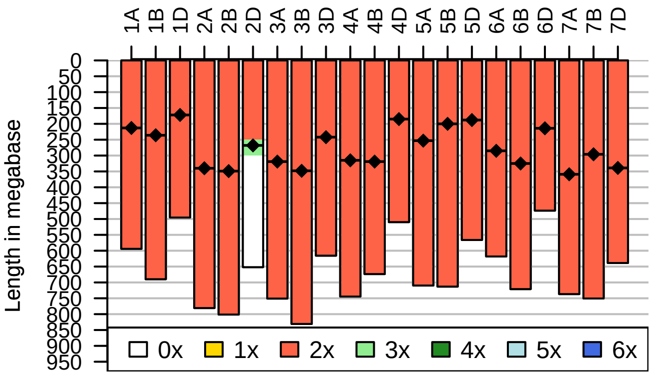

18S1−234−1 (2DL−7)

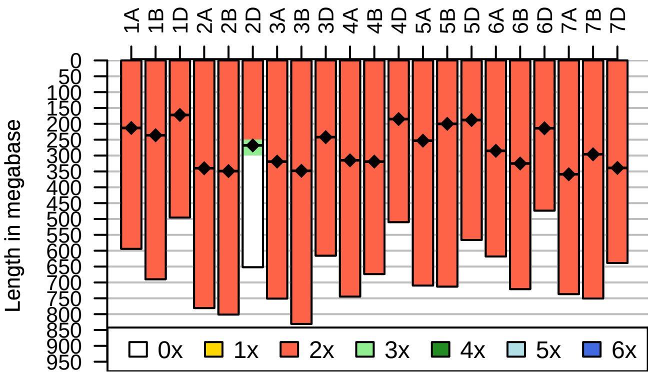

18S1−234−2 (2DL−7)

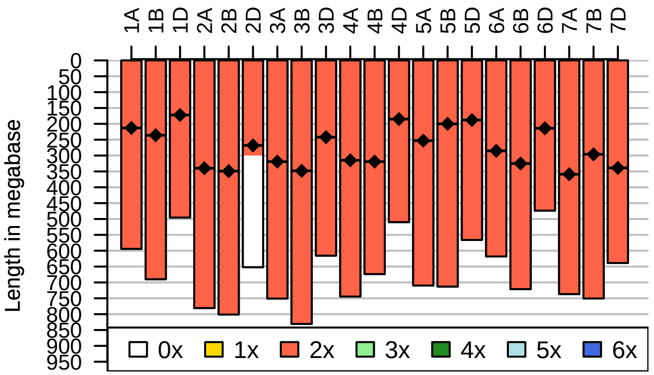

18S1−234−3 (2DL−7)

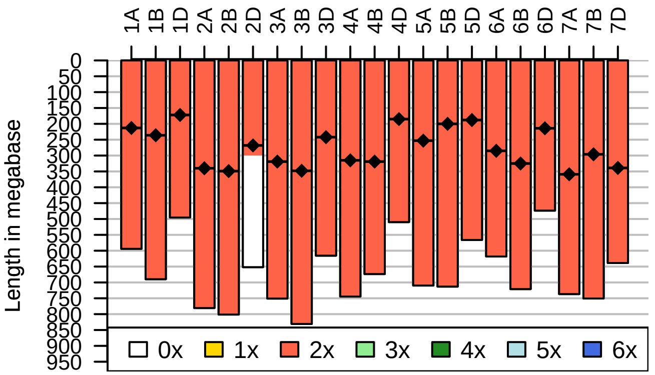

18S1−236−1 (2DL−7)

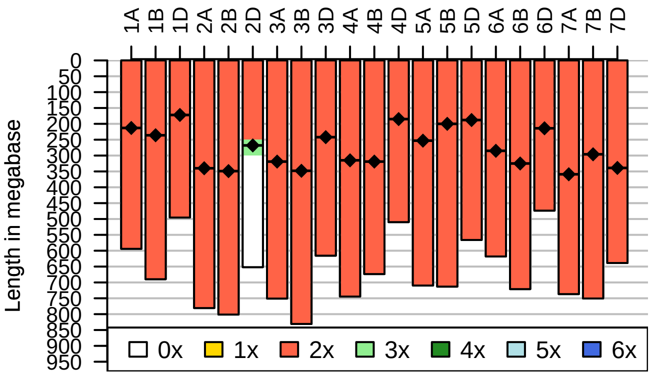

18S1−236−2 (2DL−7)

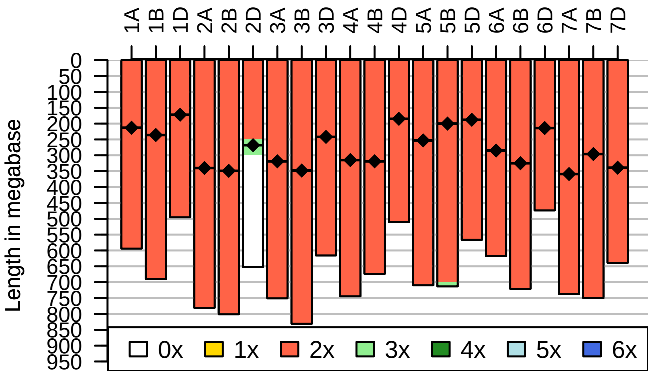

18S1−237−1 (2DL−7)

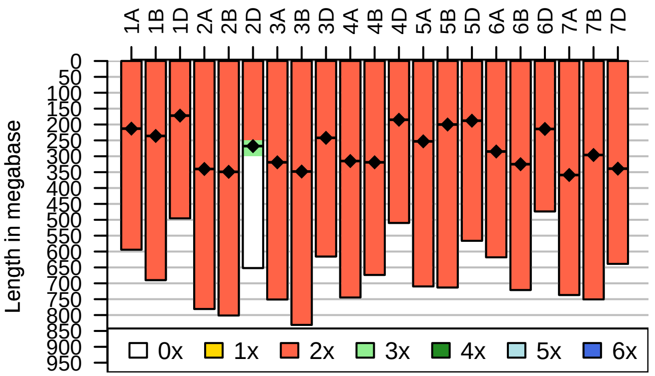

18S1−237−2 (2DL−7)

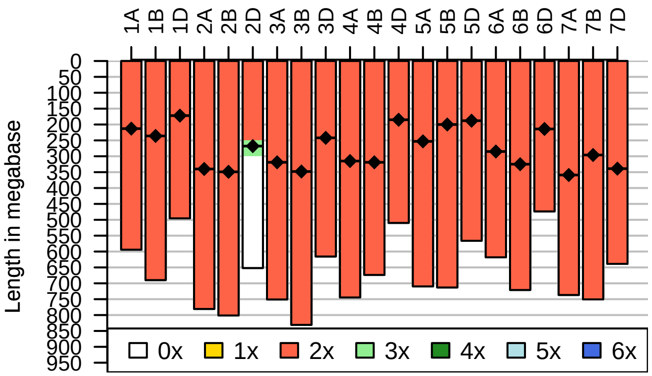

18S1−238−1 (2DL−7)

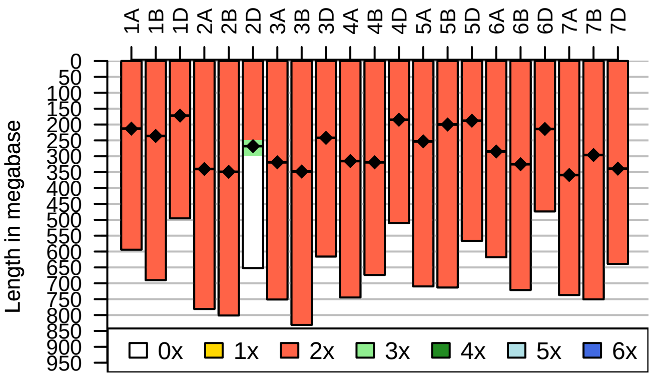

18S1−238−2 (2DL−7)

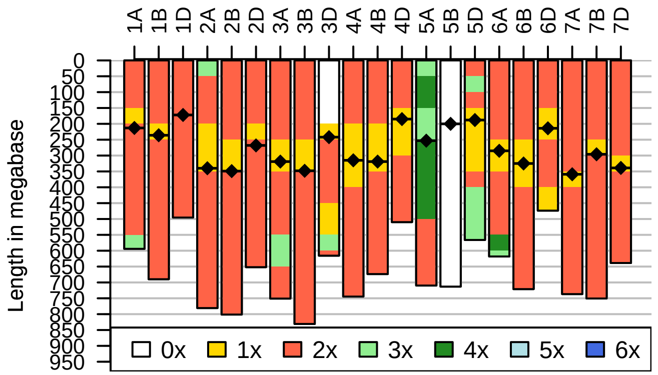

18S1−239 (3DS−2)

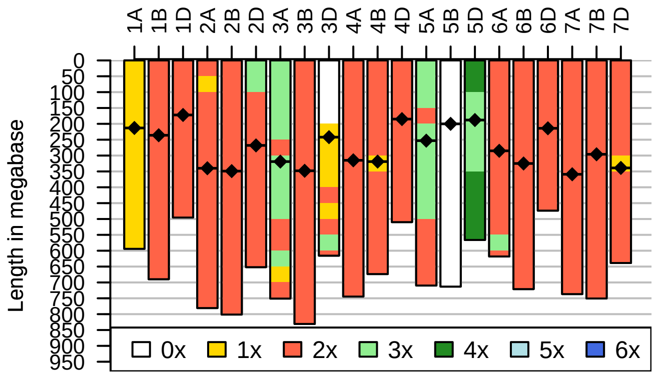

18S1−240−1 (3DS−2)

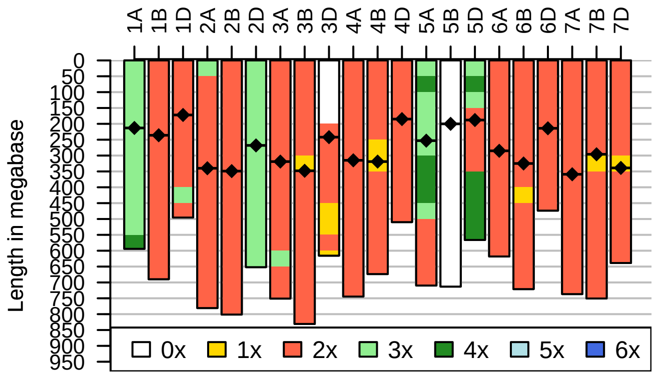

18S1−240−2 (3DS−2)

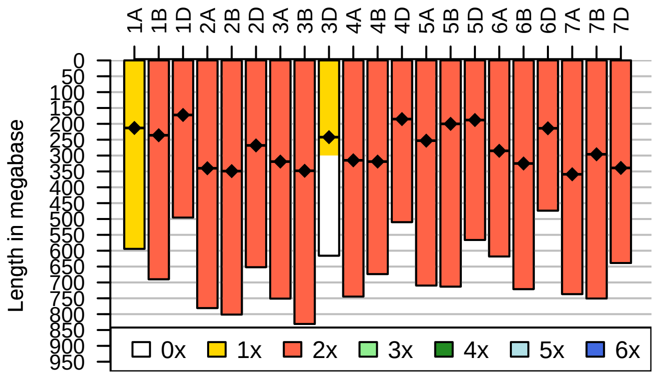

18S1−241−1 (3DL−1)

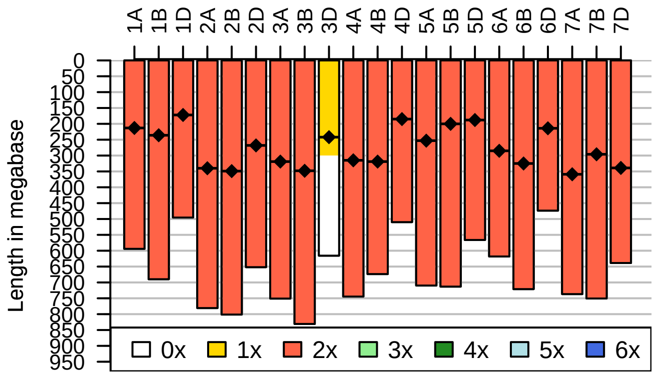

18S1−241−2 (3DL−1)

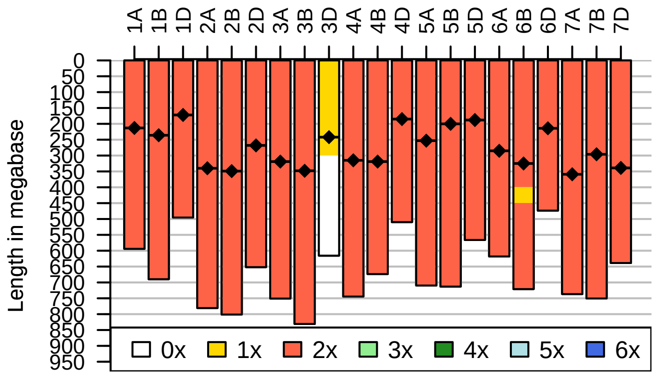

18S1−241−3 (3DL−1)

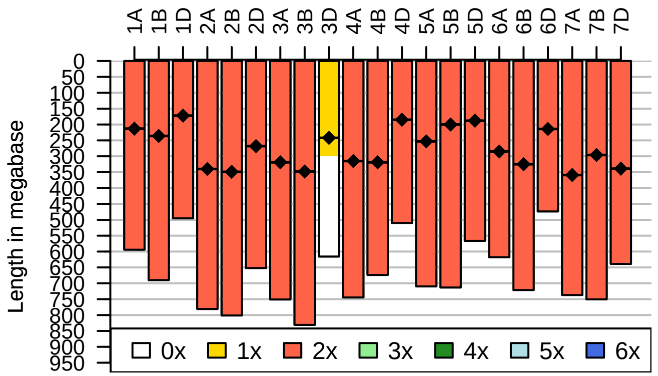

18S1−241−4 (3DL−1)

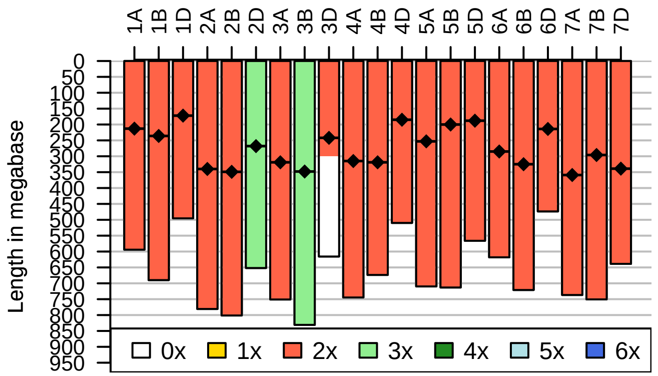

18S1−241−5 (3DL−1)

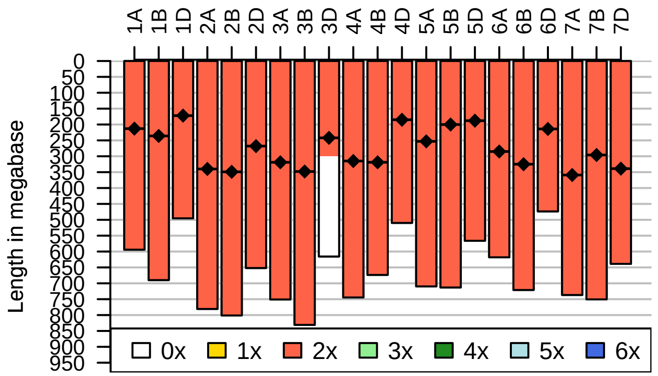

18S1−243−1 (3DL−1)

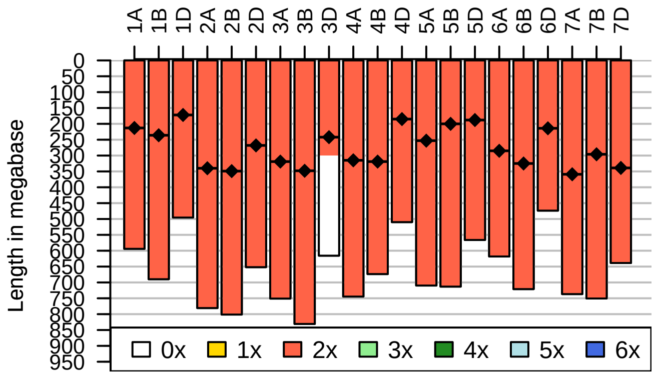

18S1−243−2 (3DL−1)

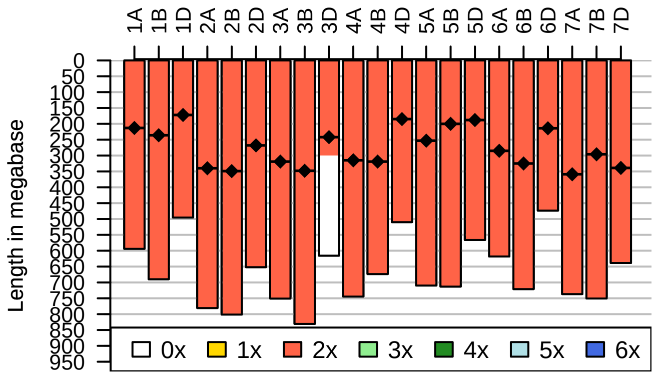

18S1−244−1 (3DL−1)

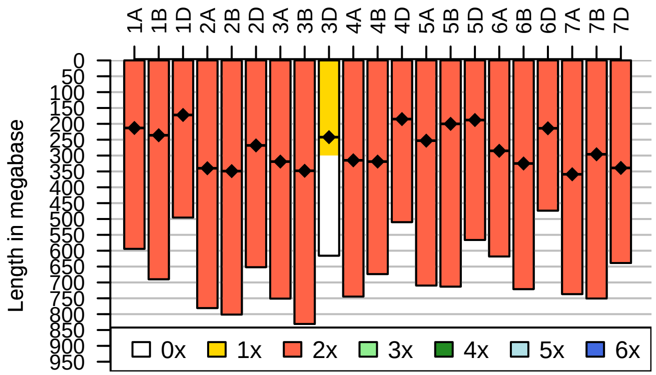

18S1−244−2 (3DL−1)

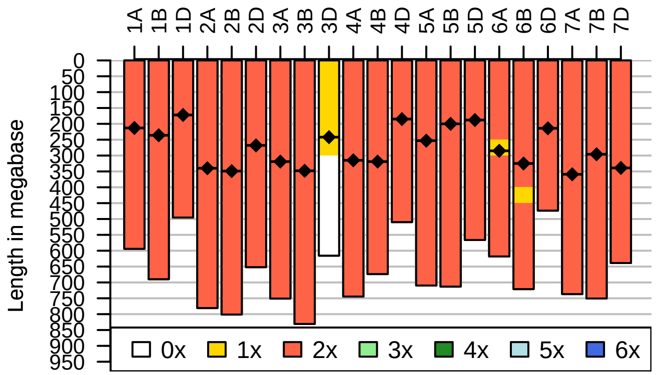

18S1−244−3 (3DL−1)

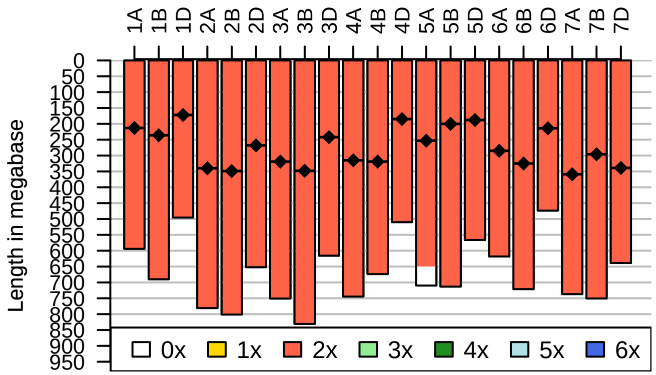

18S1−245−1 (5AL−20)

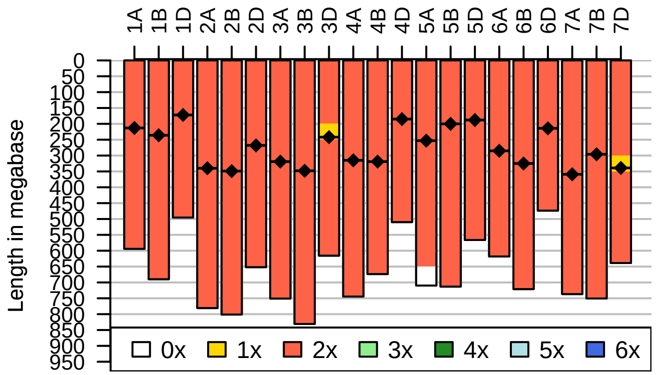

18S1−245−2 (5AL−20)

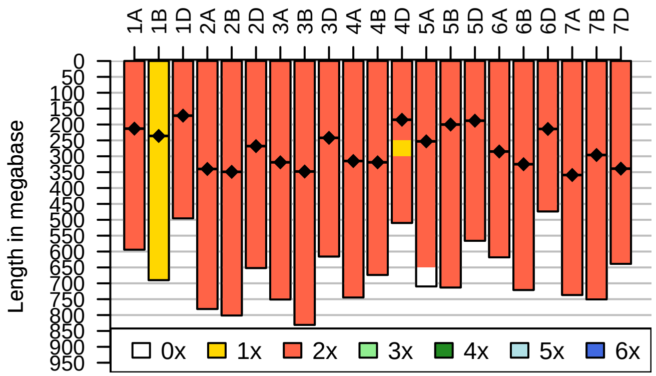

18S1−245−3 (5AL−20)

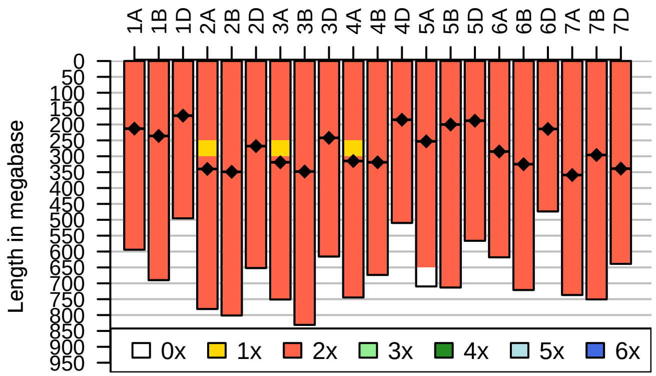

18S1−245−4 (5AL−20)

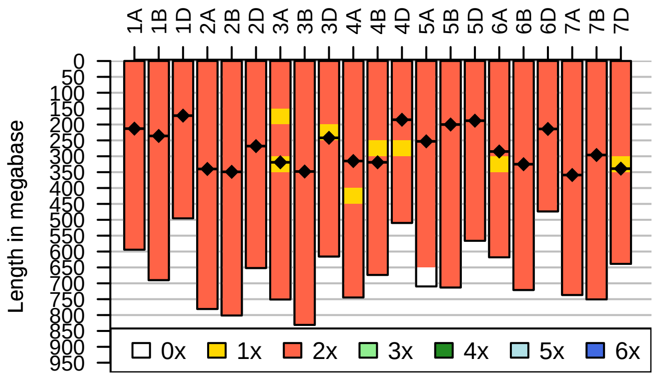

18S1−245−5 (5AL−20)

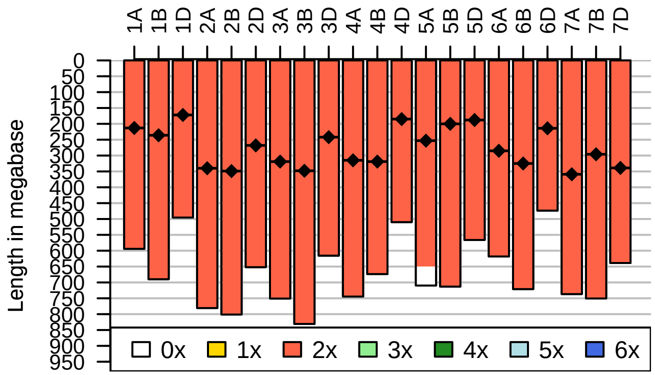

18S1−246−1 (5AL−20)

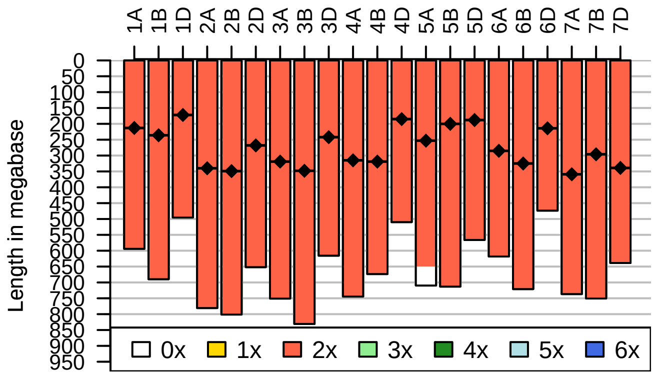

18S1−246−2 (5AL−20)

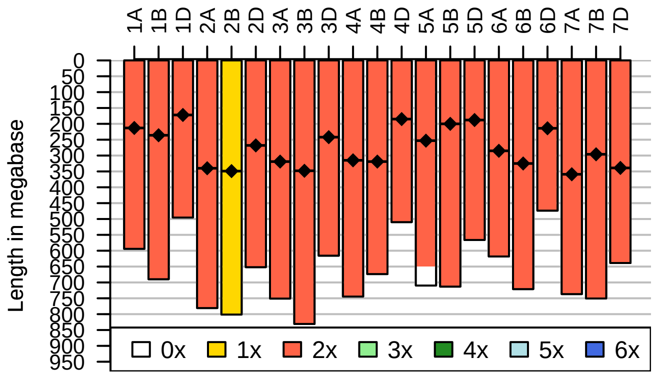

18S1−246−3 (5AL−20)

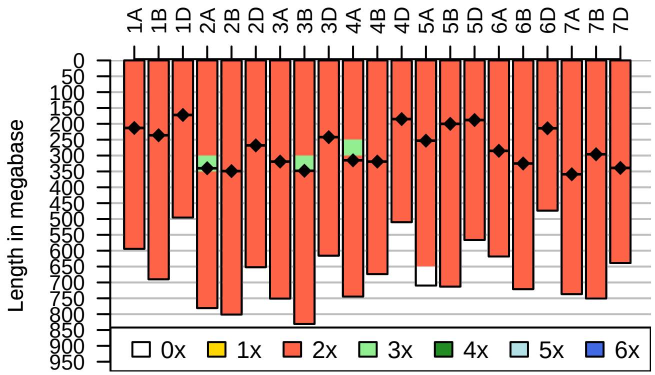

18S1−246−4 (5AL−20)

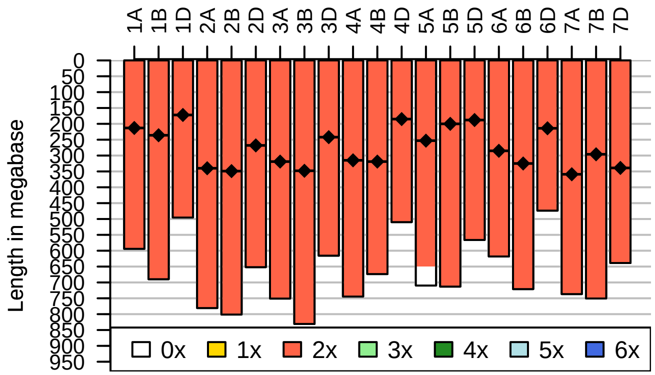

18S1−247−1 (5AL−20)

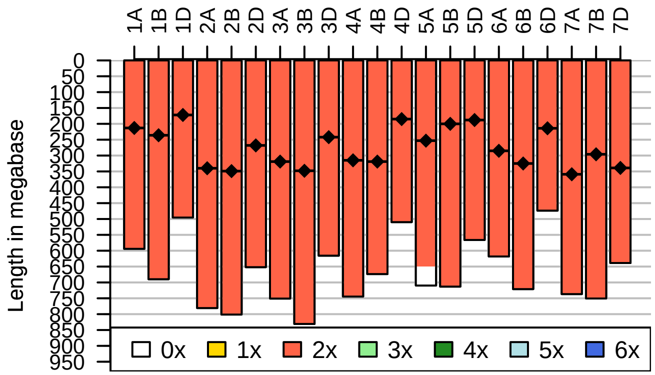

18S1−247−2 (5AL−20)

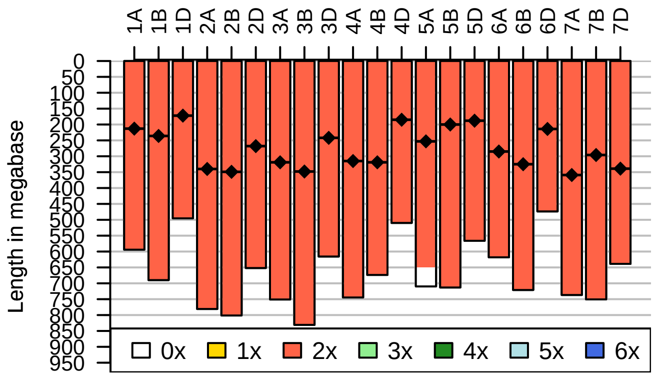

18S1−247−3 (5AL−20)

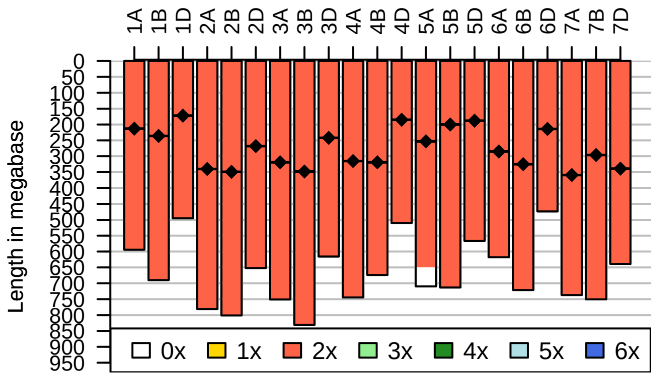

18S1−248−1 (5AL−20)

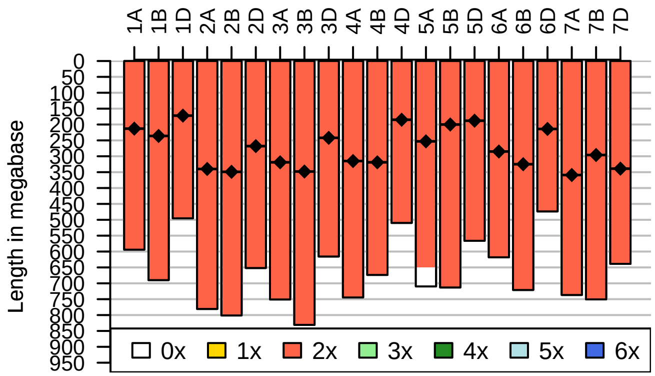

18S1−248−2 (5AL−20)

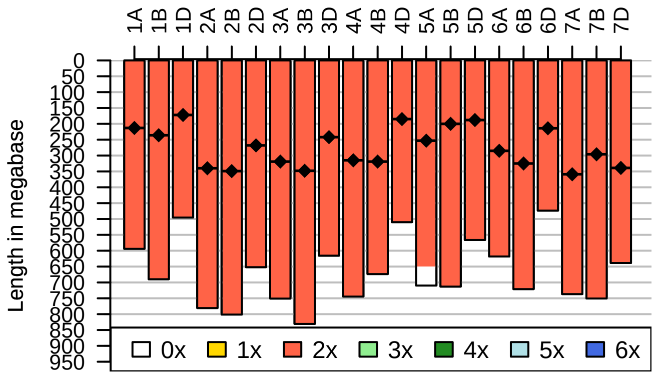

18S1−248−3 (5AL−20)

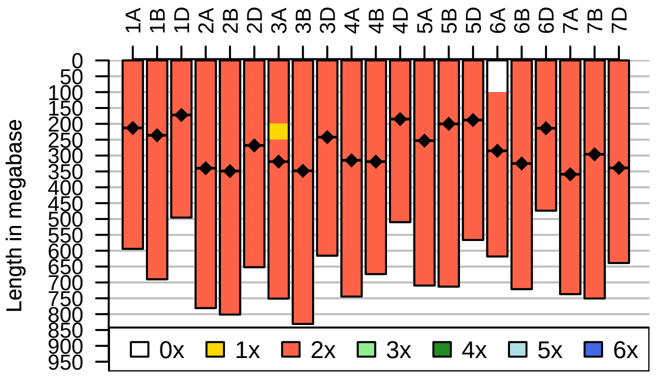

18S1−249−1 (6AS−4)

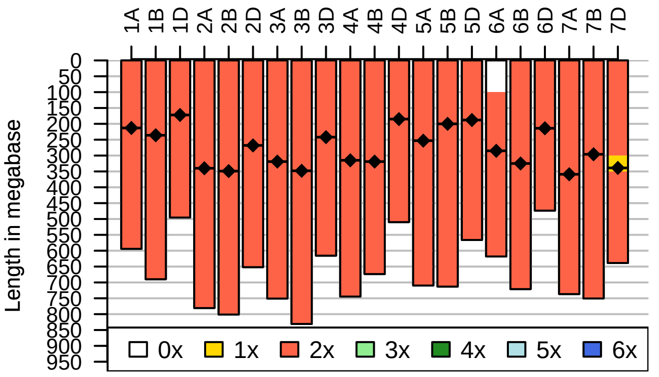

18S1−249−2 (6AS−4)

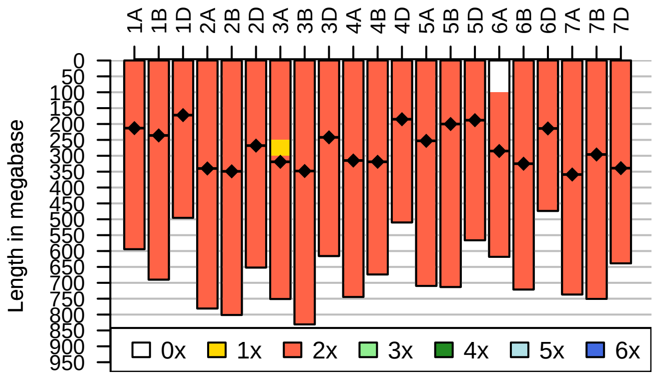

18S1−249−3 (6AS−4)

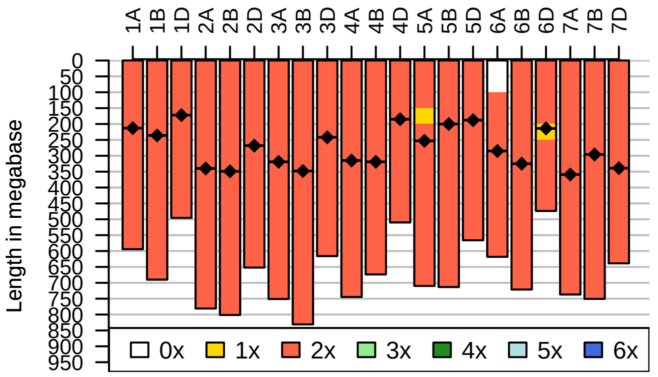

18S1−249−4 (6AS−4)

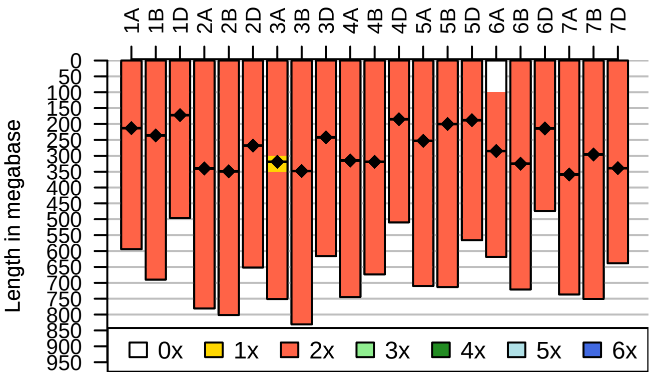

18S1−249−5 (6AS−4)

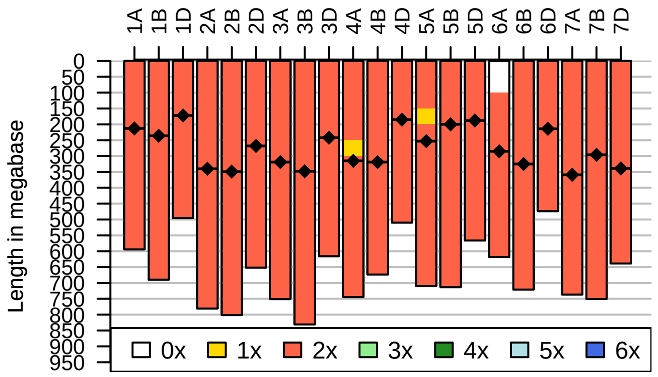

18S1−250−1 (6AS−4)

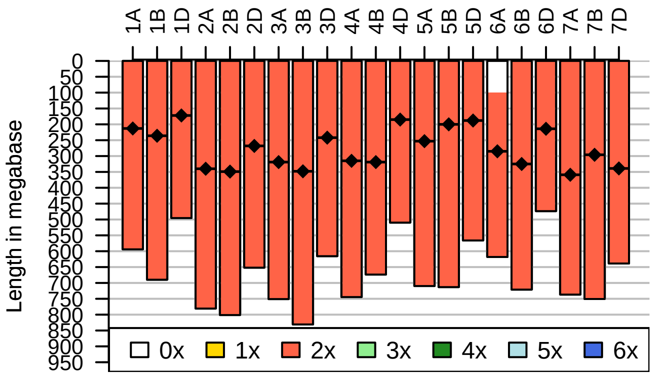

18S1−250−2 (6AS−4)

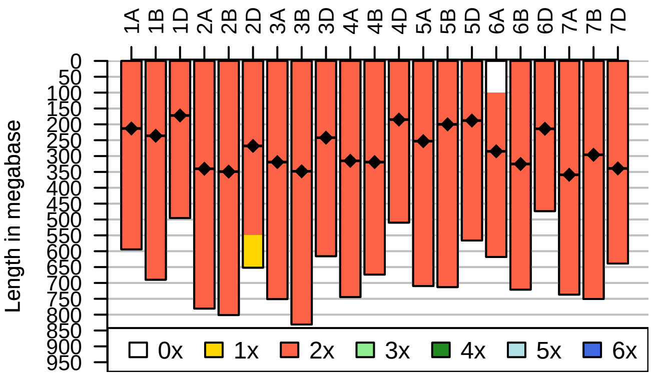

18S1−250−3 (6AS−4)

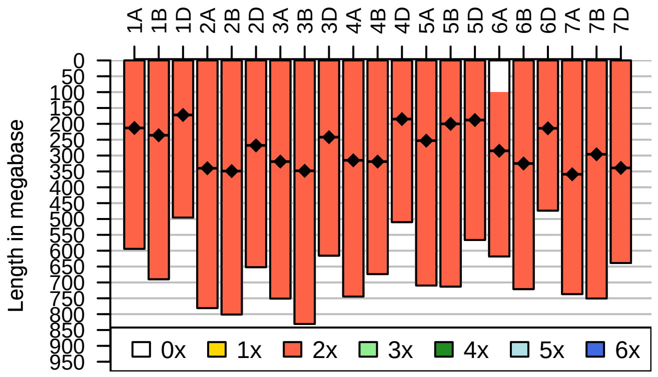

18S1−250−4 (6AS−4)

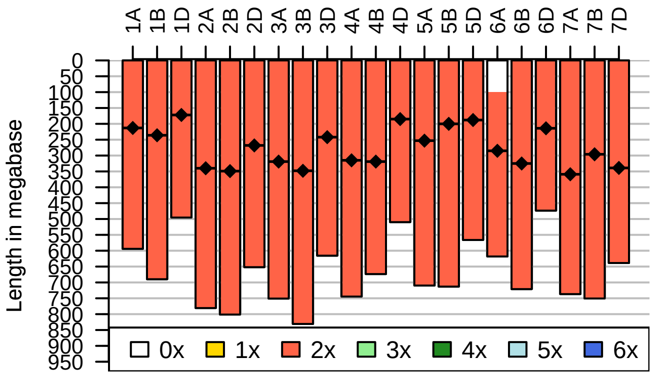

18S1−250−5 (6AS−4)

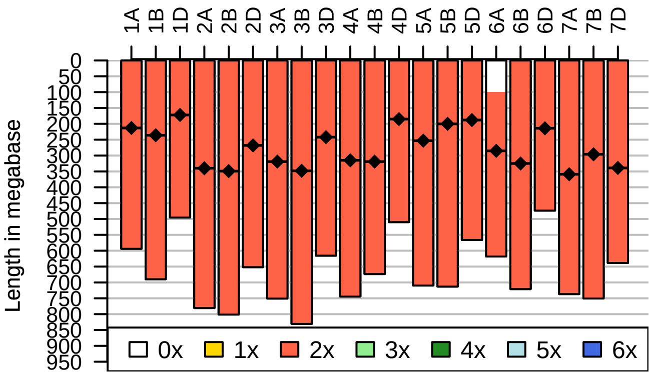

18S1−251−1 (6AS−4)

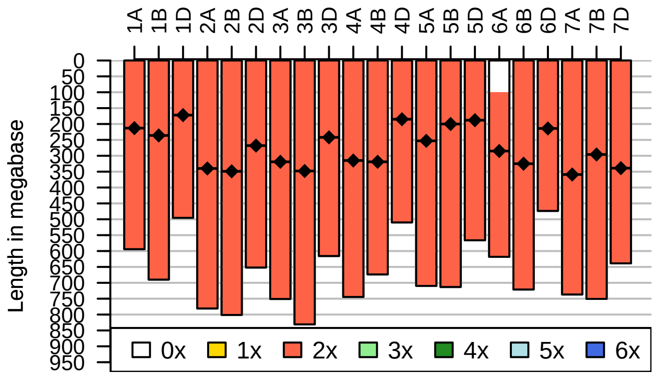

18S1−251−2 (6AS−4)

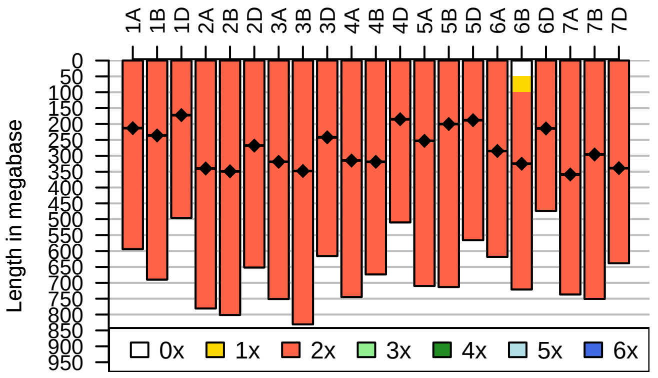

18S1−252−1 (6BS−7)

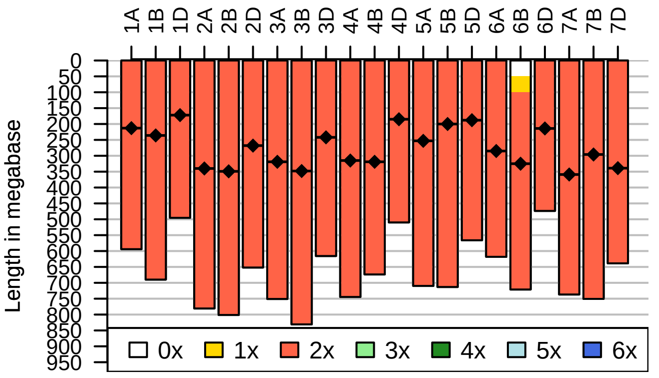

18S1−252−2 (6BS−7)

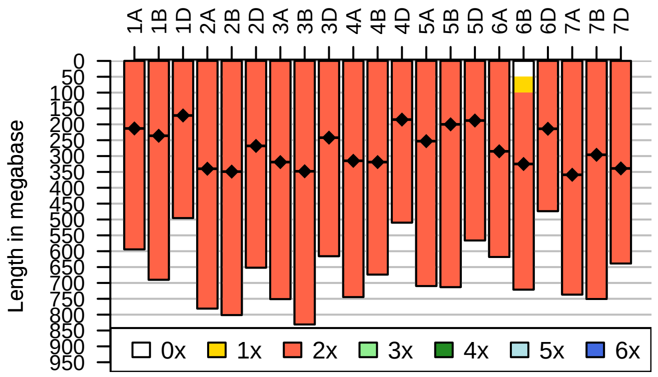

18S1−252−3 (6BS−7)

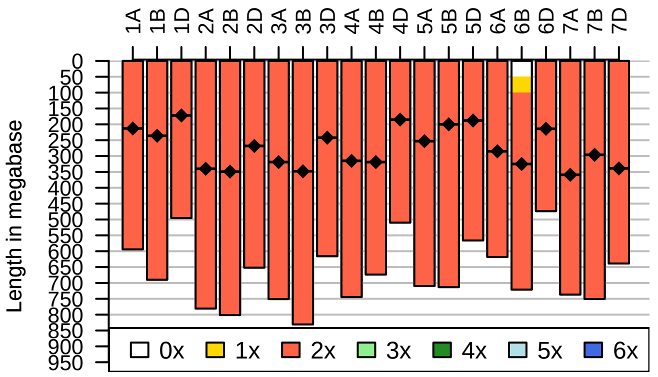

18S1−252−4 (6BS−7)

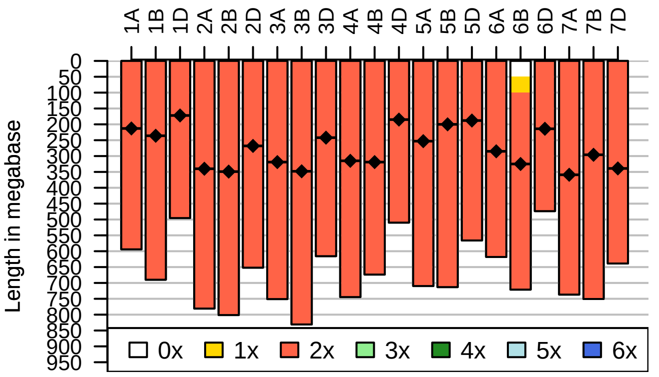

18S1−252−5 (6BS−7)

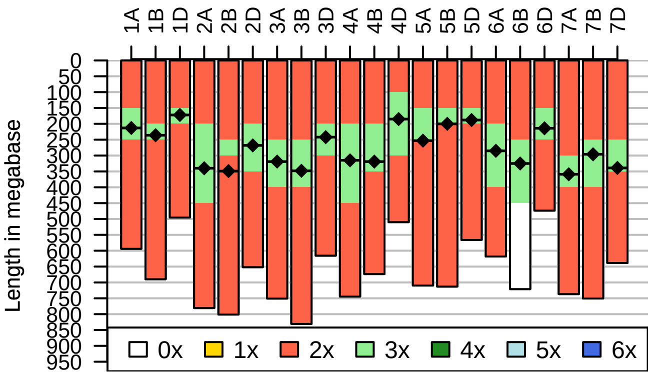

18S1−253−1 (6BL−3)

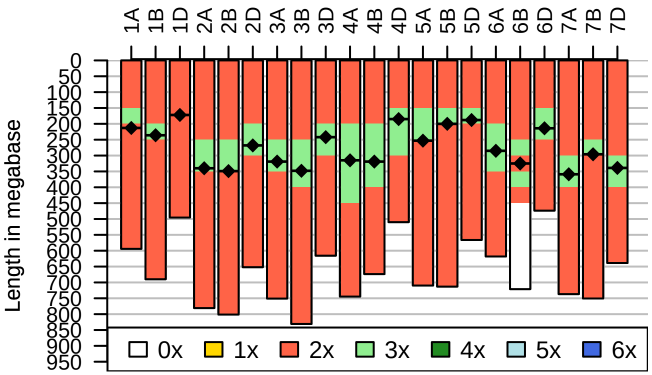

18S1−253−2 (6BL−3)

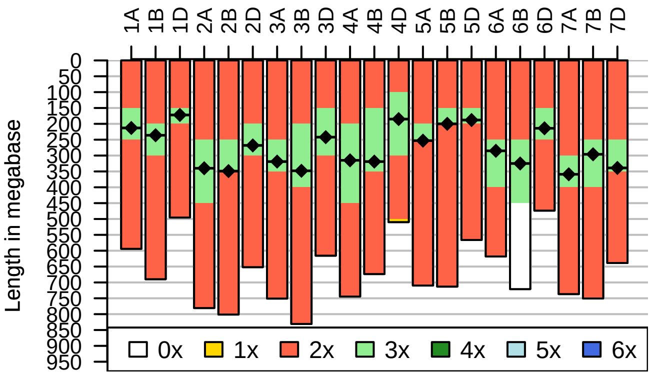

18S1−253−3 (6BL−3)

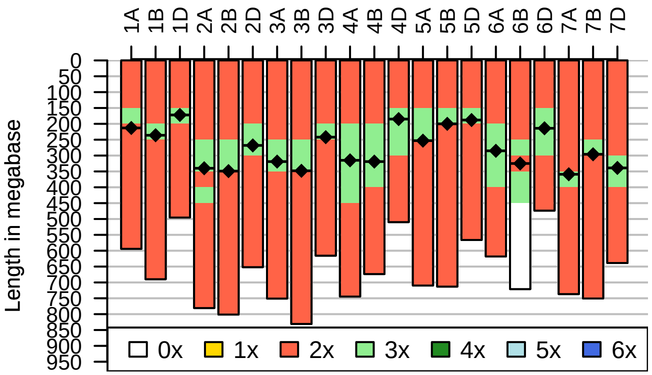

18S1−253−4 (6BL−3)

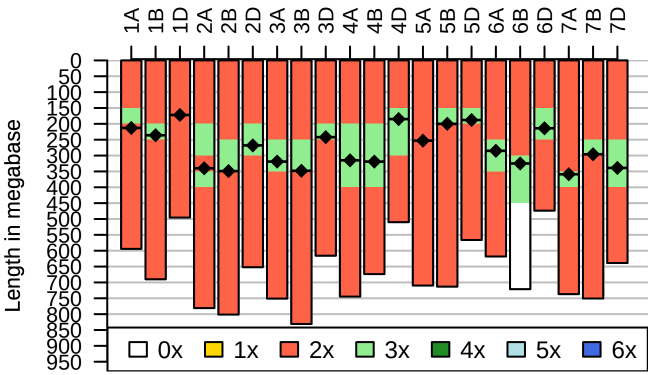

18S1−253−5 (6BL−3)

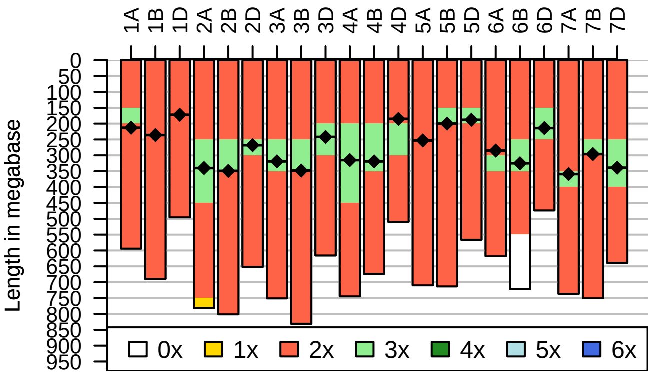

18S1−254−1 (6BL−3)

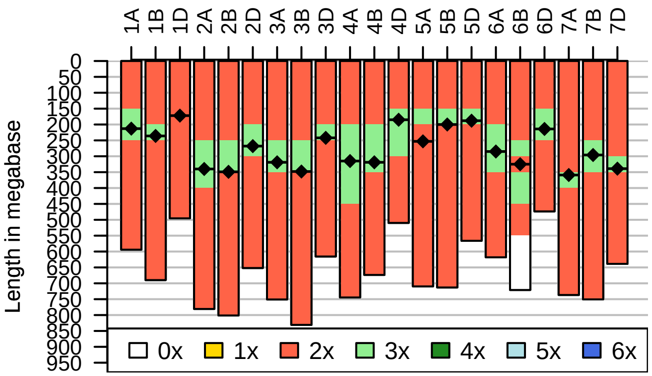

18S1−254−2 (6BL−3)

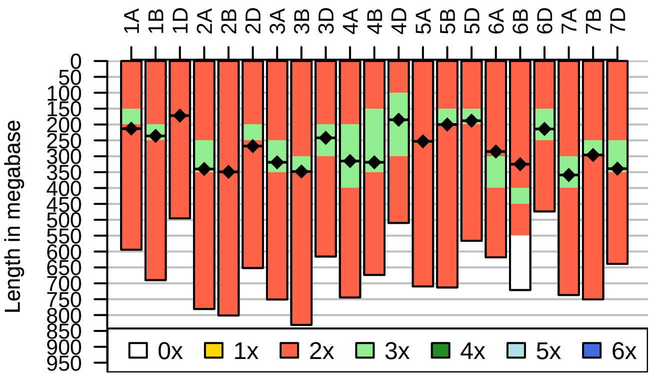

18S1−254−3 (6BL−3)

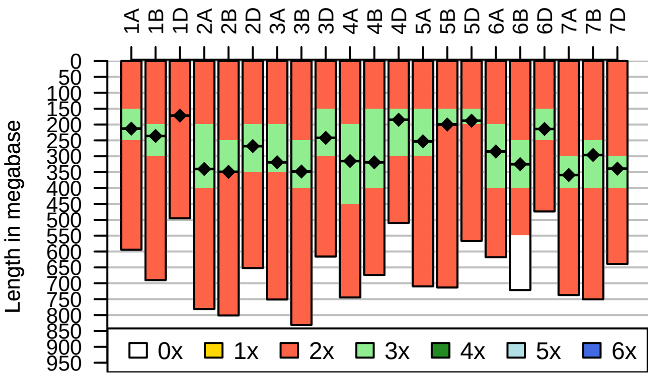

18S1−254−4 (6BL−3)

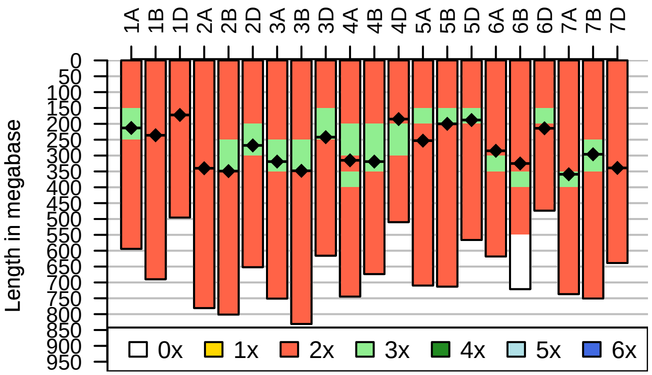

18S1−254−5 (6BL−3)

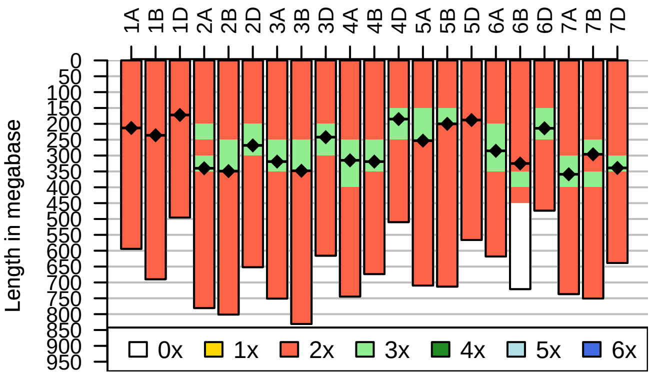

18S1−255−1 (6BL−3)

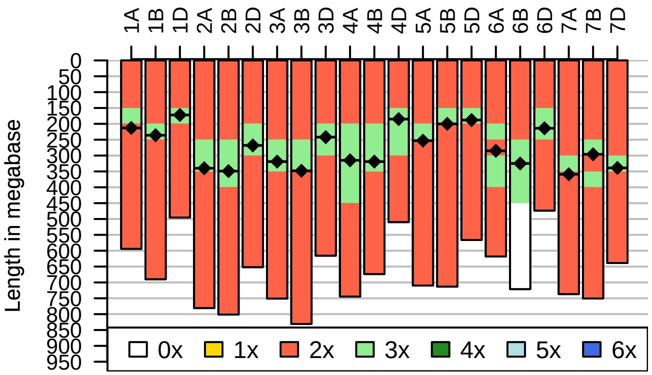

18S1−255−2 (6BL−3)

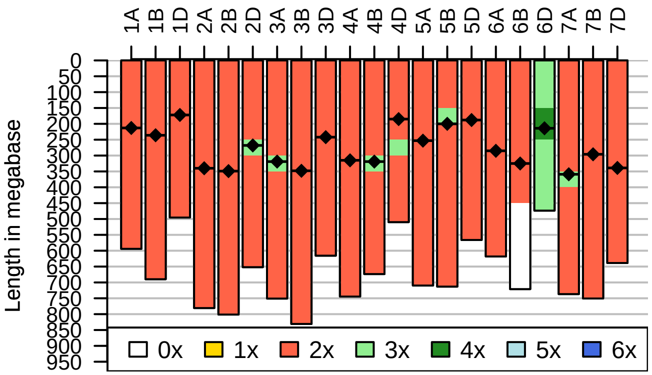

18S1−255−3 (6BL−3)

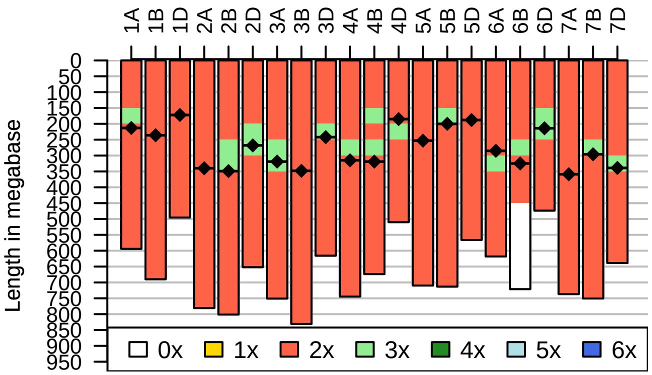

18S1−255−4 (6BL−3)

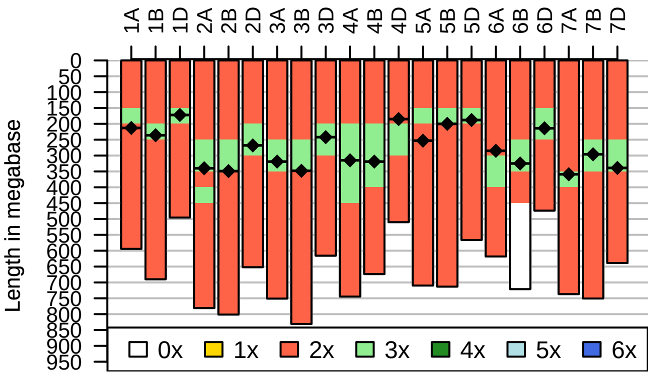

18S1−255−5 (6BL−3)

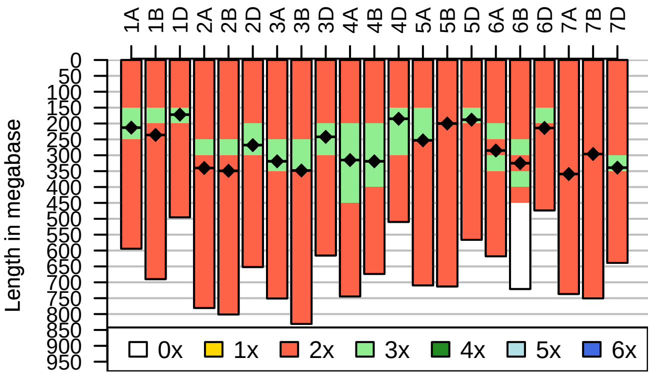

18S1−256−1 (1AS−4, 6BL−3)

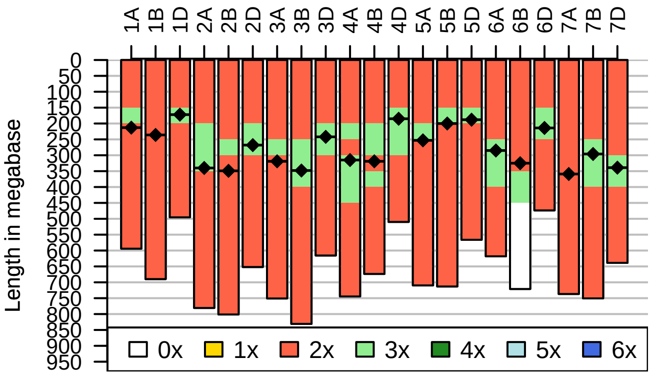

18S1−256−2 (1AS−4, 6BL−3)

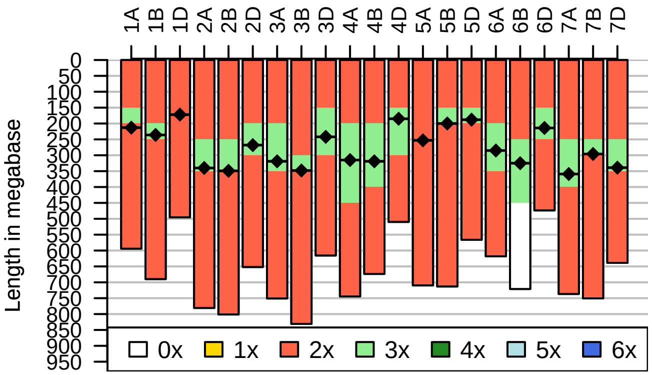

18S1−256−3 (1AS−4, 6BL−3)

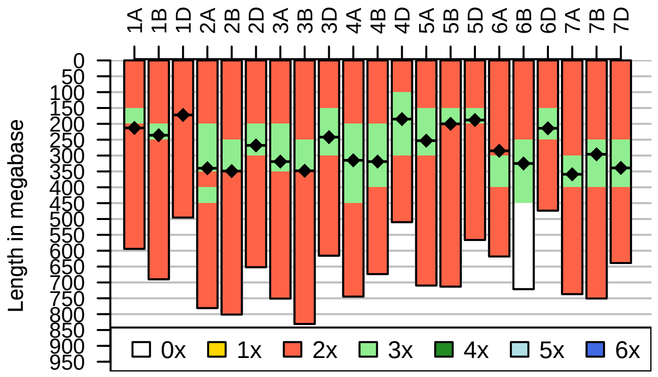

18S1−256−4 (1AS−4, 6BL−3)

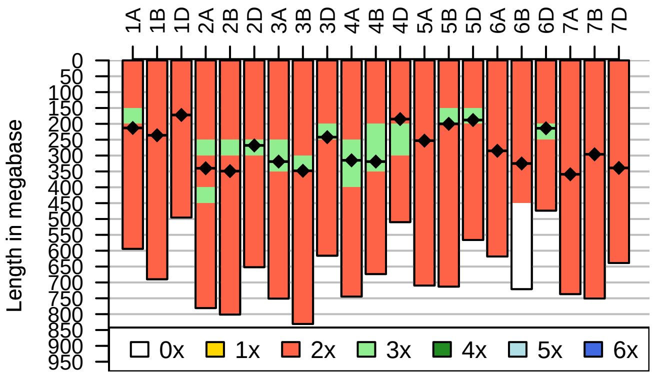

18S1−257−1 (1AS−4, 6BL−3)

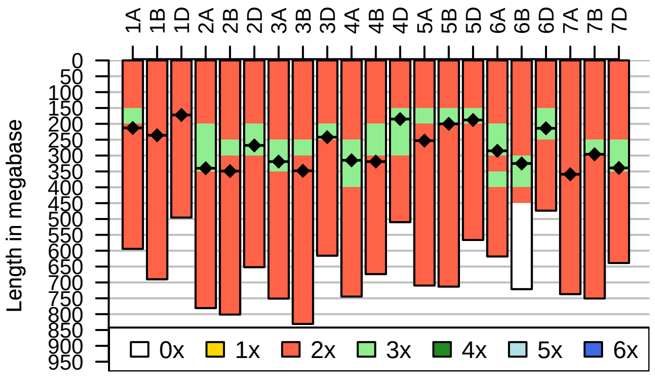

18S1−257−2 (1AS−4, 6BL−3)

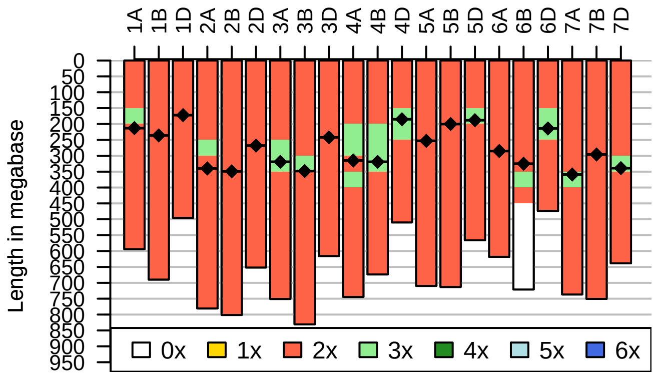

18S1−257−3 (1AS−4, 6BL−3)

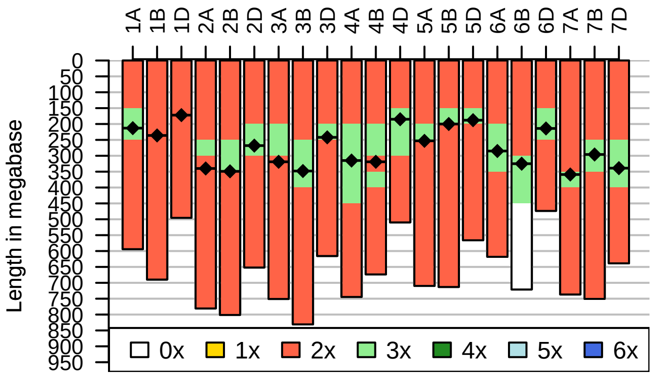

18S1−258−1 (1AS−4, 6BL−3)

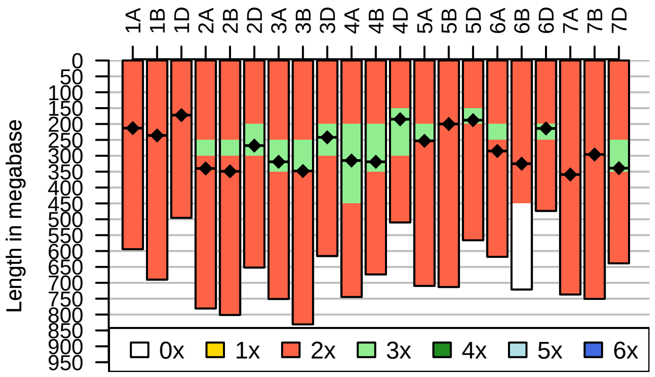

18S1−258−2 (1AS−4, 6BL−3)

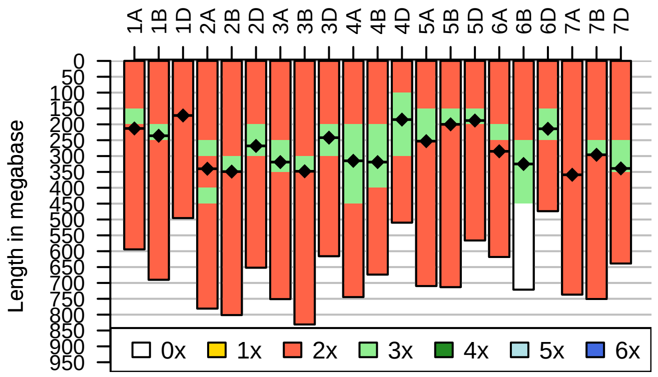

18S1−258−3 (1AS−4, 6BL−3)

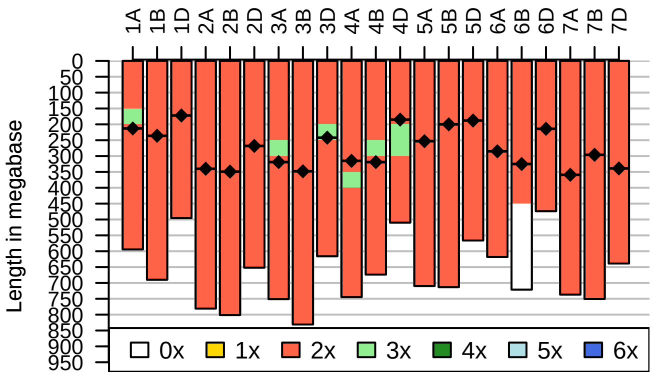

18S1−259−1 (1AS−4, 6BL−3)

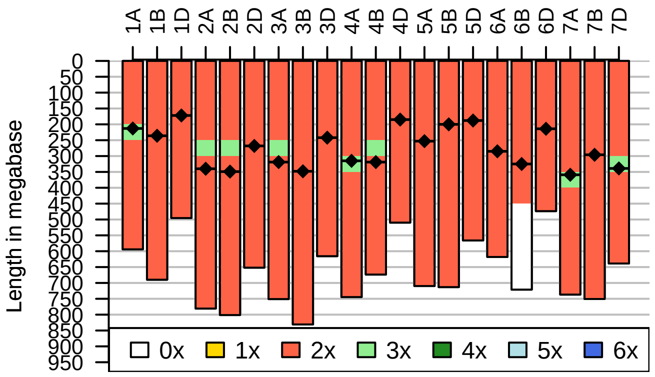

18S1−259−2 (1AS−4, 6BL−3)

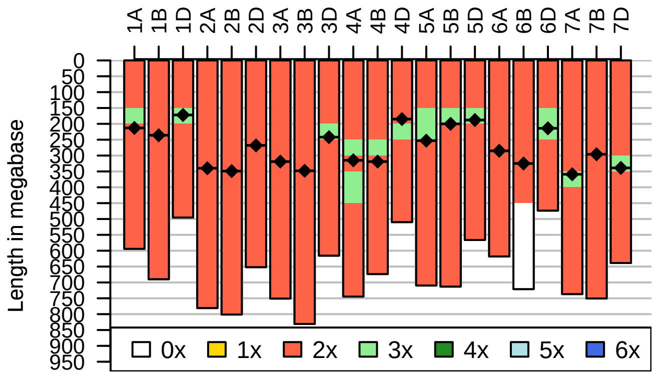

18S1−259−3 (1AS−4, 6BL−3)

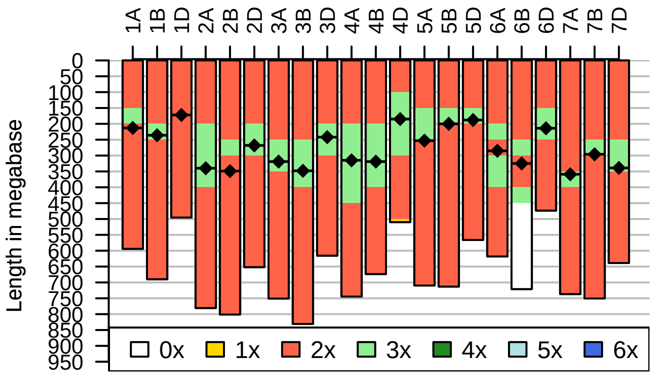

18S1−260−1 (1AS−4, 6BL−3)

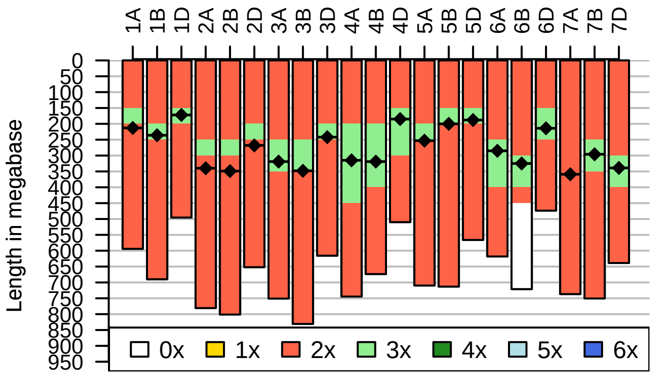

18S1−260−2 (1AS−4, 6BL−3)

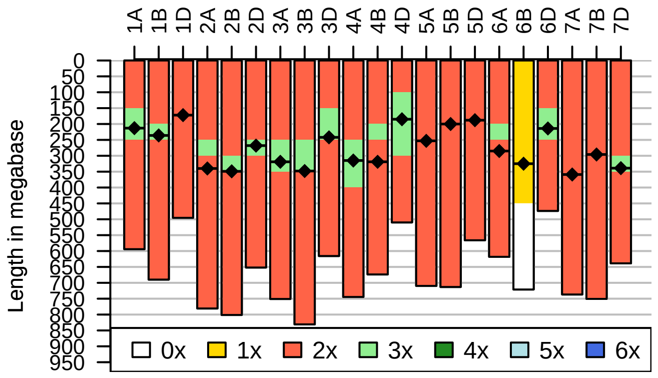

18S1−260−3 (1AS−4, 6BL−3)

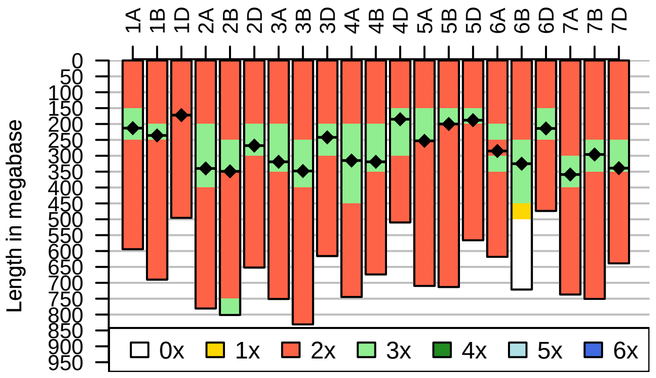

18S1−261−1 (6BL−4)

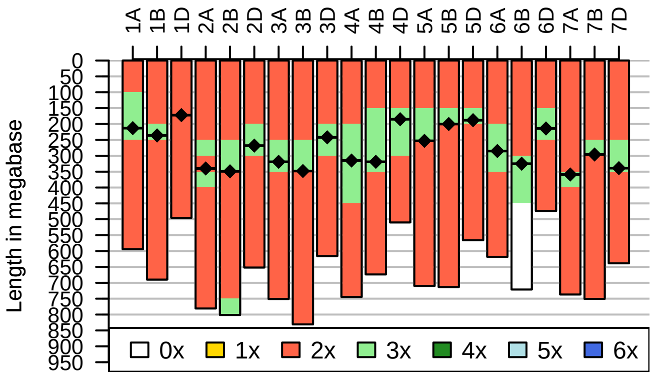

18S1−261−2 (6BL−4)

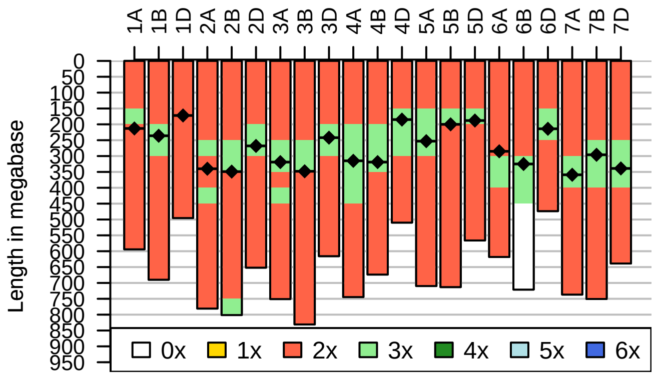

18S1−261−3 (6BL−4)

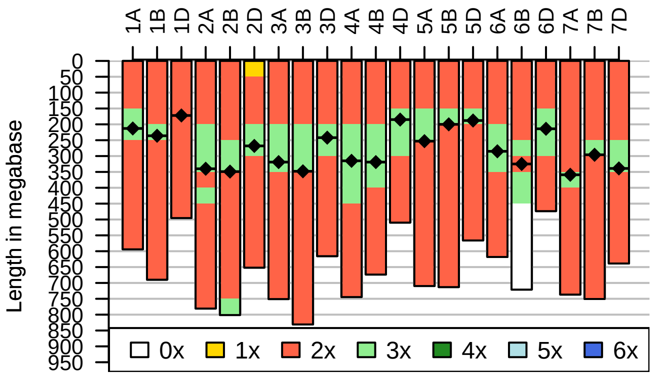

18S1−261−4 (6BL−4)

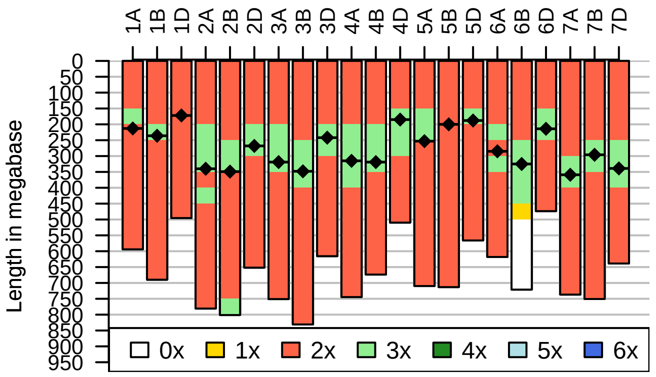

18S1−261−5 (6BL−4)

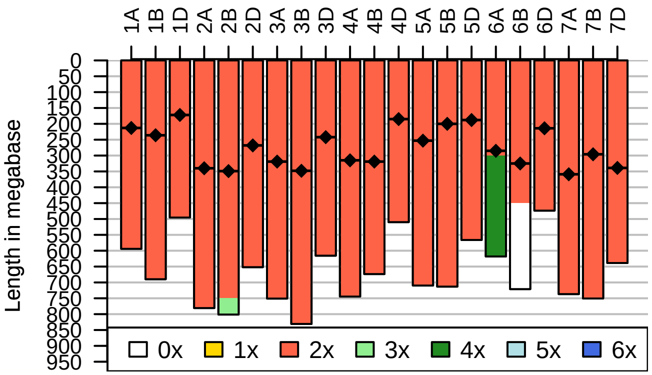

18S1−262−1 (6BL−4)

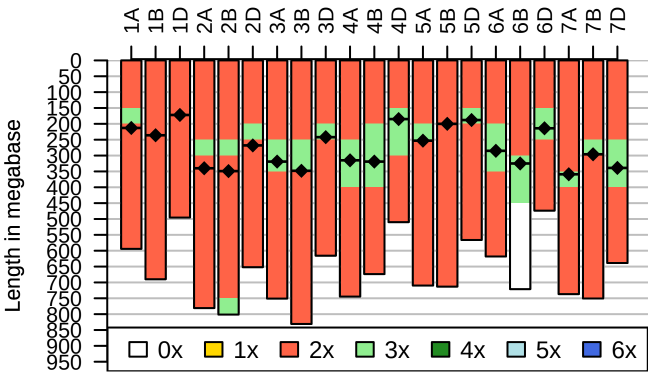

18S1−262−2 (6BL−4)

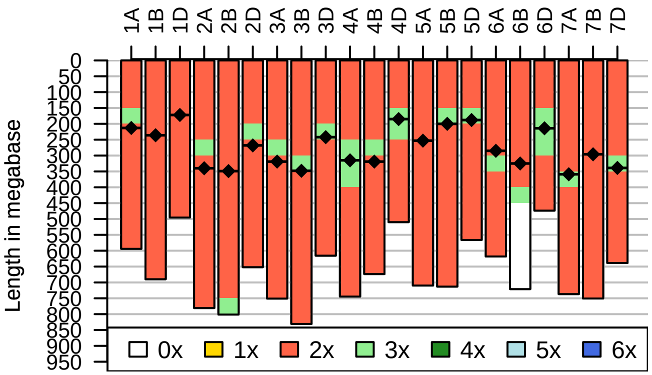

18S1−262−3 (6BL−4)

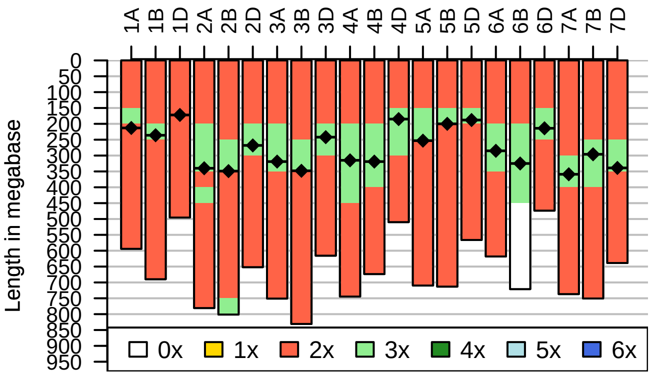

18S1−263−1 (6BL−4)

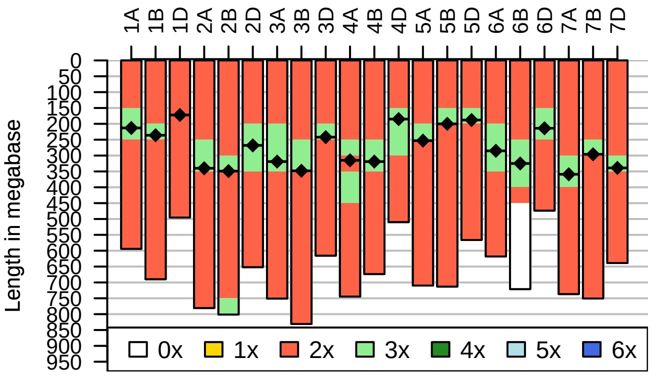

18S1−263−2 (6BL−4)

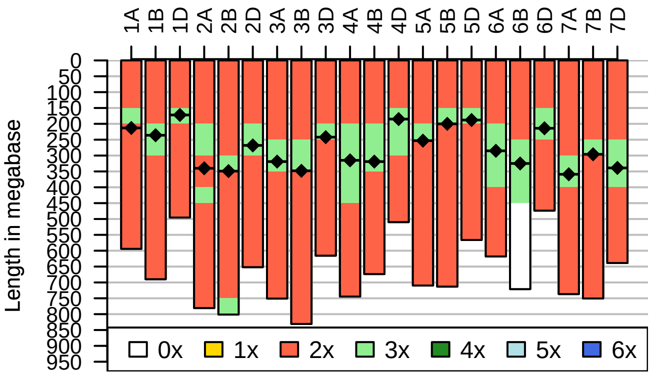

18S1−263−3 (6BL−4)

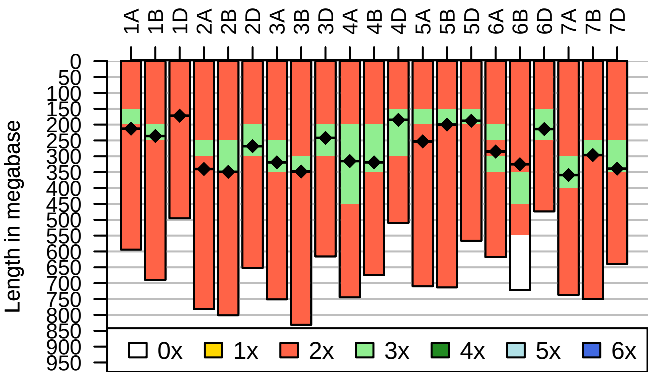

18S1−264−1 (6BL−8)

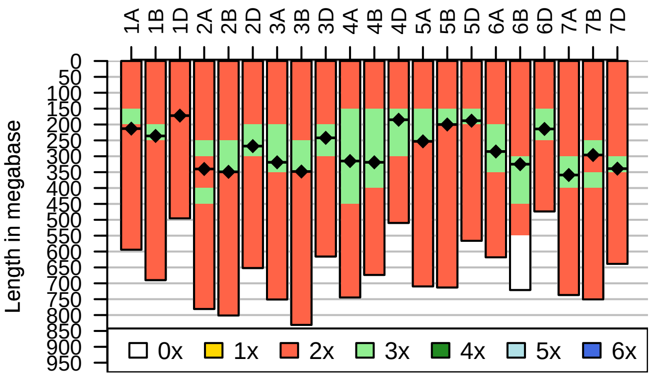

18S1−264−2 (6BL−8)

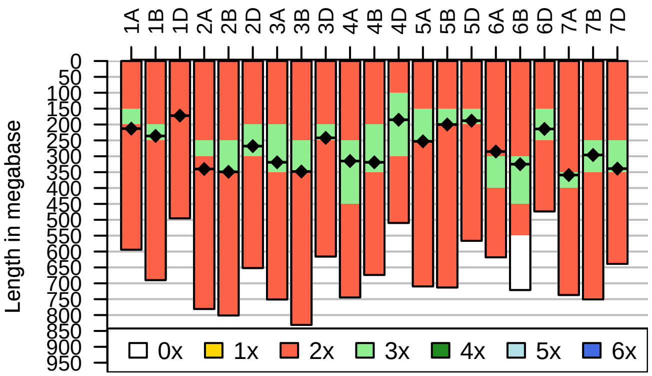

18S1−264−3 (6BL−8)

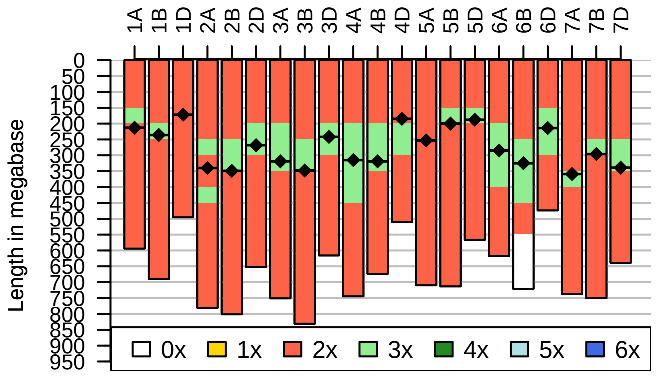

18S1−264−4 (6BL−8)

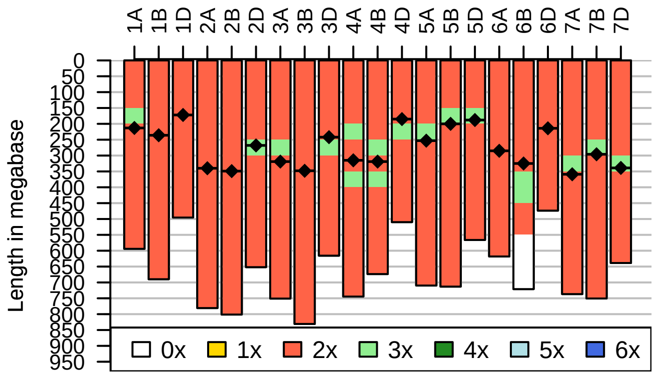

18S1−264−5 (6BL−8)

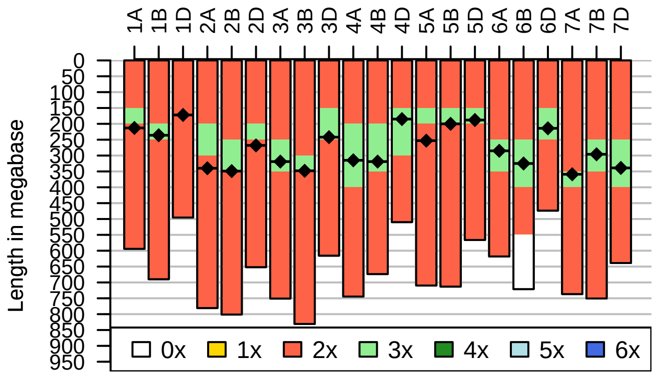

18S1−265−1 (6BL−8)

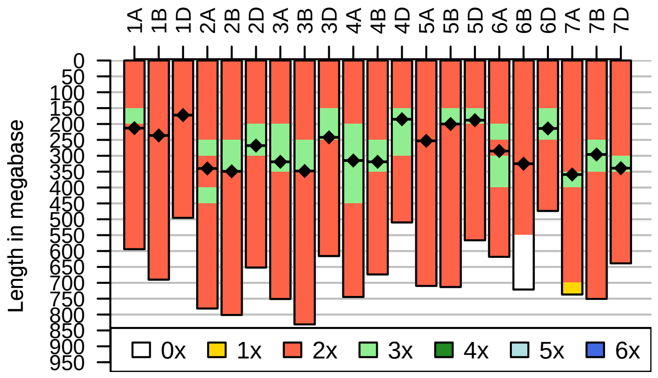

18S1−265−2 (6BL−8)

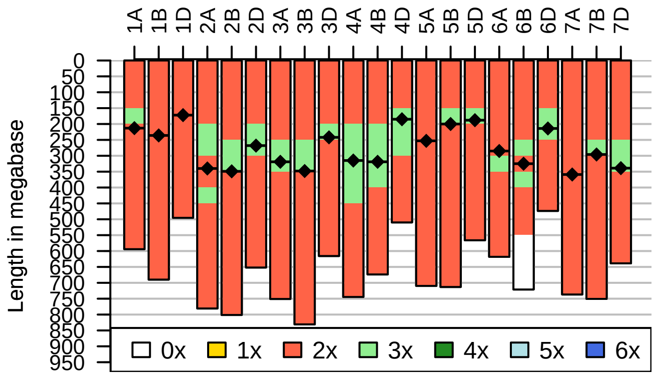

18S1−265−3 (6BL−8)

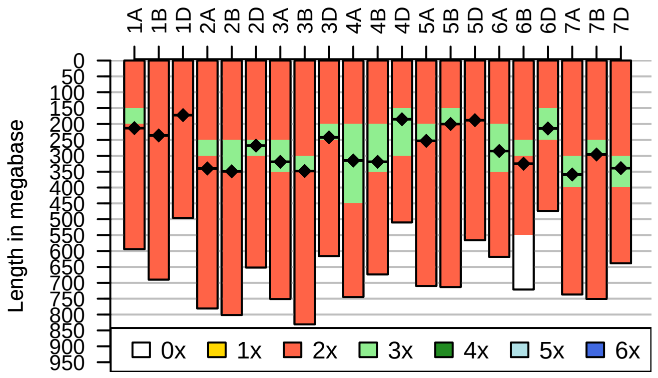

18S1−265−4 (6BL−8)

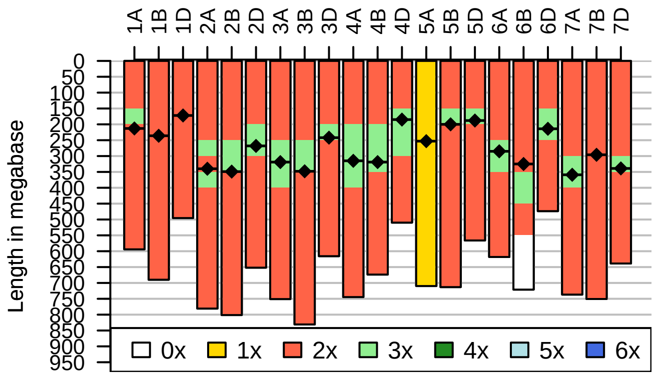

18S1−266−1 (6BL−9)

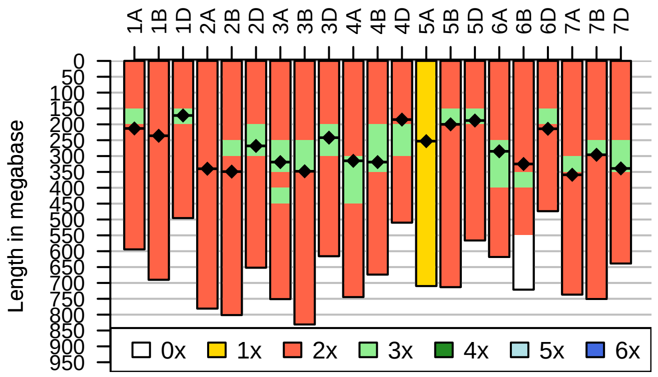

18S1−266−2 (6BL−9)

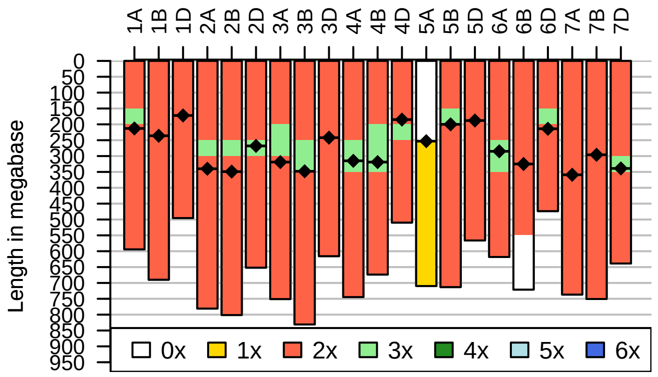

18S1−266−3 (6BL−9)

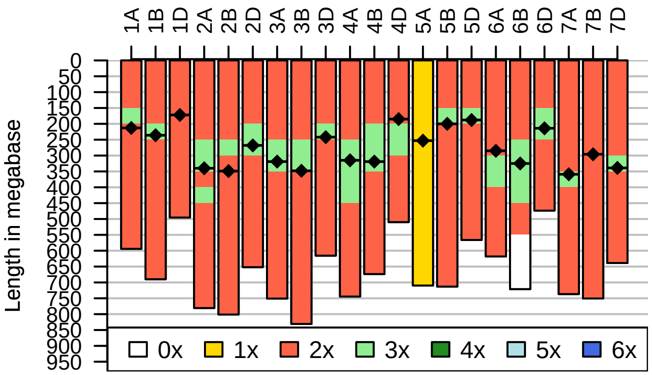

18S1−266−4 (6BL−9)

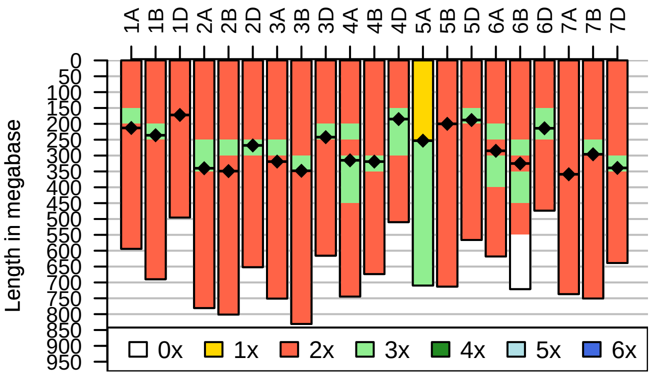

18S1−266−5 (6BL−9)

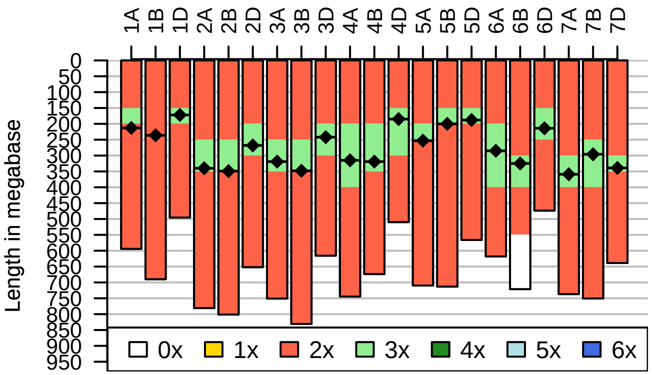

18S1−267−1 (6BL−9)

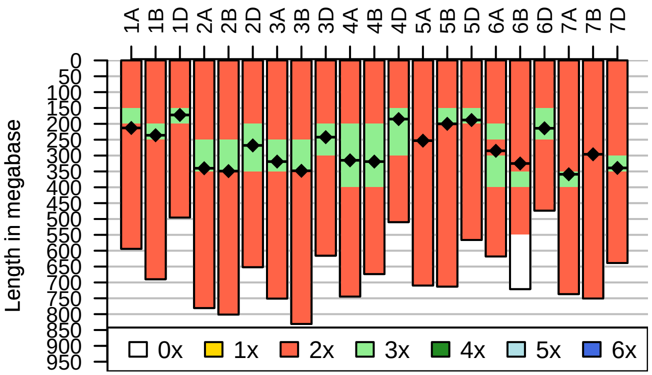

18S1−267−2 (6BL−9)

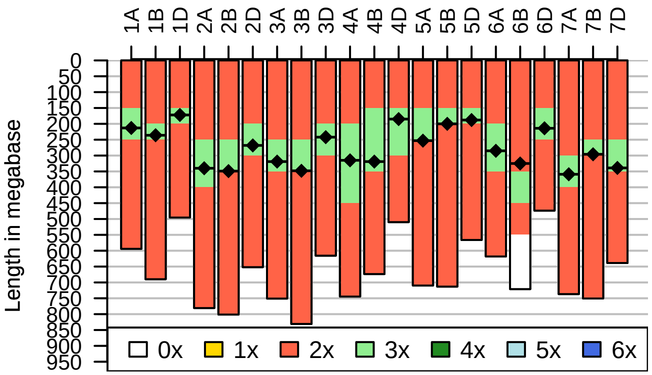

18S1−267−3 (6BL−9)

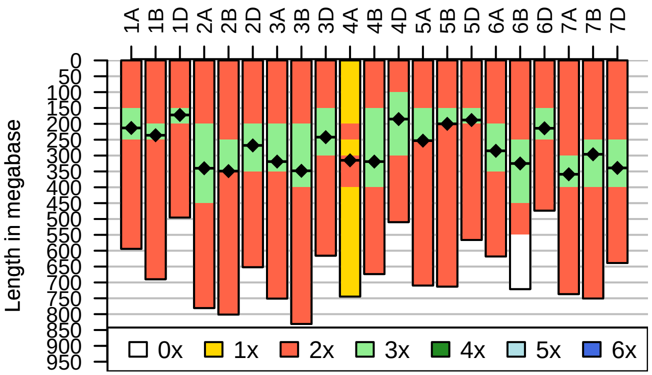

18S1−267−4 (6BL−9)

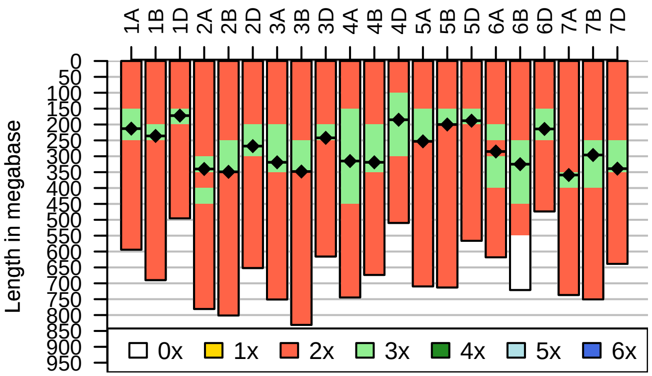

18S1−267−5 (6BL−9)

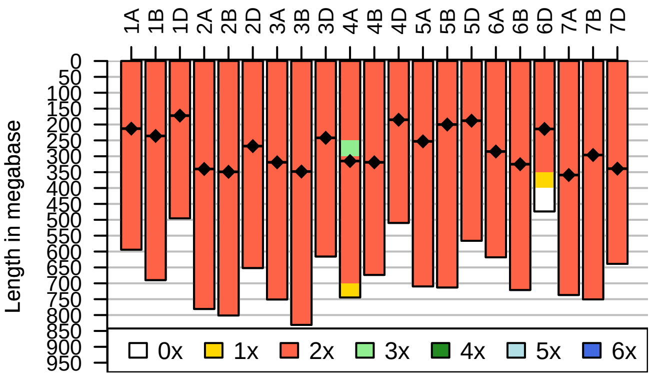

18S1−268−1 (6DL−1)

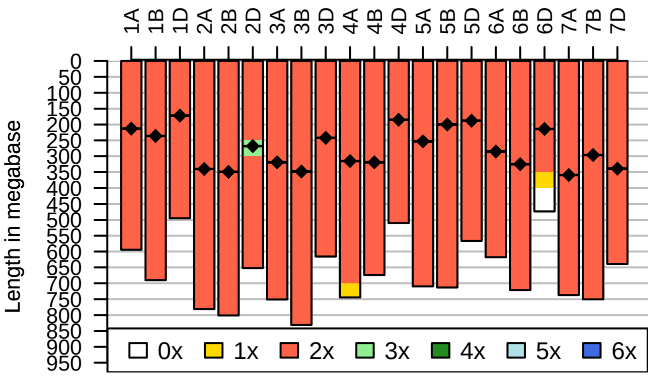

18S1−268−2 (6DL−1)

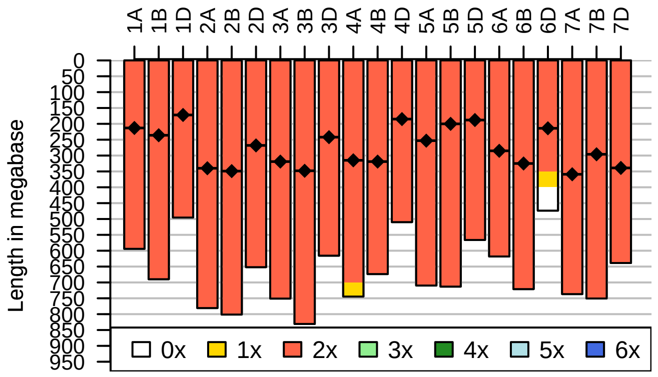

18S1−268−3 (6DL−1)

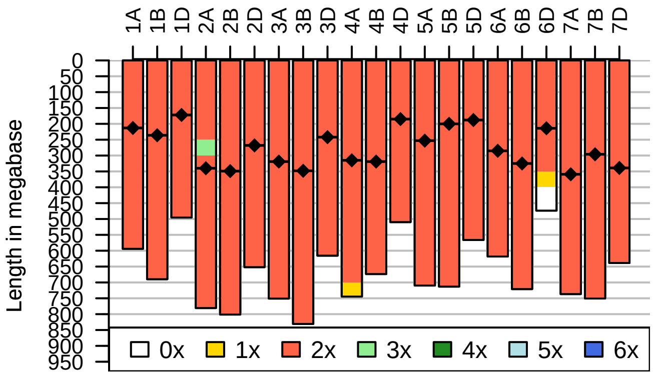

18S1−268−4 (6DL−1)

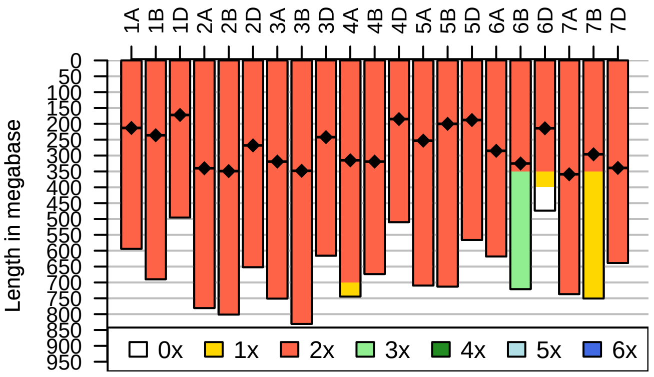

18S1−268−5 (6DL−1)

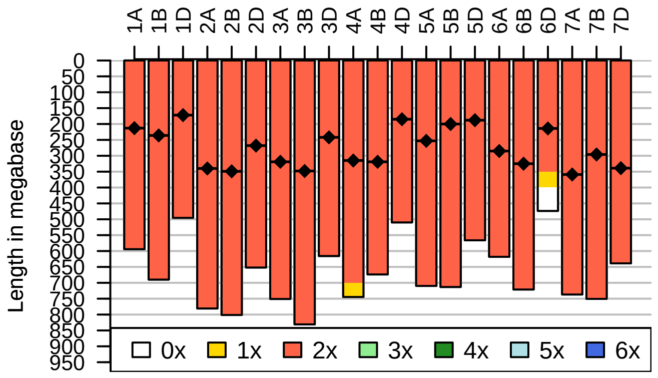

18S1−269−1 (6DL−1)

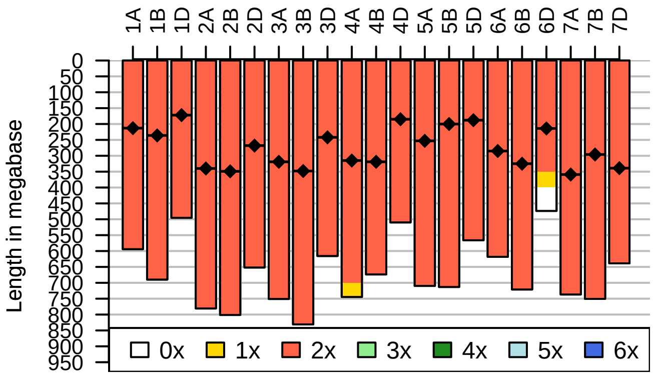

18S1−269−2 (6DL−1)

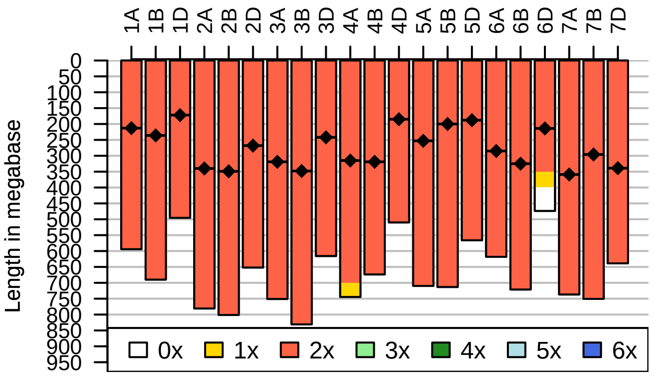

18S1−269−3 (6DL−1)

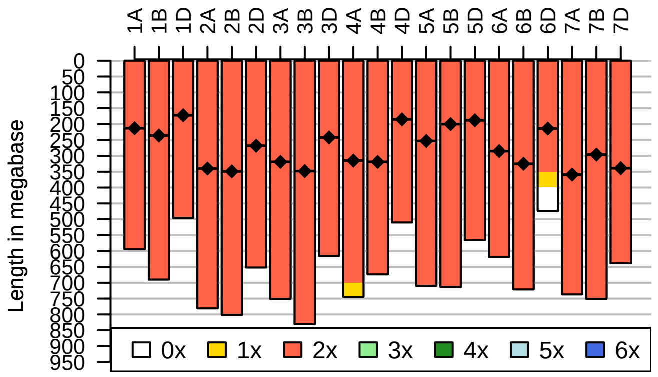

18S1−269−4 (6DL−1)

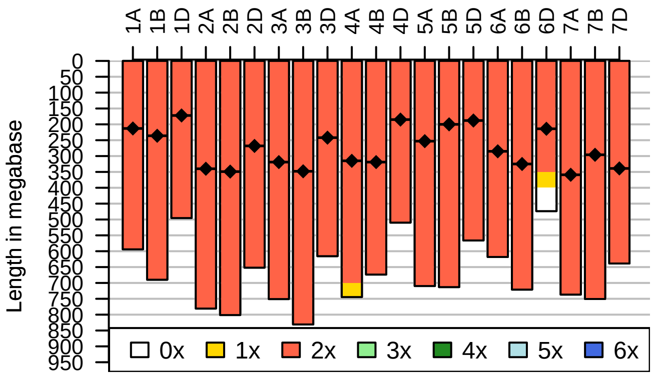

18S1−270−1 (6DL−1)

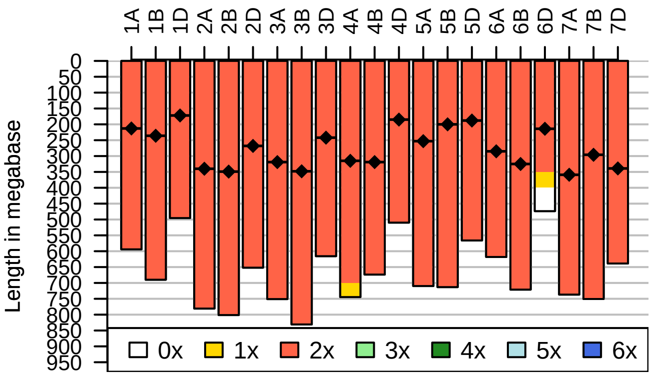

18S1−270−2 (6DL−1)

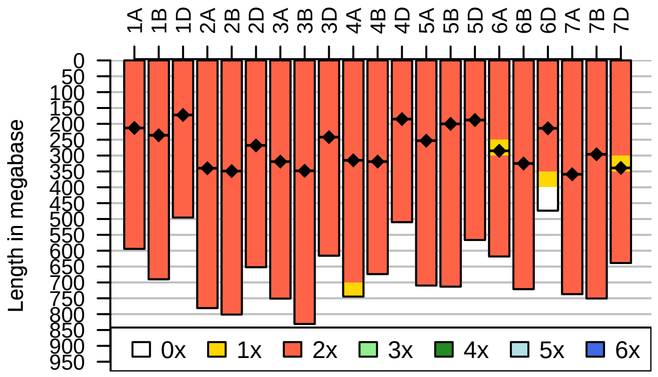

18S1−270−3 (6DL−1)

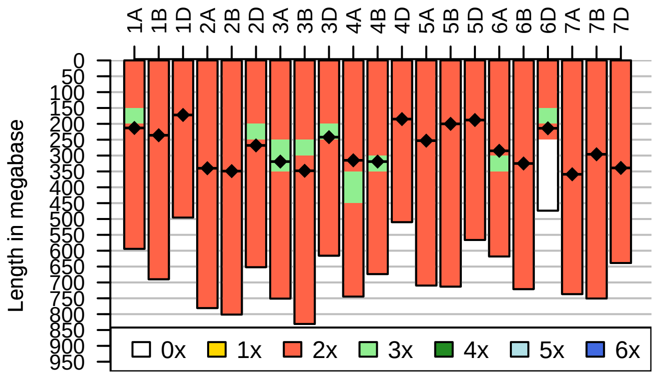

18S1−271−1 (6DL−3)

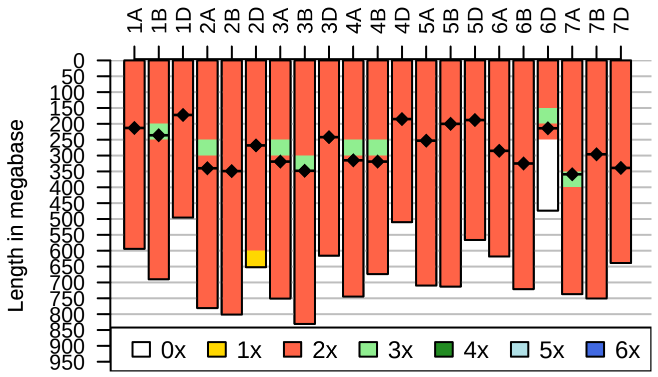

18S1−271−2 (6DL−3)

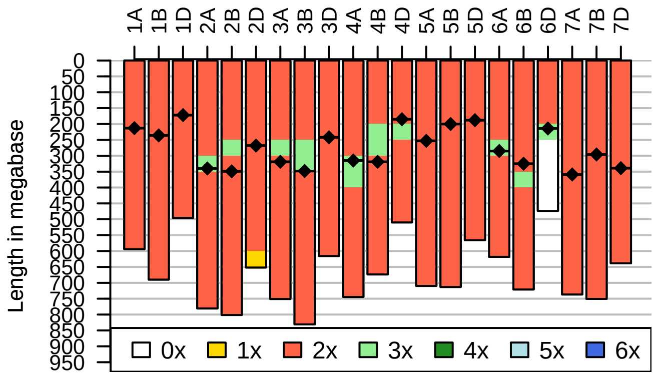

18S1−271−3 (6DL−3)

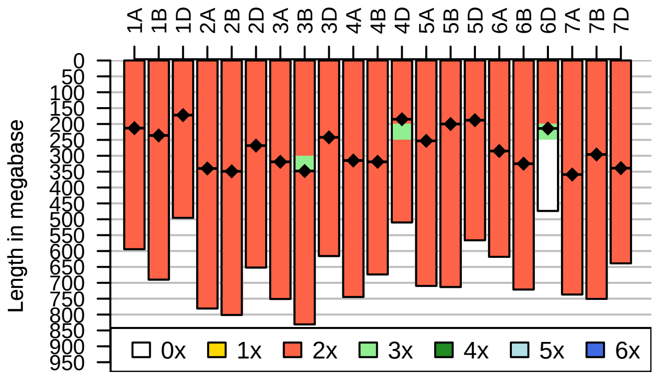

18S1−271−4 (6DL−3)

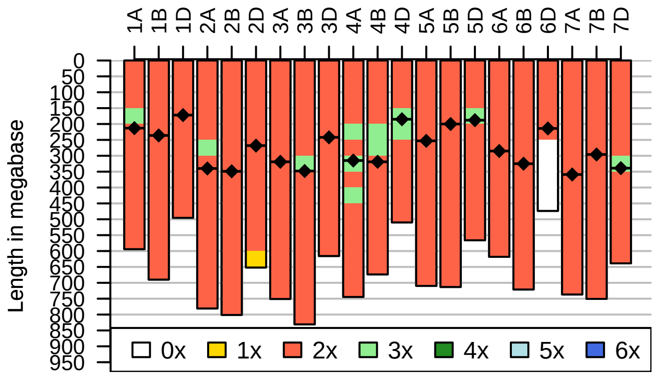

18S1−272−1 (6DL−3)

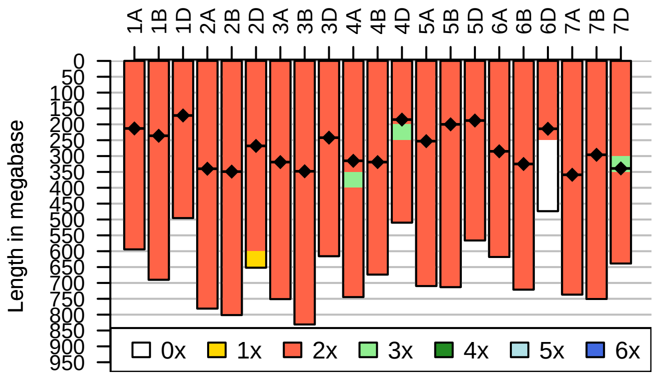

18S1−272−2 (6DL−3)

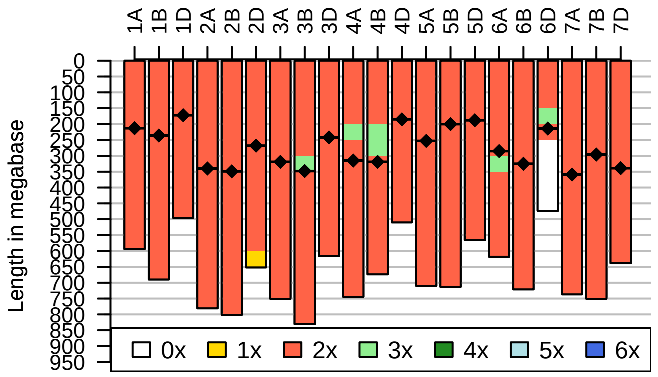

18S1−272−3 (6DL−3)

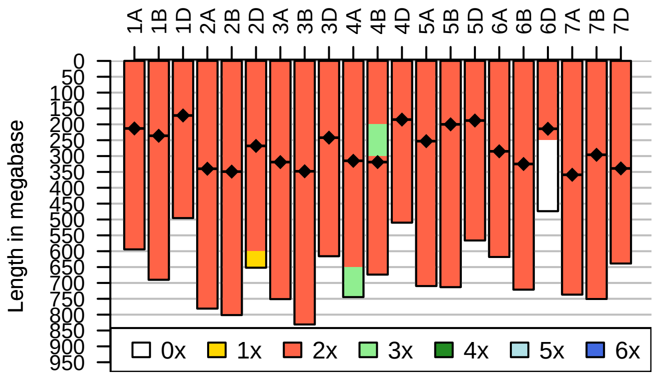

18S1−272−4 (6DL−3)

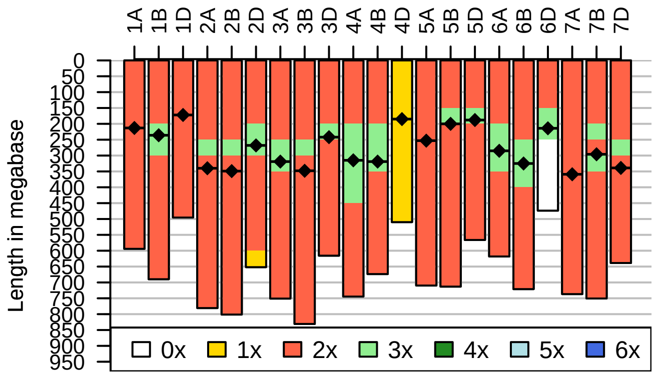

18S1−272−5 (6DL−5)

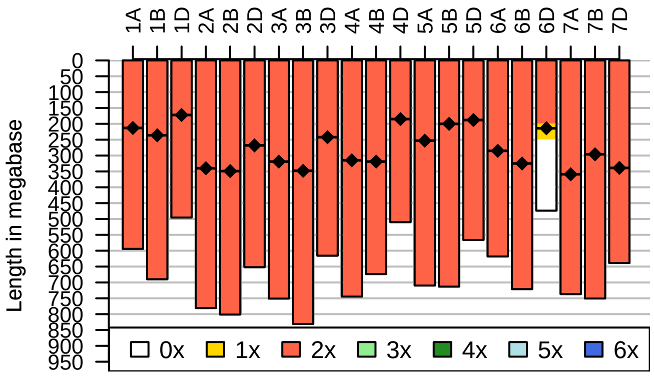

18S1−273 (TA4545 L5)

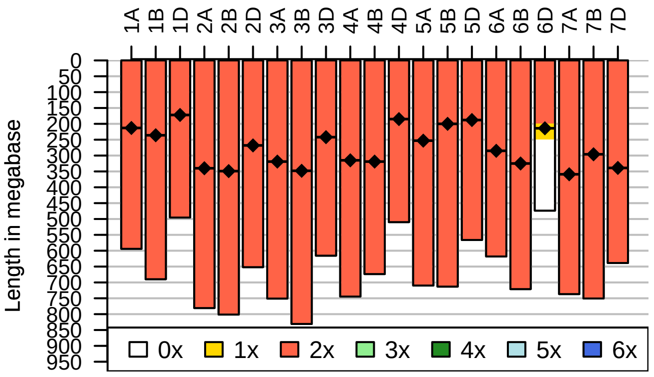

18S1−274−1 (6DL−5)

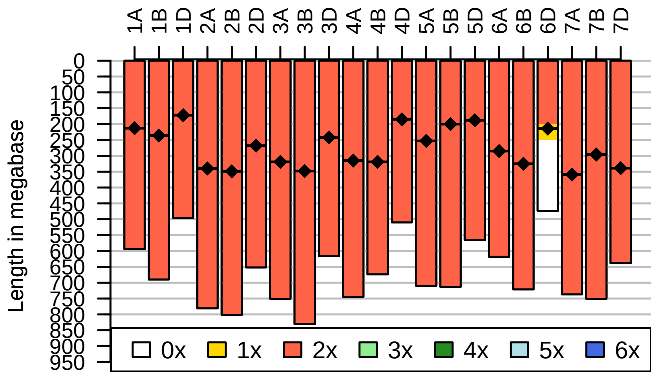

18S1−274−2 (6DL−5)

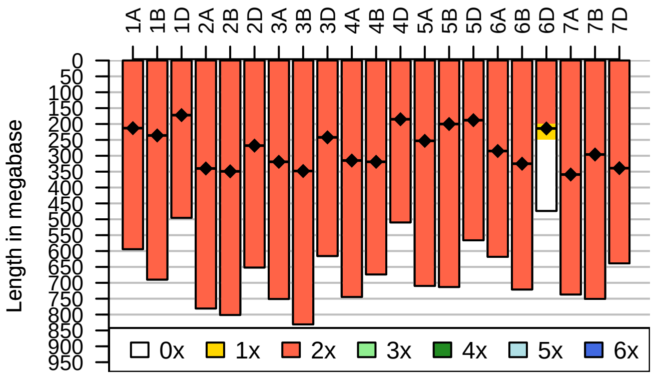

18S1−274−3 (6DL−5)

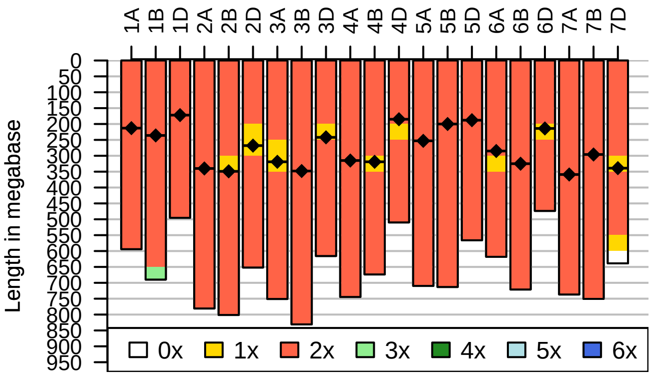

18S1−276−1 (7DL−8)

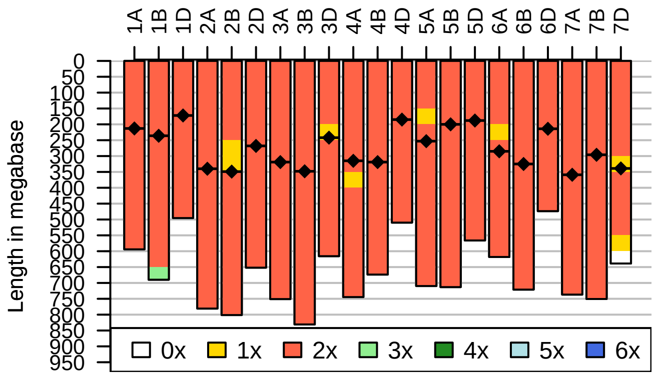

18S1−276−2 (7DL−8)

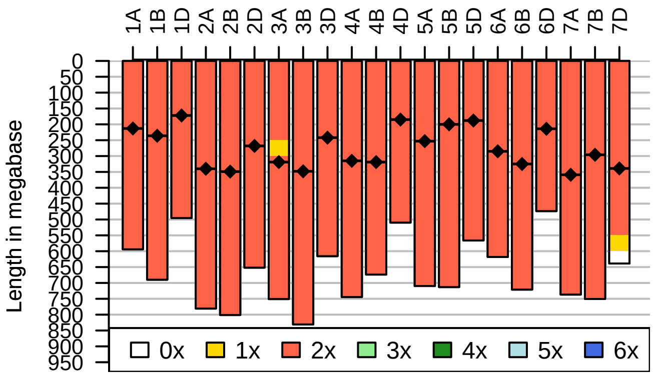

18S1−276−3 (7DL−8)

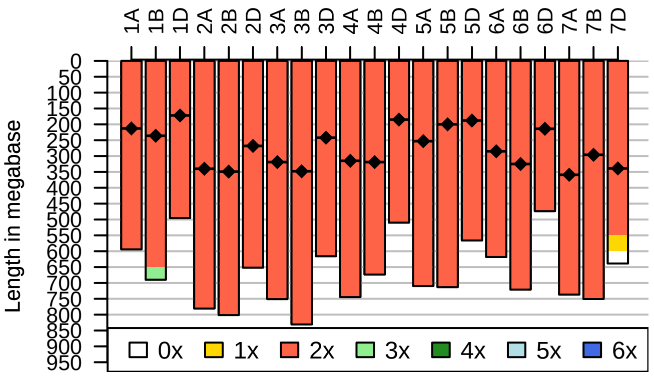

18S1−276−4 (7DL−8)

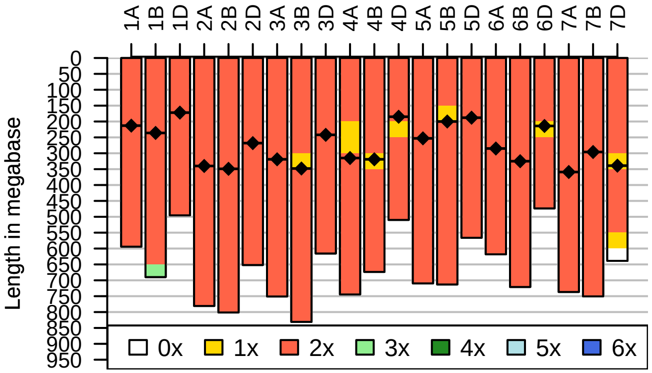

18S1−276−5 (7DL−8)

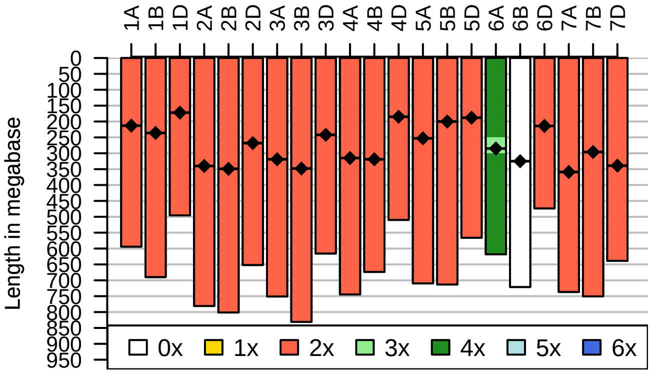

18S1−277−1 (N6B−T6A)

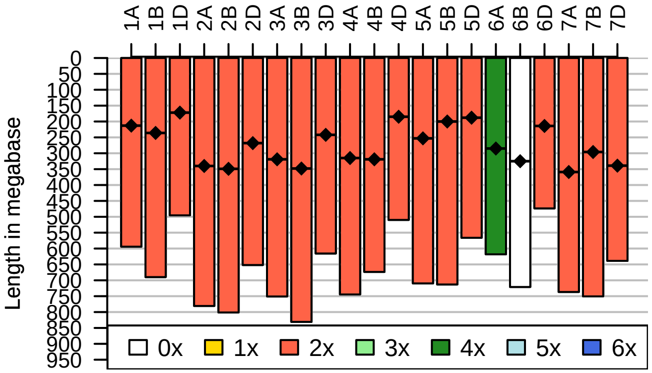

18S1−277−2 (N6B−T6A)

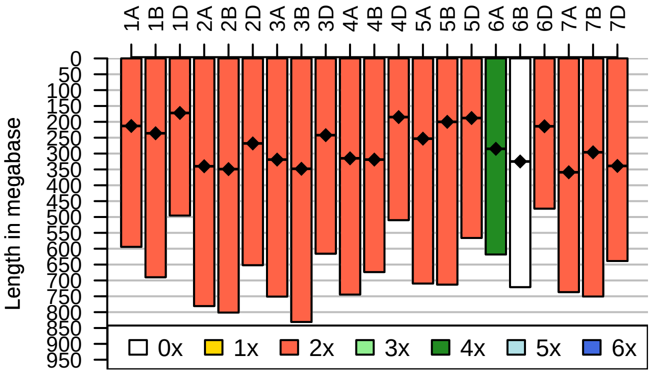

18S1−277−3 (N6B−T6A)

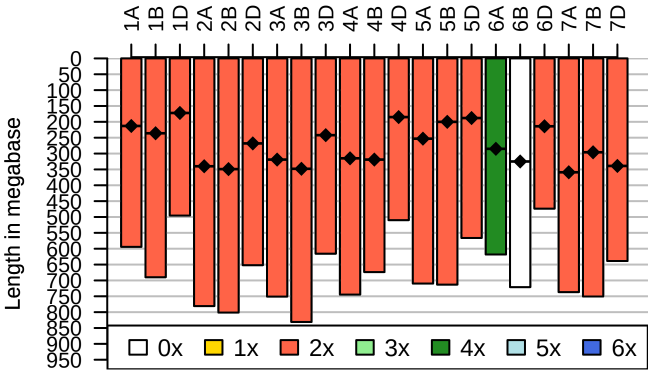

18S1−277−4 (N6B−T6A)

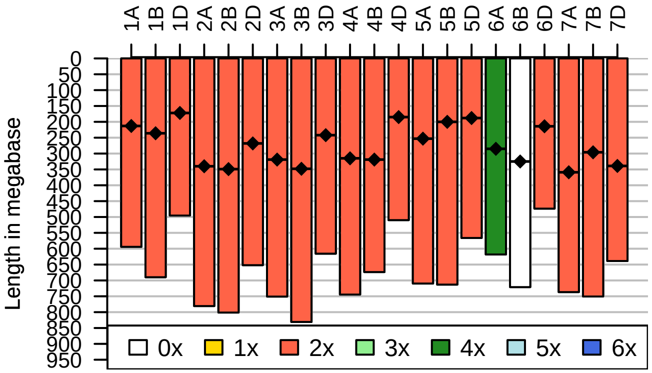

18S1−277−5 (N6B−T6A)

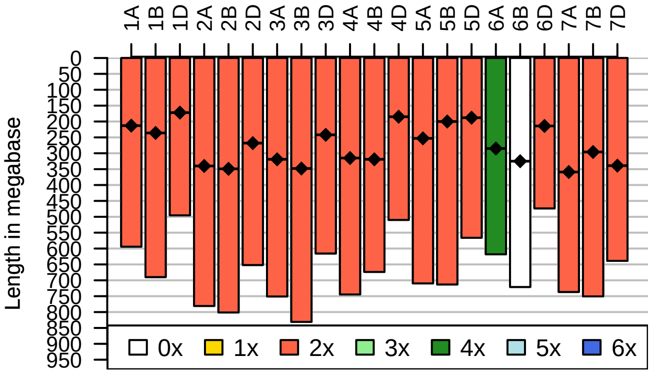

18S1−277−6 (N6B−T6A)

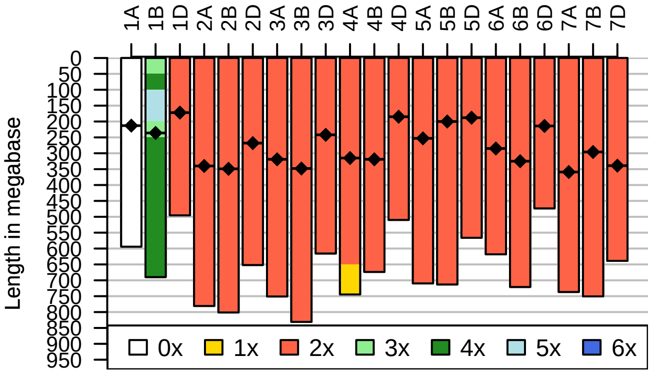

18S1−278−1 (N1A−T1B)

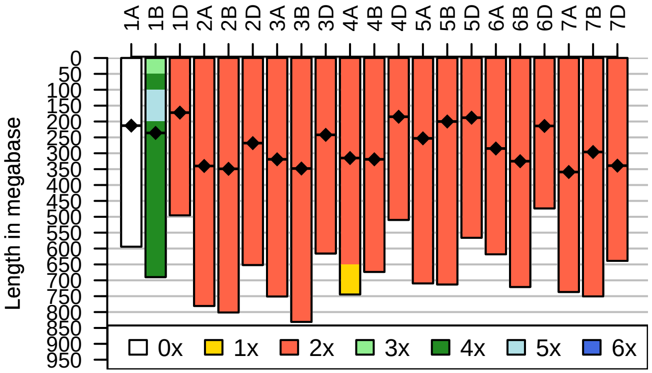

18S1−278−2 (N1A−T1B)

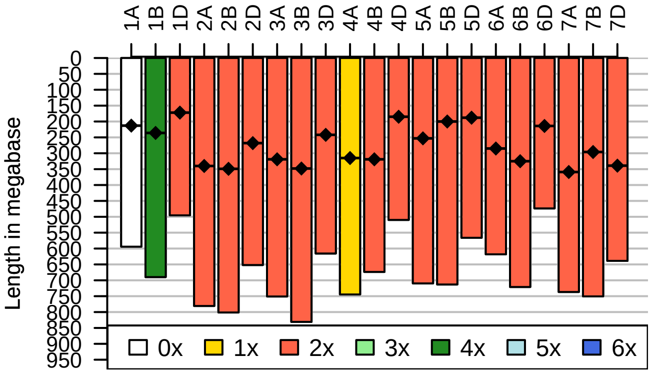

18S1−278−3 (N1A−T1B)

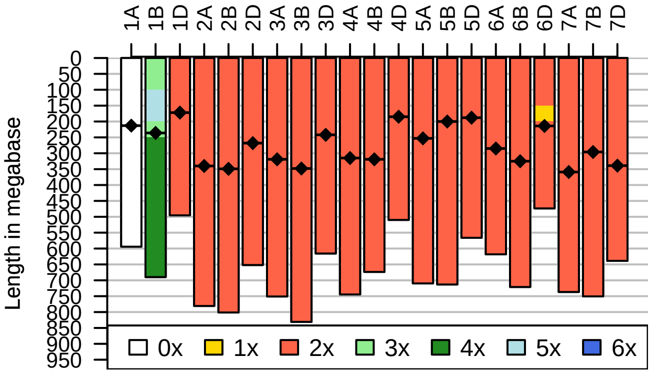

18S1−278−4 (N1A−T1B)

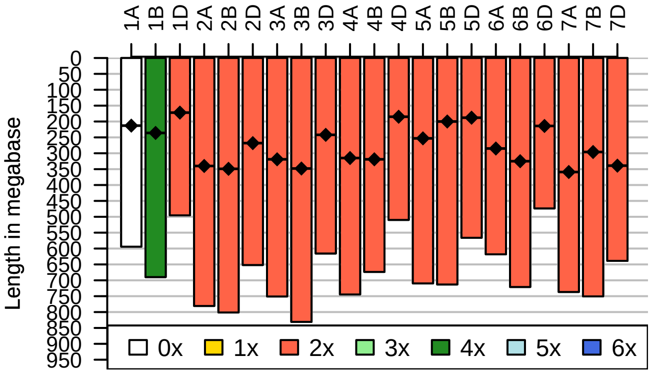

18S1−278−5 (N1A−T1B)

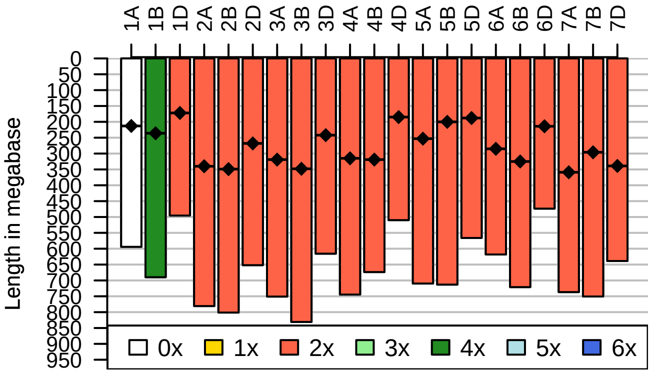

18S1−278−6 (N1A−T1B)

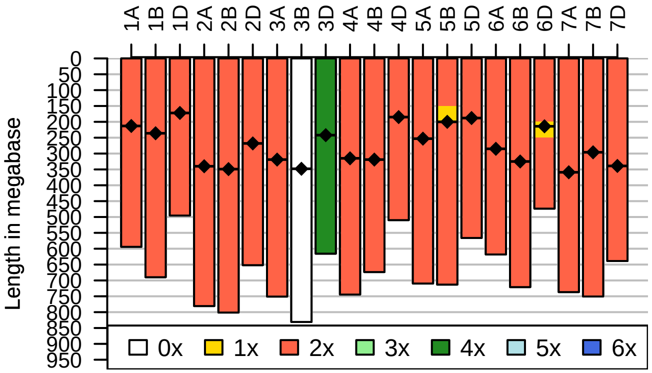

18S1−280−1 (N3B)

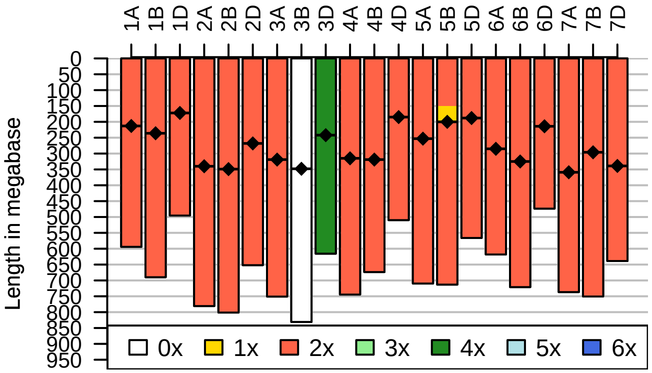

18S1−280−2 (N3B)

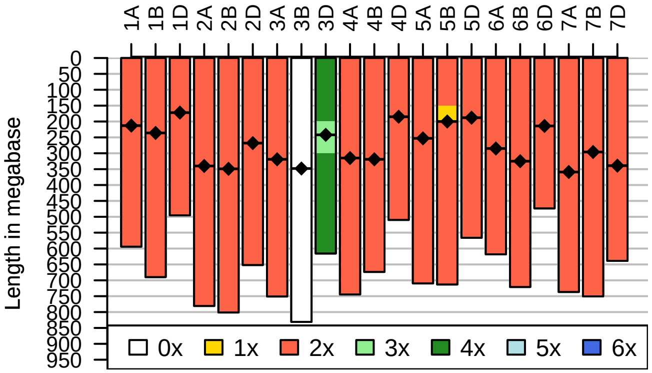

18S1−280−3 (N3B)

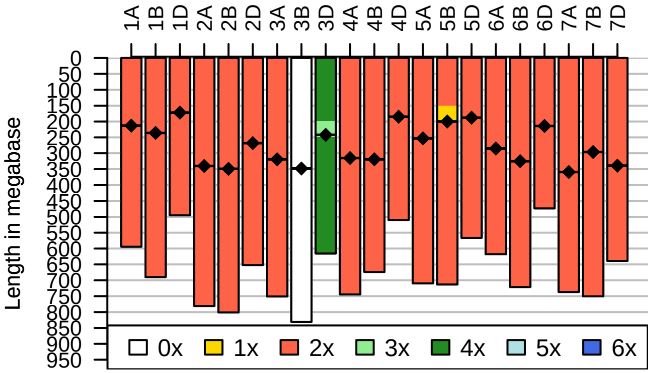

18S1−280−4 (N3B)

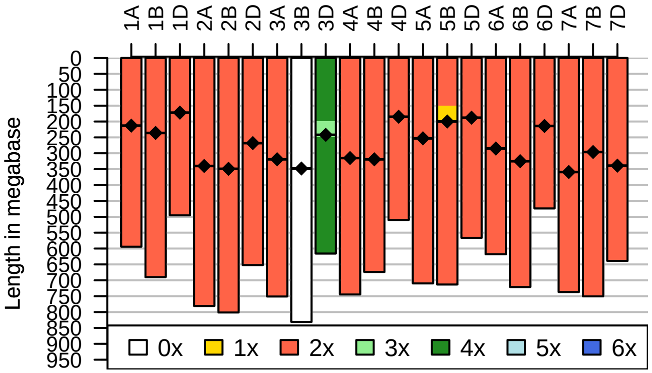

18S1−280−5 (N3B)

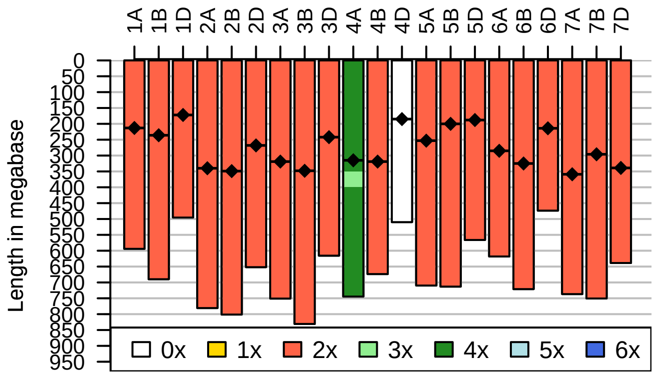

18S1−281−1 (N4D)

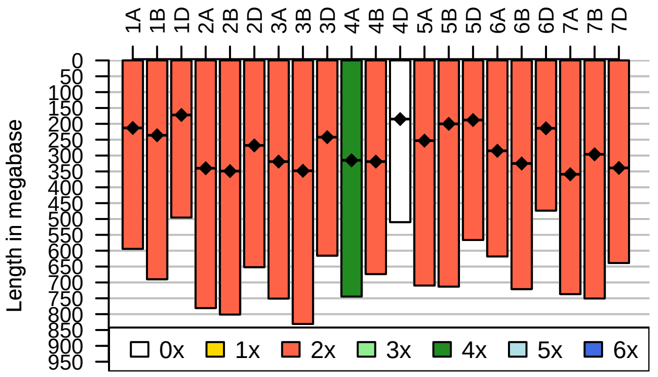

18S1−281−2 (N4D)

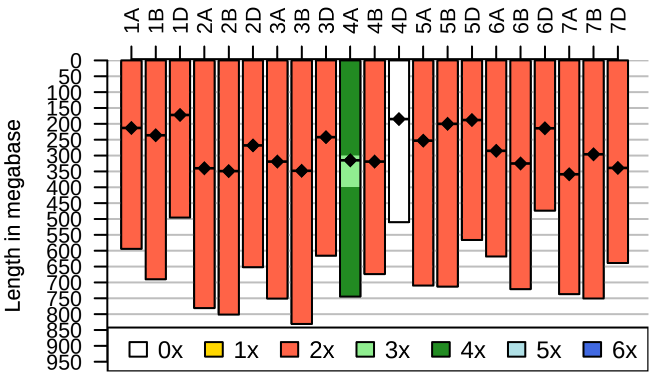

18S1−281−3 (N4D)

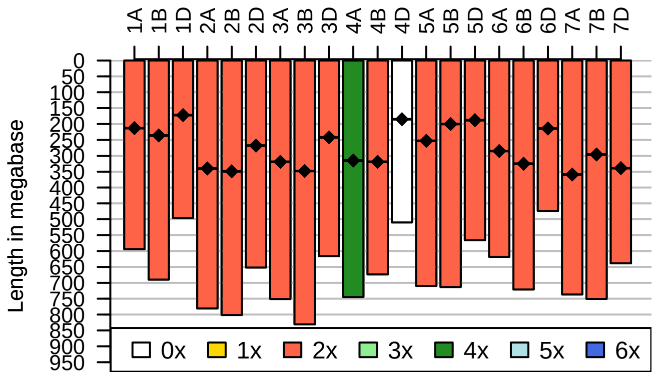

18S1−281−4 (N4D)

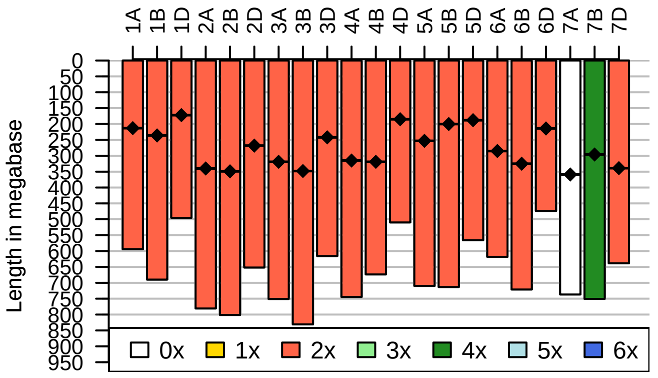

18S1−282−1 (N7A)

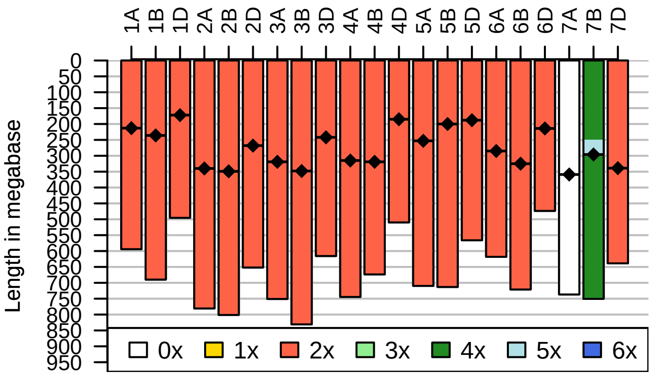

18S1−282−2 (N7A)

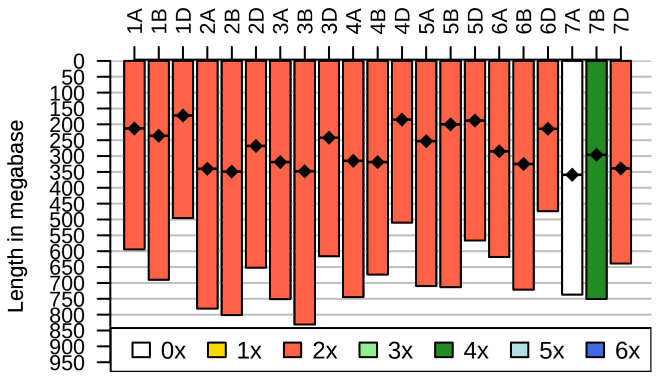

18S1−282−3 (N7A)

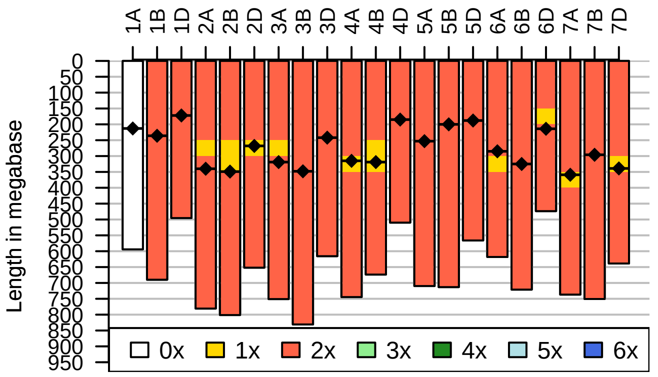

18S1−283−1 (N1A)

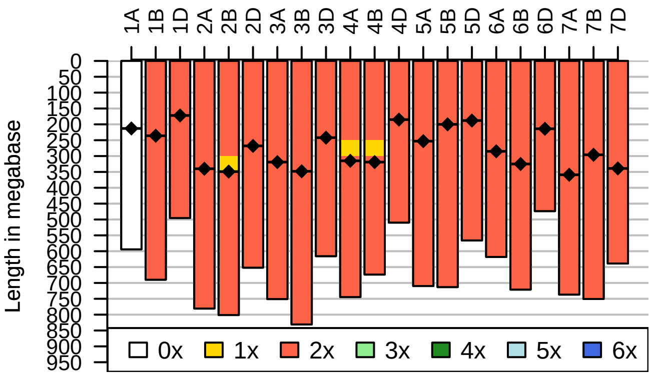

18S1−283−2 (N1A)

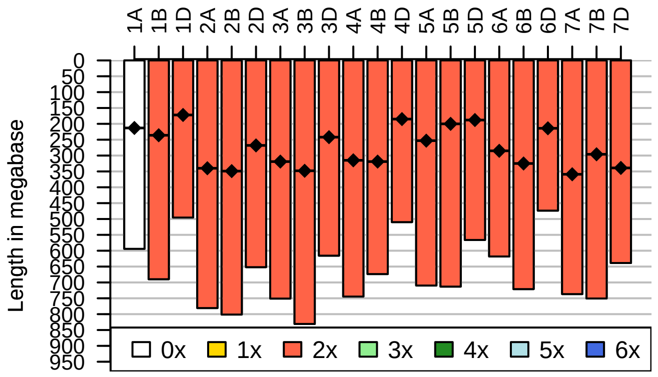

18S1−283−3 (N1A)

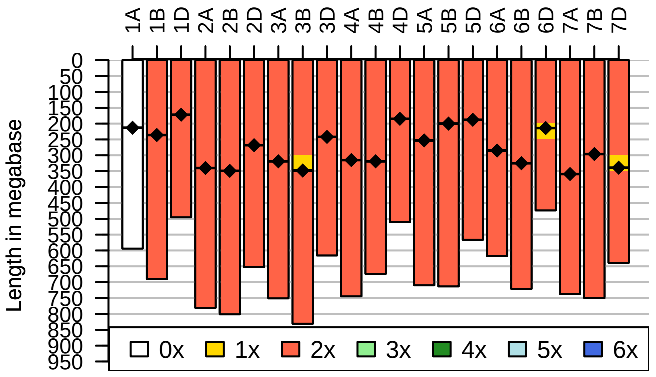

18S1−283−4 (N1A)

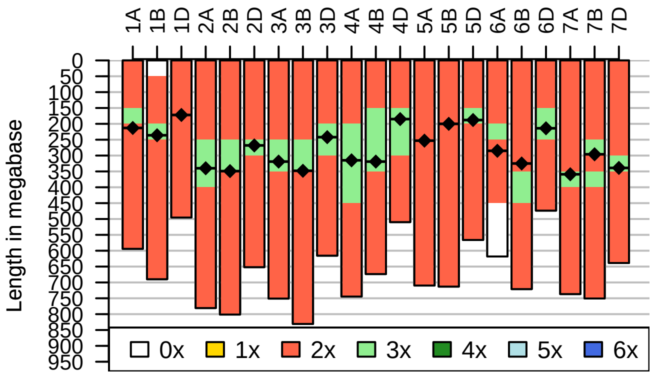

18S1−284−1 (6AL−6, 1BS−18)

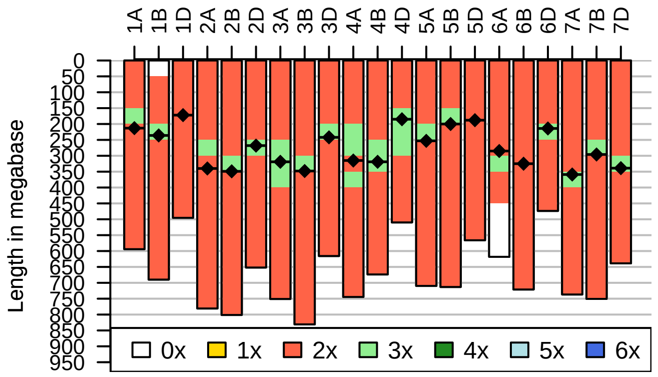

18S1−284−2 (6AL−6, 1BS−18)

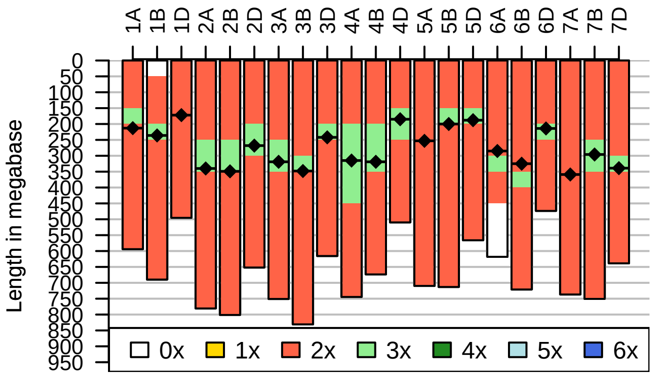

18S1−284−3 (6AL−6, 1BS−18)

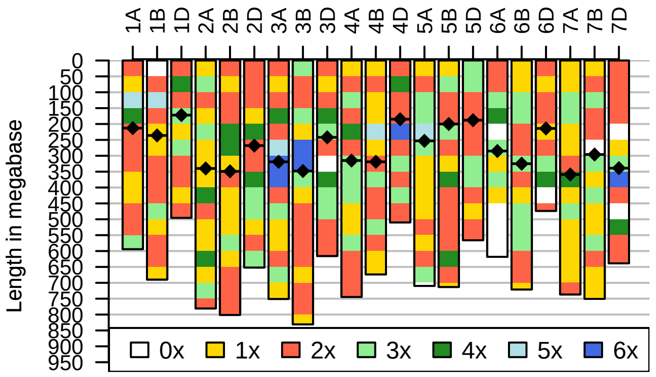

18S1−284−4 (6AL−6, 1BS−18)

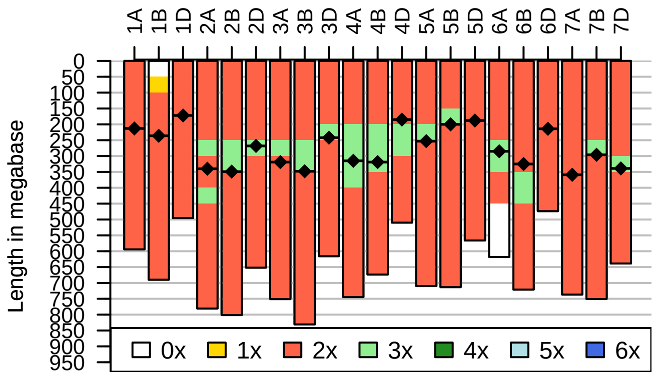

18S1−284−5 (6AL−6, 1BS−18)

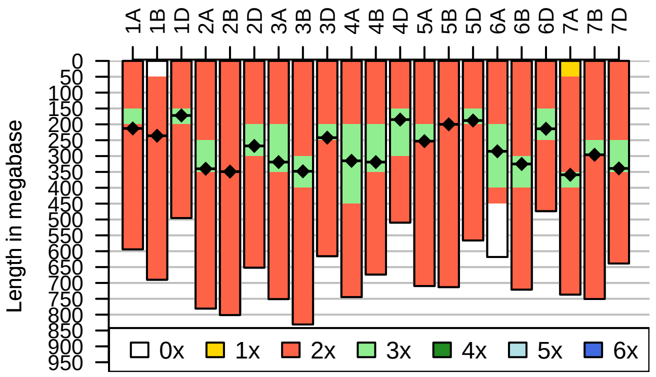

18S1−285 (6AL−6, 1BS−18)

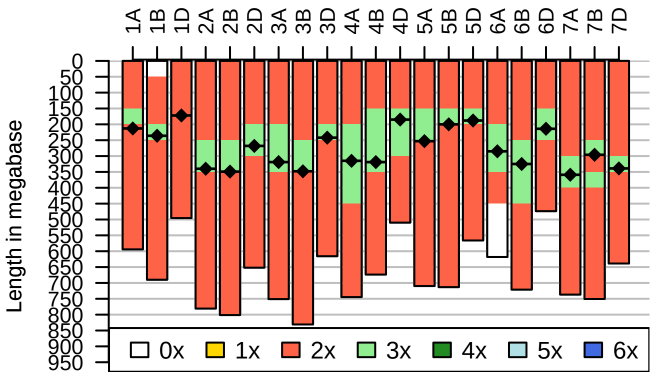

18S1−286−1 (6AL−6, 1BS−18)

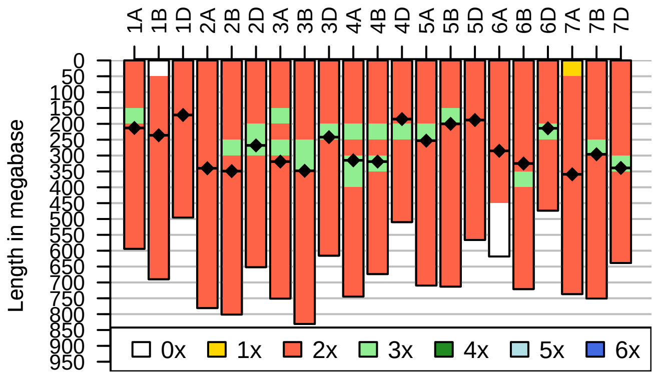

18S1−286−2 (6AL−6, 1BS−18)

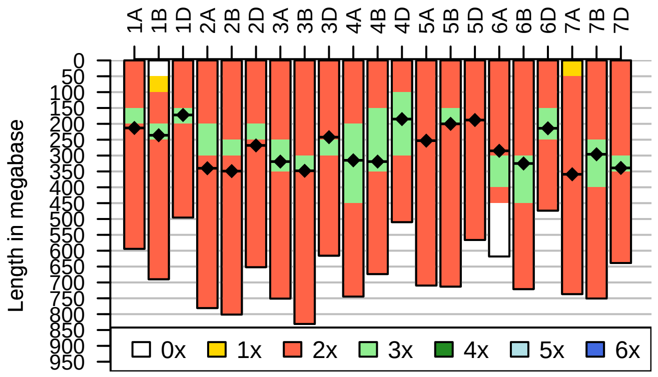

18S1−286−3 (6AL−6, 1BS−18)

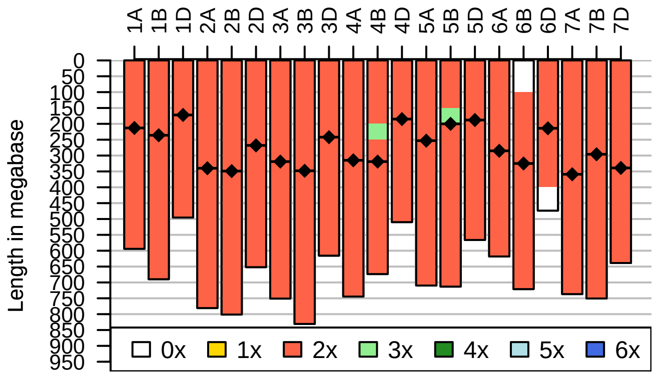

18S1−287−1 (6BS−3, 6DL−12)

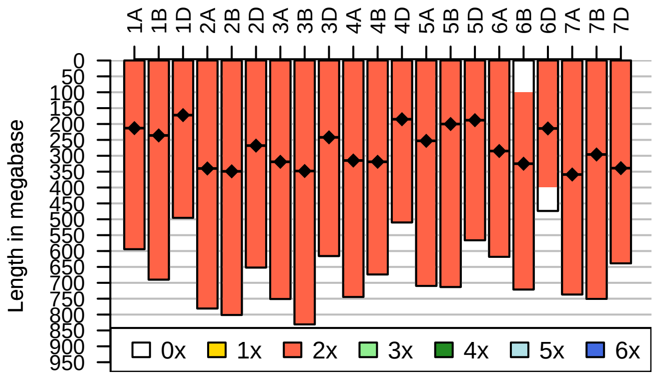

18S1−287−2 (6BS−3, 6DL−12)

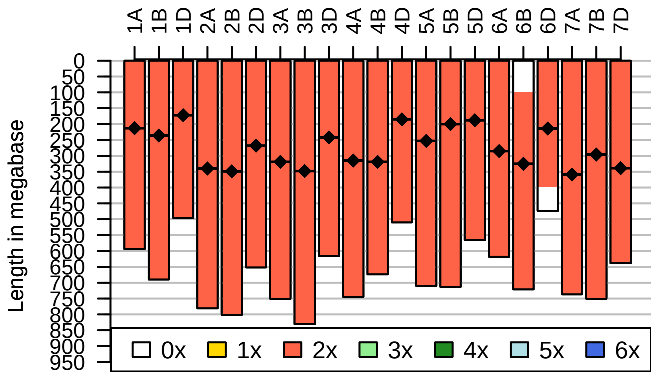

18S1−288−1 (6BS−3, 6DL−12)

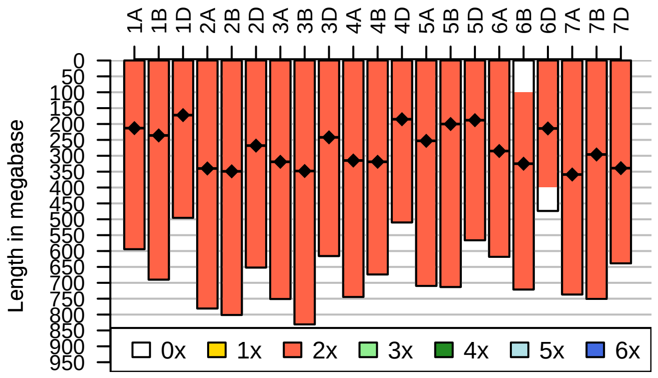

18S1−288−2 (6BS−3, 6DL−12)

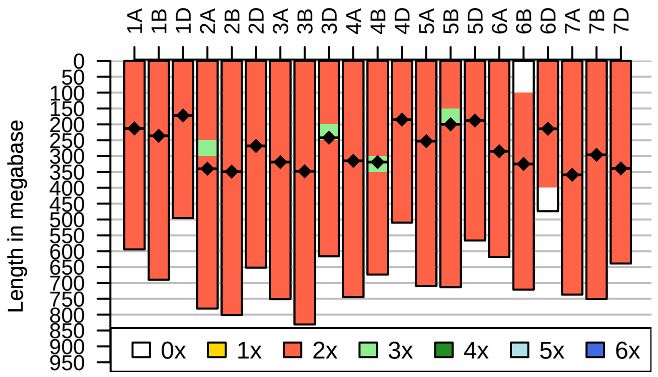

18S1−288−3 (6BS−3, 6DL−12)

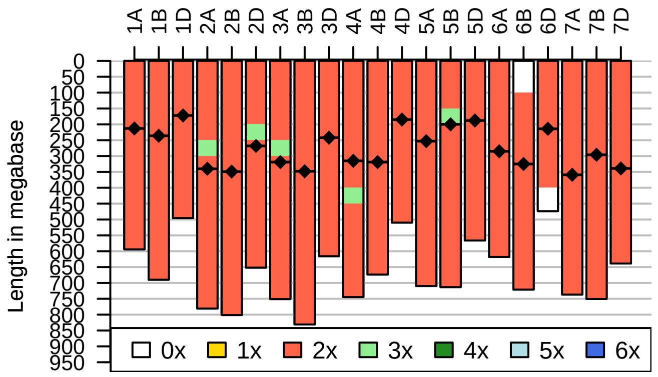

18S1−289−1 (6BS−3, 6DL−12)

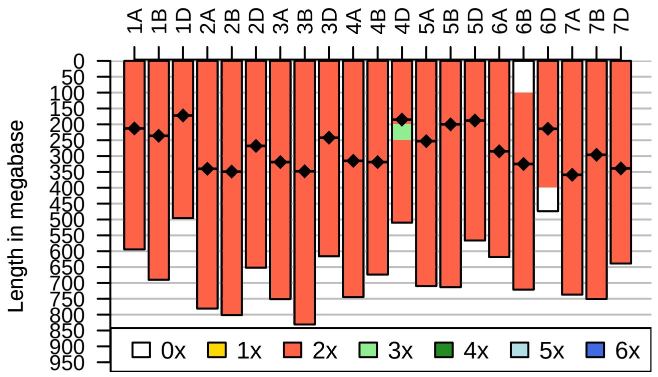

18S1−289−2 (6BS−3, 6DL−12)

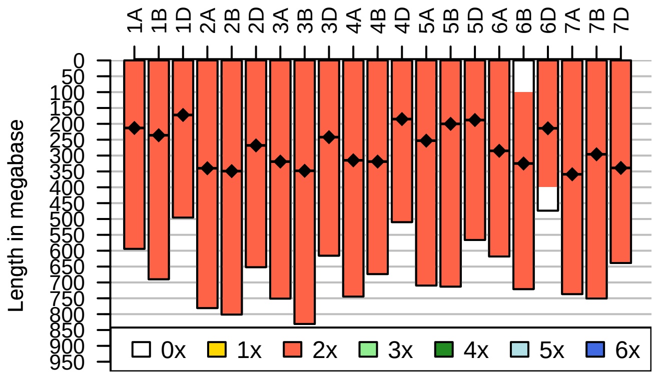

18S1−289−3 (6BS−3, 6DL−12)

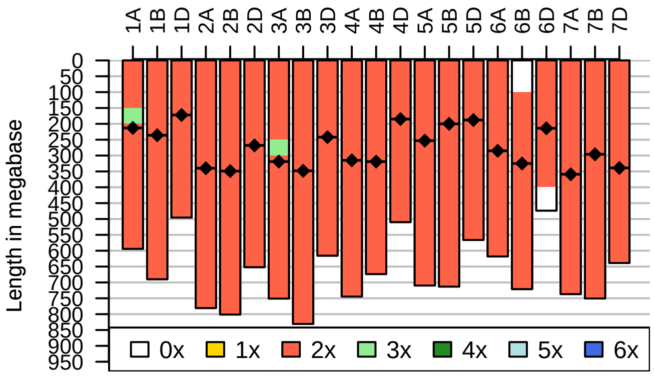

18S1−289−4 (6BS−3, 6DL−12)

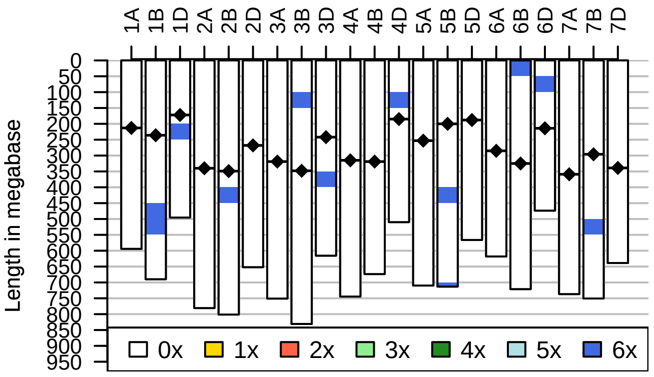

18S1−289−5 (6BS−3, 6DL−12)

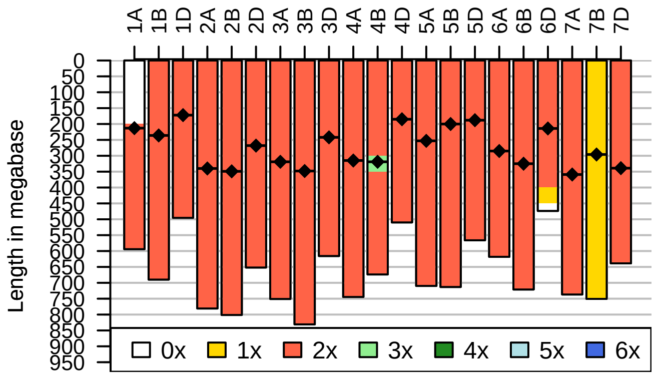

18S1−291−1 (1AS−5)

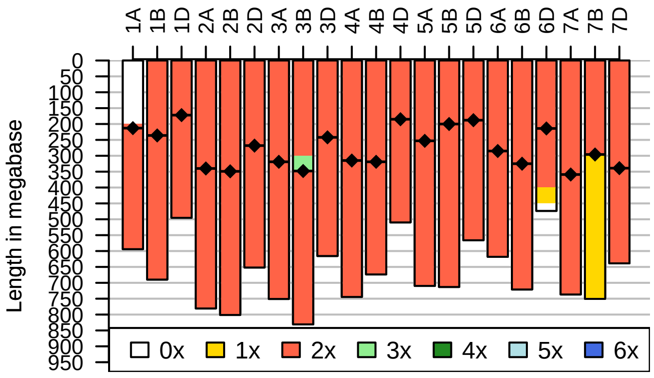

18S1−291−2 (1AS−5)

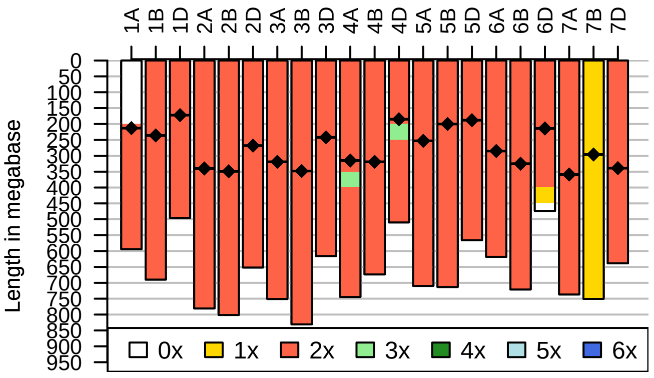

18S1−291−3 (1AS−5)

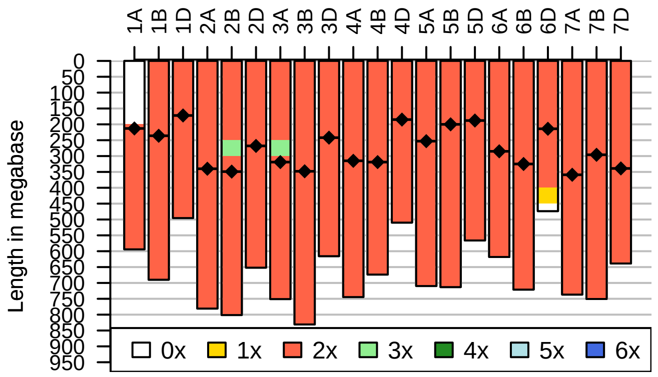

18S1−292−1 (1AS−5)

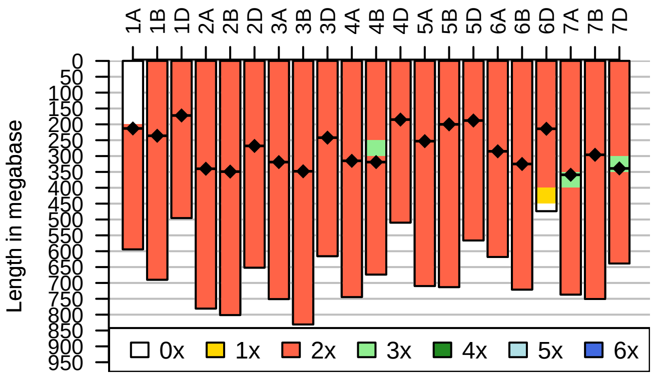

18S1−292−2 (1AS−5)

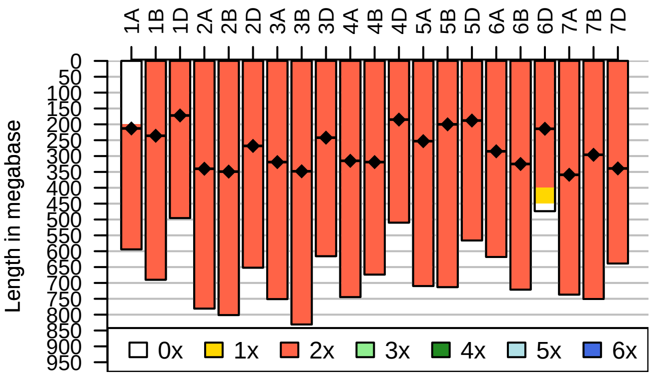

18S1−292−3 (1AS−5)

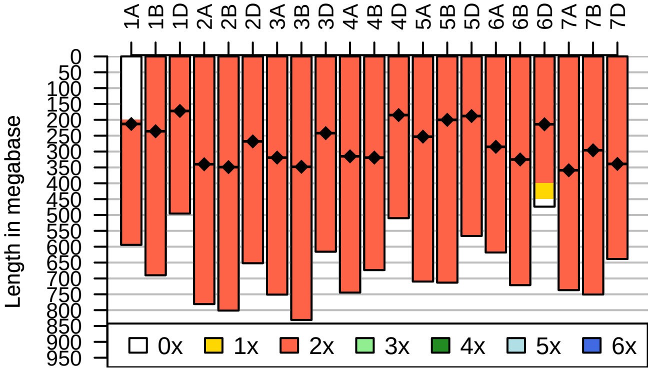

18S1−293−1 (1AS−5, 5AS−11, 6DL−13)

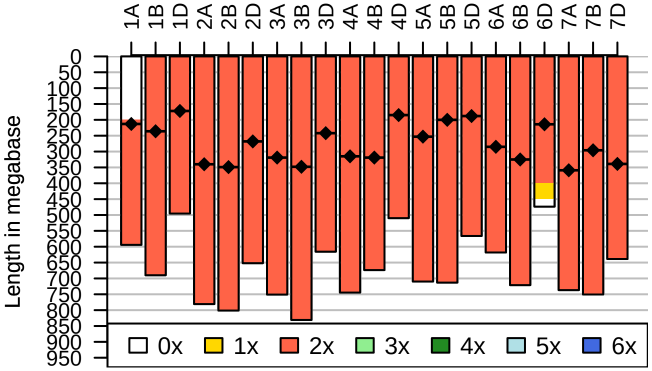

18S1−293−2 (1AS−5, 5AS−11, 6DL−13)

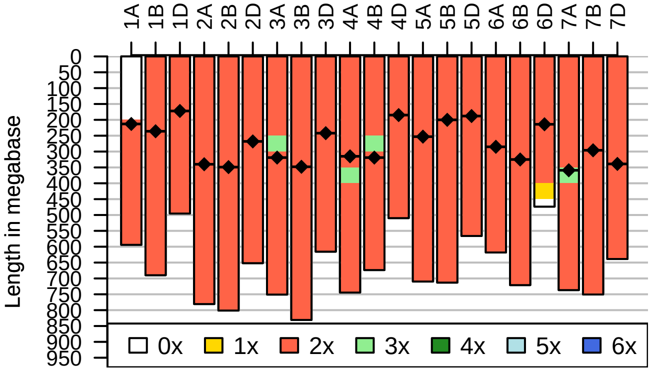

18S1−293−3 (1AS−5, 5AS−11, 6DL−13)

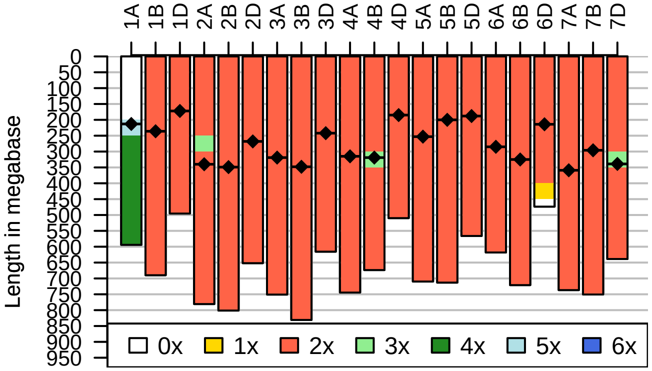

18S1−294−1 (1AS−5, 5AS−11, 6DL−13)

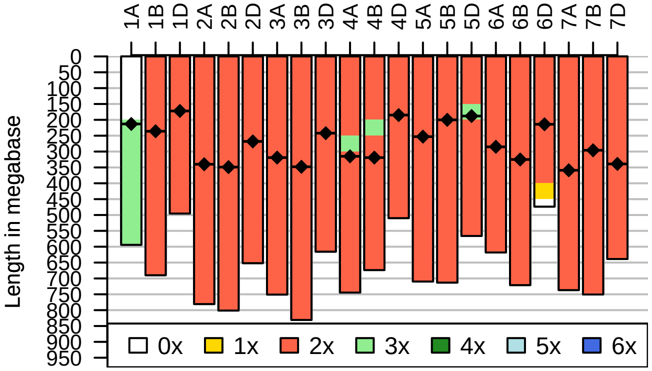

18S1−294−2 (1AS−5, 5AS−11, 6DL−13)

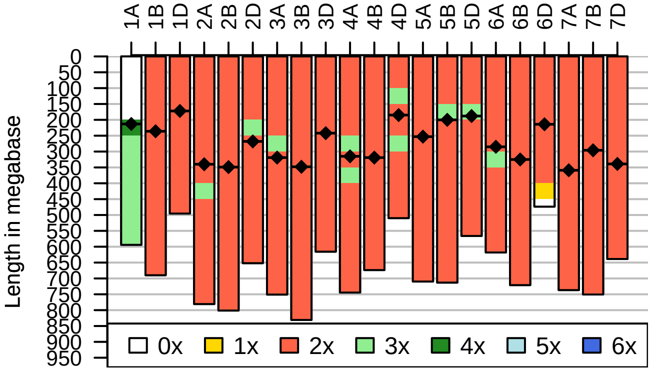

18S1−295−1 (1AS−5, 5AS−11, 6DL−13)

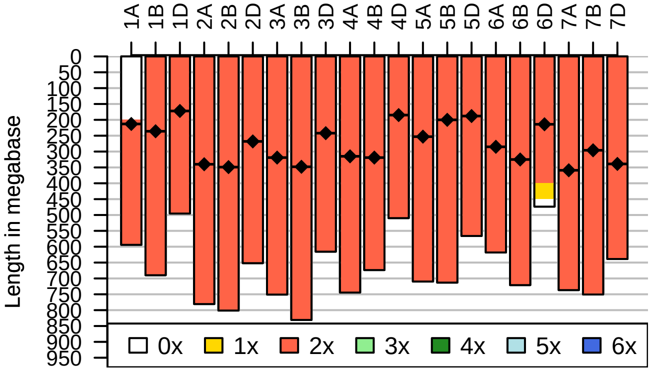

18S1−295−2 (1AS−5, 5AS−11, 6DL−13)

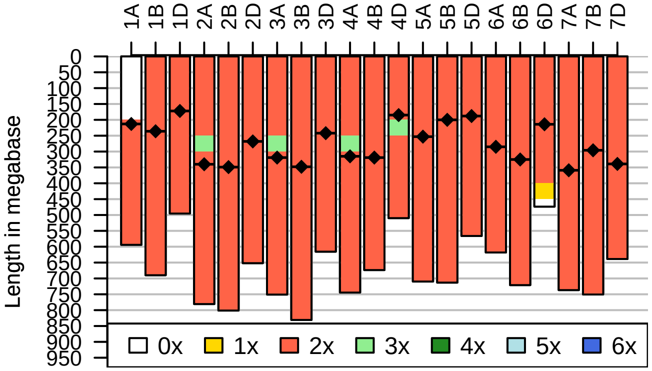

18S1−296−1 (1AS−5, 5AS−11, 6DL−13)

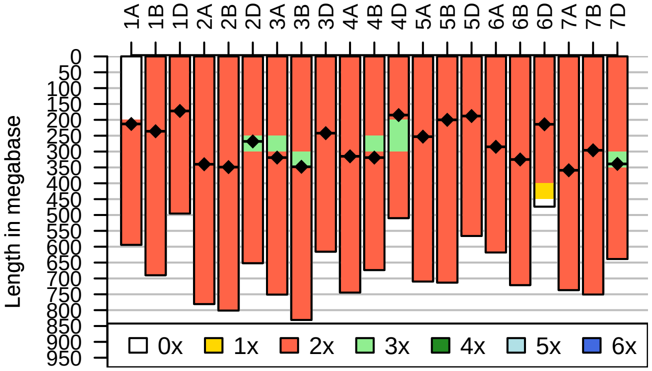

18S1−296−2 (1AS−5, 5AS−11, 6DL−13)

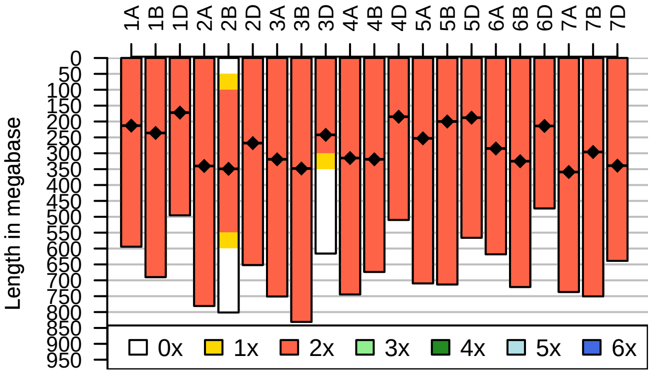

18S1−298−1 (TA4518 L15)

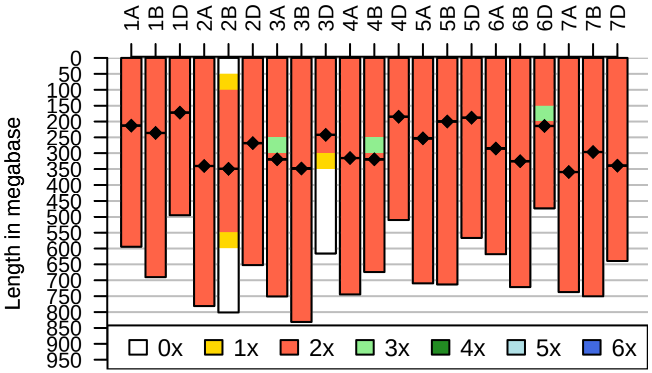

18S1−298−2 (TA4518 L15)

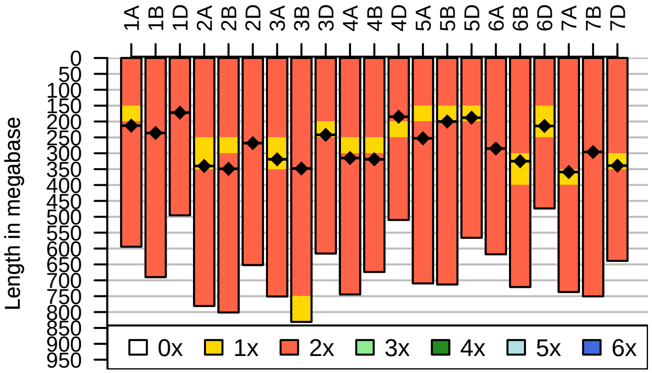

18S1−300−1 (2DL−1)

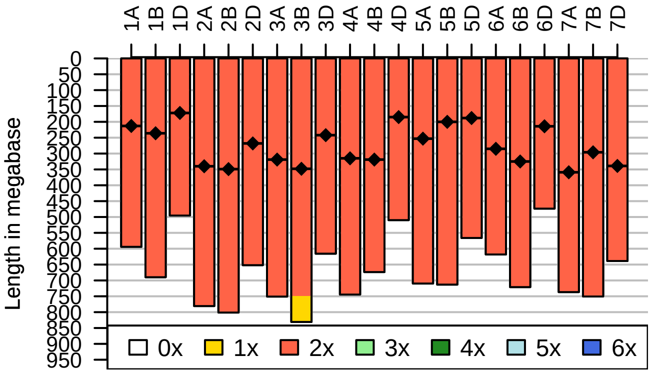

18S1−300−2 (2DL−1)

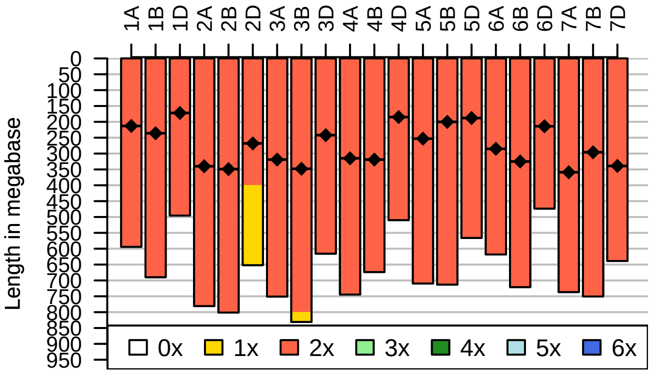

18S1−300−3 (2DL−1)

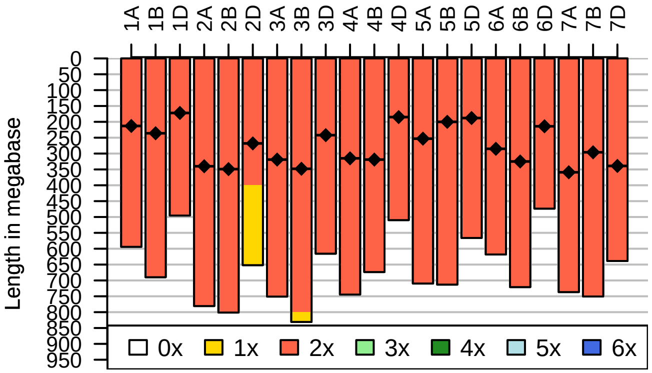

18S1−300−4 (2DL−1)

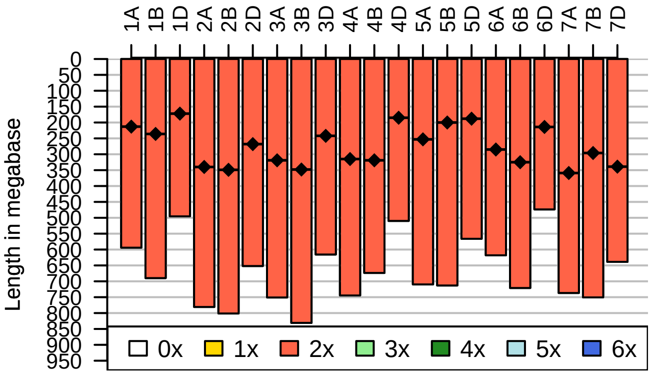

18S1−300−5 (2DL−1)

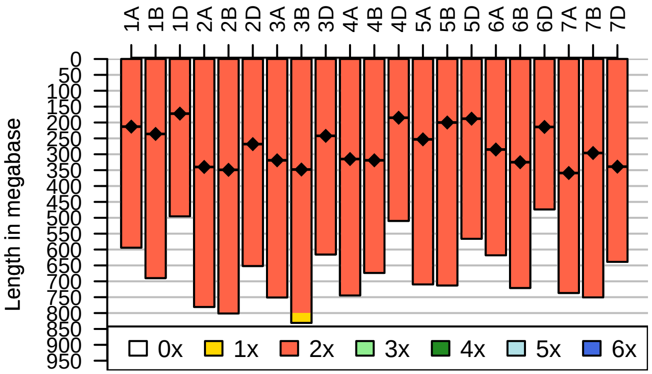

18S1−301−1 (2DL−1)

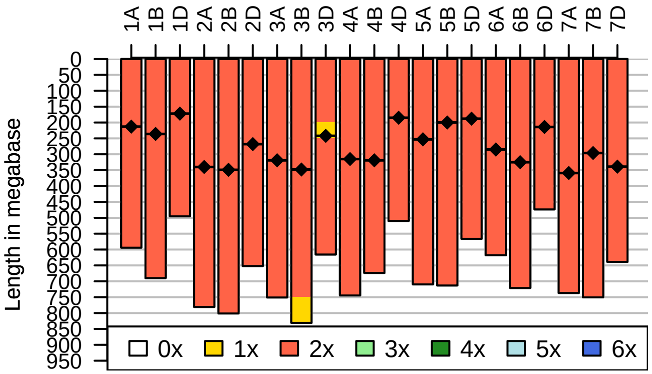

18S1−301−2 (2DL−1)

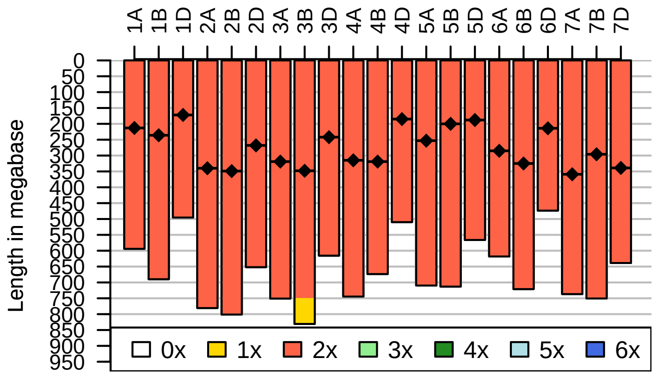

18S1−301−3 (2DL−1)

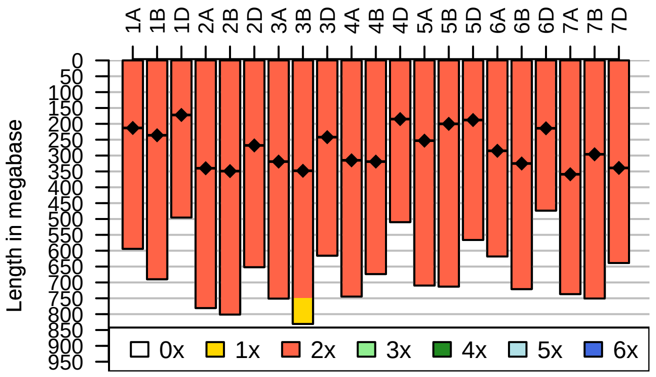

18S1−301−4 (2DL−1)

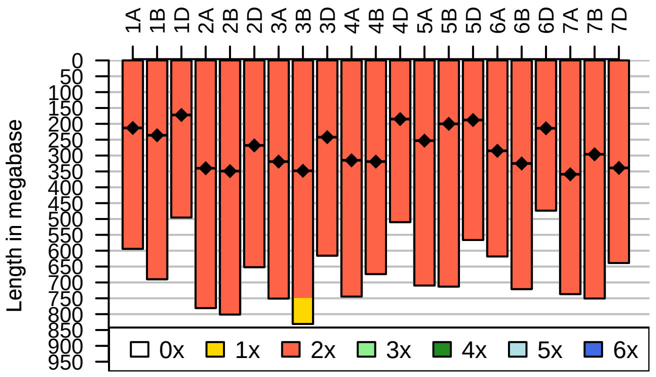

18S1−301−5 (2DL−1)

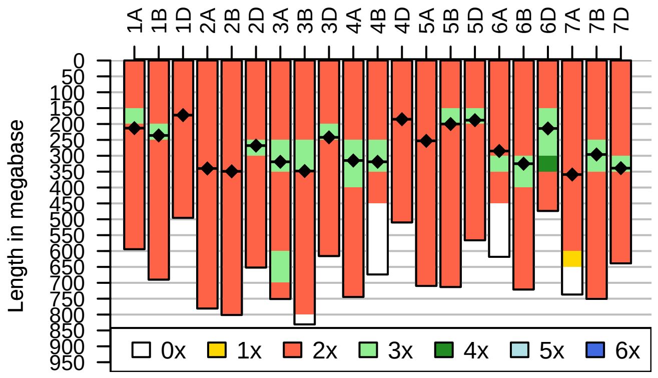

18S1−302−1 (6AL−7, 4BL−13, 7AL−21)

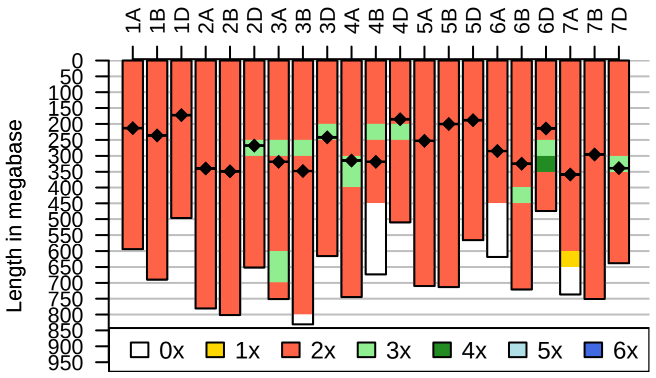

18S1−302−2 (6AL−7, 4BL−13, 7AL−21)

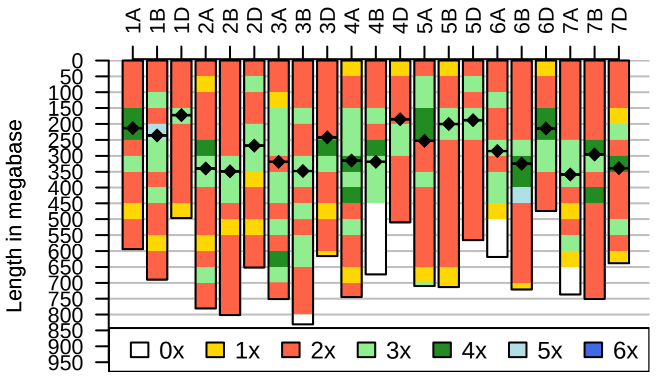

18S1−302−3 (6AL−7, 4BL−13, 7AL−21)

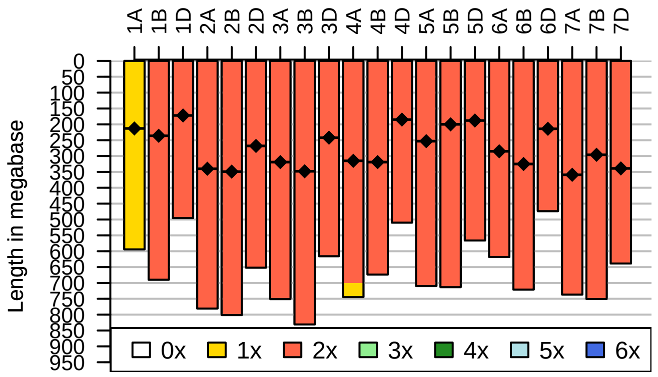

18SI−530−1 M1A

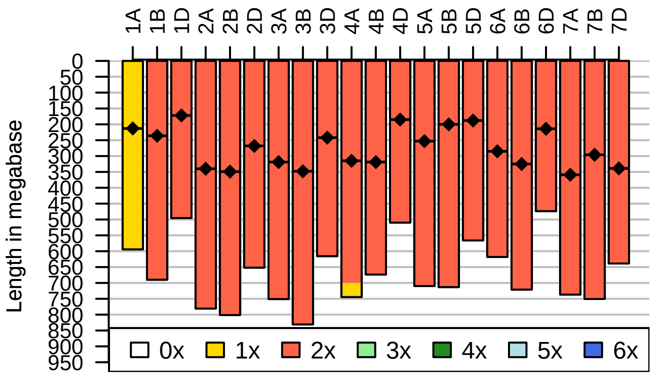

18SI−530−10 NA

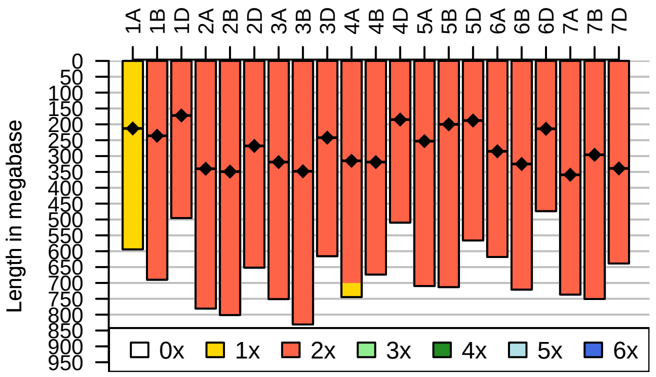

18SI−530−11 NA

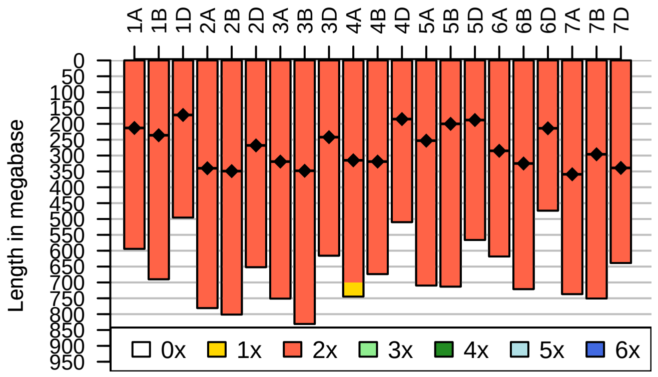

18SI−530−13 NA

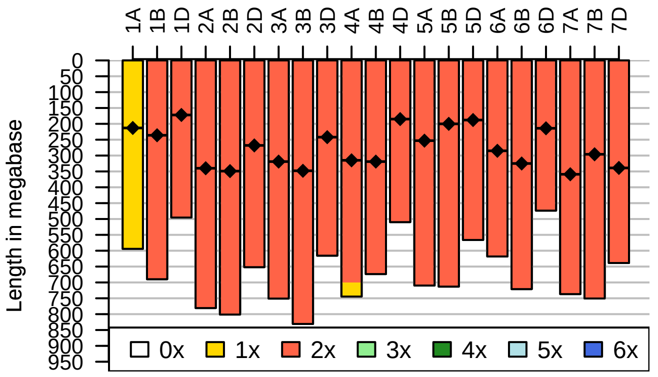

18SI−530−14 NA

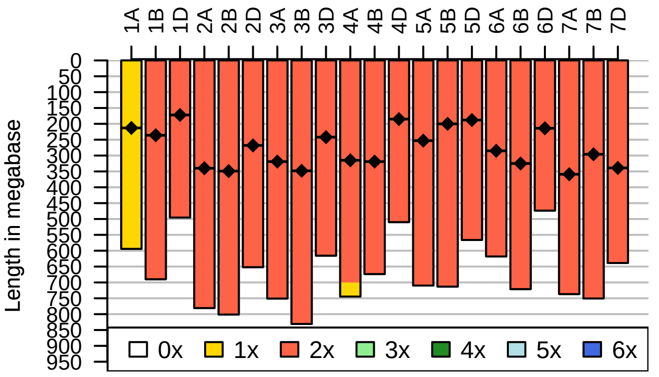

18SI−530−19 NA

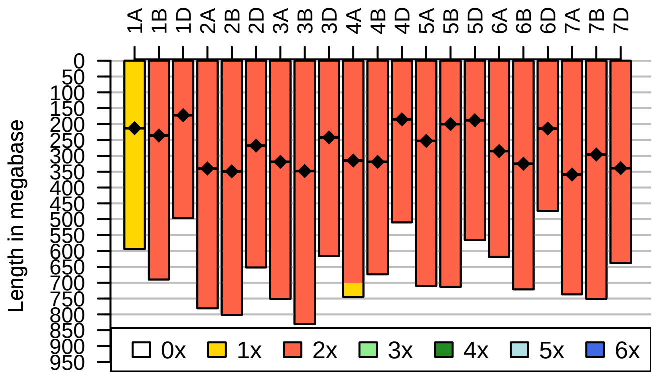

18SI−530−2 M1A

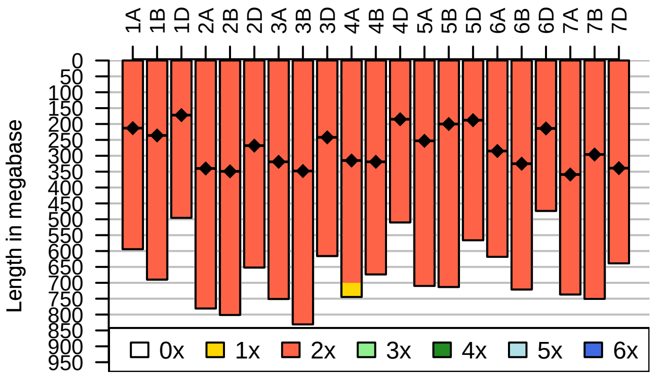

18SI−530−3 Euploid

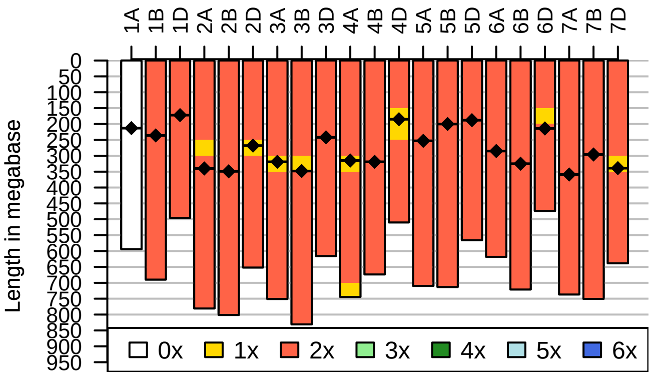

18SI−530−4 M1A

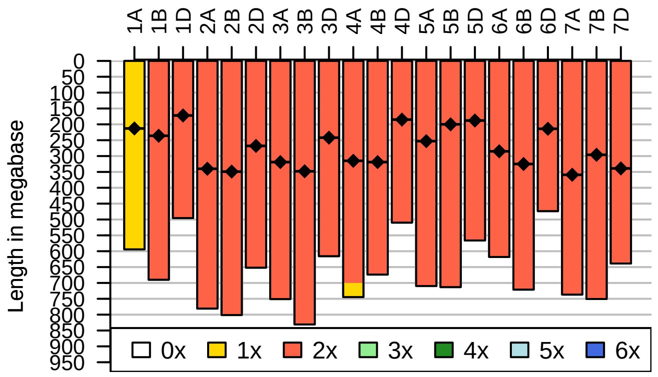

18SI−530−5 M1A

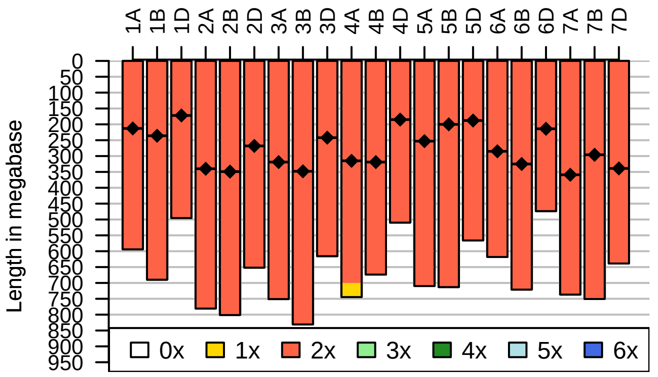

18SI−530−6 Euploid

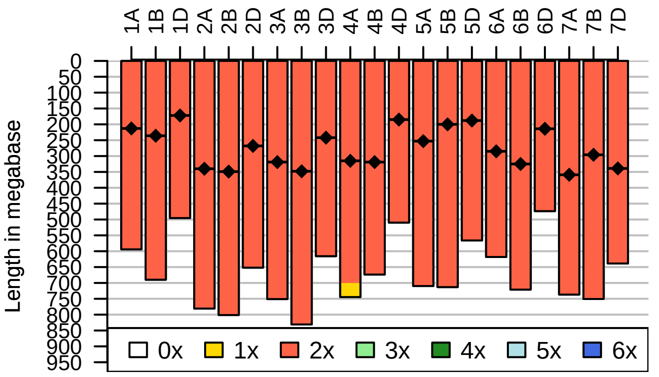

18SI−530−7 Euploid

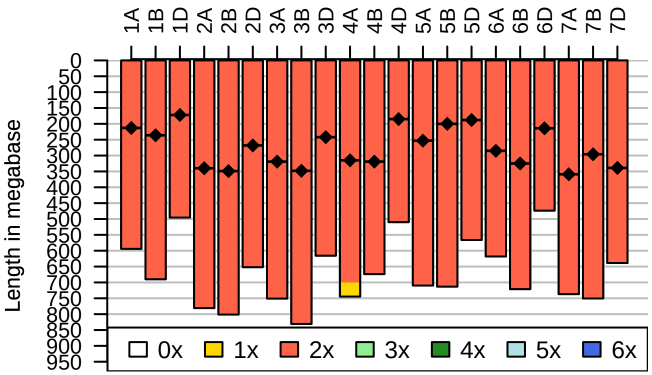

18SI−530−8 Euploid

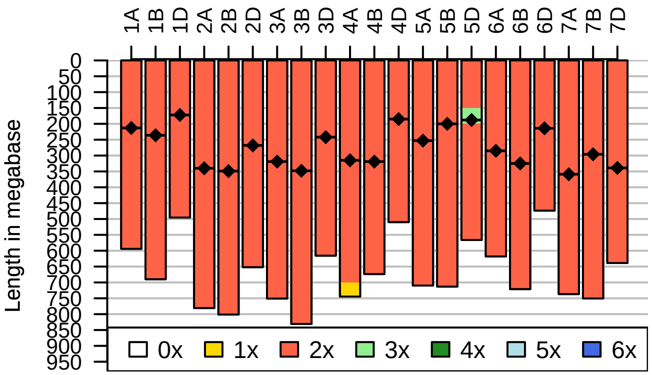

18SI−530−9 NA

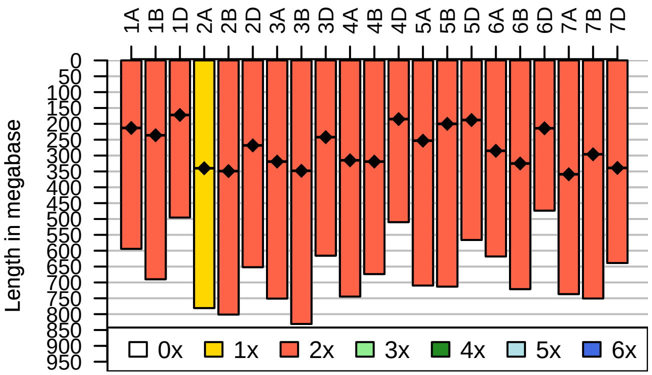

18SI−531−1 M2A

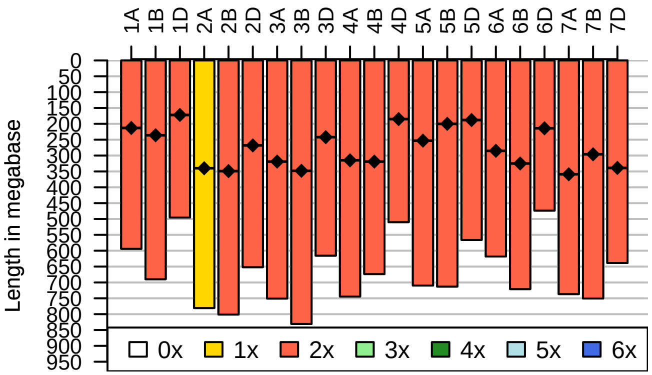

18SI−531−10 M2A

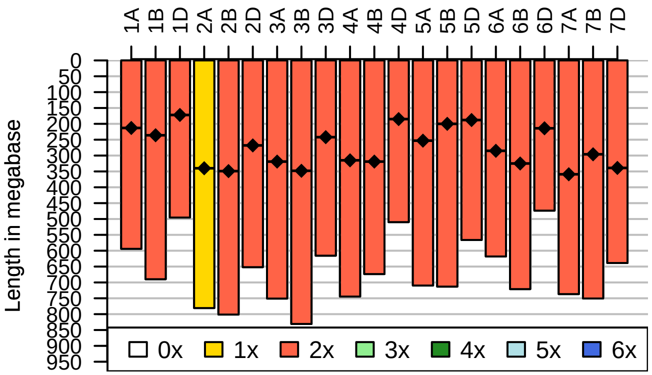

18SI−531−11 NA

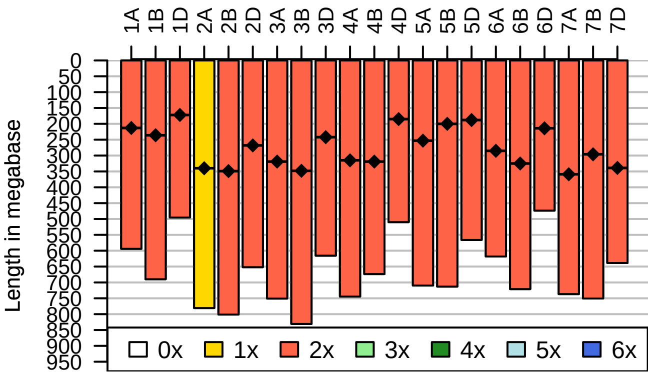

18SI−531−12 NA

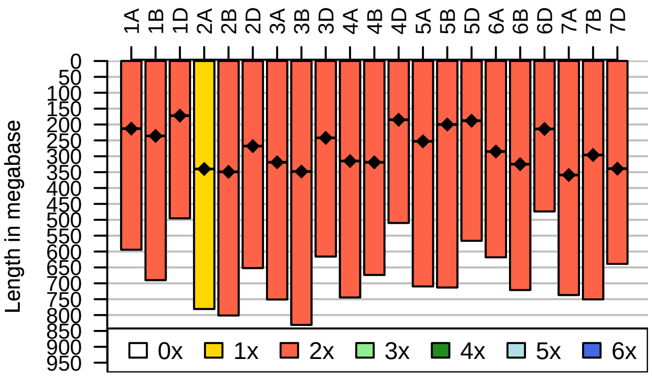

18SI−531−13 NA

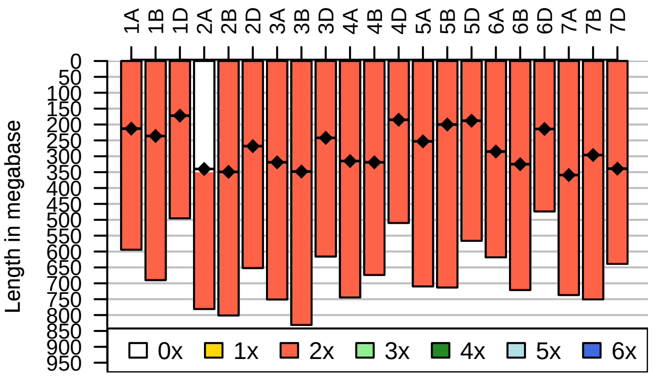

18SI−531−14 NA

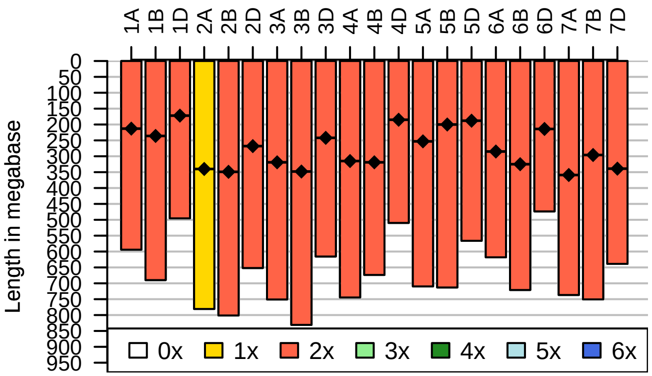

18SI−531−15 NA

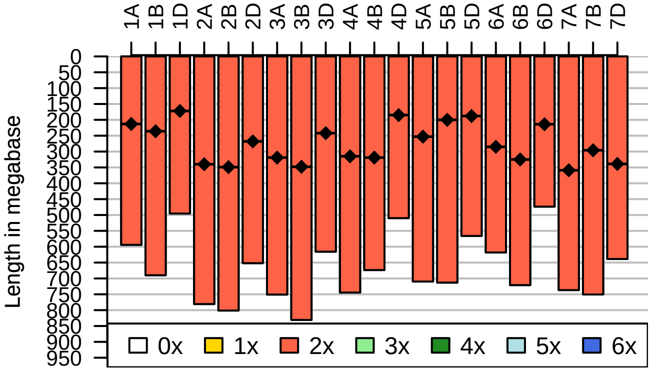

18SI−531−16 NA

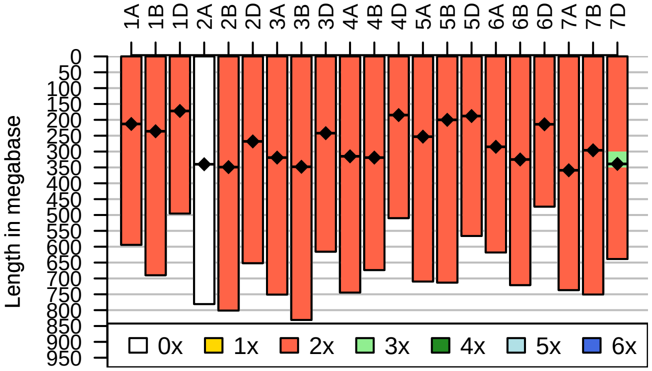

18SI−531−17 NA

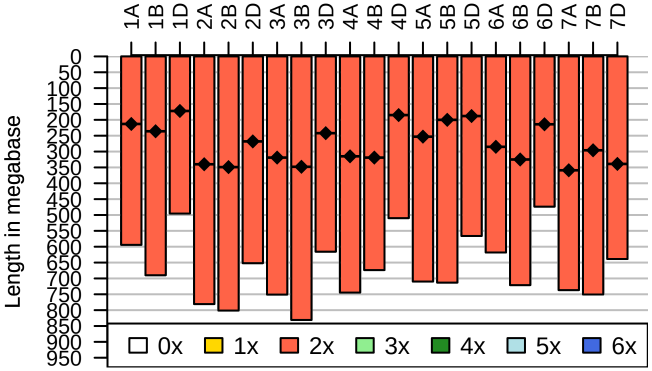

18SI−531−18 NA

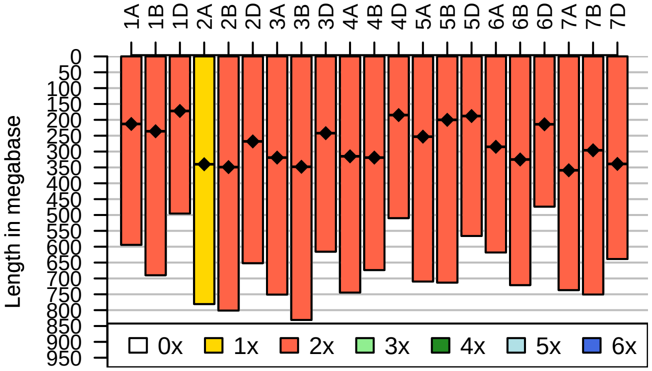

18SI−531−2 M2A

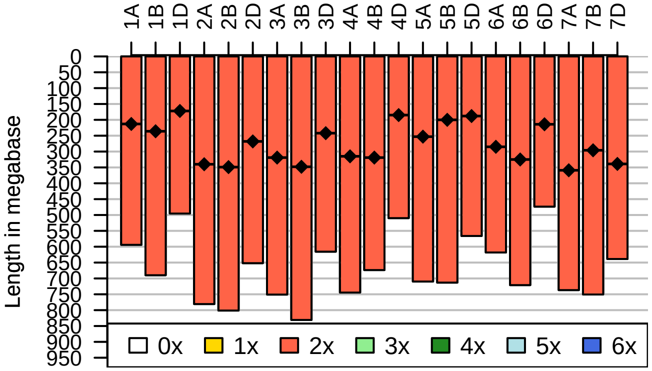

18SI−531−3 Euploid

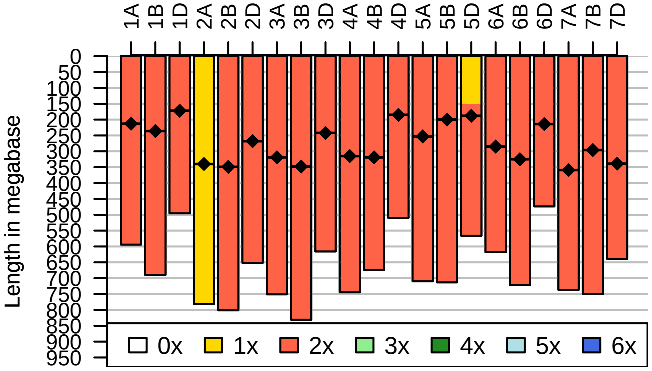

18SI−531−4 M2A

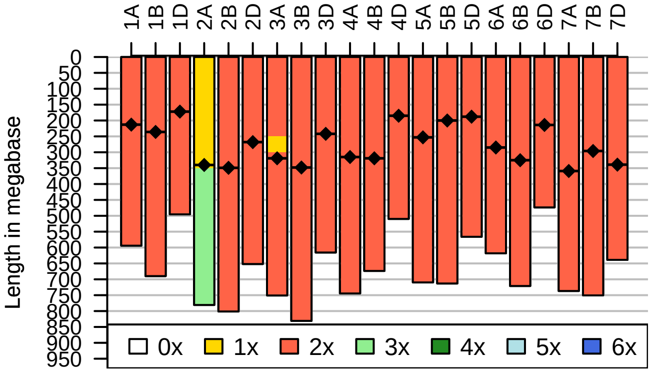

18SI−531−5 NA

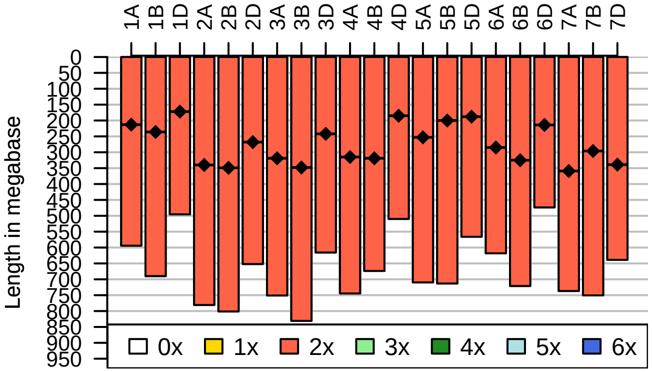

18SI−531−6 Euploid

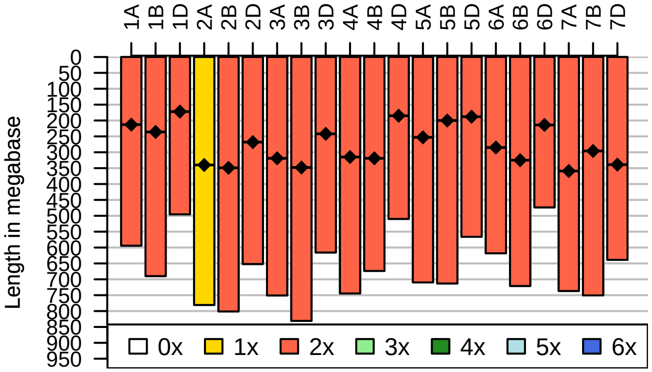

18SI−531−7 42

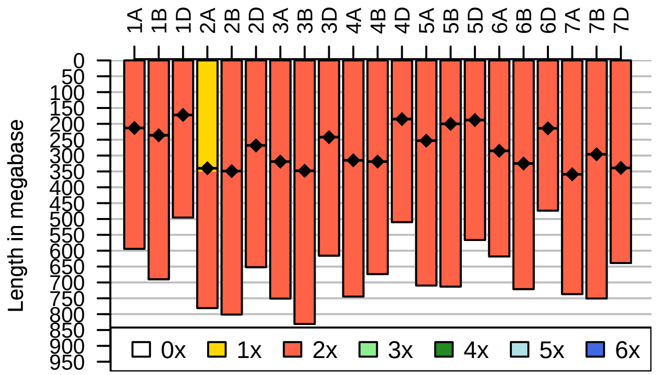

18SI−531−8 41+t

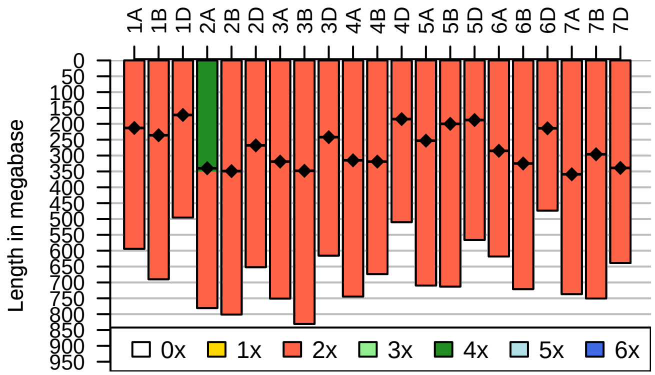

18SI−531−9 42+2A

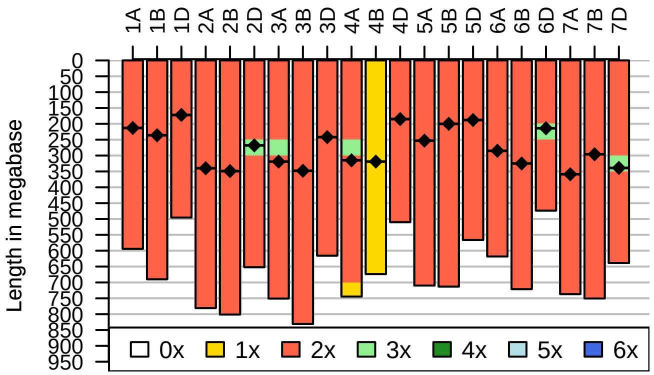

18SI−532−1 4B

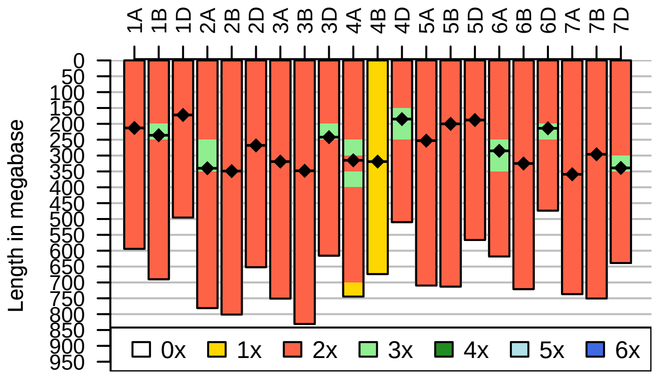

18SI−532−10 4B

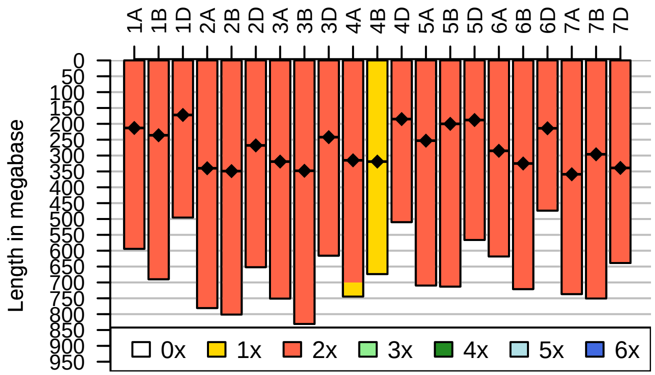

18SI−532−11 NA

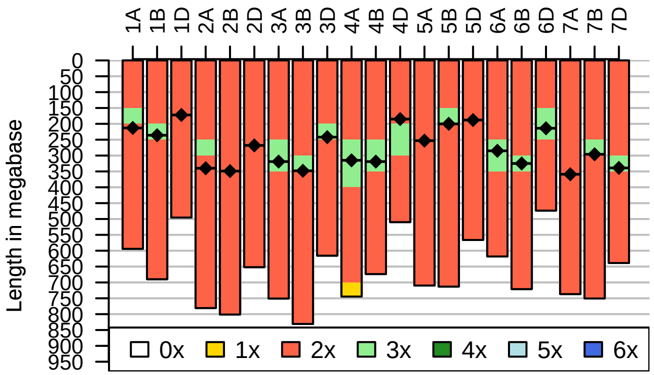

18SI−532−12 NA

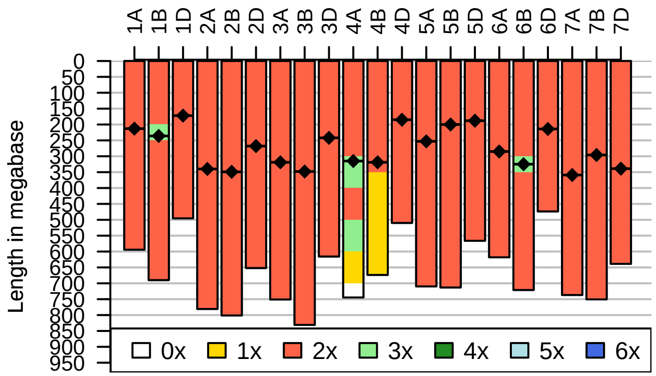

18SI−532−13 NA

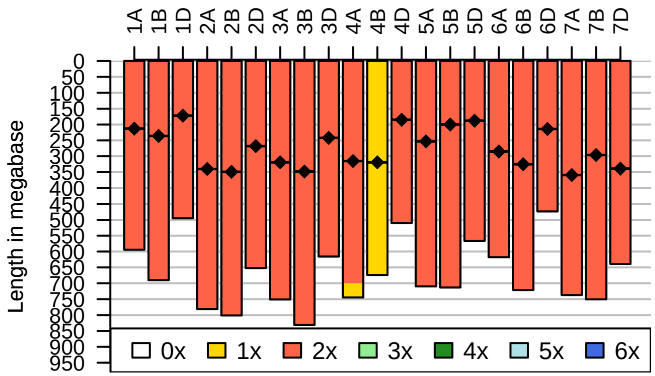

18SI−532−16 NA

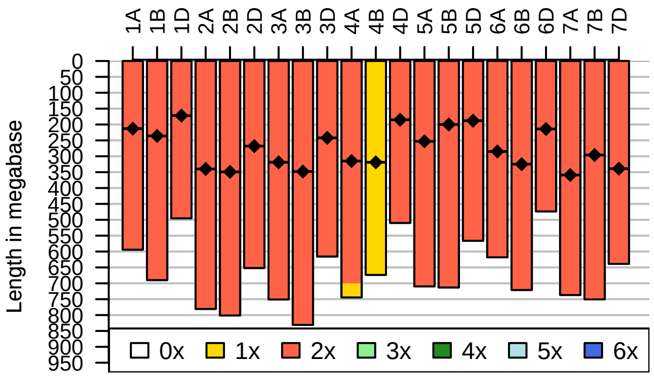

18SI−532−18 NA

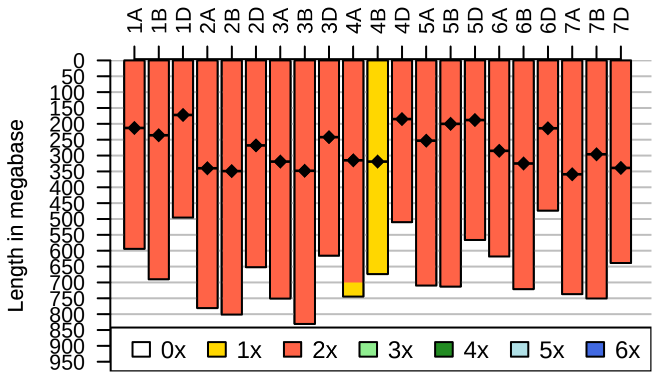

18SI−532−19 NA

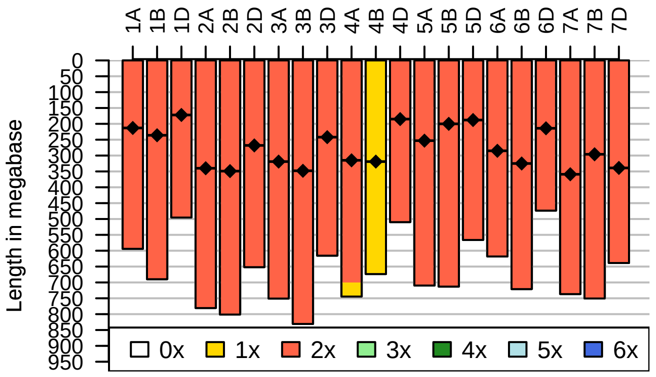

18SI−532−2 4B

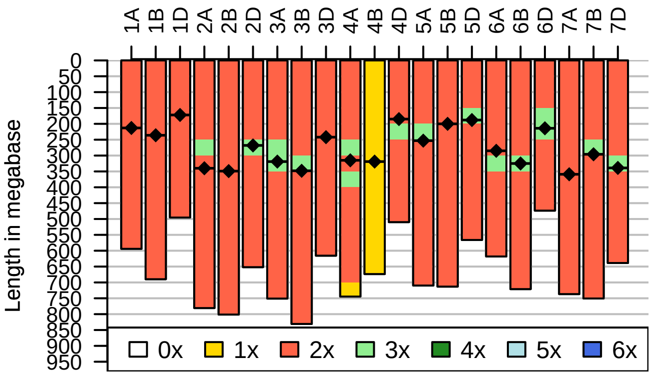

18SI−532−3 NA

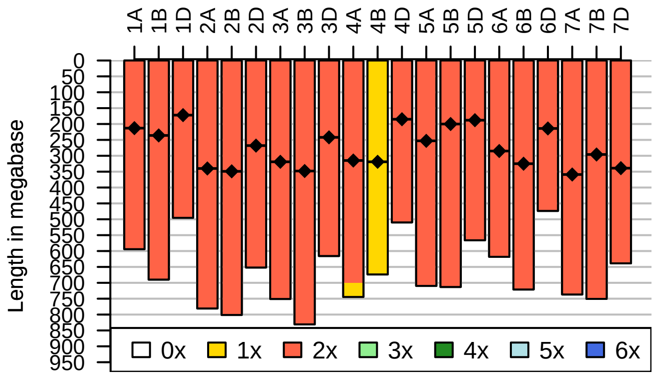

18SI−532−4 NA

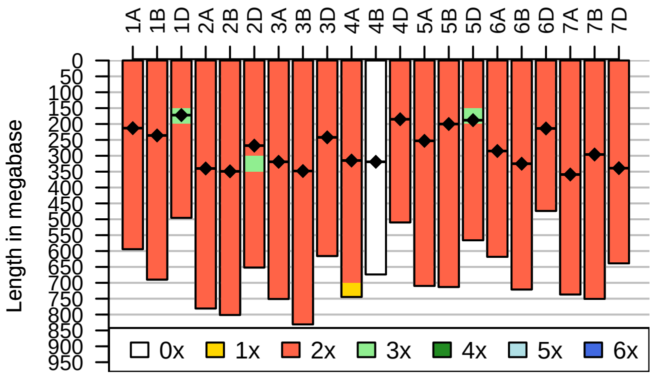

18SI−532−6 NA

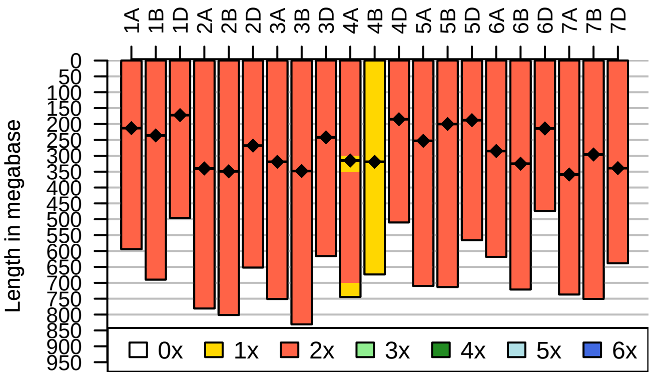

18SI−532−7 NA

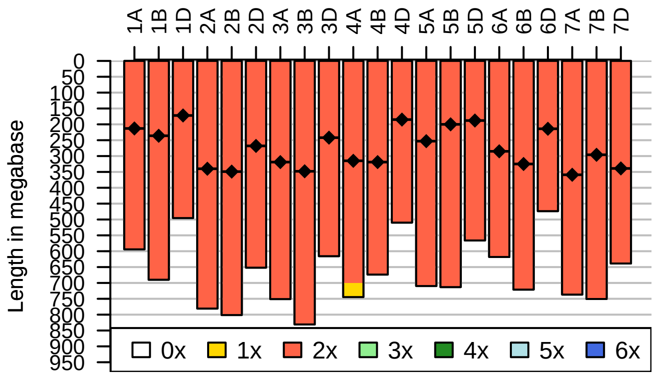

18SI−532−8 Euploid

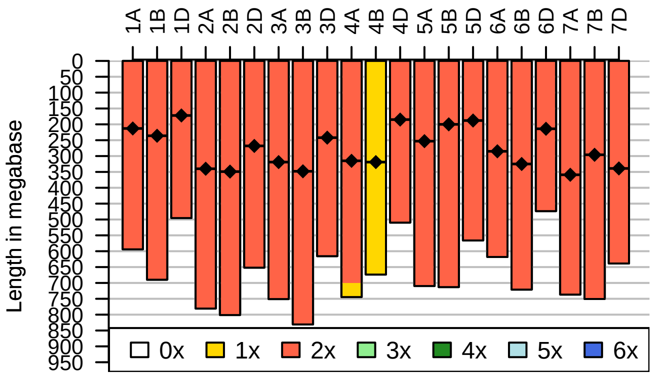

18SI−532−9 NA

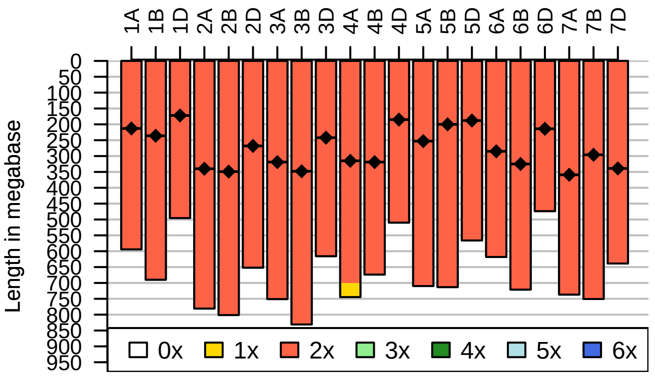

18SI−533−1 NA

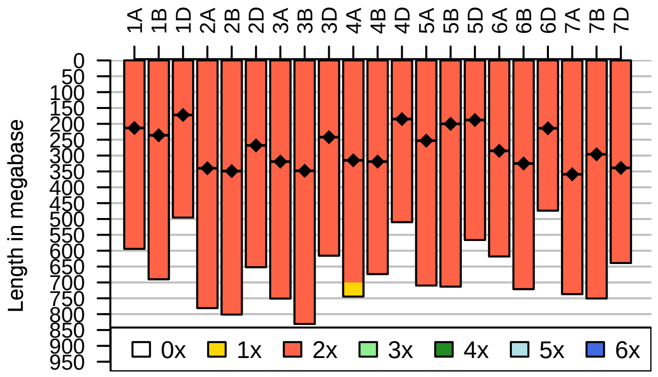

18SI−533−2 Euploid

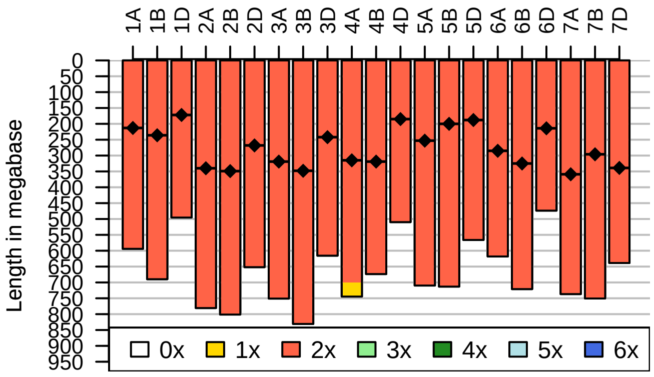

18SI−533−3 NA

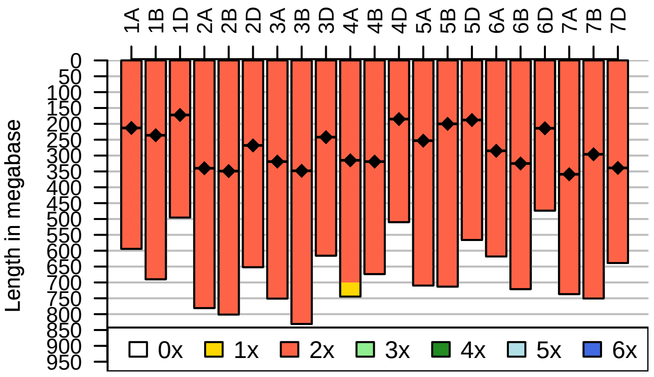

18SI−533−4 NA

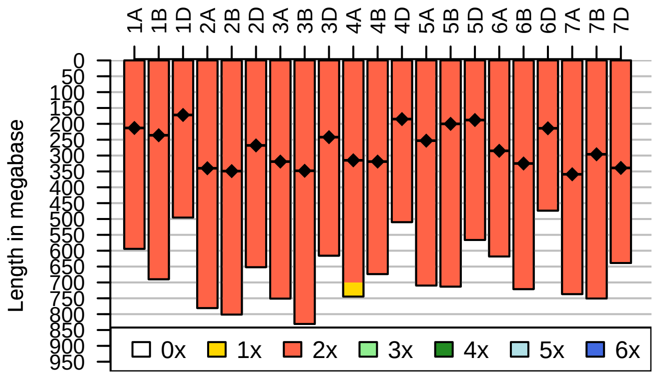

18SI−533−5 NA

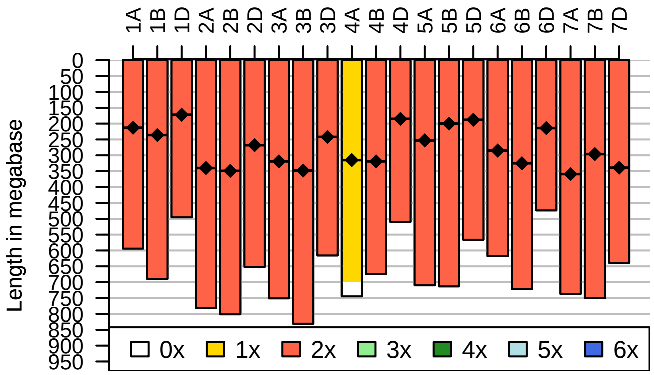

18SI−533−6 NA

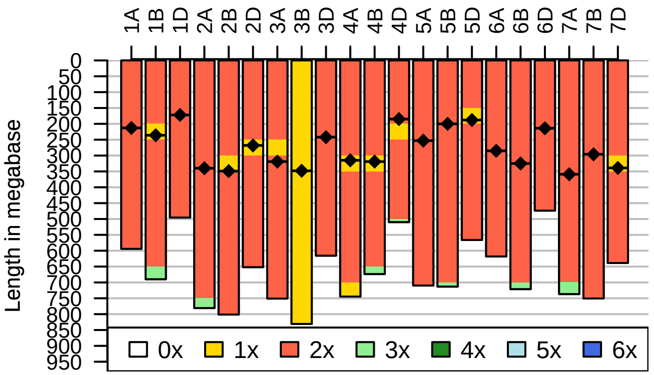

18SI−533−7 NA

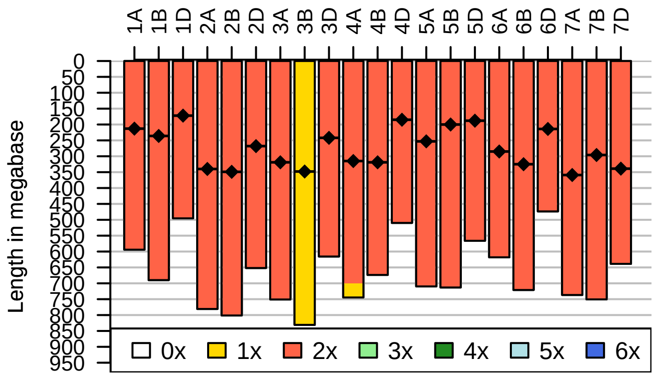

18SI−533−8 NA

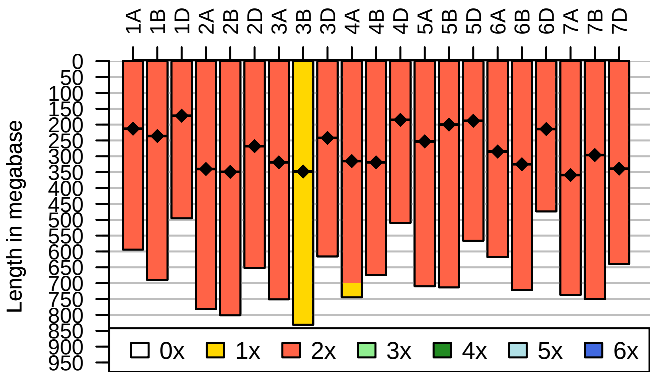

18SI−533−9 NA

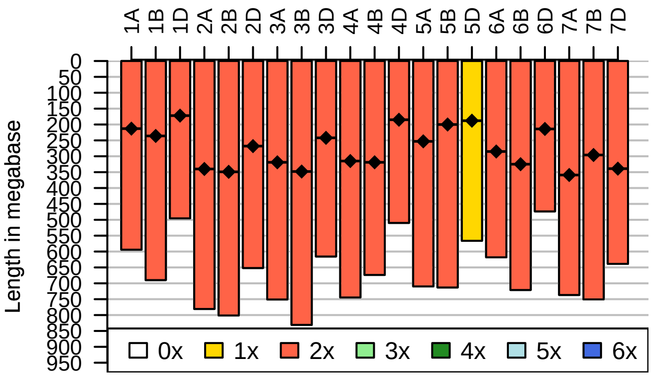

18SI−534−1 NA

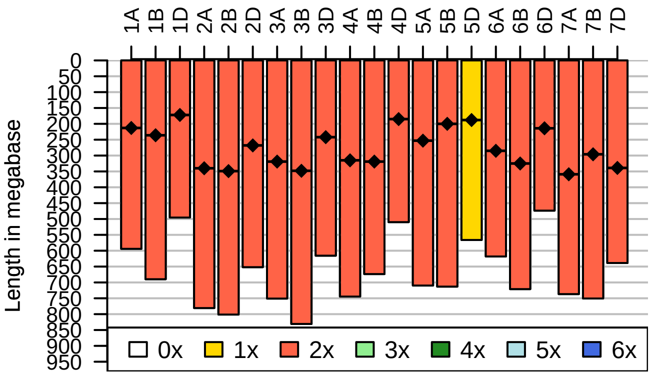

18SI−534−10 NA

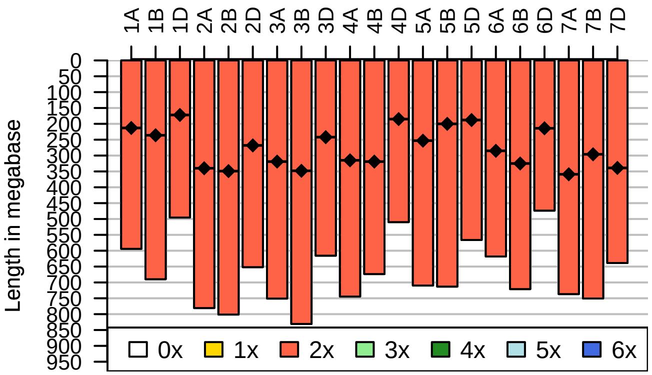

18SI−534−2 Euploid

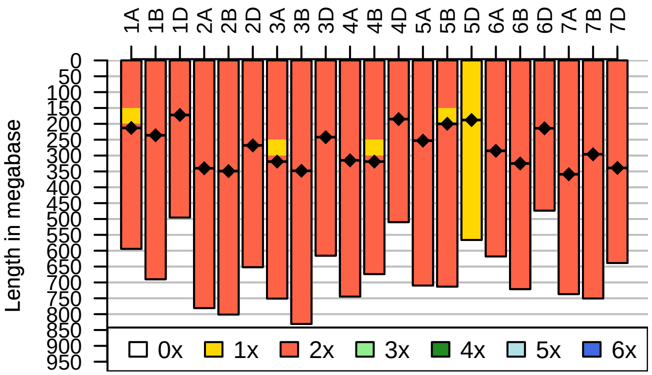

18SI−534−3 NA

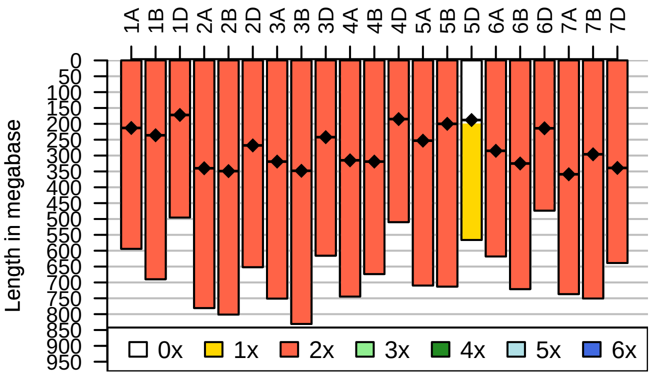

18SI−534−4 NA

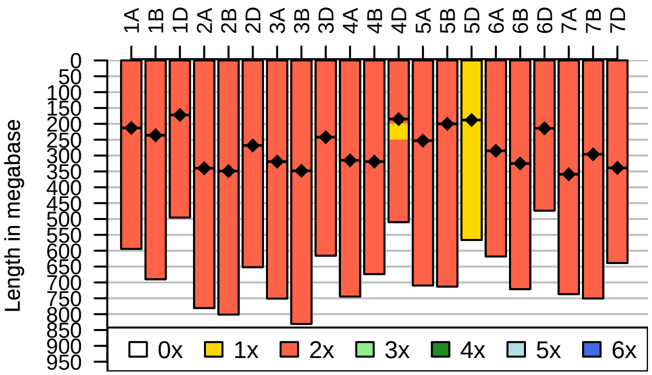

18SI−534−5 5D

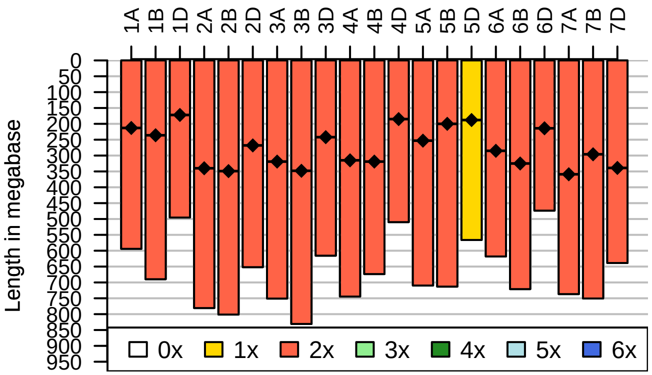

18SI−534−6 NA

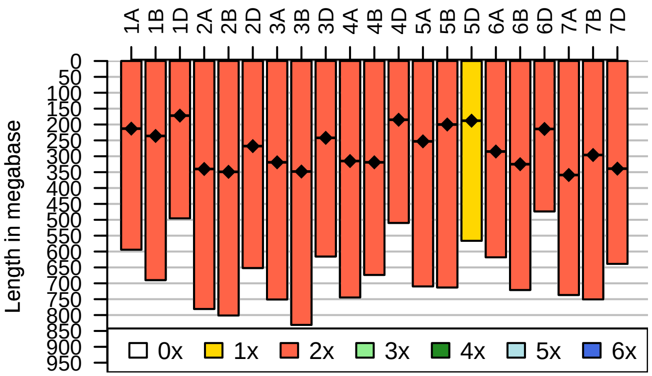

18SI−534−7 NA

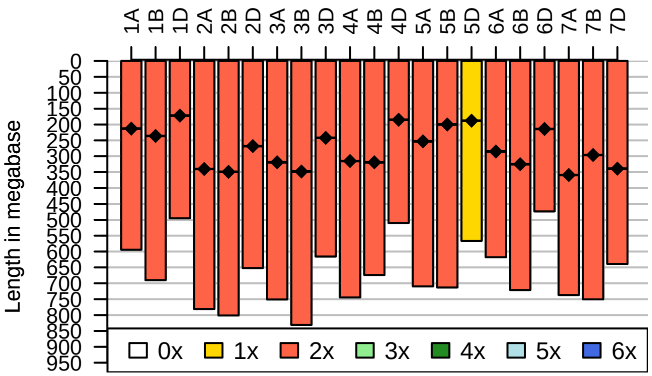

18SI−534−8 NA

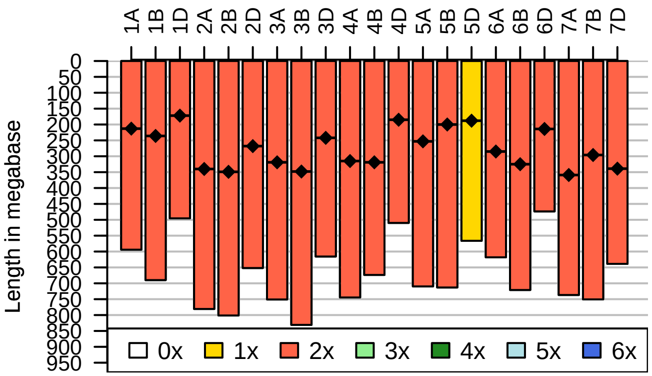

18SI−534−9 NA

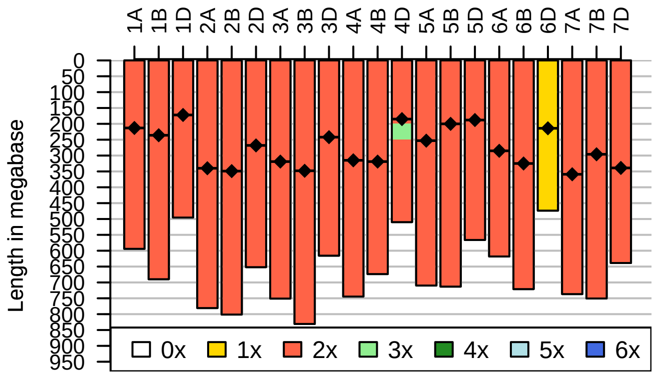

18SI−535−1 6D

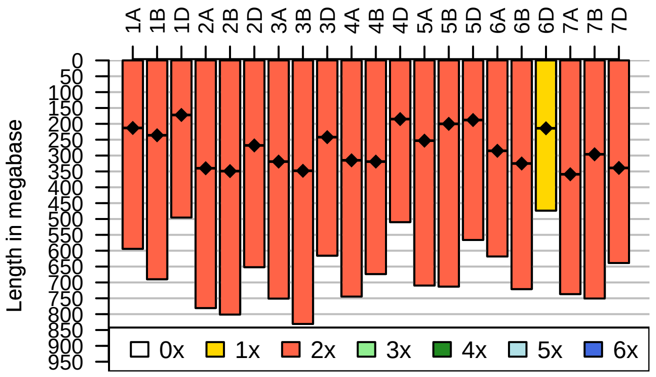

18SI−535−10 6D

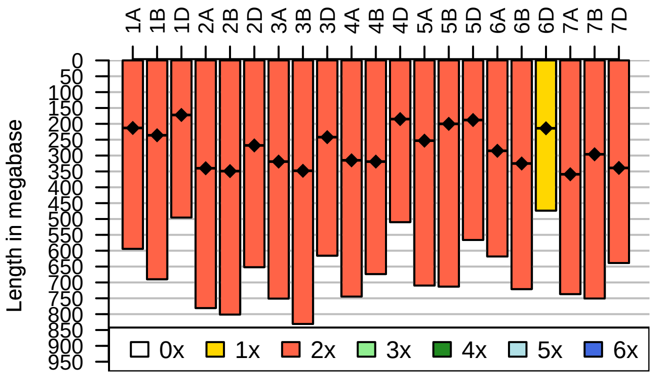

18SI−535−11 NA

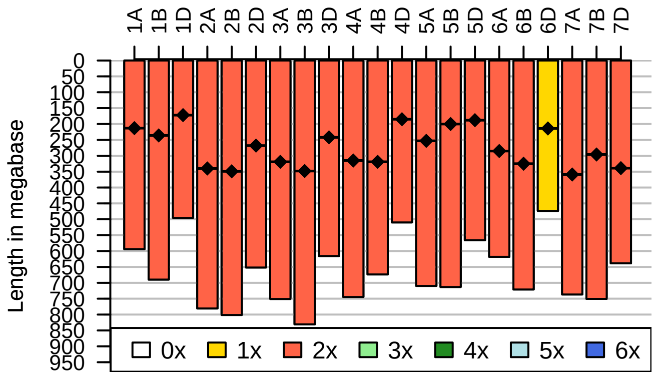

18SI−535−12 NA

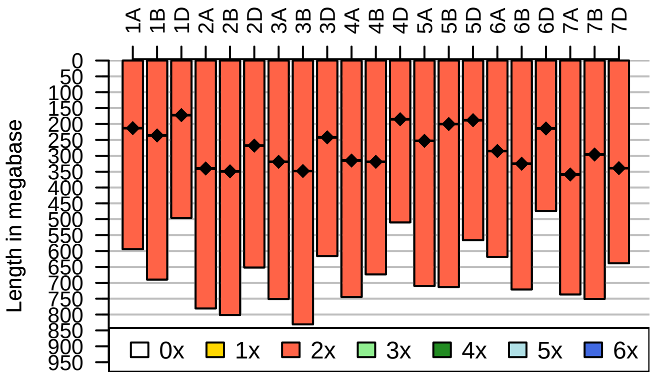

18SI−535−13 NA

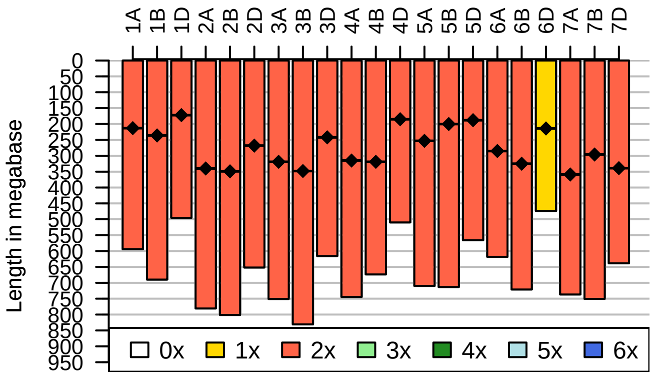

18SI−535−14 NA

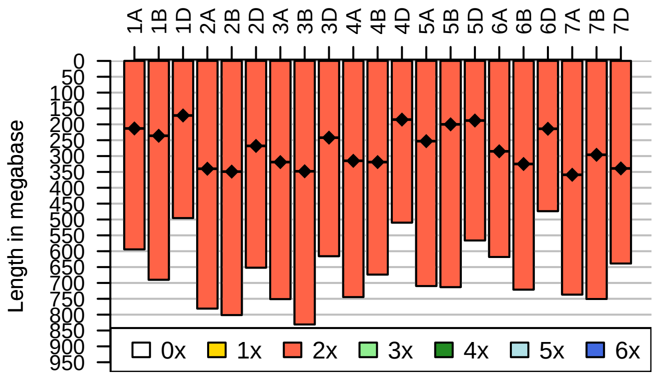

18SI−535−15 NA

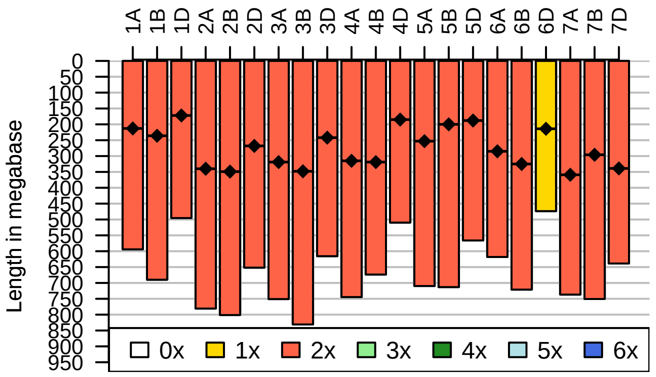

18SI−535−16 NA

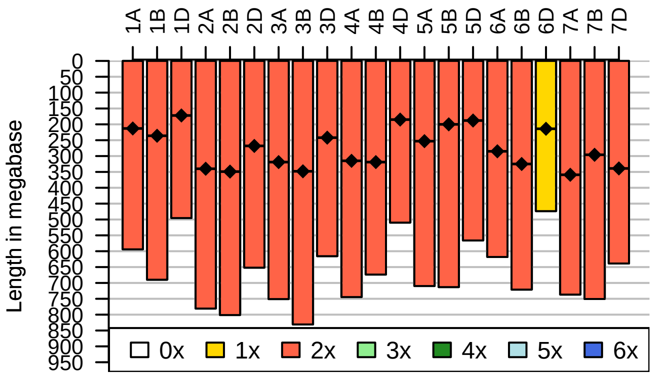

18SI−535−17 NA

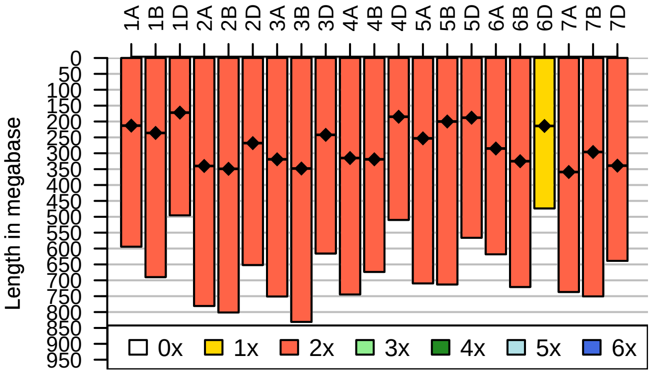

18SI−535−18 NA

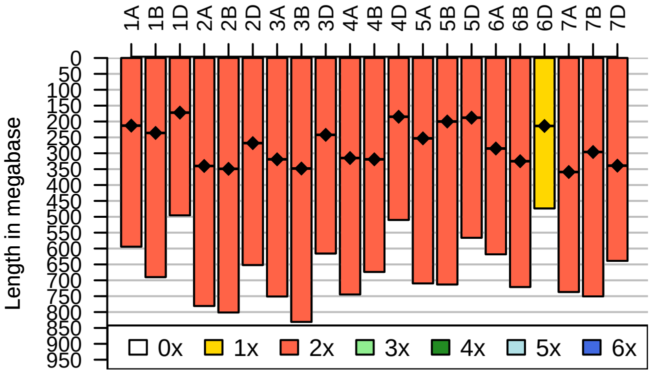

18SI−535−19 NA

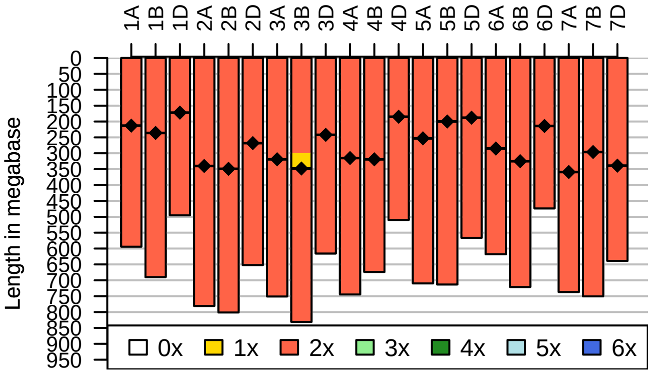

18SI−535−2 41

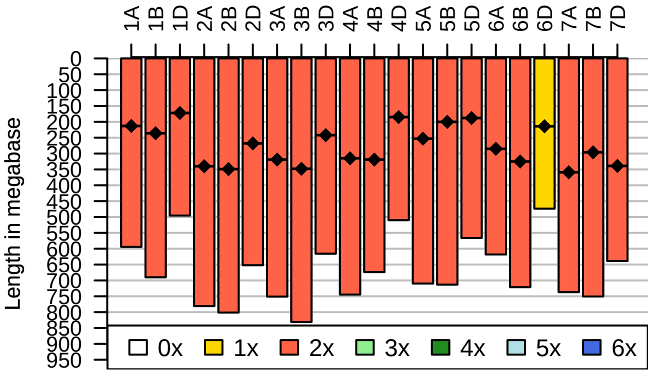

18SI−535−20 NA

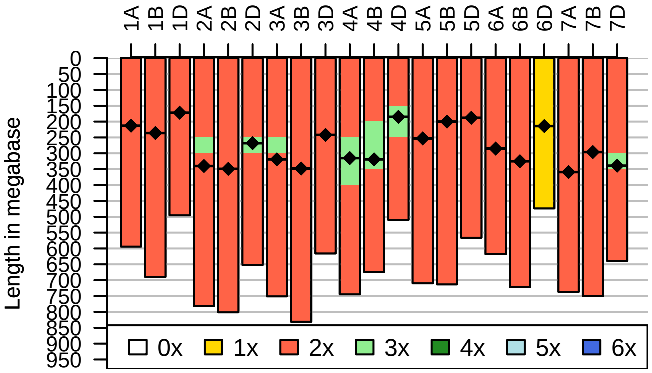

18SI−535−3 42

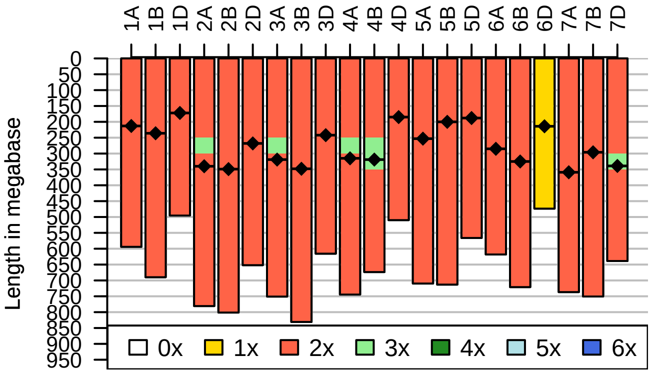

18SI−535−4 41+t

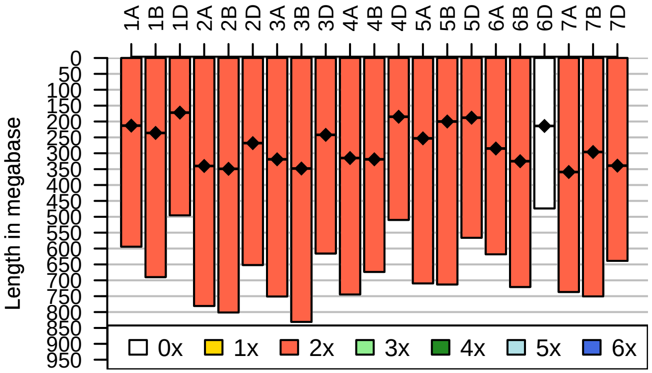

18SI−535−5 42

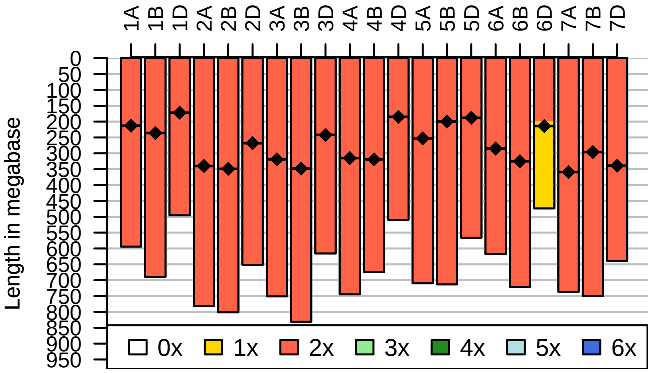

18SI−535−6 41

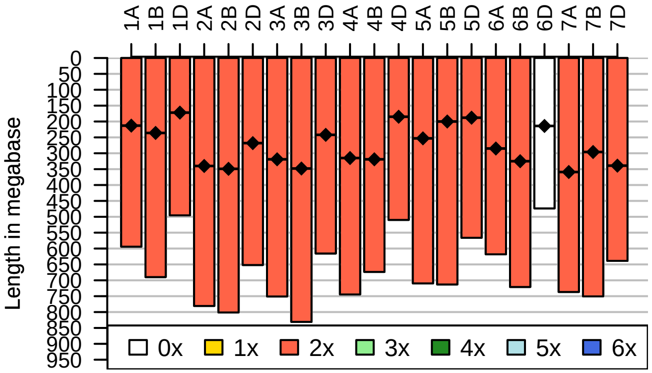

18SI−535−7 NA

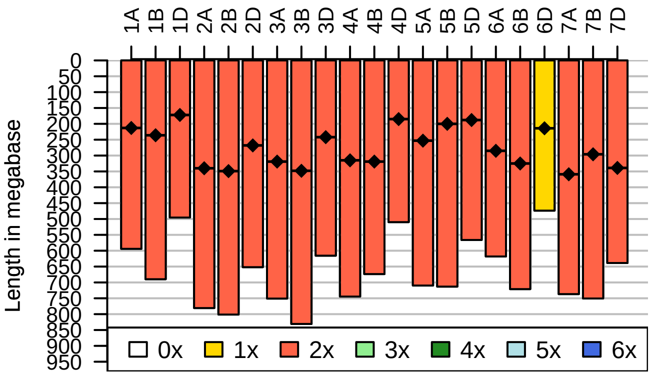

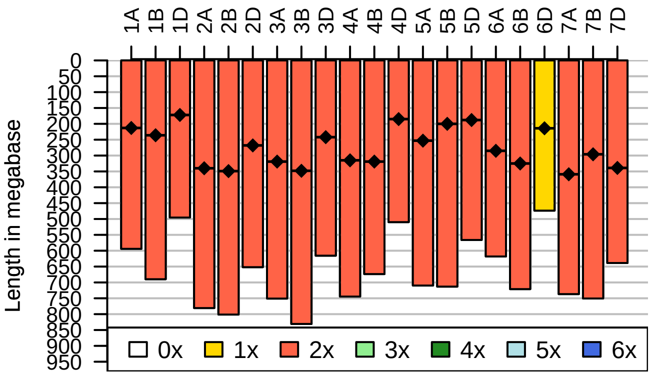

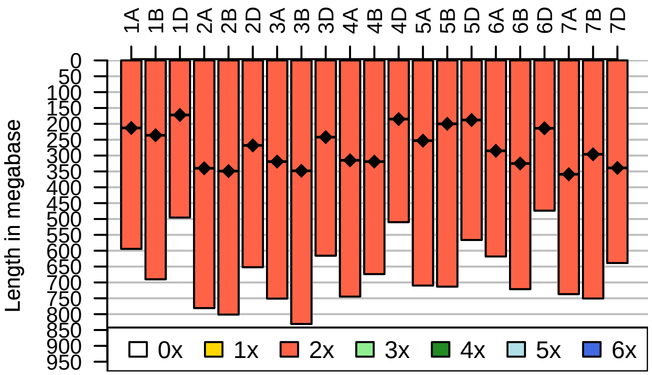

18SI−535−8 6D

18SI−535−9 6D

CS NA
